# Akamptisomerism as a Switching Element: Substituent Effects on Bond Angle Reflection and Photophysical Properties of B–O–B Porphyrins

**DOI:** 10.1021/acs.inorgchem.5c01132

**Published:** 2025-06-03

**Authors:** Karine N. de Andrade, Natalia M. Raffaeli, Rodolfo G. Fiorot

**Affiliations:** † Department of Organic Chemistry, Institute of Chemistry, 28110 Universidade Federal Fluminense (UFF), Outeiro de São João Batista, Niterói, Rio de Janiero 24020-141, Brazil

## Abstract

Developing novel molecular switches requires structural modifications that yield isomers with distinct photophysical properties. Akamptisomerism — a bond angle reflection (BAR) process occurring in low-symmetry porphyrins bridged by (F)­B–O–B­(F) units — induces distortions from the porphyrin pseudoplane, offering a promising strategy for optoelectronic switching. Herein, we report the first quantum chemical investigation of BAR in β- and *meso*-substituted porphyrinoid systems as a potential switching mechanism. Density functional theory (DFT) calculations (B3LYP-D3/def2-QZVP//B3LYP-D3/6–31+G**) confirm the occurrence of *transoid* (***t***)-akamptisomerism in all evaluated compounds, featuring a thermal interconversion barrier (***t***_**1**_ → ***t***_**2**_) of 26.6 ± 2.1 kcal mol^–1^ that is largely unaffected by porphyrin substitution across 28 compounds. Simulated UV–vis spectra (TD-CAM-B3LYP/6–31+G**) reveal that β,β-push–pull systems bearing −NMe_2_ and −NO_2_ groups on opposite pseudoplanes, combined with *meso*-^*t*^Bu substitution, exhibit significant spectral differentiation between akamptisomers (Δ*E* ***t***_**1**_/***t***_**2**_ = 320.6 meV, at the S_3_ state). This shift arises from intramolecular charge transfer between pseudoplanes and steric distortion induced by the −^*t*^Bu group. These findings establish akamptisomerism as a viable platform for constructing molecular building blocks with distinct optical signatures. Moreover, they underscore how substitution patterns can be exploited to tune the photophysical properties of BAR systems, providing valuable insights for the rational design of next-generation switchable materials.

## Graphical abstract

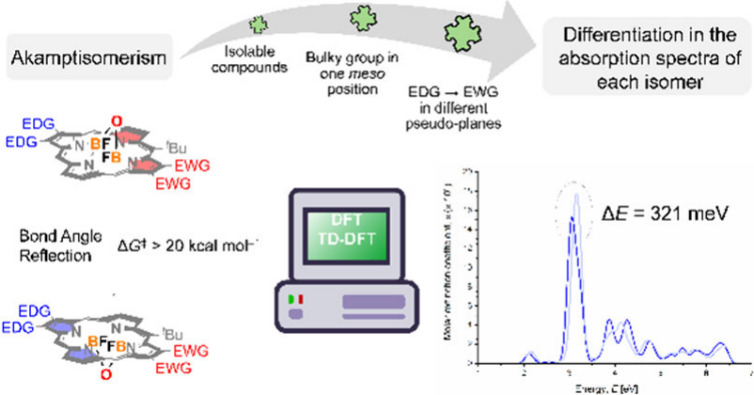

## Introduction

The interest in molecular switches has grown exponentially in recent years.^1–4^ These systems are molecules designed to respond to external stimuli that promote interconversion between reversible and isolable states.^2,5^ This interconversion occurs through structural reorganizations driven by so-called switching elements.^6^ When such changes lead to variations in optical properties, switching can be triggered by either an external electric field (electro-optical switching) or by light (photoswitching).^6–8^ The latter is well established and typically involves transformations around a double bond, such as *E*/*Z* isomerization, or ring-closing and ring-opening reactions (Scheme 1a).^6,9,10^ Additionally, restricted bond rotation in atropisomers has also been reported.^3,6,11,12^ In both electro-optical and photoswitchable systems, such structural modifications often produce species with distinct absorption profiles across the electromagnetic spectrum, commonly interpreted as “on” (active) and “off” (inactive) states.^2,10,13^

**Scheme 1:**
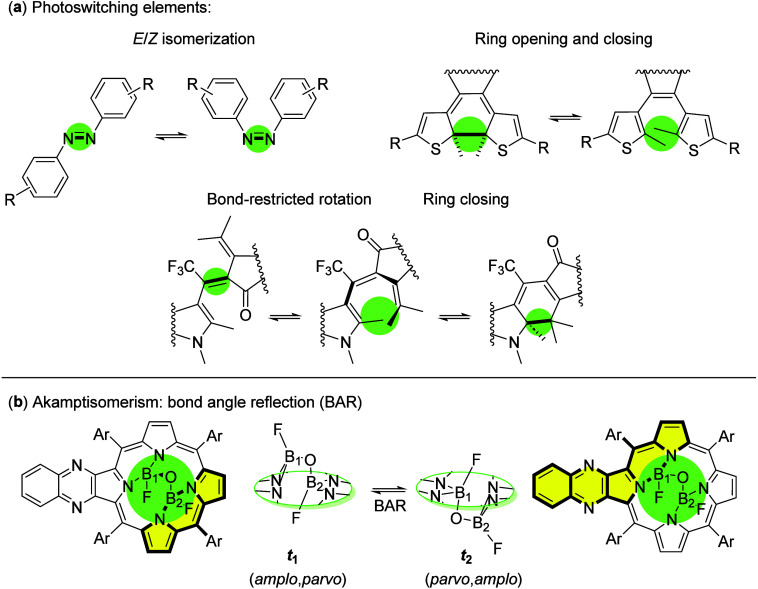
Isomerization Processes Identified in (a) Well-Known Photoswitches and (b) *transoid* (***t***) (F)­B–O–B­(F)-Quinoxalinoporphyrin[Fn sch1-fn1]

A The photoswitching element is highlighted in light green. The akamptisomers pair is shown with the pseudo-plane in bold yellow, illustrating the bond angle reflection from ***t***_**1**_ (*amplo*,*parvo*; B_**2**_ in the pseudoplane) to ***t***_**2**_ (*parvo*,*amplo*; B_**1**_ in the pseudoplane). Ar = 3,5-di-*tert*-butylphenyl.

Efforts to improve switching performance have traditionally focused on tuning the structure surrounding these well-known switching elements, particularly through the introduction of strategic substituents.^10,14^ According to our hypothesis, in addition to considering substituent effects around the switching element, it is crucial to examine the isomerization element itself — that is, the fundamental mechanism driving the process. In 2018, Canfield and co-workers introduced a novel type of conformational isomerism based on hindered bond angle reflection (BAR), termed akamptisomerism (from the Greek *akamptos*, meaning “not flexible”).^15^ Initially described as BAI (Bond Angle Inversion), the phenomenon was later refined to the mathematically more accurate term, Bond Angle Reflection.^16^

In this seminal work, they resolved the structure of four stereoisomers containing a B­(Y)–O–B­(Y) bridge anchored to a quinoxalinoporphyrin macrocycle, referred to as B_2_OY_2_, where Y = F. Specifically, two pairs of enantiomers were identified with a *transoid* (***t***) configuration, in which fluorine atoms are located on opposite sides of the porphyrin macrocycle (Scheme 1b). The hypothetical *cisoid* (***c***) configuration, in which both BF groups are on the same side of the pseudoplane, has not been experimentally observed for porphyrins. This isomerism exhibits a diastereoisomeric relationship arising from reflection across the B–O–B bond angle, leading to the formation of isomers with distinct physicochemical properties.^15^ The anchoring macrocycle adopts a stepped shape, depending on whether the BF groups are located in- or out-of-plane.^20^ During the BAR process, this stepped geometry alternates, inducing distortions in the pseudoplanes of the low-symmetric porphyrin through the pyrrole rings,^15^ thereby altering the overall macrocycle planarity (Scheme 1b, yellow highlights). Due to the novelty of this process, Canfield and co-workers proposed new stereodescriptors based on the boron centers: *amplo* and *parvo*, which respectively correspond to the BF group being located out of and within the porphyrin pseudoplane.^15^ In the scheme, the akamptisomer with (*amplo,parvo*) configuration is referred to as ***t***_**1**_ (*transoid* 1), while the isomer with (*parvo,amplo*) is referred to as ***t***_**2**_.

The insertion of (F)­B–O–B­(F) bridges, their synthesis, and their effects on the geometric structure of various porphyrinoid systems have been extensively explored in the literature.^17–21^ However, despite the identification of this new form of isomerism in such systems, akamptisomerism itself has received limited attention. Diastereoisomeric relationships within akamptisomer pairs have been studied in different frameworks, particularly to elucidate isomeric preferences in BOPPY,^22^ porphycenes,^23^ phthalocyanines,^18^ and porphyrazines.^18^ To date, the only study involving the porphyrin backbone — aside from the seminal work by Canfield and co-workers^15^ — is an electronic structure analysis of four akamptisomers of quinoxalinoporphyrin–[60]­fullerene.^24^ Computational results at the BLYP-D3­(BJ)/def2-SVP level revealed that the nature of the bridge substituents and their configuration — either *cisoid* (***c***) and *transoid* (***t***) — strongly influence the donor–acceptor characteristics of the system.^24^

Porphyrinoid macrocycles possess electronic properties that make them promising candidates for a range of optical applications, including solar cell sensitizers and photoresponsive therapeutics.^25^ With regard to their switchable behavior, such systems have been explored under various external stimuli, such as chemical and electrochemical inputs as well as electric fields.^26–28^ In this context, bond angle reflection (BAR) involving the (F)­B–O–B­(F) bridge leads to significant changes in molecular planarity, thereby modulating π-electron conjugation and the overall electronic structure. We hypothesize that such structural rearrangements may markedly affect the photophysical properties of individual akamptisomers, underscoring the potential of BAR as a mechanism for electro-optical molecular switching. Although photoswitching remains a widely adopted strategy in the design of responsive molecular systems, the present study focuses on electro-optical switching via BAR, without excluding the possibility of future photochemical activation. This focus is motivated by recent observations that the *transoid* (F)­B–O–B­(F) bridge possesses a significant electric dipole oriented approximately normal to the porphyrin plane, suggesting that the akamptisomeric state can be controlled using an external electric field.^29^

Herein, we present the first systematic computational investigation of the bond angle reflection process and its associated photophysical profiles. By exploring substituents with varying electronic characteristics and steric demands, we evaluate their impact on the bond angle reflection mechanism and the electronic properties of each akamptisomer. Computational chemistry plays a vital role in modern materials design, offering precise insights into structural phenomena such as molecular isomerism,^30^ often in synergy with experimental studies.^31,32^ Through the simulation of key properties, such as UV–vis absorption spectra across different systems, computational tools not only reduce experimental waste but also accelerate the development of innovative materials with broad technological potential.^32,33^ This fully theoretical and computational design-based approach aims to support the rational development of novel materials with desirable functional properties.

To this end, we employed density functional theory (DFT) and its time-dependent extension (TD-DFT) to investigate how structural variations in porphyrin macrocycles (Figure 1) affect akamptisomerism and the associated optical properties, particularly the absorption profiles of the *transoid* akamptisomer pairs (***t***_**1**_ and ***t***_**2**_). These variations were based on the (F)­B–O–B­(F)-porphyrin framework previously studied by Canfield and co-workers,^15^ and include the strategic integration of substituents bearing *n* and π electrons at the β,β, and *meso* positions (see Figure 1). In *variation 1*, extended π-conjugated systems were attached to the pyrrolic units. This set includes: a simple carbocycle (**1a**) for baseline comparison; a quinoxaline heterocycle (**1b**, previously explored by Canfield and co-workers^15^); a naphtho­(dione) (**1c**, electron-withdrawing); and a thiophene ring (**1d**, electron-donating). *Variations 2* and 3 examine the effects of electron-donating groups (EDGs, **a**–**d**) and electron-withdrawing groups (EWGs, **e**–**g**) at the β,β-pyrrolic (vicinal pair) and *meso* (single) positions, respectively. These modifications are key for future analyses involving push–pull systems.^34^ Lastly, *variation 4* introduces aliphatic groups at the *meso* position (single substitution, **4a**–**4e**) to evaluate how increasing steric hindrance affects the isomerization barrier.

**Figure 1:**
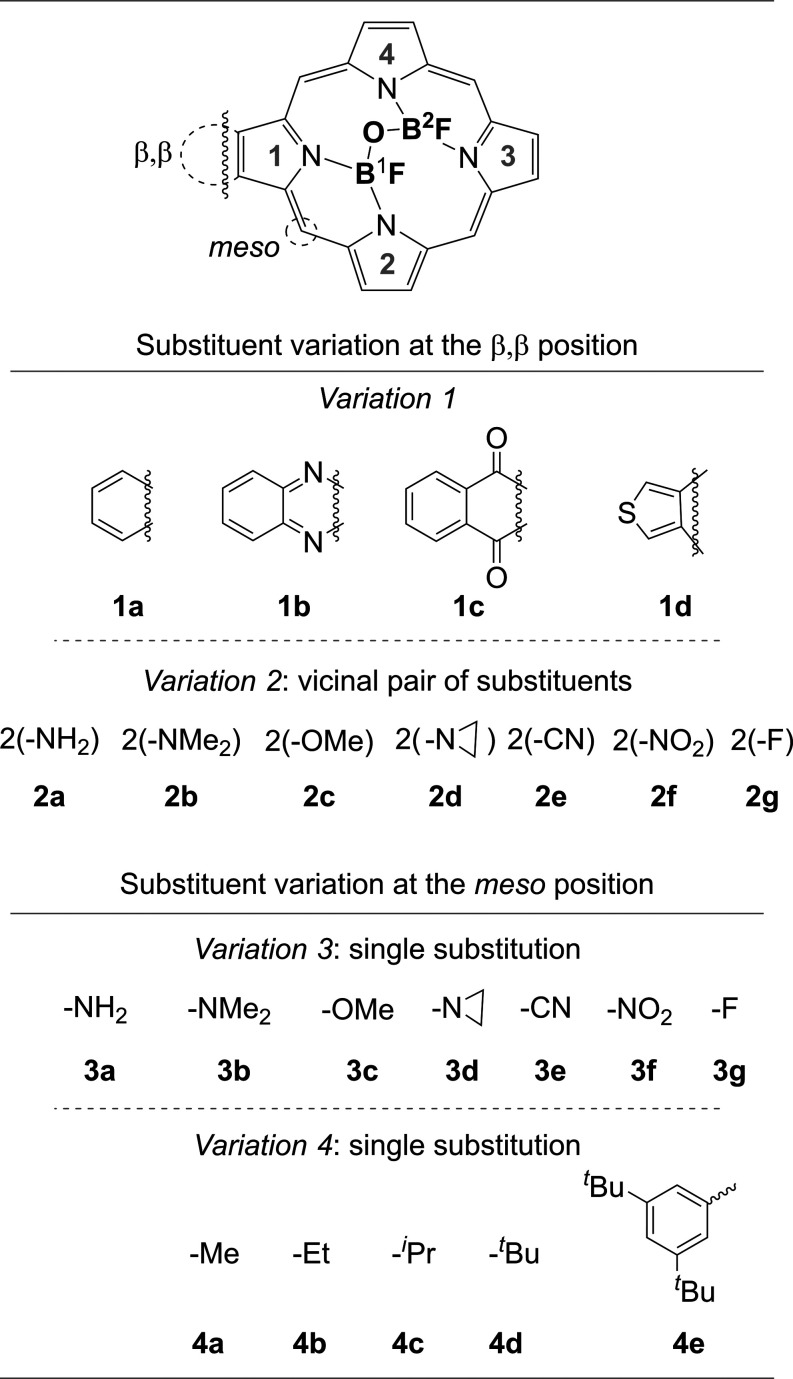
Structural variations OF THE (F)­B–O–B­(F)-porphyrin framework in β,β- and *meso*-positions.

## Computational Methodology

All calculations were performed using the Gaussian 09 software package.^35^ Ground-state structures of the akamptisomers were optimized using density functional theory (DFT) with the hybrid exchange-correlation functional B3LYP^36–38^ combined with the 6–31+G** ^39,40^ basis set. To account for long-range dispersion interactions in the highly conjugated porphyrin macrocycles, Grimme’s D3 empirical dispersion correction^41^ was included in all geometry optimizations. This level of theory was selected based on prior studies of bond angle reflection mechanisms and related processes, which demonstrated reliable performance for systems containing B_2_OF_2_ bridges.^15^ Vibrational frequency calculations were carried out to confirm the nature of the stationary points: minimum energy structures (i.e., akamptisomers) were confirmed by the absence of imaginary frequencies, while first-order saddle points (i.e., transition states, TS) were confirmed by the presence of a single imaginary frequency. Thermodynamic corrections to the electronic energies were obtained from standard statistical thermodynamics calculations implemented in Gaussian 09, yielding Gibbs free energies at 298 K and 1 atm.^42^ To refine the electronic energies, single-point calculations were performed at the def2-QZVP basis set level.^43^ This dual-level approach, frequently adopted in the literature,^44^ enables efficient optimization at moderate cost while obtaining accurate relative energies at a higher level of theory — a necessary step for a robust description of electronic structures and substituent effects in material design.

Photophysical properties were investigated using time-dependent density functional theory (TD-DFT)^45,46^ applying the TD-CAM-B3LYP/6–31+G­(d,p) level of theory.^39,40,47^ The CAM-B3LYP functional is particularly suitable for simulating electronic spectra of porphyrins due to its range-separated formalism, which allows for a more accurate description of long-range polarization and intramolecular charge-transfer transitions.^48,49^ These features are particularly relevant due to the highly conjugated nature of porphyrins.^50,51^ Simulated UV–vis absorption spectra were obtained from singlet–singlet vertical excitations, considering 80 excited states per system (*nstates = 80*, singlets). The analysis of the molecular orbitals involved in the relevant transitions was conducted at the same level of theory using full population analysis.^52^

UV–vis spectra were generated in terms of molar extinction coefficient (ε) versus energy (eV), employing a Gaussian band-shape function. The contribution of each electronic excitation (*i*) was calculated as

1 $$\varepsilon_{i}\left(ṽ\right)=1.3062974\times 10^{8}\frac{f_{i}}{\sigma }\exp\left[-{\left(\frac{ṽ-{ṽ}_{i}}{\sigma }\right)}^{2}\right]$$

Where *ṽ*_*i*_ is the excitation energy (in wavenumber, cm^–1^), *f*_*i*_ the oscillator strength, σ is the standard deviation (in cm^–1^), and *ṽ* the frequency of the incident radiation. The overall absorption spectrum is the sum of individual transitions:

2 $$\varepsilon \left(ṽ\right)=\overset{n}{\underset{i=1}{\sum }}\varepsilon_{i}\left(ṽ\right)$$

This method follows the Gaussian broadening scheme. The value of σ = 800 cm^–1^ (99.1874 meV) was selected to best reproduce the spectral profile of the unsubstituted porphyrin, used as a reference. For a detailed derivation and explanation of this approach, we refer the reader to the Gaussian manual.^53^

## Results and Discussion

The diboron complexes formed by the insertion of a B_2_OF_2_ bridge into low-symmetric porphyrins give rise to eight possible stereoisomers. These isomers differ based on the boron complexation site and the relative positioning of the fluorine atoms — either on the same face (*cisoid*) or on opposite faces (*transoid*) of the macrocycle. For a detailed visual and conceptual explanation of all possible anchoring configurations, we refer the reader to the seminal work of Canfield and co-workers,^15^ which offers a comprehensive and elegant discussion of the stereochemistry of such species.

In general, the possible complexation modes generate pairs of enantiomers: *R,R*; *S,S*; *R,S*; and *S,R*, with the boron atom as the stereogenic center. When both fluorine atoms lie on the same face of the macrocycle, the configuration is classified as *cisoid* (*R,S* and *S,R* centers), whereas when they are positioned on opposite faces, the configuration is *transoid* (*R,R* and *S,S* centers). The eight possible stereoisomers emerge due to bond angle reflection at the bridging oxygen atom. Since enantiomers share identical physicochemical properties (differing only in their interaction with polarized light), we arbitrarily selected one representative from each pair for computational evaluation — specifically, the *R*,*R* (*transoid*, ***t***) and *R*,*S* (*cisoid*, ***c***) for the porphyrins.

In the Supporting Information (SI) file, a detailed description of the protocol used to assign the recently proposed stereodescriptors (*amplo* or *parvo*) to each boron center is provided, following the suggestions of Canfield and co-workers.^15^ For simplicity, throughout this work, *transoid* systems with the stereocenters in the (*amplo,parvo*) configuration are designated as ***t***_**1**_, while their isomeric form with (*parvo,amplo*) centers is labeled ***t***_**2**_. For *cisoid* configuration, ***c***_**1**_ refers to (*amplo*,*amplo*) centers, and ***c***_**2**_ to (*parvo*,*parvo*).

### Akamptisomerism: Bond Angle Reflection

The energy profile for the bond angle reflection (BAR) was mapped for different substituted porphyrins to understand the relative stability of the *cisoid* (***c***) and *transoid* (***t***) akamptisomer pairs. Structural diversity was introduced through substitutions at the β,β-pyrrole (*variations 1–2*), and *meso* (*variations 3–4*) positions, resulting in a total of 23 systems, each with four evaluated stereoisomers (***c***_**1**_, ***c***_**2**_, ***t***_**1**_, ***t***_**2**_). For consistent reference, all substitutions (β,β-pyrrole, and *meso*) are located on the left side of the porphyrin ring (Figure 1, highlighted β,β and *meso* portions), and energy differences were calculated relative to akamptisomer ***t***_**1**_.

Scheme 2 illustrates the overall energy profile identified for the direct BAR process, showing the changes in the (F)­B–O–B­(F) bridge throughout the isomerization pathway for *transoid* and *cisoid* configurations. A statistical analysis was performed across all the 23 systems, from which the corresponding mean values and mean absolute deviations were obtained for the Gibbs free energy change of the *transoid* BAR process (***t***_**1**_ **→** *t*_**2**_) and the relative energy of the *cisoid* (***c***_**1**_) isomer. In agreement with Canfield and co-workers,^15^ ***c***_**2**_ could not be located as a minimum energy point on the potential energy surface (PES), and its optimization converged to ***c***_**1**_. Alternative processes that could allow the interconversion between akamptisomers ***t***_**1**_ and ***t***_**2**_, such as combined torsions of the B–O bond — as previously explored by Canfield and co-workers^15^ — were not reassessed here, given that the constituents of the anchored bridge remain unchanged.

**Scheme 2:**
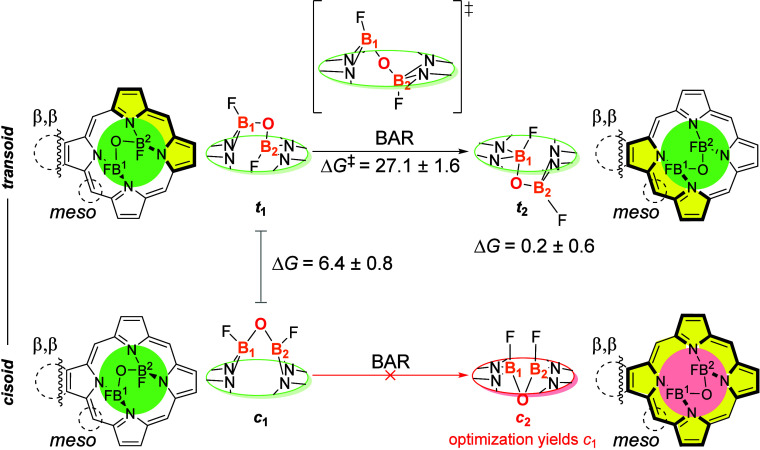
Overall Gibbs Free Energy Change^a^ for the Bond Angle Reflection (BAR) for the *transoid* ***t***_**1**_ → ***t***_2_ and *cisoid* ***c***_**1**_ → ***c***_**2**_ Obtained at the B3LYP-D3/def2-QZVP//B3LYLP-D3/6-31+G** Level Relative to ***t***_**1**_ ^b^

a Mean and mean absolute deviations values in kcal mol^–1^.
b Optimization of ***c***_**2**_ converges to ***c***_**1**_. Pyrrolic units belonging to the same pseudoplane are highlighted in yellow.

Notably, *transoid* ***t***_**1**_ configurations are consistently more stable than their *cisoid* ***c***_**1**_ counterparts by 6.4 ± 0.8 kcal mol^–1^ across all assessed systems, while being nearly isoenergetic with their ***t***_2_ akamptisomer (0.2 ± 0.6 kcal mol^–1^). The small mean absolute deviation (MAD) values (0.8 and 0.6 kcal mol^–1^) indicate that the preference for ***t***_**1**_ over ***c***_**1**_ and the energetic similarity between the two *transoid* akamptisomers are minimally influenced by substituents at the β,β- and *meso* positions. The computed preference for *transoid* versus *cisoid* stereochemical aligns with experimental observations for porphyrin diboron complexes obtained from the reaction of BF_3_•OEt_2_ with free-base porphyrin.^54^ According to the literature, the main factor favoring the *transoid* configuration in porphyrinoid macrocycles is the tetragonal distortion of the *N*_4_ cavity.^18,54^ The *cisoid* ***c***_**2**_ isomer, characterized by a fully planar geometry, does not correspond to a minimum energy point on the potential energy surface. This behavior is consistent with previous reports for a **1b**-*like* system containing 2,5-dibutyl-phenyl substituents in the *meso* positions, geometries optimized from ***c***_**2**_ initial structures converged to their more stable pair (***c***_**1**_) without encountering an energy barrier.^15^ The instability of ***c***_**2**_ may be attributed to 1,3-diaxial repulsion between the fluorine atoms in the *cisoid* configuration (Scheme 2, ***c***_**2**_) and to the strain imposed by fitting two boron atoms within the macrocyclic plane. Investigations into the stereochemical preference in *cisoid* systems are currently underway by our research group.

With regard to the bond angle reflection process, we found that the akamptisomerism phenomenon is feasible for all evaluated *transoid* systems, as supported by the overall free energy barriers for thermal interconversion (***t***_**1**_→ ***t***_**2**_), calculated as Δ*G* ^‡^ = 27.1 ± 1.6 kcal mol^–1^. In this process, the oxygen atom of the B–O–B bridge adopts a nearly linear geometry in the transition state (TS) structure (*A*_BÔB_ ≈ 179°) for all systems. Since the energy barrier exceeds the 20 kcal mol^–1^ threshold, our findings suggest that the *transoid* isomers could, in principle, be isolated experimentally.^15,55^ The low MAD value (1.6 kcal mol^–1^) once again indicates that the substituent pattern has a negligible effect on the BAR barrier.

Table 1 presents the calculated free energy changes relative to the ***t***_1_ isomer for each system. Akamptisomerism is not accessible for *cisoid* configurations, as the ***c***_**2**_ isomer was not located as a minimum on the PES. The highest calculated BAR energy barrier was 27.7 kcal mol^–1^, for system **3g** (*meso* = F), while the lowest barrier was 25.1 kcal mol^–1^, for system **4e** (β,β = 3,5-di-*tert*-butylphenyl).

**Table 1:** Gibbs Free Energy Change (Relative to Isomer ***t***_1_) for the *transoid* ***t***_**1**_→ ***t***_**2**_ Bond Angle Reflection and Relative Energy of ***c***_**1**_ ^a^

	relative Gibbs free energy, kcal mol^–1^				relative Gibbs free energy, kcal mol^–1^		
	TS (***t***_**1**_ *→* ***t***_**2**_)	***t***_**2**_	***c***_**1**_ ^a^		TS (***t***_**1**_ *→* ***t***_**2**_)	***t***_**2**_	***c***_**1**_ ^a^
**1a** (benzo)	25.9	–0.3	6.3	**3a** (−NH_2_)	27.0	1.2	6.5
**1b** (quinoxalino)	26.4	0.1	6.8	**3b** (−NMe_2_)	25.8	0.3	6.0
**1c** (naphtho(dione))	25.2	–0.1	6.1	**3c** (−OMe)	27.1	1.1	6.5
**1d** (thieno)	25.4	–0.3	6.7	**3d** (−aziridinyl)	27.3	1.4	8.1
**2a** 2(−NH_2_)	26.4	–0.6	6.4	**3e** (−CN)	27.2	–0.1	5.9
**2b** 2(-NMe_2_)	27.4	0.1	5.2	**3f** (−NO_2_)	25.6	–0.3	6.2
**2c** 2(-OMe)	27.5	0.3	5.0	**3g** (−F)	27.7	0.8	6.1
**2d** 2(-aziridinyl)	26.9	–0.4	6.0	**4a** (−Me)	27.3	–0.1	5.9
**2e** 2(−CN)	27.5	0.4	6.4	**4b** (−Et)	27.3	–0.3	5.8
**2f** 2(-NO_2_)	27.1	0.3	6.4	**4c** (−^*i*^Pr)	26.9	0.2	6.0
**2g** 2(-F)	27.4	0.4	5.8	**4d** (−^*t*^Bu)	26.1	1.2	7.5
				**4e** (3,5-di-*tert*-butylphenyl)	25.1	–0.4	5.2

a Reported values in kcal mol^–1^, computed at B3LYP-D3/def2-QZVP//B3LYLP-D3/6-31+G&& level.

b Optimization of ***c***_**2**_ converges to ***c***_**1**_.

The only substituent-dependent trend identified was for aliphatic groups at the *meso* position (*variation 4*), where we observed that an increase in molecular volume leads to a slight reduction in the energy barrier (ΔΔ*G* ^‡^ = 2.2 kcal mol^–1^) when comparing the smallest system **4a** (*meso* = methyl) with the bulkiest, **4e** (*meso* = 3,5-di-*tert*-butylphenyl). This behavior is likely associated with destabilization of the minimum energy structure (***t***_**1**_) due to steric repulsion between the bulky *meso* substituent and the peripheral β,β-pyrrole hydrogens, thereby slightly lowering the conversion barrier.

The results presented in this section consistently demonstrate that the BAR process leads to isolable species in a diastereoisomeric relationship, with low variance in the energy profile despite the structural diversity introduced by β and *meso* substitutions. This suggests that akamptisomerism is primarily governed by the anchored (F)­B–O–B­(F) bridge and macrocycle size, rather than the identity of the substituents. As a result, the (F)­B–O–B­(F)-porphyrin framework emerges as a promising building block for the investigation of unusual isomerization processes and their potential applications.

One such application is its use in molecular switches that respond to external stimuli, functioning as a bistable element. In line with our goal of evaluating whether BAR in porphyrins can serve as a novel molecular optoelectronic switching mechanism, we next examined the photophysical properties of the individual ***t***_**1**_ and ***t***_**2**_ isomers, as discussed in the following section.

### Can We Plan Molecular Devices with Distinct Interaction with Light via Akamptisomerism?

An optoelectronic switching device must fulfill several essential requirements, with thermal stability and distinct photophysical signatures being particularly critical. Thermal stability refers to a high isomerization energy barrier that prevents rapid interconversion in the absence of light. Distinct photophysical profiles, on the other hand, enable the definition of active and inactive states at specific wavelengths, allowing for precise addressability through spectral band separation.^3,10,56^ In this section, we assessed whether the bond angle reflection (BAR) and the distortion of porphyrin pseudoplanes are sufficient to achieve such separation. To this end, the photophysical properties of the individual akamptisomers ***t***_1_ and ***t***_2_ were investigated through UV–vis absorption spectra simulations based on vertical electronic excitations at the CAM-B3LYP/6–31+G** level.

To assess the influence of substituents on the macrocyclic framework, we also performed calculations for the unsubstituted (F)­B–O–B­(F)-porphyrin, hereafter referred to as B_2_OF_2_–Por. The Supporting Information provides the complete absorption spectra in terms of oscillator strength (*f*) and wavelength (λ) (Figures S3–S6), along with detailed vertical excitation data, including the wavelengths of the first ten excited states and the main electronic transitions (Table S2–S5).

In all cases, the characteristic porphyrin absorption bands are clearly observed, featuring an intense Soret band between 380 and 500 nm (corresponding to 3.3 to 2.5 eV interval) and weaker Q bands spanning 500–700 nm (2.5 to 1.8 eV).^57^ Their electronic properties, along their well-known spectral profile, are commonly interpreted using the Gouterman’s “four-orbital” model, in which porphyrin absorption is dominated by transitions involving the four frontier orbitals: the occupied HOMO–1 and HOMO, and the unoccupied LUMO and LUMO+1.^58,59^ In general, this model provides a good description of symmetric porphyrins. However, for low-symmetry systems such as those explored here, deviations from the standard profile are observed, including contributions from additional orbitals and a lifting of the degeneracy among the frontier orbitals.

Figure 2 presents the simulated UV–vis spectra for various β,β-pyrrolic substituents, comparing their impact on the overall photophysical profile relative to the unsubstituted system (B_2_OF_2_–Por, shown in black). Figure 2a illustrates the effect of structural *variation 1*, while Figures 2b and 2c depict the influence of structural *variation 2* with electron-donating and electron-withdrawing groups, respectively. Each system is color-coded, with bold lines representing akamptisomer ***t***_**1**_, and lighter lines refer to ***t***_**2**_.

**Figure 2:**
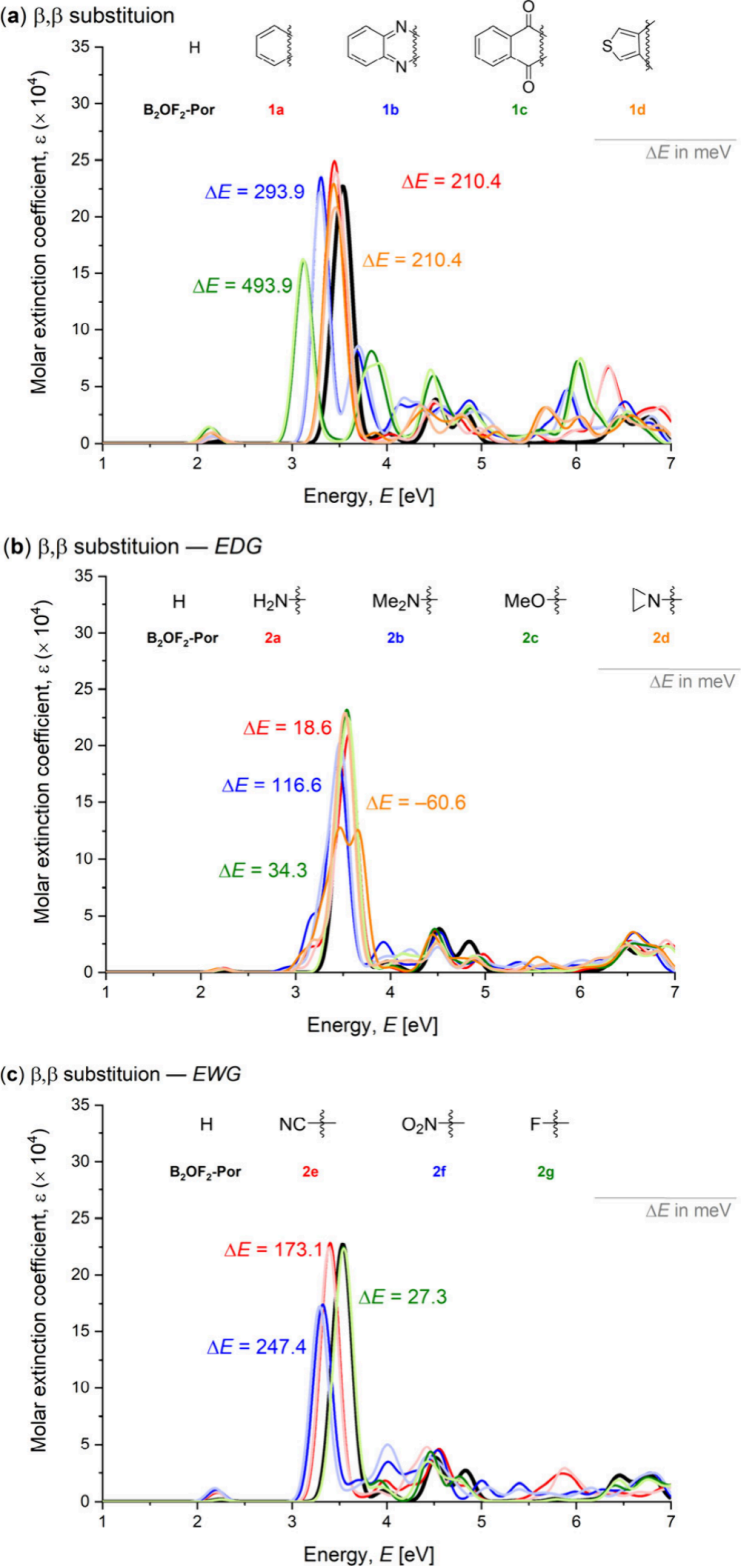
Simulated UV–vis absorption spectra calculated at TD-CAM-B3LYP/6–31+G** level for β,β substitutions: (a) structural *variation 1*, (b) structural *variation 2* with electron-donating groups, and (c) structural *variation 2* with electron-withdrawing groups. The reference system, unsubstituted porphyrin (B_2_OF_2_–Por), is represented by the black curve. Bold lines correspond to isomer ***t***_**1**_, while lighter lines correspond to ***t***_**2**_. Δ*E* values are reported in meV and denote the shift relative to the unsubstituted system, calculated based on the absorption with the highest molar absorptivity: Δ*E*B_2_OF_2_(Por) = *E*_ref_(highest ε) – *E* ***t***_**1**_(highest ε), corresponding to the Soret band shift.

The insertion of substituents at the β,β induces an overall bathochromic shift (redshift) in the systems compared to B_2_OF_2_–Por. The shifts depicted in the figure correspond to the maximum absorption peaks (highest ε, Soret bands) and were calculated relative to akamptisomer ***t***_**1**_: Δ*E* = *E*(***t***_**1**_) – *E*(B_2_OF_2_–Por), reported in meV. This redshift is attributed to a reduction in the frontier molecular orbital energy gap promoted by the substituents. Figure S8 (SI) displays the energies of the four Gouterman orbitals (HOMO–1, HOMO, LUMO, and LUMO+1) for the akamptisomer pairs and the reference system, clearly indicating a consistent decrease in the HOMO–LUMO gap across all substituted derivatives Notably, this redshift is most pronounced for system **1c** (structural *variation 1*), with Δ*E* = 493.9 meV, in which naphtho­(dione) was introduced as a substituent. The significant perturbation induced by the naphtho­(dione) moiety in the porphyrin framework is likely due to the formation of an extended conjugated system,^60,61^ which may result in enhanced interaction with visible light. Moreover, molecular orbital analysis (primarily HOMO–1/HOMO → LUMO transitions; see Figure S10, SI) suggests a possible intramolecular charge transfer (ICT) from the porphyrin macrocycle to the naphtho­(dione) unit. This effect, together with the well-established chromophoric nature of naphtho­(dione),^62^ further supports its role in modulating the photophysical response of the system. Previous studies have reported that anchoring quinone units at the β,β-pyrrolic positions of Zn-porphyrins can significantly alter the electronic structure of the macrocycle, leading to systems that absorb across the entire visible spectrum — often referred to as ‘black porphyrins’.^60^

In contrast, *meso* substitution resulted in a less pronounced redshift (Figure 3) compared to the β,β- substituted derivatives. The subtle effect of *meso* substituents on the UV–vis absorption profiles indicates a limited influence on the electronic structure, as evidenced by the relatively small decrease in the HOMO–LUMO gap concerning the reference compound (Figure S9). Nevertheless, structural *variation 3* — comprising both electron-donating (**3a–d**, Figure 3a) and electron-withdrawing groups (**3e–g**, Figure 3b) — induces a more notable redshift than the aliphatic substituents explored in *variation 4* (**4a–e**, Figure 3c). This difference can be attributed to the limited electronic effect of the aliphatic groups, whereas in *variation 3*, the substituents are π-conjugated with the porphyrin framework, thereby exerting a greater perturbation on the electronic structure.

**Figure 3:**
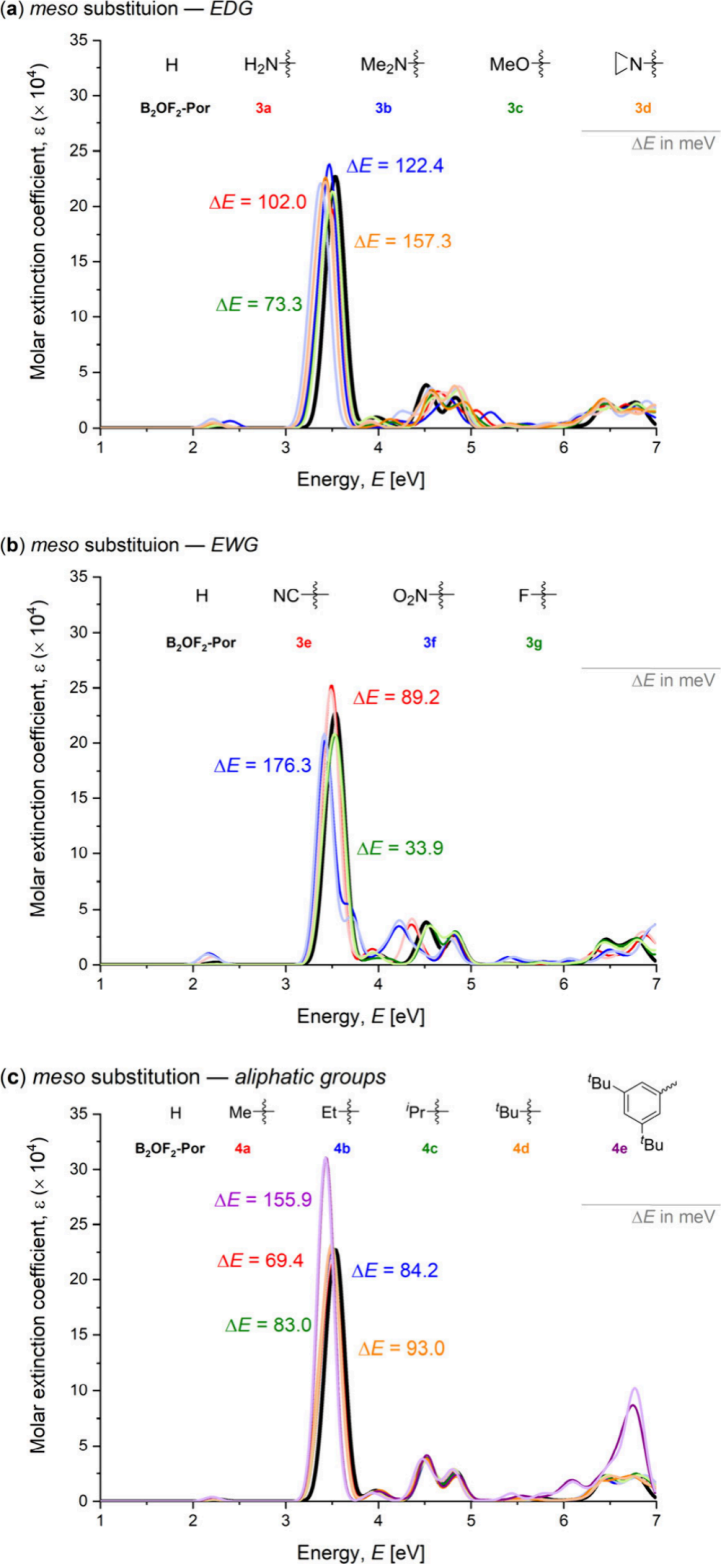
Simulated UV–vis absorption spectra calculated at TD-CAM-B3LYP/6–31+G** level for *meso* substitutions: (a) structural *variation 3* with electron-donating groups, (b) structural *variation 3* with electron-withdrawing groups, and (**c**) structural *variation 4*. The reference system, unsubstituted porphyrin (B_2_OF_2_–Por), is represented by the black curve. Bold lines correspond to isomer ***t***_**1**_, while lighter lines correspond to ***t***_**2**_. Δ*E* values are reported in meV and denote the shift relative to the unsubstituted system, calculated based on the absorption with the highest molar absorptivity: Δ*E*B_2_OF_2_(Por) = *E*_ref_(highest ε) – *E* ***t***_**1**_(highest ε), corresponding to the Soret band shift.

Although the overall substitutions — particularly those at the -β,β positions — significantly influence the porphyrin absorption spectra, no substantial differences were observed between the ***t***_**1**_ and ***t***_**2**_ absorption profiles; in most cases, their spectra are nearly superimposed (Figures 2 and 3, bold and lighter lines). This outcome suggests that, although structural distortions occur in the pseudoplanes during the BAR process, they are not pronounced enough to induce notable changes in the overall electronic structure between the ***t***_**1**_ and ***t***_**2**_ isomers. The distinct interaction of isomeric pairs with light is a key aspect in the design of optoelectronic switching compounds.^10,14^ To further investigate this aspect, we analyzed the differences in excitation wavelengths for the first five singlet excited states (S), which encompass the Q and Soret bands and, in most cases, the entire UV–vis spectrum. Table 2 presents the absolute energy differences and averages between the first five excited states, calculated as |Δ*E*|(S_*n*_) = |*E*S_*n*_ (***t***_**1**_) – *E*S_*n*_ (***t***_**2**_)| (*n* = 1 to 5), in meV.

**Table 2:** Difference (In Absolute Value) And Average between the Energy of the First Five Excited States |Δ*E*| (meV) = |*E* ***t***_**1**_ – *E* ***t***_**2**_ ^a^

		|Δ*E*|, meV						
		(S_1_)	(S_2_)	(S_3_)	(S_4_)	(S_5_)	(S)	Δ*E*B_2_OF_2_(Por)
β,β	**1a** (benzo)	10.1	13.8	6.4	21.8	0.8	10.6	210.4
	**1b** (quinoxalino)	7.0	11.1	7.1	4.6	33.7	12.7	293.9
	**1c** (naphtho(dione))	24.9	16.4	28.4	5.4	17.5	18.5	493.9
	**1d** (thieno)	17.6	21.0	9.9	40.9	15.4	21.0	210.4
	**2a** 2(−NH_2_)	6.8	13.7	178.2	143.0	63.1	81.0	18.6
	**2b** 2(−NMe_2_)	3.1	19.1	111.2	104.9	23.6	52.4	116.6
	**2c** 2(−OMe)	2.7	1.7	8.4	61.9	0.0	14.9	34.3
	**2d** 2(−aziridinyl)	32.6	12.9	96.1	135.5	55.1	66.4	– 60.6
	**2e** 2(−CN)	12.7	14.7	13.9	10.8	21.2	14.7	173.1
	**2f** 2(−NO_2_)	26.5	12.5	29.5	41.0	28.3	27.6	247.4
	**2g** 2(−F)	0.8	3.5	0.8	4.3	8.6	3.6	27.3
*meso*	**3a** (−NH_2_)	6.7	28.7	1.9	4.6	5.8	9.5	102.0
	**3b** (−NMe_2_)	40.2	195.4	78.9	85.9	34.3	86.9	122.4
	**3c** (−OMe)	1.8	7.7	10.7	1.9	3.9	5.2	73.3
	**3d** (−aziridinyl)	2.2	19.4	3.3	0.8	34.9	12.1	157.3
	**3e** (−CN)	3.5	17.0	10.2	8.7	4.3	8.7	89.2
	**3f** (−NO_2_)	6.5	26.1	9.4	12.1	0.1	10.8	176.3
	**3g** (−F)	5.2	12.2	3.3	9.3	27.9	11.6	33.9
	**4a** (−Me)	4.8	12.3	7.5	9.7	6.3	8.1	69.4
	**4b** (−Et)	1.6	6.3	1.1	1.5	1.6	2.4	84.2
	**4c** (−^*i*^Pr)	2.0	5.4	1.6	1.4	0.3	2.1	83.0
	**4d** (−^*t*^Bu)	12.8	4.0	11.0	2.2	3.1	6.6	93.0
	**4e** (3,5-di-*tert*-butylphenyl)	8.3	4.9	8.2	7.4	7.6	7.3	155.9

a Displacement relative to the unsubstituted system calculated in relation to the absorptions of the highest epsilon: Δ*E*B_2_OF_2_(Por) = *E*_ref_(highest ε) – *E* ***t***_**1**_(highest ε), in meV. Vertical excitation calculations obtained at TD-CAM-B3LYP/6-31+G**.

The most significant overall differentiation was observed for compound **3b** (*meso*-NMe_2_), with an average absolute difference ( $\overset{®}{\left|\Delta E\right|}$ ­(S)) of 86.9 meV, and the largest deviation occurring in the second excited state (|Δ*E*| (S_2_) = 195.4 meV. Within each structural variation, other compounds also stand out, such as **1c** (β,β-naphtho­(dione)), **2a** (β,β 2­(−NH_2_)), and **4d** (*meso*-^*t*^Bu). Once again, β,β- demonstrates a promising behavior, reinforcing the idea that incorporating chromophoric units into the (F)­B–O–B­(F)-porphyrin framework is a viable strategy to achieve both bathochromic shift and ***t***_**1**_/***t***_**2**_ absorption differentiation. Regarding electron-withdrawing groups, all substituents showed limited impact on the ***t***_**1**_/***t***_**2**_ differentiation for both -β,β (**2e–g**) and *meso* substitutions (**3e–g**). Among these, −NO_2_ derivatives (**2f** and **3f**) showed the most pronounced, albeit moderate, effect, with an average $\overset{®}{\left|\Delta E\right|}$ ­(S) = 27.6 meV for β,β substitution and 10.8 for *meso* substitution. In the case of **3b**, the observed differences in the absorption profiles are attributed to steric hindrance caused by peripheral repulsion between the *meso* substituent and the adjacent pyrrolic hydrogens (see Figure S2b, SI file), particularly in the ***t***_**2**_ isomer. This interaction likely reduces π–π overlap in one of the akamptisomer, leading to a slight twist of the substituent and consequently modifying its individual absorption behavior.

Despite the relatively small differences between the absorption profiles of the akamptisomers, the evaluated systems overall exhibit nearly identical optical responses. As a final strategy, new compounds were designed based on the key insights gathered in this section, particularly concerning the effects of *meso*-induced steric distortion and the resulting electronic perturbations. To this end, push–pull system within the (F)­B–O–B­(F)-porphyrin framework were explored.

### Building a *Chimera*: Design of Push–Pull Devices

The previous assessment of electron-donating (EDG) and electron-withdrawing groups (EWG) attached to the porphyrin moiety allowed us to rationally design push–pull systems, aiming to induce more pronounced alterations in the electronic structure of the porphyrin and thus achieve akamptisomeric ***t***_**1**_/***t***_**2**_ pairs with significantly distinct UV–vis absorption profiles. Such systems are widely reported for their ability to perturb the electronic structure of porphyrins,^34,63–65^ forming a D−π–A arrangement, where D is an electron-donating unit and A an electron-acceptor (or withdrawing) group, with the porphyrin acting both as a π-connector and a donating moiety.^34^ Based on these insights, we evaluated push–pull systems featuring different substitution patterns at the β,β, and *meso* position (Figure 4), employing −NH_2_ and −NMe_2_ as electron-donating groups, and −NO_2_ and −CN as electron-withdrawing groups.

**Figure 4:**
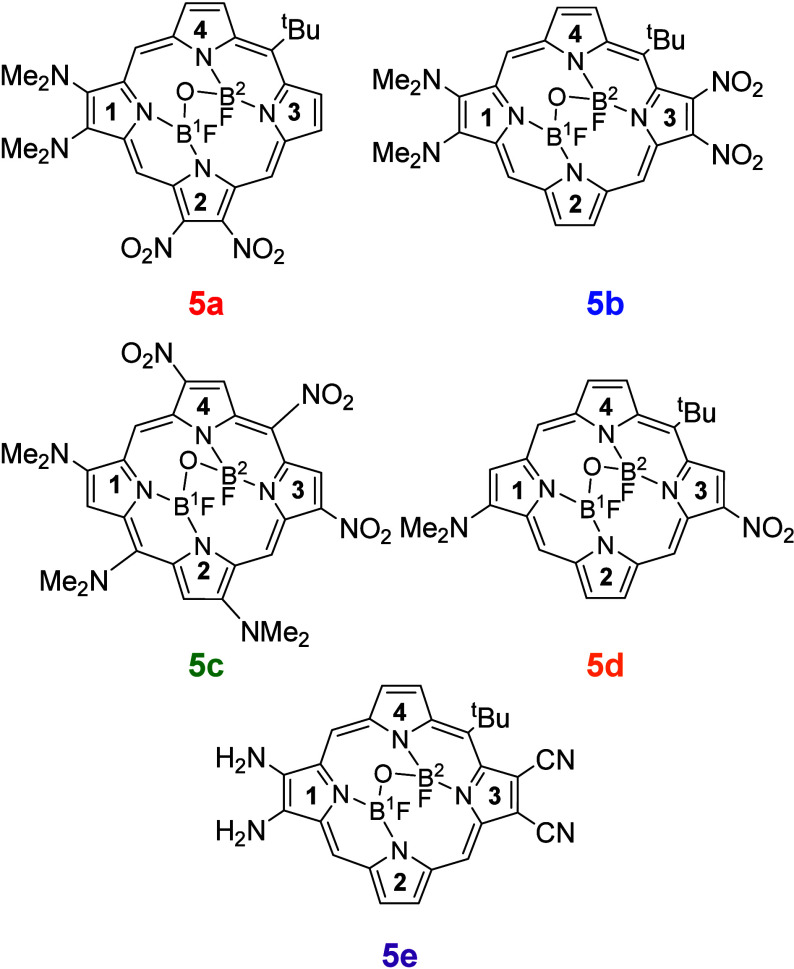
Proposed push–pull systems in the (F)­B–O–B­(F)-porphyrin framework.

Such substituents were selected based on their performance in distinguishing the ***t***_**1**_/***t***_**2**_ absorption profiles (Table 2). A -^*t*^Bu was introduced at one *meso* position (except in **5c**), as it appears to distort the pseudoplane and potentially enhance the ***t***_**1**_/***t***_**2**_ differentiation. To investigate this effect, the -^*t*^Bu group was positioned on one of the pseudoplanes formed by the (1 + 2) and (3 + 4) rings. Push–pull compounds **5a** and **5b** share identical substituents but differ in the spatial arrangement of the β,β-substituted rings, which may influence the intramolecular charge transfer. In **5c** and **5d**, the substitution pattern of **5b** was maintained, but with only a single substitution on the pyrrolic ring (β instead of β,β). Compound **5c** features both β and *meso* substitutions, while **5d** carries only a β substitution, with a -^*t*^Bu group still occupying one *meso* position (as in **5a** and **5b**). These different patterns were evaluated to assess potential peripheral repulsion between β,β substituents, which could be inferred from the nonplanarization of the Me_2_N-porphyrin core in the optimized structures. Additionally, compound **5e** was investigated, featuring a reduced steric hindrance by replacing the electron-donating (−NH_2_ to −NMe_2_) and electron-withdrawing (−NO_2_ to −CN) groups.

The term *chimera*, as used here, refers to the heterogeneous combination of substitution patterns designed to explore potential building blocks with distinct absorption profiles. By strategically varying these features, we aim to fine-tune the optical properties of individual akamptisomers.

Our simulations indicate that all *transoid* configurations of these push–pull porphyrinoid systems undergo akamptisomerism, with BAR energy barriers ranging from 20 to 26 kcal mol^–1^ (Table 3). Similar to systems **1**–**4**, the various substitution patterns in **5a–e** had minimal effects on the energy barrier, with Δ*G* ^‡^ = 23.5 ± 1.4 kcal mol^–1^, as well as on the relative energy between the isomers (−1.5 ± 1.7 kcal mol^–1^). Moreover, the *cisoid* ***c***_**1**_ configuration remained more energetic than its *transoid* counterpart by Δ*G* = 5.1 ± 1.1 kcal mol^–1^. At the same time, its isomer ***c***_**2**_ was not identified as a minimum energy point on the potential energy surface.

**Table 3:** Gibbs Free Energy Change (Relative to Isomer ***t***_1_) for the *transoid* ***t***_**1**_→ ***t***_**2**_ Bond Angle Reflection and Relative Energy of ***c***_**1**_ ^a^

	relative Gibbs free energy, kcal mol^–1^		
	TS (***t***_**1**_ *→* ***t***_**2**_)	***t***_**2**_	***c*_*1*_**
**5a**	23.5	–0.9	5.3
**5b**	21.5	–3.7	3.4
**5c**	25.4	0.7	5.5
**5d**	24.1	–1.5	6.5
**5e**	23.2	–2.7	4.8

a Reported values in kcal mol^–1^, computed at the B3LYP-D3/def2-QZVP//B3LYLP-D3/6-31+G** level.

Due to the akamptisomeric relationship and the possibility of isolating individual ***t***_**1**_/***t***_**2**_ isomers, we evaluated their absorption profiles (Figure 5). The vertical excitations and corresponding oscillator strengths are organized in Figure S7 and Table S6 in the SI file. In all cases, a redshift was observed compared to the reference system (B_2_OF_2_–Por), indicating a significant ability of these substitutions to perturb the electronic structure of the porphyrin framework. This finding is consistent with literature reports that describe how electron-donating and electron-withdrawing groups enhance absorption in the visible region.^34^

**Figure 5:**
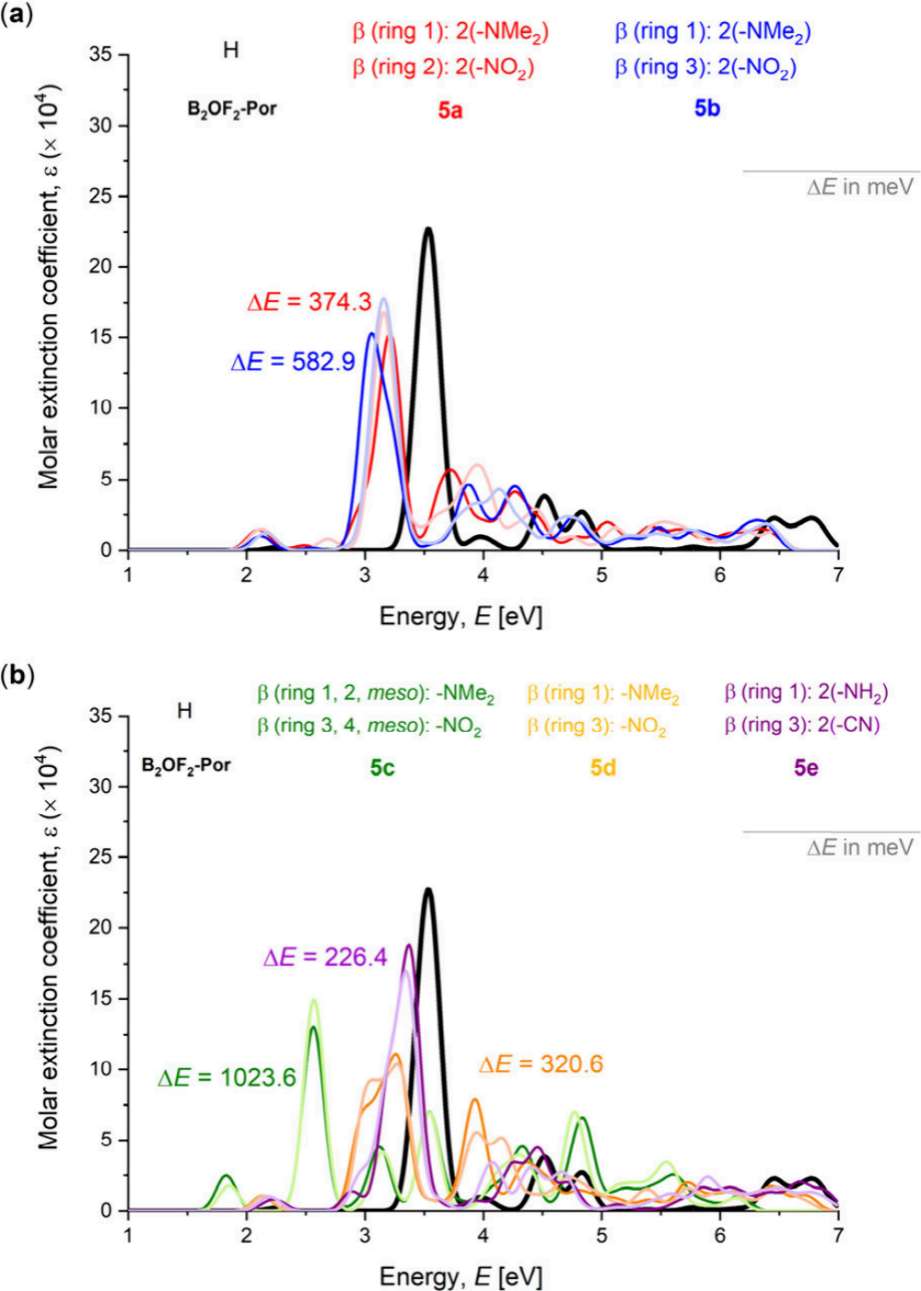
Simulated UV–vis absorption spectra calculated at the TD-CAM-B3LYP/6–31+G** level for push–pull systems: (a) **5a** and **5b**; (b) **5c**, **5d**, and **5e**. The reference system, unsubstituted porphyrin (B_2_OF_2_–Por), is represented by the black curve. Bold lines correspond to isomer ***t***_**1**_, while lighter lines correspond to ***t***_**2**_. Δ*E* values are reported in meV and denote the shift relative to the unsubstituted system, calculated based on the absorption with the highest molar absorptivity: Δ*E*B_2_OF_2_(Por) = *E*_ref_(highest ε) – *E* ***t***_**1**_(highest ε), corresponding to the Soret band shift.

The most significant impact was observed for **5c**, which exhibited a redshift (Δ*E*) of 1023.6 meV. This system features substitutions at both the β (one EDG at ring 1, another EDG at ring 2, one EWG at ring 3, and another EWG at ring 4) and *meso* positions, potentially facilitating a donor–acceptor effect across the entire macrocycle. The redshift can be directly attributed to a decrease in the HOMO–LUMO energy gap compared to Por−B_2_OF_2_, as shown in Figure S10 (SI), with the most substantial reduction observed for **5c** (energy gap of 3.5 eV). This overall gap decrease may be attributed to the push–pull effect, suggested by the intramolecular charge transfer character (EDG → EWG) observed in most cases, particularly in the HOMO → LUMO electronic transition. Figure 6 illustrates the frontier molecular orbitals of the ***t***_**1**_ push–pull systems. A similar profile was identified for the ***t***_**2**_ isomer, with the corresponding molecular orbitals organized in Figure S12 of the SI file.

**Figure 6:**
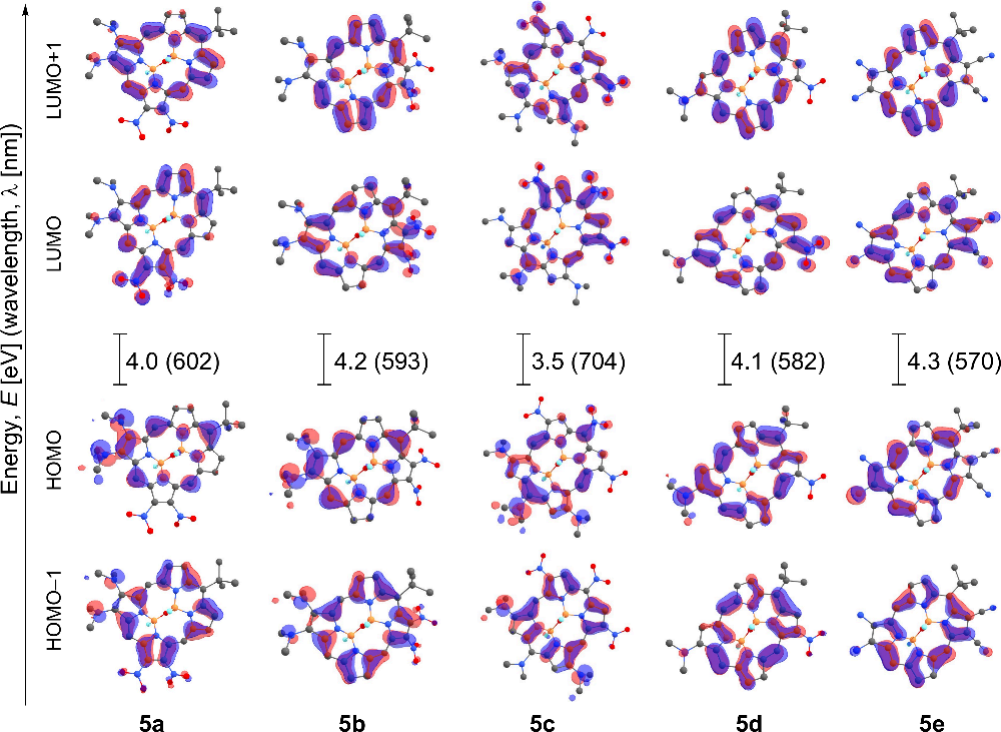
Frontier molecular orbitals (contour value set to 0.03) for akamptisomer ***t***_**1**_ of **5a**–**5e**. Hydrogen atoms were hidden for clarity. The intramolecular charge transfer character D → π → A is evident, where D represents the electron-donating unit (−NMe_2_, −NH_2_, and porphyrin), π denotes the connector (typically the porphyrin framework), and A signifies the acceptor unit (−NO_2_ and −CN). Kohn–Sham molecular orbitals were obtained using CAM-B3LYP/6–31+G** in the optimized structure (B3LYP-D3/6–31+G**).

From the differentiation between the ***t***_**1**_ and ***t***_**2**_ absorption profiles, Figure 5 illustrates a slight distinction between the bold (***t***_**1**_) and lighter (***t***_**2**_) absorption lines. The variance across the first five excitations (Table 4) shows that, while **5c** exhibits the most substantial overall redshift (Δ*E*), the difference between the ***t***_**1**_ and ***t***_**2**_ absorption spectra remains minimal. This suggests that the phenomenon of akamptisomerism as a photoswitching element is not directly correlated with the ability of the substituents to promote a bathochromic shift relative to the unsubstituted B_2_OF_2_–Por system. Significant differentiation between the ***t***_**1**_ and ***t***_**2**_ absorption spectra was observed only for **5a** and **5b**, with **5b** demonstrating the most pronounced effect among all evaluated systems. In **5b**, the differentiation is evident not only from the average $\overset{®}{\left|\Delta E\right|}$ ­(S) = 115.4 meV, but also from the largest single transition shift, observed for S_3_, with a difference of 320.8 meV.

**Table 4:** Difference (In Absolute Value) and Average between the Wavelengths of the First Five Excited States |Δ*E*| (meV) = |*E* ***t***_**1**_ – *E* ***t***_**2**_|^a^

	|Δ*E*| (S_1_)	|Δ*E*| (S_2_)	|Δ*E*| (S_3_)	|Δ*E*| (S_4_)	|Δ*E*| (S_5_)	$\overset{®}{\left|\Delta E\right|}\left(S\right)$	Δ*E*B_2_OF_2_(Por)
**5a**	13.7	13.1	201.0	146.3	49.9	84.8	374.3
**5b**	41.2	44.6	**320.8**	76.7	93.6	115.4	582.9
**5c**	10.5	25.2	11.6	0.8	53.4	20.3	1023.6
**5d**	11.5	11.0	34.7	19.5	19.6	19.3	320.6
**5e**	12.5	47.4	79.4	20.2	24.0	36.7	226.4

a Displacement relative to the unsubstituted system calculated in relation to the absorptions of the highest epsilon: Δ*E*B_2_OF_2_(Por) = *E*_ref_(highest ε) – *E* ***t***_**1**_(highest ε). Vertical excitation calculations obtained in TD-CAM-B3LYP/6-31+G**.

To understand the origin of the S_3_ differentiation in **5b**, we examined the primary electronic transition for this absorption, which corresponds to the HOMO–2→LUMO transition (63% and 64% for ***t***_**1**_ and ***t***_**2**_, respectively). This transition exhibits an intramolecular charge transfer character from the donating site (−NMe_2_) to the accepting site (−NO_2_), as depicted in Figure 7a. In the ***t***_**1**_ isomer, the EWG (−NO_2_) groups are positioned within the boron pseudoplane, while the EDG (−NMe_2_) groups are located outside this plane. Conversely, in ***t***_**2**_, the EDG lies within the plane, while the EWG is positioned outside the plane (Figure 7b). Thus, although intramolecular charge transfer occurs in both cases, the push–pull effect is mediated through different pseudoplanes, which is the primary cause of the differentiation between ***t***_**1**_ and ***t***_**2**_.

**Figure 7:**
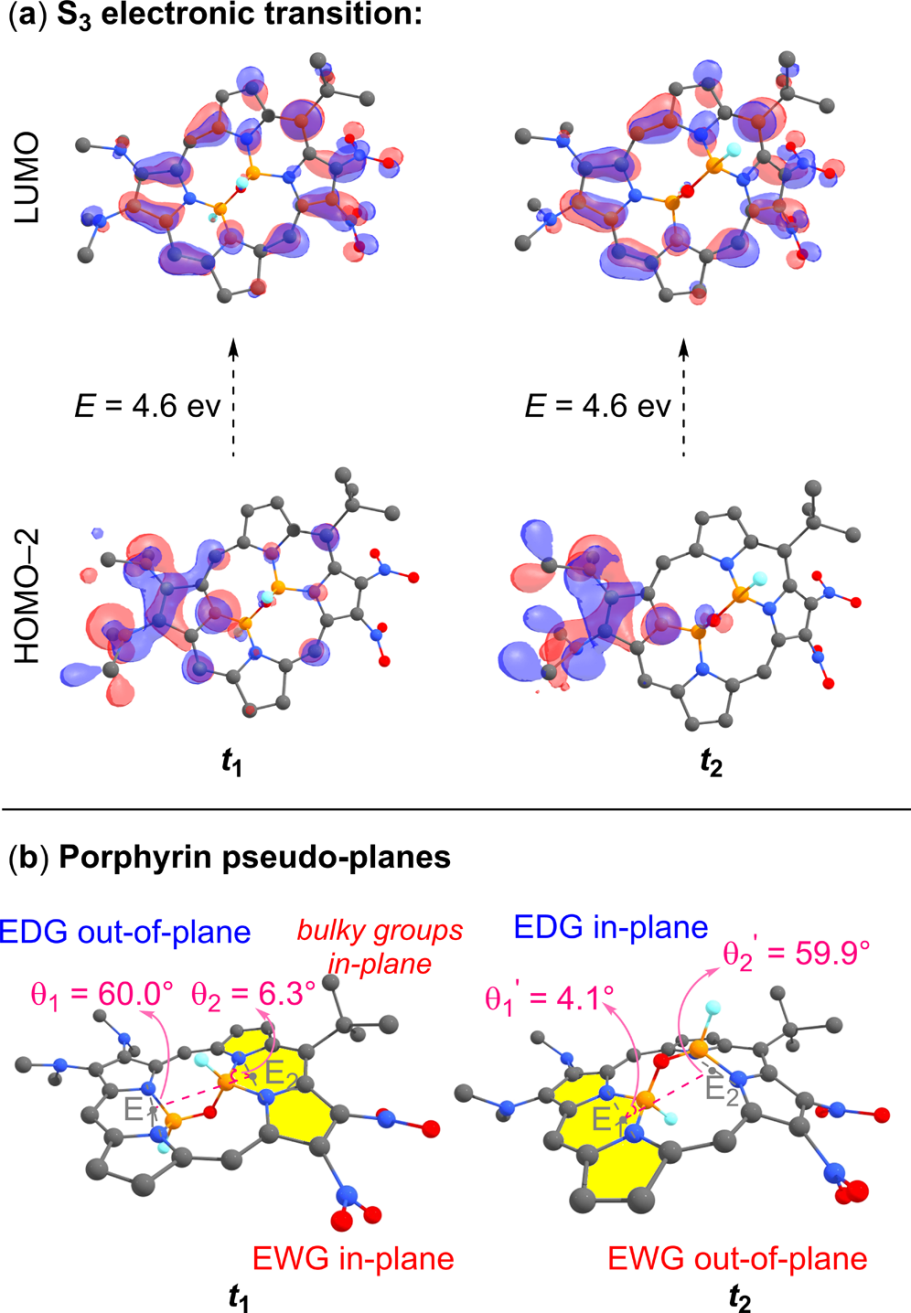
(a) Frontier molecular orbitals (contour value set to 0.03) for the major S_3_ electronic transition of **5b** akamptisomers ***t***_**1**_ and ***t***_**2**_. The intramolecular charge transfer character D → A is evident, where D represents the electron-donating unit (−NMe_2_) and A represents the acceptor unit (−NO_2_). Kohn–Sham molecular orbitals were obtained using CAM-B3LYP/6–31+G** in the optimized structure, at the B3LYP-D3/6–31+G** level. (b) Optimized structure of **5b** ***t***_**1**_ and ***t***_**2**_ showing the in-plane and out-of-plane portions and the location of electronic-donating (EDG) and electron-withdrawing (EWG) groups. θ_i_ corresponds to the angle between E_1_–E_2_–B (θ_1_) and E_2_–E_1_–B (θ_2_), where E_i_ (in gray) is defined by placing a dummy atom between the N···N atoms to define the pseudoplanes. Hydrogen atoms were hidden for clarity.

Additionally, the **5b** system exhibits a pronounced distortion characteristic. In ***t***_**1**_, the boron atom located in-plane is slightly elevated above the porphyrin pseudoplane compared to ***t***_**2**_, as indicated by θ_2_ = 6.3° in ***t***_**1**_ and θ_1_′ = 4.1° in ***t***_**2**_ (Figure 7b). This increased θ_2_ angle in ***t***_**1**_ can be attributed to the distortion caused by the bulky −^*t*^Bu group at the *meso* position, neighboring the −NO_2_ group, which is positioned in-plane in this structure (Figure 7b, pseudoplane highlighted in yellow).

In all other evaluated systems, the effect of steric congestion is reduced due to the nature of the substituents and/or the substitution pattern. In **5a**, the electron-donating and electron-withdrawing groups are located on opposite sides of the -^*t*^Bu group, which is flanked only by hydrogen atoms, thereby minimizing its distortion effect (***t***_**1**_: θ_2_ = 4.7°, θ_1_′ = 4.2). In **5c**, the *meso* position is not occupied by a -^*t*^Bu group; instead, it is substituted with both EDG and EWG groups, resulting in a minor distortion (***t***_**1**_: θ_2_ = 4.3°, θ_1_′ = 5.0) due to only one β-substitution. A similar pattern is observed for **5d**, where only one β-substitution is present alongside neighboring hydrogen atoms, despite the presence of a -^*t*^Bu group at the *meso* position (***t***_**1**_: θ_2_ = 5.0°, θ_1_′ = 4.7). In **5e**, although featuring β,β-substitution, the use of a linear electron-withdrawing group (−CN) helps reduce steric congestion (***t***_**1**_: θ_2_ = 5.1°, θ_1_′ = 4.2).

Thus, we ascribe the differentiation observed in **5b** to the combination of the push–pull effect occurring across different pseudoplanes and the greater distortion in one of the akamptisomers caused by the insertion of bulky substituents at the *meso* position.

Despite the overall small magnitude of the average differentiation ( $\overset{®}{\left|\Delta E\right|}$ ­(S) = 115.4 meV), the significant S_3_ differentiation (|Δ*E*| = 320.8 meV) provides compelling evidence that distortion of the pseudoplanes can lead to isomeric differentiation. Moreover, the interplay between bond angle reflection and akamptisomerism can directly influence the electronic structure of the porphyrin, representing the first indication that such a structural change could serve as a novel photoswitching mechanism. These findings are pivotal for advancing our understanding of electronic interactions in systems exhibiting akamptisomerismo.

It is noteworthy that the substitutions employed here maintain high structural simplicity, enabling the development of a simplified model that can be further explored through various substitution patterns on the porphyrin framework, a well-known organic building block. This potential paves the way for designing new molecular photoswitches with a wide range of possible applications. Evaluations regarding the behavior of the BAR in the excited state are currently underway in our research group.

## Conclusion

The exploration of novel structural alterations capable of generating photoswitchable compounds is essential for advancing new technological materials. Among the fundamental requirements for designing an optoelectronic switching, two key factors stand out: (*i*) the occurrence of an isomerization process that generates isolable compounds, and (*ii*) differentiation in the photophysical profiles of the resulting isomers. Together, these features enable the selective addressability of the isomers through band separation, broadening their potential applications.

Herein, we investigated, for the first time, bond angle reflection (BAR) — also known as akamptisomerism — as a novel photoswitching mechanism in low-symmetry porphyrins. Using DFT methods (B3LYP-D3/def2-QZVP//B3LYP-D3/6–31+G**), we demonstrated that all analyzed systems undergo akamptisomerism via *transoid* (F)­B–O–B­(F) bond angle reflection. This process exhibits a calculated energy barrier of Δ*G* ^‡^ = 27.1 ± 1.6 kcal mol^–1^, leading to the formation of isolable, effectively isoenergetic isomers (Δ*G* = 0.2 ± 0.6 kcal mol^–1^) in a diastereomeric relationship. The consistency in the energetic profiles across different β,β- and *meso*-substituted systems suggests that these peripheral modifications exert minimal influence on the akamptisomerism process, which instead appears to be primarily controlled by the intrinsic properties of the (F)­B–O–B­(F) bridge and the size of the macrocyclic framework.

The designed models exhibited a redshift compared to the unsubstituted reference (Por-B_2_OF_2_), with the most significant electronic perturbations observed for β-substituted systems. However, among the 23 initial models, the akamptisomer pairs displayed largely similar UV–vis absorption profiles. Although the BAR process induces distortion of the porphyrin pseudoplane, it does not significantly alter the electronic structure of the individual isomers.

To further investigate this effect, we explored five push–pull systems incorporating key geometric and electronic features identified in the initial set. In this subset, akamptisomerism was confirmed (Δ*G* ^‡^ = 23.5 ± 1.4 kcal mol^–1^), and one promising system exhibited UV–vis differentiation of up to 320.8 meV in the S_0_ → S_3_ transition. This β,β-substituted system, bearing -NMe_2_ and -NO_2_ groups positioned on opposite pseudoplanes and a bulky -^*t*^Bu group at the *meso* position, exhibited differentiation due to intramolecular charge transfer occurring between distinct pseudoplanes. The configuration alternates between out-of-plane electron-donating groups (-NMe_2_) and in-plane electron-withdrawing groups (-NO_2_ and -^*t*^Bu) in the akamptisomers. Furthermore, steric repulsion between the in-plane -^*t*^Bu and β-NO_2_ groups enhances the porphyrin distortion, contributing to the observed spectral differences.

Thus, our theoretical model establishes akamptisomerism as a promising switching element for the rational design of novel switchable compounds. This strategy can be realized by strategically positioning electron-donating, electron-withdrawing, and bulky groups according to the trends identified herein. Future work will focus on investigating the behavior of these systems in the excited state to further validate light-induced isomerization and confirm the applicability of akamptisomerism as a functional (photo)­switching mechanism.

## Supplementary Material



## Notes

Details of stereodescriptor determinations, additional electronic and geometric information, and all Cartesian coordinates (PDF)

## References

[1] Dattler D., Fuks G., Heiser J., Moulin E., Perrot A., Yao X., Giuseppone N. (2020). Design of
Collective Motions from Synthetic Molecular
Switches, Rotors, and Motors. Chem. Rev..

[2] Fitzmaurice O., Bartkowski M., Giordani S. (2022). Molecular Switches—Tools for
Imparting Control in Drug Delivery Systems. Front. Chem..

[3] Volarić J., Szymanski W., Simeth N. A., Feringa B. L. (2021). Molecular Photoswitches
in Aqueous Environments. Chem. Soc. Rev..

[4] Hoorens M.
W. H., Medved’ M., Laurent A. D., Di Donato M., Fanetti S., Slappendel L., Hilbers M., Feringa B. L., Jan Buma W., Szymanski W. (2019). Iminothioindoxyl
as a Molecular Photoswitch
with 100 Nm Band Separation in the Visible Range. Nat. Commun..

[5] Feringa, B. L. ; Browne, W. R. Molecular Switches; Wiley-VCH: Weinheim, Germany, 2011.

[6] Browne W. R., Feringa B. L. (2009). Light Switching of Molecules on Surfaces. Annu. Rev. Phys. Chem..

[7] Mrozek, T. ; Ajayaghosh, A. ; Daub, J. Optoelectronic Molecular Switches Based on Dihydroazulene-Vinylheptafulvene (DHA-VHF). In Molecular Switches; Feringa, B. L. , Browne, W. R. , Eds.; Wiley, 2001; pp 63–106. 10.1002/3527600329.ch3.

[8] Raymo F. M., Giordani S. (2002). All-Optical Processing with Molecular Switches. Proc. Natl. Acad. Sci. U. S. A..

[9] Zhou X. X., Lin M. Z. (2013). Photoswitchable
Fluorescent Proteins: Ten Years of
Colorful Chemistry and Exciting Applications. Curr. Opin. Chem. Biol..

[10] Jago D., Gaschk E. E., Koutsantonis G. A. (2023). History
and Fundamentals of Molecular
Photochromism. Aust. J. Chem..

[11] Goulet-Hanssens A., Eisenreich F., Hecht S. (2020). Enlightening Materials with Photoswitches. Adv. Mater..

[12] Hermanns V., Scheurer M., Dreuw A., Wachtveitl J., Braun M., Heckel A. (2022). Electronic Circular
Dichroism Unravels
Atropisomers of a Broadly Absorbing Fulgide Derivative. ChemPhotoChem..

[13] Feringa B. L. (2007). The Art
of Building Small: From Molecular Switches to Molecular Motors. J. Org. Chem..

[14] Zhang Z., Wang W., O’Hagan M., Dai J., Zhang J., Tian H. (2022). Stepping Out of the Blue: From Visible
to Near-IR Triggered Photoswitches. Angew. Chem.,
Int. Ed..

[15] Canfield P. J., Blake I. M., Cai Z.-L., Luck I. J., Krausz E., Kobayashi R., Reimers J. R., Crossley M. J. (2018). A New Fundamental
Type of Conformational Isomerism. Nat. Chem..

[16] Canfield P. J., Reimers J. R., Crossley M. J. (2024). Polytopal Rearrangement Model of
Stereoisomerization” and Its Potential as the Basis for a Systematic
Model of All Stereoisomerism. ACS Org. Inorg.
Au.

[17] Brothers P. J. (2002). Recent
Developments in the Coordination Chemistry of Porphyrin Complexes
Containing Non-Metallic and Semi-Metallic Elements. J. Porphyr. Phthalocyanines.

[18] Tay A. C. Y., Frogley B. J., Ware D. C., Conradie J., Ghosh A., Brothers P. J. (2019). Tetrahedral Pegs
in Square Holes: Stereochemistry of
Diboron Porphyrazines and Phthalocyanines. Angew.
Chem., Int. Ed..

[19] Belcher W. J., Boyd P. D. W., Brothers P. J., Liddell M. J., Rickard C. E. F. (1994). New
Coordination Mode for the Porphyrin Ligand in the Boron Porphyrin
Complex B2OF2­(TTP). J. Am. Chem. Soc..

[20] Novikova N., Brothers P. J., Simpson M. C. (2014). Spectroscopic Analysis of Boron Porphyrin
Complexes. Int. J. Nanotechnol..

[21] Sinha A., Chatterjee T., Ravikanth M. (2022). Synthesis and Properties of Boron
Porphyrinoids. Coord. Chem. Rev..

[22] Wang S., Wang Z., Song W., Gao H., Wu F., Zhao Y., Chan K. S., Shen Z. (2022). B-O-B Bridged
BOPPY
Derivatives: Synthesis, Structures, and Acid-Catalyzed Cis - Trans
Isomeric Interconversion. Dalton Trans..

[23] Xu N., Ono T., Morita Y., Komatsu T., Hisaeda Y. (2021). Rectangular Holes in
Porphyrin Isomers Act As Mono- and Binucleating Ligands: Stereochemistry
of Mono- and Diboron Porphycenes and Their Protonation Behaviors. Inorg. Chem..

[24] Stasyuk A. J., Stasyuk O. A., Solà M., Voityuk A. A. (2019). Peculiar Photoinduced
Electron Transfer in Porphyrin-Fullerene Akamptisomers. Chem. - Eur. J..

[25] Auwärter W., Écija D., Klappenberger F., Barth J. V. (2015). Porphyrins at Interfaces. Nat. Chem..

[26] Hush N. S., Reimers J. R., Hall L. E., Johnston L. A., Crossley M. J. (1998). Optimization
and Chemical Control of Porphyrin-Based Molecular Wires and Switches. Ann. N.Y. Acad. Sci..

[27] Crossley M. J., Johnston L. A. (2002). Laterally-Extended
Porphyrin Systems Incorporating
a Switchable Unit. Chem. Commun..

[28] Sendt K., Johnston L. A., Hough W. A., Crossley M. J., Hush N. S., Reimers J. R. (2002). Switchable Electronic
Coupling in Model Oligoporphyrin
Molecular Wires Examined through the Measurement and Assignment of
Electronic Absorption Spectra. J. Am. Chem.
Soc..

[29] Canfield P. J., Crossley M. J. (2025). Rigorous treatment of polytopal rearrangements reveal
surprising complexity of stereoisomerism configuration landscapes. Chem. Sci..

[30] de
Andrade K. N., Martorano L. H., Correa G. S., dos Santos F. M., Carneiro J. W. de M., de Albuquerque A. C.
F., Gomes A. C. C., Fiorot R. G. (2023). Going beyond Structural Effects: Explicit Solvation
Influence on the Rotational Isomerism of *C*-Glycosylated
Flavonoids. Org. Chem. Front..

[31] de
Andrade K., Fajardo J. R., Leal C., Carneiro J. W., Fiorot R. (2023). Computation-Guided Support to Experiments by the Exploration
of Reaction Mechanisms: Organic Synthesis, Natural Products and Environmental
Issues. J. Braz. Chem. Soc..

[32] Sharma V., Wang C., Lorenzini R. G., Ma R., Zhu Q., Sinkovits D. W., Pilania G., Oganov A. R., Kumar S., Sotzing G. A., Boggs S. A., Ramprasad R. (2014). Rational Design
of All Organic Polymer Dielectrics. Nat. Commun..

[33] Guan B., Jiang H., Wei Y., Liu Z., Wu X., Lin H., Huang Z. (2021). Density Functional
Theory Researches for Atomic Structure,
Properties Prediction, and Rational Design of Selective Catalytic
Reduction Catalysts: Current Progresses and Future Perspectives. Mol. Catal..

[34] Bureš F. (2014). Fundamental
Aspects of Property Tuning in Push-Pull Molecules. RSC Adv..

[35] Frisch, M. J. ; Trucks, G. W. ; Schlegel, H. B. ; Scuseria, G. E. ; Robb, M. A. ; Cheeseman, J. R. ; Scalmani, G. ; Barone, V. ; Petersson, G. A. ; Nakatsuji, H. ; Li, X. ; Caricato, M. ; Marenich, A. ; Bloino, J. ; Janesko, B. G. ; Gomperts, R. ; Mennucci, B. ; Hratchian, H. P. ; Ortiz, J. V. ; Izmaylov, A. F. ; Sonnenberg, J. L. ; Williams-Young, D. ; Ding, F. ; Lipparini, F. ; Egidi, F. ; Goings, J. ; Peng, B. ; Petrone, A. ; Henderson, T. ; Ranasinghe, D. ; Zakrzewski, V. G. ; Gao, J. ; Rega, N. ; Zheng, G. ; Liang, W. ; Hada, M. ; Ehara, M. ; Toyota, K. ; Fukuda, R. ; Hasegawa, J. ; Ishida, M. ; Nakajima, T. ; Honda, Y. ; Kitao, O. ; Nakai, H. ; Vreven, T. ; Throssell, K. ; Montgomery, J. A. ; Ogliaro, J. E. P. F., Jr. ; Bearpark, M. ; Heyd, J. J. ; Brothers, E. ; Kudin, K. N. ; Staroverov, V. N. ; Keith, T. ; Kobayashi, R. ; Normand, J. ; Raghavachari, K. ; Rendell, A. ; Burant, J. C. ; Iyengar, S. S. ; Tomasi, J. ; Cossi, M. ; Millam, J. M. ; Klene, M. ; Adamo, C. ; Cammi, R. ; Ochterski, J. W. ; Martin, R. L. ; Morokuma, K. ; Farkas, O. ; Foresman, J. B. ; Fox, D. J. . Gaussian 09, rev. A.02; Gaussian, Inc.: Wallingford, CT, 2016.

[36] Becke A. D. (1993). Density-functional
Thermochemistry. III. The Role of Exact Exchange. J. Chem. Phys..

[37] Becke A. D. (1993). A New Mixing
of Hartree-Fock and Local Density-functional Theories. J. Chem. Phys..

[38] Lee C., Yang W., Parr R. G. (1988). Development
of the Colle-Salvetti
Correlation-Energy Formula into a Functional of the Electron Density. Phys. Rev. B.

[39] Petersson G. A., Al-Laham M. A. (1991). A Complete Basis Set Model Chemistry. II. Open-shell
Systems and the Total Energies of the First-row Atoms. J. Chem. Phys..

[40] Gordon M. S., Binkley J. S., Pople J. A., Pietro W. J., Hehre W. J. (1982). Self-Consistent
Molecular-Orbital Methods. 22. Small Split-Valence Basis Sets for
Second-Row Elements. J. Am. Chem. Soc..

[41] Grimme S., Antony J., Ehrlich S., Krieg H. (2010). A Consistent and Accurate
Ab Initio Parametrization of Density Functional Dispersion Correction
(DFT-D) for the 94 Elements H-Pu. J. Chem. Phys..

[42] Ochterski, J. W. . Thermochemistry in Gaussian; Gaussian, Inc.: Wallingford, CT, 2000.

[43] Weigend F., Ahlrichs R. (2005). Balanced Basis Sets
of Split Valence, Triple Zeta Valence
and Quadruple Zeta Valence Quality for H to Rn: Design and Assessment
of Accuracy. Phys. Chem. Chem. Phys..

[44] Bursch M., Mewes J., Hansen A., Grimme S. (2022). Best-Practice DFT Protocols
for Basic Molecular Computational Chemistry**. Angew. Chem., Int. Ed..

[45] Runge E., Gross E. K. U. (1984). Density-Functional
Theory for Time-Dependent Systems. Phys. Rev.
Lett..

[46] Adamo C., Jacquemin D. (2013). The Calculations of Excited-State
Properties with Time-Dependent
Density Functional Theory. Chem. Soc. Rev..

[47] Yanai T., Tew D. P., Handy N. C. (2004). A New Hybrid
Exchange-Correlation
Functional Using the Coulomb-Attenuating Method (CAM-B3LYP). Chem. Phys. Lett..

[48] Drzewiecka-Matuszek A., Rutkowska-Zbik D. (2021). Application
of TD-DFT Theory to Studying Porphyrinoid-Based
Photosensitizers for Photodynamic Therapy: A Review. Molecules.

[49] Mack J., Stone J., Nyokong T. (2014). Trends in the TD-DFT
Calculations
of Porphyrin and Phthalocyanine Analogs. J.
Porphyr. Phthalocyanines.

[50] Cai Z.-L., Crossley M. J., Reimers J. R., Kobayashi R., Amos R. D. (2006). Density Functional Theory for Charge
Transfer: The
Nature of the N-Bands of Porphyrins and Chlorophylls Revealed through
CAM-B3LYP, CASPT2, and SAC-CI Calculations. J. Phys. Chem. B.

[51] Rätsep M., Cai Z.-L., Reimers J. R., Freiberg A. (2011). Demonstration and Interpretation
of Significant Asymmetry in the Low-Resolution and High-Resolution *Q y* Fluorescence and Absorption Spectra of Bacteriochlorophyll *a*. J. Chem. Phys..

[52] Baerends E. J., Ricciardi G., Rosa A., van Gisbergen S. J. A. (2002). A DFT/TDDFT
Interpretation of the Ground and Excited States of Porphyrin and Porphyrazine
Complexes. Coord. Chem. Rev..

[53] Creating UV/Visible Plots from the Results of Excited States Calculations. Gaussian, 2017. https://gaussian.com/uvvisplot/.

[54] Brothers P. J. (2011). Boron Complexes
of Pyrrolyl Ligands. Inorg. Chem..

[55] The IUPAC Compendium of Chemical Terminology; Gold, V. , Ed.; International Union of Pure and Applied Chemistry (IUPAC): Research Triangle Park, NC, 2019. 10.1351/goldbook.

[56] Harris J.
D., Moran M. J., Aprahamian I. (2018). New Molecular Switch Architectures. Proc. Natl. Acad. Sci. U. S. A..

[57] Giovannetti, R. The Use of Spectrophotometry UV-Vis for the Study of Porphyrins. In Macro To Nano Spectroscopy; Uddin, J. , Ed.; IntechOpen, 2012. 10.5772/38797.

[58] Gouterman M., Wagnière G. H., Snyder L. C. (1963). Spectra of Porphyrins. J. Mol. Spectrosc..

[59] Wamser C. C., Ghosh A. (2022). The Hyperporphyrin
Concept: A Contemporary Perspective. JACS Au.

[60] Banala S., Rühl T., Wurst K., Kräutler B. (2009). “Blackening”
Porphyrins by Conjugation with Quinones. Angew.
Chem., Int. Ed..

[61] Banala S., Wurst K., Kräutler B. (2016). Panchromatic
π-Extended Porphyrins
from Conjugation with Quinones. ChemPlusChem..

[62] Kuboyama A. (1960). The *n* -Π* Transition Band of Acenaphthenequinone. Bull. Chem. Soc. Jpn..

[63] Parsa Z., Naghavi S. S., Safari N. (2018). Designing
Push-Pull Porphyrins for
Efficient Dye-Sensitized Solar Cells. J. Phys.
Chem. A.

[64] Rathi P., Ekta, Kumar S., Banerjee D., Soma V. R., Sankar M. (2020). Unsymmetrical β-Functionalized
‘Push-Pull’ Porphyrins: Synthesis and Photophysical,
Electrochemical and Nonlinear Optical Properties. Dalton Trans..

[65] Sekaran B., Jang Y., Misra R., D’Souza F. (2019). Push-Pull
Porphyrins via β-Pyrrole Functionalization: Evidence of Excited
State Events Leading to High-Potential Charge-Separated States. Chem. - Eur. J..

