# Supplementary material for: Akamptisomerism as a Switching Element: Substituent Effects on Bond Angle Reflection and Photophysical Properties of B–O–B Porphyrins
Source: Inorg Chem. 2025 Jun 3;64(23):11683–95. doi: 10.1021/acs.inorgchem.5c01132 (PMC12175122; doi:10.1021/acs.inorgchem.5c01132)
Supplement: Supplementary file 1 [file ic5c01132_si_001.pdf]

Akamptisomerism as a Switching Element: Substituent Effects on Bond Angle Reflection and Photophysical Properties of B–O–B Porphyrins

Karine N. de Andrade[1,†], Natalia M. Raffaeli[1,†], Rodolfo G. Fiorot[1,*]

[1]Department of Organic Chemistry, Institute of Chemistry, Universidade Federal Fluminense (UFF), Outeiro de São João Batista, 24020-141, Niterói, RJ, Brazil

[†] These authors equally contributed to the work.

*Corresponding author: Rodolfo G. Fiorot – rodolfofiorot@id.uff.br

Contents

1. *Amplo*/*parvo* stereodescriptors for akamptisomers pairs............2

2. Analysis of akamptisomer pairs: structural insights............4

3. Absorption profile: light interaction............5

3.1 UV-Vis spectra: oscillator strength............5

3.2 Vertical excitation: absorption spectra and electronic transitions............9

4. Energy diagram of frontier orbitals............17

5. Molecular orbitals — spatial visualization............21

6. Matrices of optimized structures............22

1. *Amplo*/*parvo* stereodescriptors for akamptisomers pairs

The stereodescriptors proposed by Canfield and co-workers, *amplo* and *parvo*, can be calculated using a reference line connecting the nitrogen pyrrolic atoms, as illustrated in the following figure. The centers of this line, named E_1 and E_2, allow us to visualize the angles θ_1 and θ_2. We definied the center by placing a dummy atom between the nitrogen atoms. During the bond angle reflection (BAR), these angles turn into θ_1' and θ_2', respectively. The stereodescriptor *parvo* is the BAR center where the ratio θ_i/θ_i' is a value lower than 2/3, while the *amplo* center should have θ_i/θ_i' a value higher than 3/2. The calculated θ_i/θ_i' and the stereodescriptors for all evaluated systems are organized in table S1.

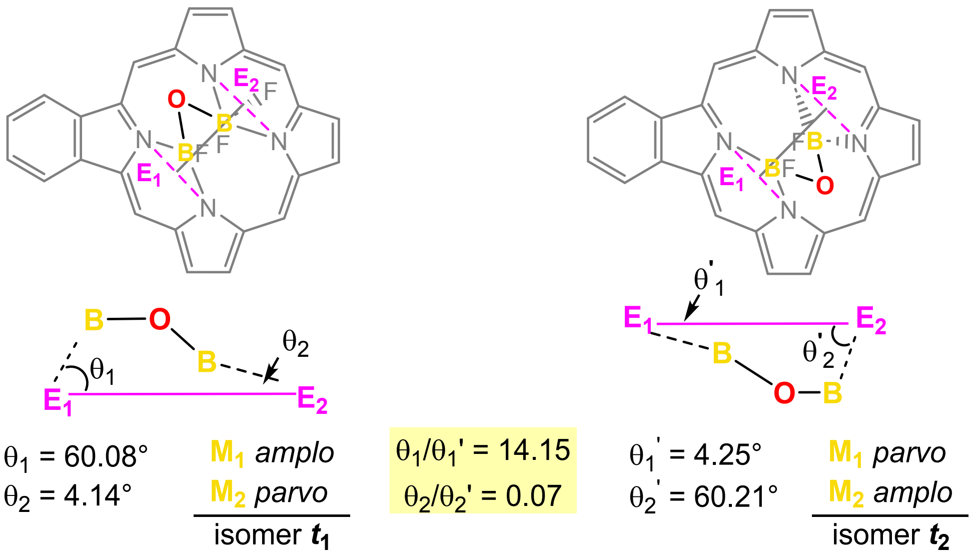

Figure S1: Determination of *amplo*/*parvo* from the ratio θ_i/θ_i' for an exemplified system (**1a**). Reported angles obtained from the optimized geometry at B3LYLP-D3/6-31+G** level.

Table S1. *Amplo*/*parvo* stereodescriptors determination calculated at B3LYLP-D3/6-31+G** for the akamptisomer pairs and *cisoid* (c_1) configuration.

		θ_1/θ_1'	θ_2/θ_2'	Descritor			θ_1/θ_1'	θ_2/θ_2'	Descritor
	t_1	14.15	0.07	*amplo, parvo*	**3a**	t_1	14.00	0.07	*amplo, parvo*
**1a**	t_2	0.07	14.15	*parvo, amplo*		t_2	0.07	15.00	*parvo, amplo*
	c_1	64.84	65.19	*amplo,amplo*		c_1	64.77	64.77	*amplo,amplo*
	t_1	14.00	0.07	*amplo, parvo*	**3b**	t_1	13.50	0.07	*amplo, parvo*
**1b**	t_2	0.07	14.70	*parvo, amplo*		t_2	0.07	14.30	*parvo, amplo*
	c_1	64.45	64.87	*amplo,amplo*		c_1	64.61	64.55	*amplo,amplo*

	t_1	15.00	0.07	*amplo, parvo*		t_1	13.90	0.07	*amplo, parvo*
**1c**	t_2	0.07	14.95	*parvo, amplo*	**3c**	t_2	0.07	14.60	*parvo, amplo*
	c_1	65.33	65.56	*amplo,amplo*		c_1	64.77	64.74	*amplo,amplo*
	t_1	13.71	0.07	*amplo, parvo*		t_1	13.70	0.07	*amplo, parvo*
**1d**	t_2	0.07	14.12	*parvo, amplo*	**3d**	t_2	0.07	15.20	*parvo, amplo*
	c_1	64.47	64.88	*amplo,amplo*		c_1	65.44	65.53	*amplo,amplo*
	t_1	14.60	0.07	*amplo, parvo*		t_1	15.20	0.07	*amplo, parvo*
**2a**	t_2	0.07	14.86	*parvo, amplo*	**3e**	t_2	0.07	15.10	*parvo, amplo*
	c_1	65.84	65.80	*amplo,amplo*		c_1	65.51	65.38	*amplo,amplo*
	t_1	14.77	0.07	*amplo, parvo*		t_1	14.40	0.07	*amplo, parvo*
**2b**	t_2	0.07	13.97	*parvo, amplo*	**3f**	t_2	0.07	14.10	*parvo, amplo*
	c_1	64.98	65.05	*amplo,amplo*		c_1	65.18	64.97	*amplo,amplo*
	t_1	15.29	0.06	*amplo, parvo*		t_1	15.10	0.07	*amplo, parvo*
**2c**	t_2	0.07	15.53	*parvo, amplo*	**3g**	t_2	0.06	15.50	*parvo, amplo*
	c_1	65.59	65.63	*amplo,amplo*		c_1	65.51	65.46	*amplo,amplo*
	t_1	14.70	0.07	*amplo, parvo*		t_1	15.10	0.07	*amplo, parvo*
**2d**	t_2	0.07	14.4	*parvo, amplo*	**4a**	t_2	0.07	15.04	*parvo, amplo*
	c_1	65.30	65.44	*amplo,amplo*		c_1	65.16	65.15	*amplo,amplo*
	t_1	14.91	0.06	*amplo, parvo*		t_1	15.10	0.07	*amplo, parvo*
**2e**	t_2	0.07	15.46	*parvo, amplo*	**4b**	t_2	0.07	15.03	*parvo, amplo*
	c_1	65.50	65.62	*amplo,amplo*		c_1	65.15	65.13	*amplo,amplo*
	t_1	14.80	0.07	*amplo, parvo*		t_1	14.71	0.07	*amplo, parvo*
**2f**	t_2	0.07	15.02	*parvo, amplo*	**4c**	t_2	0.07	14.66	*parvo, amplo*
	c_1	65.50	65.60	*amplo,amplo*		c_1	64.88	64.82	*amplo,amplo*
	t_1	15.12	0.07	*amplo, parvo*		t_1	13.25	0.07	*amplo, parvo*
**2g**	t_2	0.07	15.14	*parvo, amplo*	**4d**	t_2	0.08	14.00	*parvo, amplo*
	c_1	65.71	65.65	*amplo,amplo*		c_1	63.70	63.43	*amplo,amplo*
		–	–			t_1	14.86	0.07	*amplo, parvo*
		–	–		**4e**	t_2	0.07	15.01	*parvo, amplo*
		–	–			c_1	65.17	65.25	*amplo,amplo*
		θ_1/θ_1'	θ_2/θ_2'	Descritor			θ_1/θ_1'	θ_2/θ_2'	Descritor
	t_1	14.33	0.08	*amplo, parvo*		t_1	12.75	0.08	*amplo, parvo*
**5a**	t_2	0.07	12.74	*parvo, amplo*	**5d**	t_2	0.08	11.94	*parvo, amplo*
	c_1	64.63	64.72	*amplo,amplo*		c_1	62.78	62.94	*amplo,amplo*
	t_1	14.70	0.10	*amplo, parvo*		t_1	14.16	0.09	*amplo, parvo*
**5b**	t_2	0.07	9.55	*parvo, amplo*	**5e**	t_2	0.07	11.71	*parvo, amplo*
	c_1	64.06	64.24	*amplo,amplo*		c_1	62.05	61.71	*amplo,amplo*
	t_1	11.94	0.07	*amplo, parvo*			–	–	
**5c**	t_2	0.08	13.80	*parvo, amplo*			–	–	
	c_1	66.31	65.63	*amplo, amplo*			–	–	

2. Analysis of akamptisomer pairs: structural insights

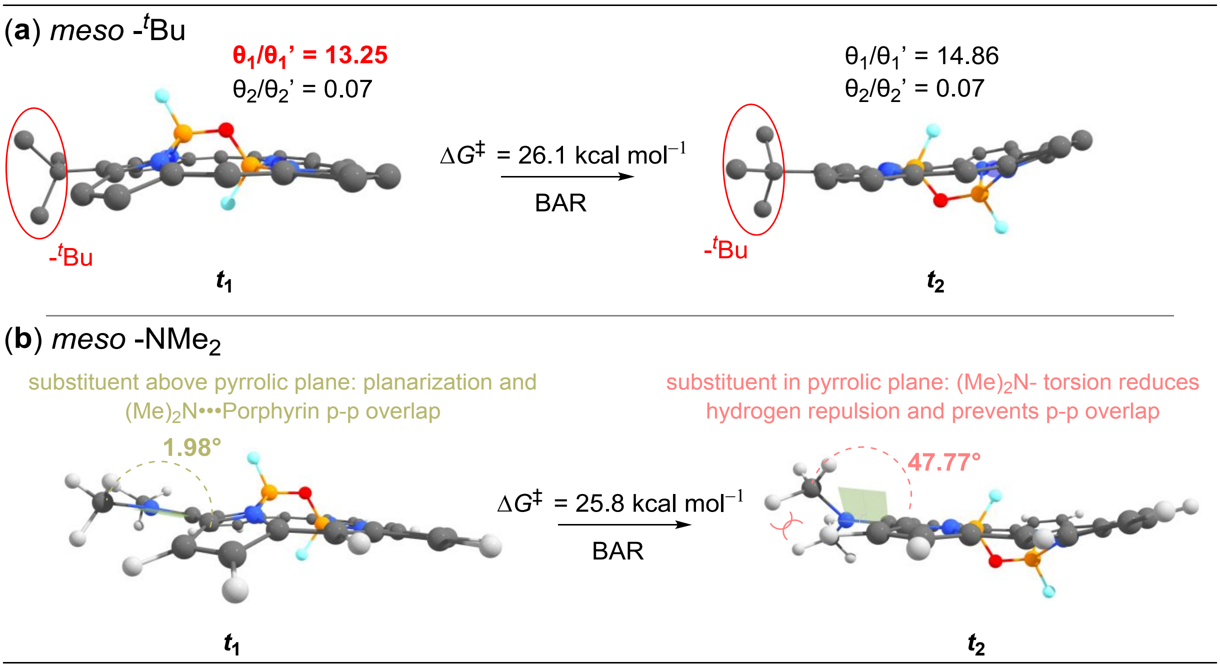

Figure S2: Optimized structure at B3LYLP-D3/6-31+G** level of (**a**) **4d** — *meso*-tert-butyl and (**b**) **3b** – *meso* dimethylamine (-NMe_2).

3. Absorption profile: light interaction

3.1 UV-Vis spectra: oscillator strength

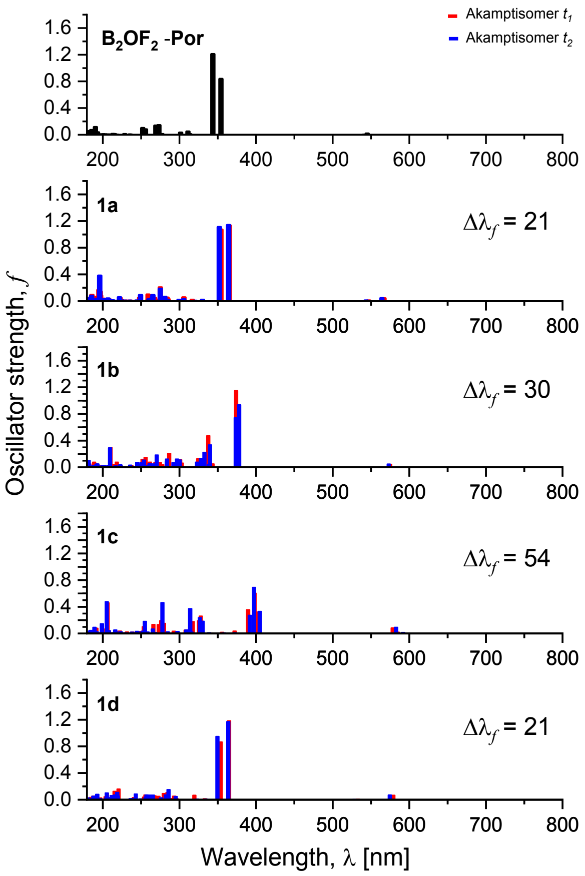

Figure S3: Theoretical UV-Vis absorption spectra in terms of oscillator strength *f* calculated at TD-CAM-B3LYP/6-31+G** for structural *variation 1* and referential non-substituted system (B_2OF_2-Por, in black). Red lines correspond to isomer $\boldsymbol{t_1}$, and blue lines to $\boldsymbol{t_2}$.

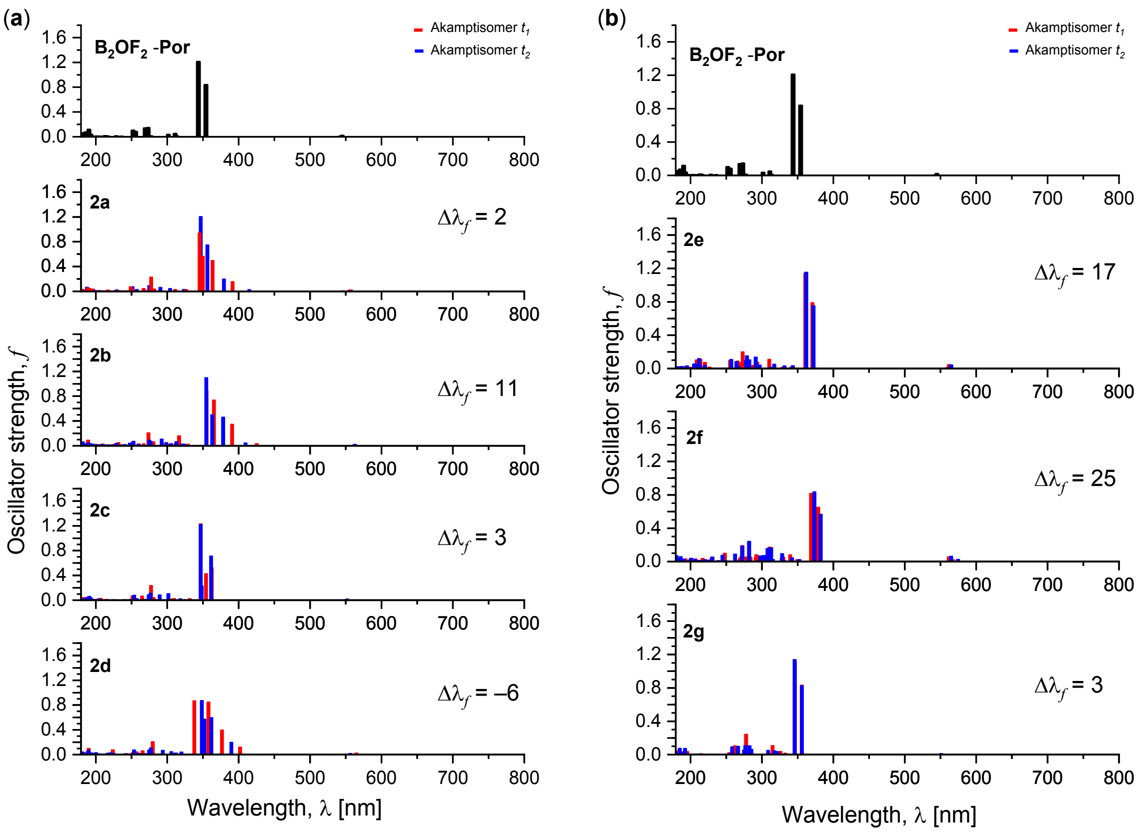

Figure S4: Theoretical UV-Vis absorption spectra in terms of oscillator strength *f* calculated at TD-CAM-B3LYP/6-31+G** for structural *variation 2* (**a**) electron donor groups, and (**b**) electron withdrawer groups. Referential non-substituted system, B_2OF_2-Por, represented in black. Red lines correspond to isomer $\boldsymbol{t_1}$, and blue lines to $\boldsymbol{t_2}$.

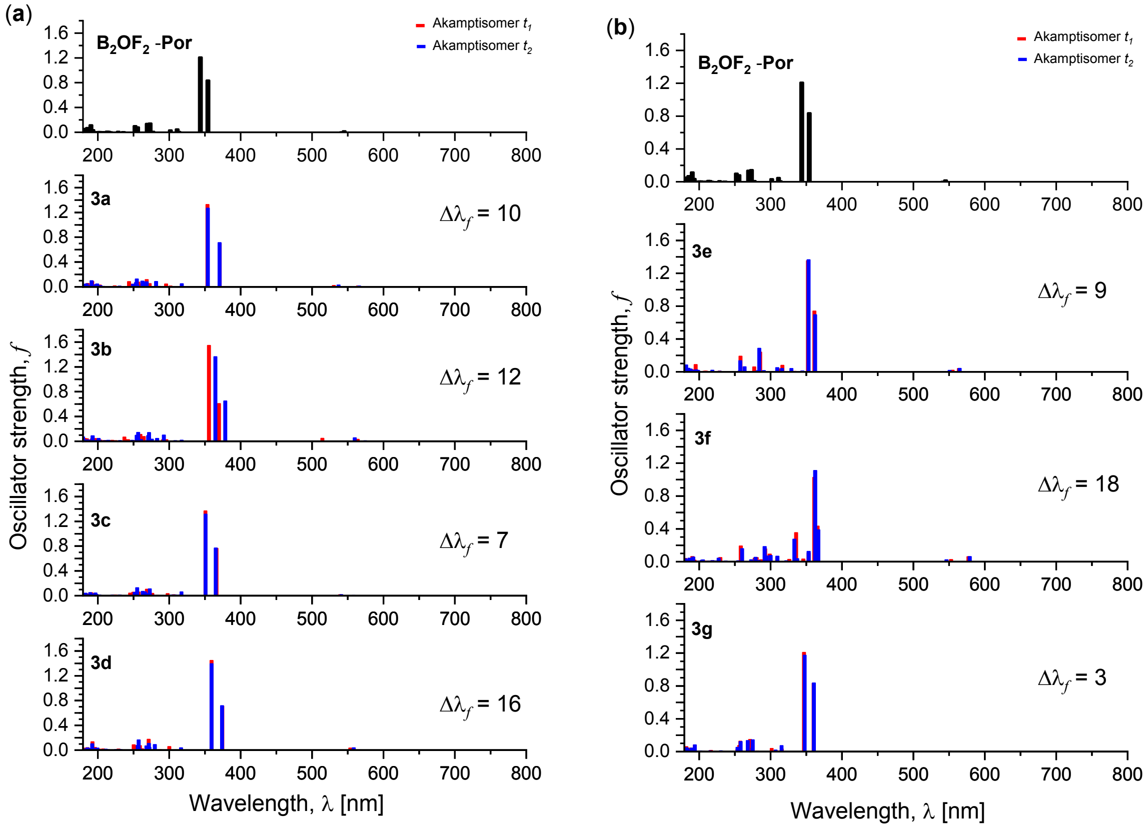

Figure S5: Theoretical UV-Vis absorption spectra in terms of oscillator strength *f* calculated at TD-CAM-B3LYP/6-31+G** for structural *variation 3* (**a**) electron donor groups, and (**b**) electron withdrawer groups. Referential non-substituted system, B_2OF_2-Por, represented in black. Red lines correspond to isomer $\boldsymbol{t_1}$, and blue lines to $\boldsymbol{t_2}$.

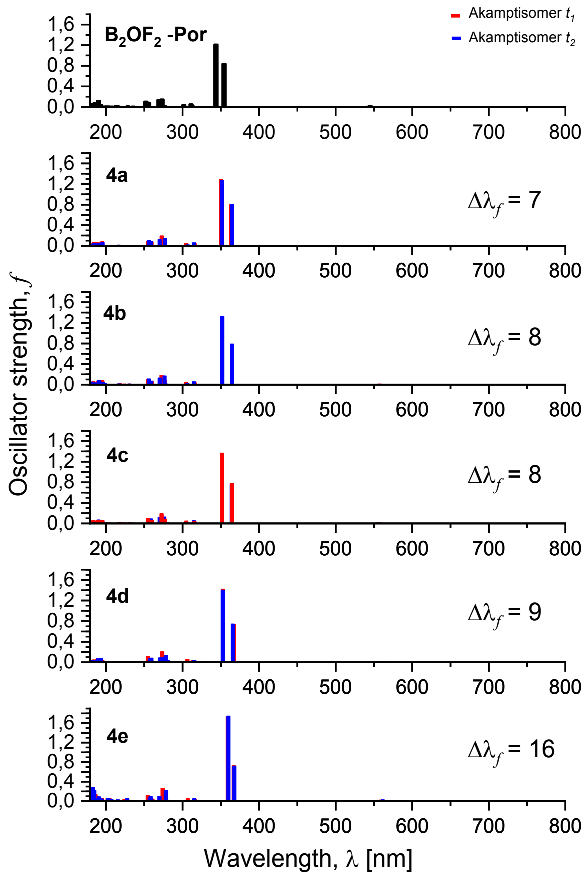

Figure S6: Theoretical UV-Vis absorption spectra in terms of oscillator strength *f* calculated at TD-CAM-B3LYP/6-31+G** for structural *variation 4* and referential non-substituted system (B_2OF_2-Por, in black). Red lines correspond to isomer $\boldsymbol{t_1}$, and blue lines to $\boldsymbol{t_2}$.

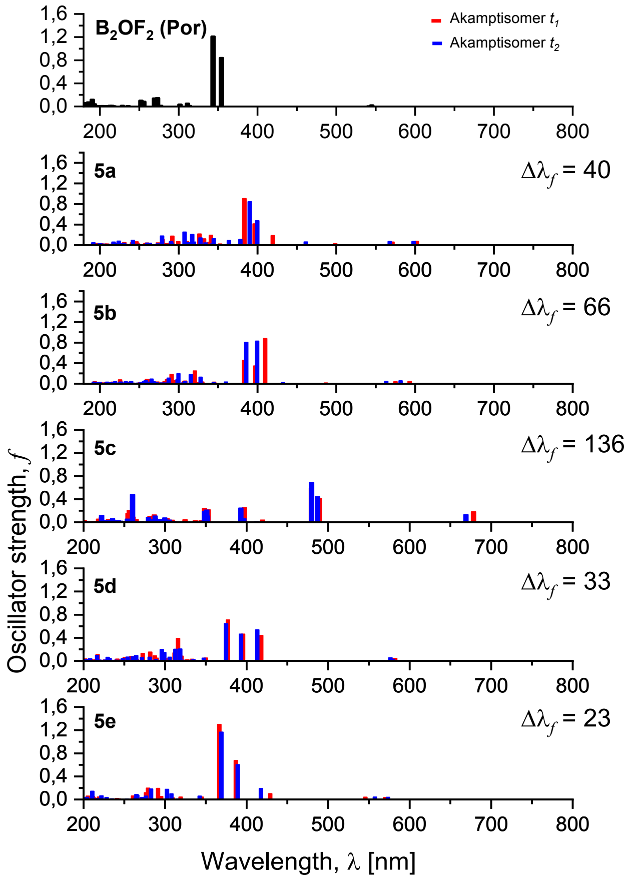

Figure S7: Theoretical UV-Vis absorption spectra in terms of oscillator strength *f* calculated at TD-CAM-B3LYP/6-31+G** for structural *variation 5* (push-pull devices) and referential non-substituted system (B_2OF_2-Por, in black). Red lines correspond to isomer $\boldsymbol{t_1}$, and blue lines to $\boldsymbol{t_2}$.

3.2 Vertical excitation: absorption spectra and electronic transitions

Table S2: Wavelength (λ), oscillator strength (*f*), and dominant electronic transitions of the first ten excited states obtained with CAM-B3LYP/6-31+G** for π conjugation extension in β,β-pyrrolic position – structural *variation 1*.

No	*λ* (nm)	*f*	Electronic transitions	λ (nm)	*f*	Electronic transitions
	**1a** – t_1			**1a** – t_2		
S_1	567 (*Q*)	0.0409	H→L (66%)	564 (*Q*)	0.0422	H→L (66%)
S_2	547 (*Q*)	0.0087	H–1→L (48%). H→L+1 (48%)	544 (*Q*)	0.0074	H–1→L (48%). H→L+1 (49%)
S_3	364 (*B*)	1.1334	H–1→L+1 (34%)	364 (*B*)	1.1383	H–1→L+1 (30%)
S_4	354 (*B*)	1.0699	H–1→L+1 (33%)	352 (*B*)	1.1090	H–1→L+1 (37%)
S_5	329	0.0125	H–3→L (78%)	330	0.0176	H–2→L (62%)
S_6	316	0.0192	H–5→LUMO (19%)	320	0.0037	H–3→LUMO (53%)
S_7	305	0.0552	H–2→L+1 (54%)	305	0.0269	H–2→L+1 (78%)
S_8	302	0.0008	H–3→L+1 (56%)	299	0.0159	H–3→L+1 (54%)

S_9	285	0.0269	H–7→LUMO (19%)	284	0.0173	H–4→L+1 (29%)
S_{10}	283	0.0496	H–7→LUMO (27%)	280	0.0623	H–7→LUMO (36%)
	**1b** – t_1			**1b** – t_2		
S_1	575 (*Q*)	0.0451	H→L (69%)	573 (*Q*)	0.0476	H→L (70%)
S_2	551 (*Q*)	0.0033	H–1→L (52%). H→L+1 (45%)	548 (*Q*)	0.0018	H–1→L (53%). H→L+1 (45%)
S_3	377 (*B*)	0.6022	H–1→L+1 (44%)	378 (*B*)	0.9361	H–1→L+1 (44%)
S_4	373 (*B*)	1.1488	H→L+1 (41%)	373 (*B*)	0.7441	H→L+2 (26%)
S_5	343	0.0496	H–6→L+2 (41%)	339	0.3359	H→L+2 (28%)
S_6	337	0.4734	HOMO→L+2 (50%)	338	0.0413	H–7→L+2 (44%)
S_7	333	0.0651	H–4→LUMO (63%)	332	0.2243	H–3→LUMO (20%)
S_8	324	0.1265	H–1→L+2 (57%)	327	0.1342	H–1→L+2 (55%)
S_9	318	0.0107	H–5→LUMO (43%)	322	0.0778	H–5→LUMO (30%)
S_{10}	303	0.0624	H–4→L+1 (61%)	302	0.015	H–3→L+1 (31%)
	**1c** – t_1			**1c** – t_2		
S_1	585 (*Q*)	0.0404	H→L (64%)	592 (*Q*)	0.0162	H→L (70%)
S_2	578 (*Q*)	0.0830	H–1→L (61%). H→L+1 (30%)	583 (*Q*)	0.0928	H–1→L (65%). H→L+1 (31%)
S_3	401 (*B*)	0.3215	H–1→L+1 (41%)	404 (*B*)	0.3323	H–1→L+1 (40%)
S_4	398 (*B*)	0.6055	H→L+1 (30%)	397 (*B*)	0.6915	H→L+1 (27%)
S_5	371	0.3567	H–6→L (26%)	391	0.2729	H–7→L (48%)
S_6	371	0.0361	H–9→LUMO (23%)	372	0.0122	H–10→LUMO (38%)
S_7	355	0.0187	H–2→LUMO (34%)	357	0.0097	H–2→LUMO (52%)
S_8	341	0.0028	H–2→LUMO (37%)	338	0.0128	H–3→LUMO (36%)
S_9	327	0.2615	HOMO→L+2 (51%)	330	0.1845	HOMO→L+2 (30%)
S_{10}	325	0.1898	H–1→L+2 (50%)	326	0.2369	H–1→L+2 (37%)
	**1d** – t_1			**1d** – t_2		
S_1	579 (*Q*)	0.0673	H→L (74%)	574 (*Q*)	0.071	H→L (74%)
S_2	533 (*Q*)	0.0051	H–1→L (50%). H→L+1 (47%)	528 (*Q*)	0.0034	H–1→L (51%). H→L+1 (47%)
S_3	365 (*B*)	1.1841	H→L+1 (40%)	364 (*B*)	1.1713	H→L+1 (42%)
S_4	354 (*B*)	0.8649	H–1→L+1 (55%)	349 (*B*)	0.948	H–1→L+1 (58%)
S_5	333	0.0135	H–3→L (79%)	334	0.0085	H–2→L (50%)
S_6	319	0.0665	H–2→LUMO (43%)	322	0.0075	H–3→LUMO (50%)
S_7	296	0.0169	H–3→L+1 (73%)	295	0.0493	H–3→L+1 (55%)
S_8	293	0.0527	H–4→LUMO (29%)	294	0.0073	H–4→LUMO (24%)
S_9	290	0.0158	H–4→L+1 (34%).	285	0.151	H–5→LUMO (30%)
S_{10}	284	0.1064	HOMO→L+2 (38%)	284	0.0134	HOMO→L+2 (39%)

Table S3: Wavelength (λ), oscillator strength (*f*) and dominant electronic transitions of the first ten excited states obtained with CAM-B3LYP/6-31+G** for substitutions in β,β-pyrrolic position – structural *variation 2*.

No	λ (nm)	*f*	Electronic transitions	λ (nm)	*f*	Electronic transitions
			EDG			
	**2a** – t_1			**2a** – t_2		
S_1	557 (*Q*)	0.0218	H→L (58%)	556 (*Q*)	0.0191	H→L (61%)
S_2	546 (*Q*)	0.0077	H–1→L (47%) H→L+1 (46%)	549 (*Q*)	0.0022	H–1→L (52%). H→L+1 (46%)
S_3	391 (*B*)	0.1566	H–2→L+1 (50%)	415 (*B*)	0.0273	H–2→L+1 (45%)
S_4	363 (*B*)	0.4972	H–1→L+1 (39%)	379 (*B*)	0.1988	H–2→L (39%)

S_5	350 (*B*)	0.5651	H–2→L (46%)	356 (*B*)	0.7491	H–1→L+1 (45%)
S_6	345	0.9418	H–1→L (32%)	347	1.2053	H→L+1 (48%)
S_7	326	0.0273	H–3→LUMO (90%)	323	0.0309	H–3→LUMO (83%)
S_8	311	0.0275	H–3→L+1 (80%)	311	0.0182	H–4→LUMO (86%)
S_9	308	0.0041	H–4→LUMO (70%)	304	0.0442	H–3→L+1 (72%)
S_{10}	281	0.0368	H–4→L+1 (40%)	290	0.0637	H–4→L+1 (49%)
	**2b – t_1**			**2b – t_2**		
S_1	563 (*Q*)	0.0204	H→L (61%)	562 (*Q*)	0.0228	H→L (61%)
S_2	559 (*Q*)	0.0020	H–1→L (47%). H→L+1 (49%)	554 (*Q*)	0.0033	H–1→L (49%). H→L+1 (48%)
S_3	425 (*B*)	0.032	H–2→L+1 (44%)	410 (*B*)	0.0451	H–2→L+1 (41%)
S_4	391 (*B*)	0.3466	H–1→L+1 (28%)	378 (*B*)	0.4607	H–1→L+1 (27%)
S_5	365 (*B*)	0.7359	H–1→L+1 (37%)	363 (*B*)	0.5003	H–2→L (29%)
S_6	355	0.8919	H→L+1 (38%)	355	1.0973	H→L+1 (42%)
S_7	329	0.0248	H–4→LUMO (55%)	323	0.0239	H–4→LUMO (52%)
S_8	316	0.1591	H–3→L+1 (39%)	321	0.0257	H–4→LUMO (29%)
S_9	311	0.0342	H–5→LUMO (25%)	312	0.0653	H–5→LUMO (41%)
S_{10}	303	0.0262	H–4→L+1 (36%)	305	0.0318	H–4→L+1 (73%)
	**2c – t_1**			**2c – t_2**		
S_1	552 (*Q*)	0.0172	H→L (57%)	552 (*Q*)	0.0169	H→L (58%)
S_2	548 (*Q*)	0.0045	H–1→L (49%). H→L+1 (44%)	547 (*Q*)	0.0035	H–1→L (50%). H→L+1 (46%)
S_3	362 (*B*)	0.5224	H–1→L+1 (26%)	361 (*B*)	0.7113	H–1→L+1 (40%)
S_4	354 (*B*)	0.4274	H–1→L+1 (32%)	348 (*B*)	0.2279	H–2→L+1 (36%)
S_5	347	1.2321	H→L+1 (48%)	347	1.2195	H→L+1 (46%)
S_6	331	0.0247	H–2→LUMO (41%)	332	0.0076	H–2→L+1 (36%)
S_7	324	0.0072	H–3→LUMO (72%)	318	0.0228	H–3→LUMO (59%)
S_8	309	0.0261	H–3→L+1 (89%)	306	0.0042	H–4→LUMO (45%)
S_9	306	0.0185	H–4→LUMO (72%)	301	0.1046	H–3→L+1 (33%)
S_{10}	280	0.0411	H–6→LUMO (53%)	289	0.0844	H–4→L+1 (49%)
	**2d – t_1**			**2d – t_2**		
S_1	560 (*Q*)	0.0182	H→L (61%)	558 (*Q*)	0.0188	H→L (61%)
S_2	556 (*Q*)	0.0182	H–1→L (49%). H→L+1 (47%)	552 (*Q*)	0.001	H–1→L (49%). H→L+1 (47%)
S_3	430 (*B*)	0.0182	H–2→L (44%)	417 (*B*)	0.0258	H–2→L (43%)
S_4	394 (*B*)	0.0182	H–2→L+1 (34%)	383 (*B*)	0.2705	H–2→L (33%)
S_5	361 (*B*)	0.0182	H–1→L+1 (39%)	359 (*B*)	0.7976	H–1→L+1 (36%)
S_6	349	1.1581	H→L+1 (38%)	349	1.1805	H→L+1 (36%)
S_7	327	0.0108	H–3→LUMO (92%)	324	0.0175	H–3→LUMO (85%)
S_8	310	0.0304	H–3→L+1 (85%)	311	0.0207	H–4→LUMO (83%)
S_9	308	0.0115	H–4→LUMO (73%)	305	0.0382	H–3→L+1 (72%)
S_{10}	282	0.0389	H–6→LUMO (30%)	291	0.0621	H–4→L+1 (49%)
EWG						
	**2e – t_1**			**2e – t_2**		
S_1	562 (*Q*)	0.0465	H–1→L (59%)	565 (*Q*)	0.0441	H–1→L (37%)
S_2	560 (*Q*)	0.0114	H→L (59%)	563 (*Q*)	0.0197	H→L (38%)
S_3	371 (*B*)	0.7882	H→L+1 (36%)	372 (*B*)	0.7508	H→L+1 (44%)
S_4	361 (*B*)	1.1413	H–1→L+1 (34%)	362 (*B*)	1.1546	H–1→L+1 (34%)
S_5	341	0.0195	H–2→L (66%)	343	0.0334	H–1→L+1 (42%)
S_6	329	0.0156	H–3→LUMO (61%)	331	0.0309	H–3→LUMO (75%)
S_7	317	0.0337	H–2→L+1 (47%)	317	0.0518	H–2→L+1 (76%)
S_8	310	0.1096	H–4→LUMO (37%)	312	0.0134	H–3→L+1 (57%)
S_9	293	0.0745	H–5→LUMO (30%)	296	0.0405	H–4→LUMO (61%)
S_{10}	287	0.0374	H–4→LUMO (28%)	291	0.1372	H–5→LUMO (43%)

	**2f** – ***t***$_1$			**2f** – ***t***$_2$		
S_1	568 (*Q*)	0.0216	H→L (68%)	575 (*Q*)	0.0256	H→L (70%)
S_2	562 (*Q*)	0.0563	H–1→L (63%)	565 (*Q*)	0.063	H–1→L (64%)
S_3	379 (*B*)	0.6501	H→L+1 (41%)	382 (*B*)	0.5671	H→L+1 (38%)
S_4	369 (*B*)	0.8179	H–1→L+1 (40%)	373 (*B*)	0.8356	H–1→L+1 (36%)
S_5	349	0.0226	H–2→L (71%)	352	0.0243	H–2→L (50%)
S_6	339	0.0807	H–3→LUMO (25%)	342	0.0453	H–2→LUMO (26%)
S_7	329	0.0505	H–9→LUMO (10%)	331	0.0106	H–3→LUMO (22%)
S_8	327	0.0378	H–3→LUMO (36%)	328	0.0942	H–2→L+1 (64%)
S_9	314	0.0238	H–2→L+1 (75%)	315	0.0244	H–3→L+1 (33%)
S_{10}	310	0.1725	HOMO→L+2 (37%)	312	0.1668	HOMO→L+2 (33%)
	**2g** – ***t***$_1$			**2g** – ***t***$_2$		
S_1	551 (*Q*)	0.0112	H→L (47%)	551 (*Q*)	0.0127	H→L (52%)
S_2	546 (*Q*)	0.0035	H–1→L (43%)	546 (*Q*)	0.0021	H–1→L (47%)
S_3	356 (*B*)	0.833	H–1→L+1 (57%)	356 (*B*)	0.8243	H–1→L+1 (57%)
S_4	346 (*B*)	1.1098	H→L+1 (48%)	346 (*B*)	1.1395	H→L+1 (49%)
S_5	332	0.0171	H–2→L (59%)	333	0.0079	H–2→L (82%)
S_6	325	0.0381	H–3→LUMO (63%)	321	0.0356	H–3→LUMO (69%)
S_7	314	0.1082	H–3→L+1 (42%)	317	0.0454	H–2→L+1 (66%)
S_8	312	0.0132	H–3→L+1 (40%)	308	0.0506	H–3→L+1 (58%)
S_9	287	0.0077	H–4→LUMO (28%)	285	0.0612	H–6→LUMO (33%)
S_{10}	280	0.034	H–6→LUMO (64%)	282	0.1053	H–4→LUMO (31%)

Table S4: Wavelength (λ), oscillator strength (*f*) and dominant electronic transitions of the first ten excited states obtained with CAM-B3LYP/6-31+G** for substitutions in meso position – structural *variation 3*.

No	λ (nm)	*f*	Electronic transitions	λ (nm)	*f*	Electronic transitions
			EDG			
	**3a** – ***t***$_1$			**3a** – ***t***$_2$		
S_1	564 (*Q*)	0.0117	H→L (67%)	566 (*Q*)	0.012	H→L (67%)
S_2	530 (*Q*)	0.0211	H→L+1 (61%)	537 (*Q*)	0.0283	H→L+1 (64%)
S_3	371 (*B*)	0.704	H–1→L+1 (66%)	370 (*B*)	0.7123	H–1→L+1 (67%)
S_4	353 (*B*)	1.3265	H–1→L (61%)	354 (*B*)	1.2681	H–1→L (63%)
S_5	318	0.0044	H–2→L (81%)	317	0.0493	H–2→L (82%)
S_6	307	0.0	H–2→L+1 (85%)	311	0.0009	H–2→L+1 (83%)
S_7	301	0.0114	H–3→L+1 (69%)	305	0.0009	H–3→LUMO (69%)
S_8	295	0.0454	H–3→LUMO (52%)	304	0.0008	H–3→L+1 (62%)
S_9	285	0.0062	H–4→LUMO (50%)	281	0.0844	HOMO→L+2 (79%)
S_{10}	281	0.0004	HOMO→L+2 (61%)	277	0.0024	H–4→LUMO (48%)
	**3b** – ***t***$_1$			**3b** – ***t***$_2$		
S_1	564 (*Q*)	0.0311	H→L (72%)	574 (*Q*)	0.0066	H→L+1 (62%)
S_2	514 (*Q*)	0.046	H→L+1 (64%)	560 (*Q*)	0.0545	H→L (64%)
S_3	369 (*B*)	0.6059	H–1→L+1 (71%)	378 (*B*)	0.6497	H–1→L (62%)
S_4	356 (*B*)	1.5458	H–1→L (63%)	364 (*B*)	1.3638	H–1→L+1 (60%)
S_5	315	0.0018	H–3→L (56%)	317	0.0155	H–3→L+1 (64%)
S_6	299	0.0	H–2→L+1 (44%)	316	0.002	H–3→LUMO (70%)
S_7	295	0.0241	H–2→L+1 (41%)	308	0.0091	H–4→LUMO (31%)
S_8	291	0.0215	H–2→LUMO (47%)	307	0.004	H–4→LUMO (30%)
S_9	284	0.0029	HOMO→L+2 (63%)	292	0.0985	H–2→LUMO (45%)
S_{10}	283	0.0159	H–4→LUMO (39%)	283	0.0447	H–2→L+1 (55%)
	**3c** – ***t***$_1$			**3c** – ***t***$_2$		

S_1	558 (*Q*)	0.003	H→L (45%)	558 (*Q*)	0.0046	H→L (40%)
S_2	539 (*Q*)	0.0136	H→L+1 (43%)	541 (*Q*)	0.0163	H→L+1 (37%)
S_3	366 (*B*)	0.7627	H–1→L+1 (52%)	365 (*B*)	0.7688	H–1→L+1 (42%)
S_4	351 (*B*)	1.3675	H–1→L (50%)	351 (*B*)	1.3156	H–1→L (38%)
S_5	317	0.0165	H–2→L (57%)	317	0.0613	H–2→L (80%)
S_6	312	0.0	H–2→L+1 (73%)	313	0.0073	H–2→L+1 (81%)
S_7	306	0.0138	H–3→LUMO (40%)	308	0.0097	H–3→LUMO (68%)
S_8	297	0.0322	H–3→L+1 (31%)	303	0.0105	H–3→L+1 (66%)
S_9	283	0.004	H–4→LUMO (30%)	277	0.007	H–4→LUMO (41%)
S_{10}	276	0.0366	H–4→L+1 (19%)	272	0.1084	H–4→L+1 (26%)
	**3d – t_1**			**3d – t_2**		
S_1	567 (*Q*)	0.0011	H→L+1 (59%)	567 (*Q*)	0.0014	H→L+1 (60%)
S_2	553 (*Q*)	0.0322	H→L (62%)	558 (*Q*)	0.0362	H→L (63%)
S_3	374 (*B*)	0.7095	H–1→L (59%)	374 (*B*)	0.7151	H–1→L (60%)
S_4	359 (*B*)	1.4464	H–1→L+1 (61%)	359 (*B*)	1.3941	H–1→L+1 (62%)
S_5	316	0.0054	H–2→L+1 (72%)	319	0.0003	H–2→L (89%)
S_6	316	0.0003	H–2→LUMO (90%)	316	0.0373	H–2→L+1 (85%)
S_7	309	0.0049	H–3→LUMO (78%)	309	0.0004	H–3→LUMO (68%)
S_8	300	0.0573	H–3→L+1 (43%)	307	0.0017	H–3→L+1 (65%)
S_9	287	0.0065	H–4→L+1 (53%)	279	0.0884	HOMO→L+2 (44%)
S_{10}	284	0.004	H–4→LUMO (44%)	279	0.004	H–4→L+1 (53%)
			EWG			
	**3e – t_1**			**3e – t_2**		
S_1	564 (*Q*)	0.0039	H→L (66%)	565 (*Q*)	0.0406	H→L (67%)
S_2	555 (*Q*)	0.0184	H–1→L (59%)	551 (*Q*)	0.0161	H–1→L (57%)
S_3	361 (*B*)	0.7387	H–1→L+1 (65%)	362 (*B*)	0.3946	H–1→L+1 (64%)
S_4	352 (*B*)	1.3516	H→L+1 (57%)	353 (*B*)	1.3647	H→L+1 (56%)
S_5	344	0.0061	H–2→L (90%)	344	0.0086	H–2→L (87%)
S_6	333	0.0042	H–3→LUMO (85%)	329	0.0395	H–3→LUMO (81%)
S_7	316	0.0785	H–4→LUMO (50%)	315	0.0385	H–2→L+1 (42%)
S_8	311	0.0349	H–2→L+1 (77%)	309	0.0494	H–4→LUMO (40%)
S_9	288	0.0061	H–6→LUMO (84%)	290	0.0134	H–6→LUMO (71%)
S_{10}	285	0.239	H–5→LUMO (52%)	284	0.2856	H–5→LUMO (67%)
	**3f – t_1**			**3f – t_2**		
S_1	577 (*Q*)	0.0607	H→L (75%)	579 (*Q*)	0.0623	H→L (75%)
S_2	552 (*Q*)	0.0262	H–1→L (60%)	546 (*Q*)	0.0222	H–1→L (59%)
S_3	366 (*B*)	0.4322	H–1→L+1 (56%)	367 (*B*)	0.3873	H–1→L+1 (55%)
S_4	351 (*B*)	1.0269	H→L+1 (46%)	362 (*B*)	1.1098	H→L+1 (47%)
S_5	353	0.1049	H–2→L (74%)	353	0.1246	H–2→L (74%)
S_6	345	0.0319	H–3→LUMO (83%)	337	0.0359	H–3→LUMO (81%)
S_7	335	0.3531	H–4→LUMO (20%)	333	0.274	H–7→LUMO (16%)
S_8	326	0.0272	H–4→LUMO (36%)	321	0.011	H–2→L+1 (31%)
S_9	309	0.0541	HOMO→L+2 (41%)	309	0.0656	H–2→L+1 (40%)
S_{10}	298	0.0919	H–5→LUMO (53%)	299	0.0757	H–6→LUMO (58%)
	**3g – t_1**			**3g – t_2**		
S_1	551 (*Q*)	0.0023	H→L (50%). H–1→L (50%)	552 (*Q*)	0.0021	H→L (50%). H–1→L (50%)
S_2	544 (*Q*)	0.0013	H→L (55%). H–1→L+1 (45%)	547 (*Q*)	0.0021	H→L (55%). H–1→L+1 (44%)
S_3	360 (*B*)	0.8328	H–1→L (50%). H→L+1 (50%)	361 (*B*)	0.8347	H–1→L (50%). H→L+1 (50%)
S_4	347 (*B*)	1.208	H–1→L+1 (54%). H→L (43%)	348 (*B*)	1.1742	H–1→L+1 (54%). H→L (41%)
S_5	321	0.0004	H–2→L (91%)	323	0.0000	H–2→L (88%)

S_6	315	0.0311	H–2→L+1 (82%)	315	0.0721	H–2→L+1 (87%)
S_7	311	0.0076	H–3→LUMO (81%)	313	0.0011	H–3→LUMO (75%)
S_8	301	0.038	H–3→L+1 (56%)	307	0.0178	H–3→L+1 (70%)
S_9	281	0.0054	H–4→L+1 (49%)	278	0.0019	H–4→L+1 (71%)
S_{10}	274	0.0863	H–4→LUMO (33%)	274	0.1429	H–4→LUMO (37%)

Table S5: Wavelength (λ), oscillator strength (*f*) and dominant electronic transitions of the first ten excited states obtained with CAM-B3LYP/6-31+G** for substitution in meso position – structural *variation 4*.

No	λ (nm)	*f*	Electronic transitions	λ (nm)	*f*	Electronic transitions
	**4a – t_1**			**4a – t_2**		
S_1	556 (*Q*)	0.0062	H–1→L (54%). H→L+1 (46%)	557 (*Q*)	0.006	H–1→L (53%). H→L+1 (46%)
S_2	552 (*Q*)	0.0037	H→L (56%). H–1→L+1 (44%)	555 (*Q*)	0.0046	H→L (56%). H–1→L+1 (44%)
S_3	364 (*B*)	0.7982	H–1→L (47%). H→L+1 (54%)	364 (*B*)	0.7966	H–1→L (47%). H→L+1 (54%)
S_4	350 (*B*)	1.2856	H→L (42%). H–1→L+1 (55%)	351 (*B*)	1.2728	H→L (41%). H–1→L+1 (55%)
S_5	327	0.0000	H–2→L (91%)	327	0.0000	H–2→L (91%)
S_6	316	0.0063	H–3→LUMO (83%)	315	0.0022	H–3→LUMO (79%)
S_7	315	0.0372	H–2→L+1 (79%)	315	0.0545	H–2→L+1 (89%)
S_8	304	0.0456	H–3→L+1 (52%)	306	0.0159	H–3→L+1 (63%)
S_9	281	0.0022	H–4→L+1 (43%)	279	0.0077	H–4→L+1 (48%)
S_{10}	276	0.071	H–4→LUMO (40%)	276	0.0431	H–6→LUMO (40%)
	**4b – t_1**			**4b – t_2**		
S_1	558 (*Q*)	0.0067	H–1→L (54%). H→L+1 (46%)	557 (*Q*)	0.0063	H–1→L (54%). H→L+1 (46%)
S_2	554 (*Q*)	0.0044	H→L (56%). H–1→L+1 (44%)	556 (*Q*)	0.0058	H→L (56%). H–1→L+1 (43%)
S_3	364 (*B*)	0.781	H–1→L (46%). H→L+1 (55%)	364 (*B*)	0.7845	H–1→L (47%). H→L+1 (54%)
S_4	351 (*B*)	1.3129	H→L (42%). H–1→L+1 (55%)	352 (*B*)	1.3206	H→L (41%). H–1→L+1 (56%)
S_5	327	0.0000	H–2→L (92%)	327	0.0001	H–2→L (91%)
S_6	316	0.0046	H–3→LUMO (83%)	315	0.0024	H–3→LUMO (79%)
S_7	315	0.0355	H–2→L+1 (82%)	314	0.0526	H–2→L+1 (89%)
S_8	304	0.0451	H–3→L+1 (53%)	306	0.0168	H–3→L+1 (62%)
S_9	281	0.0027	H–4→L+1 (43%)	279	0.0086	H–4→L+1 (45%)
S_{10}	276	0.0828	H–4→LUMO (37%)	276	0.0166	H–6→LUMO (45%)
	**4c – t_1**			**4c – t_2**		
S_1	557 (*Q*)	0.0066	H–1→L (54%). H→L+1 (45%)	557 (*Q*)	0.0061	H–1→L (54%). H→L+1 (46%)
S_2	554 (*Q*)	0.0051	H→L (56%). H–1→L+1 (44%)	555 (*Q*)	0.0059	H→L (56%). H–1→L+1 (43%)
S_3	364 (*B*)	0.7697	H–1→L (45%). H→L+1 (53%)	364 (*B*)	0.7721	H–1→L (45%). H→L+1 (52%)
S_4	352 (*B*)	1.3638	H→L (41%). H–1→L+1 (54%)	352 (*B*)	1.3597	H→L (40%). H–1→L+1 (54%)
S_5	327	0.0002	H–2→L (91%)	327	0.0006	H–2→L (92%)
S_6	316	0.0045	H–3→LUMO (81%)	315	0.0031	H–3→LUMO (79%)

S_7	315	0.0369	H–2→L+1 (78%)	314	0.0507	H–2→L+1 (89%)
S_8	304	0.0452	H–3→L+1 (52%)	305	0.0199	H–3→L+1 (62%)
S_9	281	0.0026	H–6→LUMO (31%)	279	0.0189	H–4→L+1 (41%)
S_{10}	276	0.0812	H–4→LUMO (37%)	276	0.0555	H–6→LUMO (37%)
	**4d – t_1**			**4d – t_2**		
S_1	562 (*Q*)	0.0105	H–1→L (45%)	559 (*Q*)	0.0102	H→L (37%)
S_2	559 (*Q*)	0.0075	H→L (46%)	558 (*Q*)	0.0051	H–1→L (33%)
S_3	367 (*B*)	0.7349	H→L+1 (46%)	365 (*B*)	0.7402	H→L+1 (40%)
S_4	353 (*B*)	1.4178	H–1→L+1 (46%)	352 (*B*)	1.4018	H–1→L+1 (41%)
S_5	329	0.0001	H–2→L (91%)	329	0.0045	H–2→L (92%)
S_6	320	0.0035	H–3→LUMO (84%)	316	0.0142	H–3→LUMO (77%)
S_7	313	0.0329	H–2→L+1 (74%)	315	0.0357	H–2→L+1 (85%)
S_8	306	0.0524	H–3→L+1 (45%)	305	0.0183	H–3→L+1 (58%)
S_9	281	0.007	H–6→LUMO (44%)	280	0.029	H–6→LUMO (38%)
S_{10}	278	0.0676	H–4→LUMO (40%)	278	0.1256	H–4→LUMO (25%)
	**4e – t_1**			**4e – t_2**		
S_1	559 (*Q*)	0.0073	H–1→L (50%).<br>H→L+1 (42%)	561 (*Q*)	0.024	H→L (58%)
S_2	559 (*Q*)	0.0183	H→L (53%)	560 (*Q*)	0.0059	H→L+1 (45%).<br>H→L (54%)
S_3	366 (*B*)	0.7281	H→L+1 (54%)	367 (*B*)	0.7163	H→L+1 (55%)
S_4	359 (*B*)	1.7343	H–1→L+1 (56%)	360 (*B*)	1.742	H–1→L+1 (57%)
S_5	328	0.0005	H–4→L (89%)	329	0.0003	H–4→L (86%)
S_6	318	0.0028	H–5→LUMO (81%)	316	0.0043	H–5→LUMO (79%)
S_7	315	0.034	H–4→L+1 (78%)	315	0.0459	H–4→L+1 (84%)
S_8	306	0.0477	H–5→L+1 (49%)	307	0.0118	H–5→L+1 (56%)
S_9	294	0.0038	H–2→LUMO (63%)	289	0.0039	H–2→LUMO (79%)
S_{10}	290	0.0092	H–3→LUMO (53%)	284	0.0025	H–3→LUMO (69%)

Table S6: Wavelength (λ), oscillator strength (*f*) and dominant electronic transitions of the first ten excited states obtained with CAM-B3LYP/6-31+G** for the push-pull systems (structural *variation 5*).

No	λ (nm)	*f*	Electronic transitions	λ (nm)	*f*	Electronic transitions
	**5a – t_1**			**5a – t_2**		
S_1	602 (*Q*)	0.0721	H→LUMO (65%)	598 (*Q*)	0.0689	H→LUMO (72%)
S_2	571 (*Q*)	0.0578	H-1→LUMO (58%)	567 (*Q*)	0.0675	H-1→LUMO (67%)
S_3	498 (*Q*)	0.026	H-2→LUMO (63%)	461 (*Q*)	0.0595	H-2→LUMO (64%)
S_4	419 (*B*)	0.1831	H→L+1 (34%)	399 (*B*)	0.4717	H-1→L+1 (44%)
S_5	396 (*B*)	0.4135	H-2→L+1 (25%)	390 (*B*)	0.8423	H→L+1 (42%)
S_6	383 (*B*)	0.9035	H-1→L+1 (38%)	378 (*B*)	0.107	H-2→L+1 (62%)
S_7	352	0.0219	H-4→LUMO (45%)	363	0.0876	H-3→LUMO (62%)
S_8	340	0.1905	H-5→LUMO (20%)	344	0.123	H-5→LUMO (28%)
S_9	338	0.0535	H-4→LUMO (30%)<br>H-3→LUMO (30%)	342	0.0199	H-4→LUMO (57%)
S_{10}	332	0.1212	H-1→L+2 (17%)	334	0.0422	H-1→L+2 (27%)
	**5b – t_1**			**5b – t_2**		
S_1	593 (*Q*)	0.0438	H→LUMO (61%)	581 (*Q*)	0.054	H→LUMO (65%)
S_2	575 (*Q*)	0.0368	H-1→LUMO (58%)	563 (*Q*)	0.045	H-1→LUMO (64%)
S_3	486	0.0132	H-2→LUMO (76%)	432	0.0199	H-2→LUMO (83%)
S_4	409 (*B*)	0.8774	H→L+1 (58%)	399 (*B*)	0.8273	H->L+1 (57%)
S_5	397 (*B*)	0.3452	H-1→L+1 (48%)	385 (*B*)	0.803	H-1→L+1 (57%)

S_6	383 (*B*)	0.4553	H-2→L+1 (62%)	359 (*B*)	0.0313	H-3→LUMO (65%)
S_7	347	0.0022	H-3→LUMO (38%)	351	0.0019	H-2→L+1 (91%)
S_8	345	0.0301	H-4→LUMO (14%)	344	0.0222	H-4→LUMO (15%)
S_9	329	0.0317	H-4→LUMO (33%)	330	0.0078	H-4→LUMO (26%)
S_{10}	324	0.0257	H-3→LUMO (25%)	327	0.1256	H-1→L+2 (20%)
	**5c** – t_1			**5c** – t_2		
S_1	704 (*Q*)	0.0028	H-1→LUMO (56%)	700 (*Q*)	0.0014	H-1→LUMO (55%)
S_2	678 (*Q*)	0.1806	H→LUMO (75%)	669 (*Q*)	0.1301	H→LUMO (69%)
S_3	489	0.4121	H→L+1 (53%)	487	0.4413	H→L+1 (52%)
S_4	479 (*B*)	0.5936	H-1→L+1 (68%)	479 (*B*)	0.6883	H-1→L+1 (66%)
S_5	419	0.0374	H-2→LUMO (85%)	412	0.012	H-2→LUMO (86%)
S_6	397	0.2513	H-3→LUMO (79%)	396	0.0609	H-3→LUMO (39%)
S_7	394	0.0762	H-2→L+1 (78%)	392	0.2448	H-2→L+1 (49%)
S_8	381	0.0087	H→L+2 (55%)	380	0.0015	H→L+2 (52%)
S_9	352	0.2147	H-4→LUMO (29%)	352	0.1074	H-5→LUMO (40%)
S_{10}	350	0.0507	H-5→LUMO (45%)	351	0.2103	H-3→L+1 (19%)
	**5d** – t_1			**5d** – t_2		
S_1	582 (*Q*)	0.0428	H→LUMO (56%)	579 (*Q*)	0.0262	H→LUMO (42%)
S_2	579 (*Q*)	0.037	H-1→LUMO (34%)	576 (*Q*)	0.0528	H→LUMO (27%)
S_3	417 (*B*)	0.4396	H-2→LUMO (46%)	413 (*B*)	0.5373	H-2→LUMO (41%)
S_4	395 (*B*)	0.4615	H-1→LUMO (30%)	393 (*B*)	0.4633	H-1→L+1 (30%)
S_5	377 (*B*)	0.7072	H-1→L+1 (26%)	374 (*B*)	0.6456	H-2→LUMO (34%)
S_6	349	0.0496	H-2→L+1 (85%)	347	0.046	H-2→L+1 (83%)
S_7	333	0.0276	H-9→L+2 (41%)	334	0.0205	H-9→L+2 (35%)
S_8	327	0.018	H-3→LUMO (65%)	321	0.0116	H-3→LUMO (38%)
S_9	319	0.0814	H-3→L+1 (53%)	318	0.2056	H-3→L+1 (36%)<br>HOMO→L+2 (36%)
S_{10}	315	0.3887	HOMO→L+2 (39%)	312	0.2018	HOMO→L+2 (27%)
	**5e** – t_1			**5e**– t_2		
S_1	570 (*Q*)	0.0317	H→LUMO (42%)	573 (*Q*)	0.038	H→LUMO (41%)
S_2	545 (*Q*)	0.0392	H-1→LUMO (36%)	557 (*Q*)	0.0424	H-1→LUMO (37%)
S_3	429	0.1008	H-2→LUMO (77%)	417	0.1889	H-2→LUMO (71%)
S_4	386 (*B*)	0.6751	H→L+1 (53%)	389 (*B*)	0.6035	H→L+1 (51%)
S_5	366 (*B*)	1.2992	H-1→L+1 (56%)	369 (*B*)	1.1653	H-1→L+1 (57%)
S_6	344	0.0419	H-2→L+1 (94%)	342	0.0582	H-2→L+1 (84%)
S_7	332	0.0033	H-3→LUMO (81%)	331	0.0017	H-3→LUMO (62%)
S_8	319	0.0423	H-3→L+1 (60%)	315	0.009	H-4→LUMO (63%)
S_9	309	0.0286	H-4→L+1 (60%)	307	0.0971	H-4→L+1 (55%)
S_{10}	295	0.0517	H-4→LUMO (46%)	302	0.1747	H-5→LUMO (25%)

4. Energy diagram of frontier orbitals

(**a**)

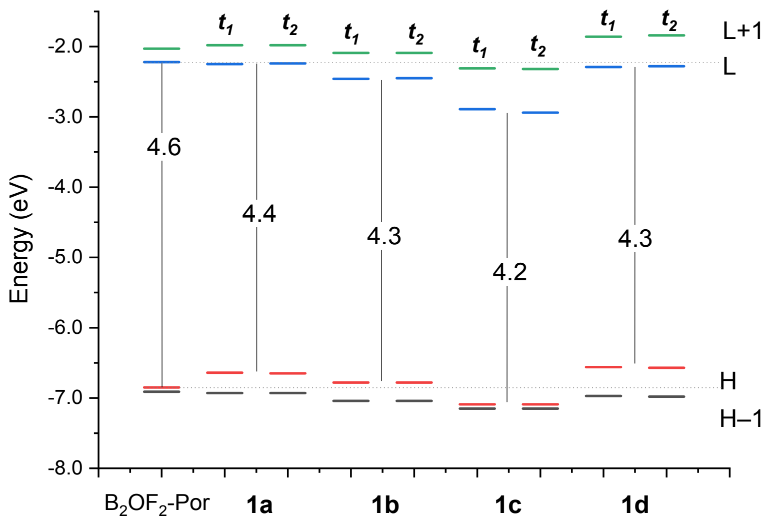

(**b**)

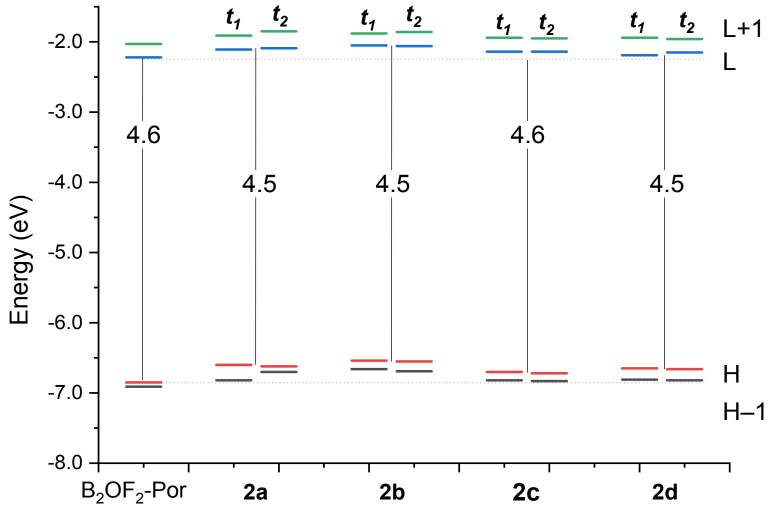

(**c**)

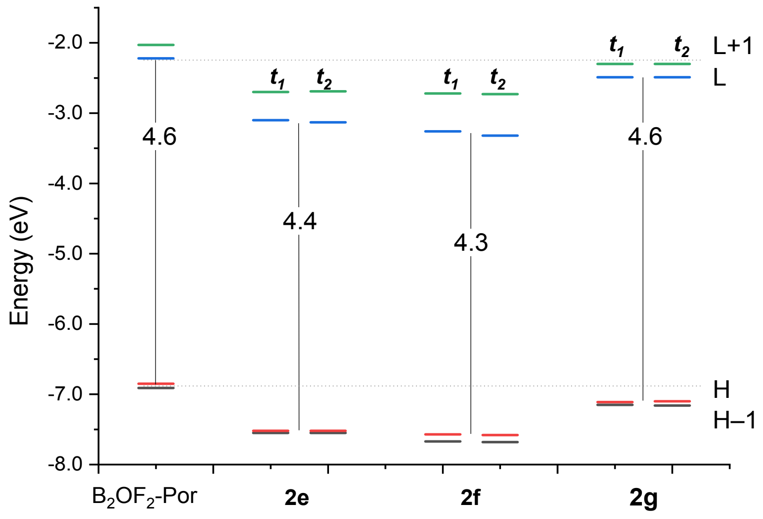

Figure S8: Energy diagrams for the frontier orbitals obtained at CAM-B3LYP/6-31+G** level for β,β substitutions: (**a**) structural *variation 1*, (**b**) structural *variation 2* — electron donor groups, and (**c**) structural *variation 2* — electron withdrawer groups. H refers do HOMO and L to LUMO.

(**a**)

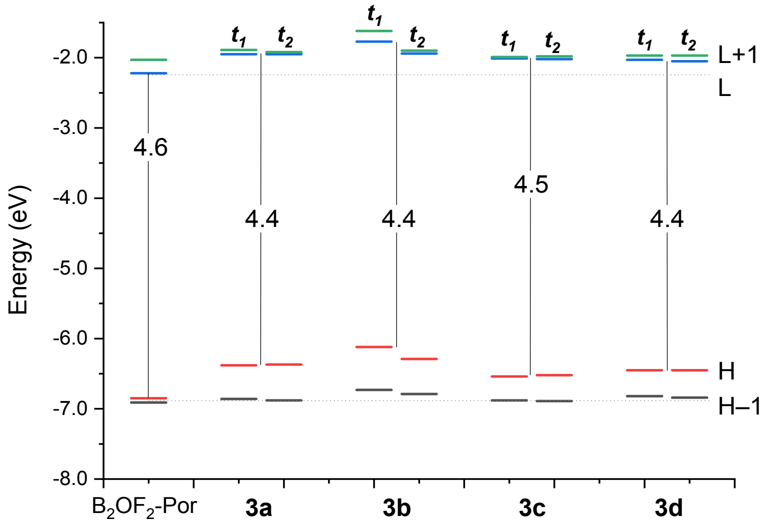

(**b**)

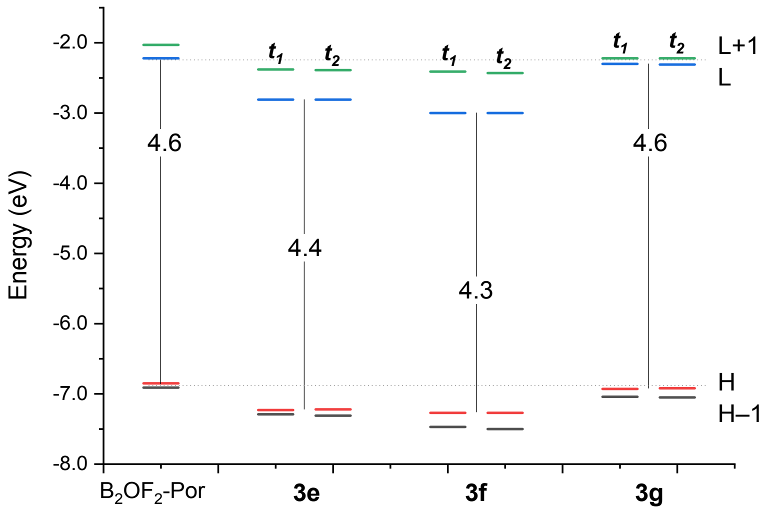

(**c**)

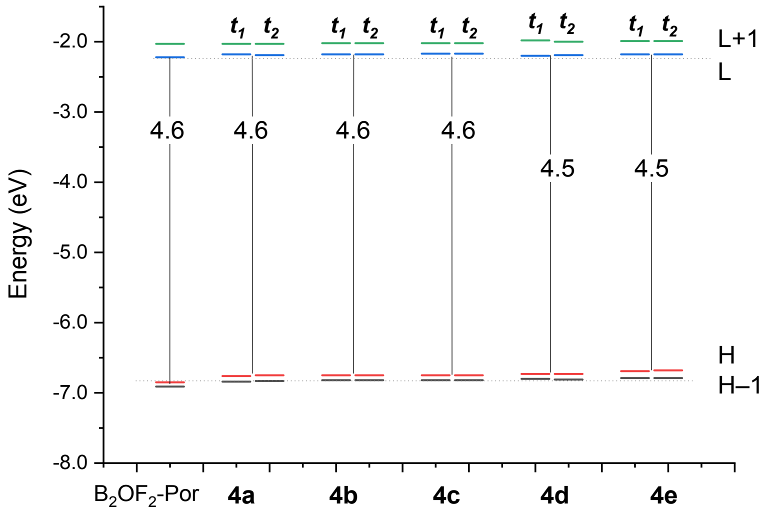

Figure S9: Energy diagrams for the frontier orbitals obtained at CAM-B3LYP/6-31+G** level for *meso* substitutions: (**a**) structural *variation 3* — electron donor groups, (**b**) structural *variation 3* — electron withdrawer groups, and (**c**) structural *variation 4*. H refers to HOMO and L to LUMO.

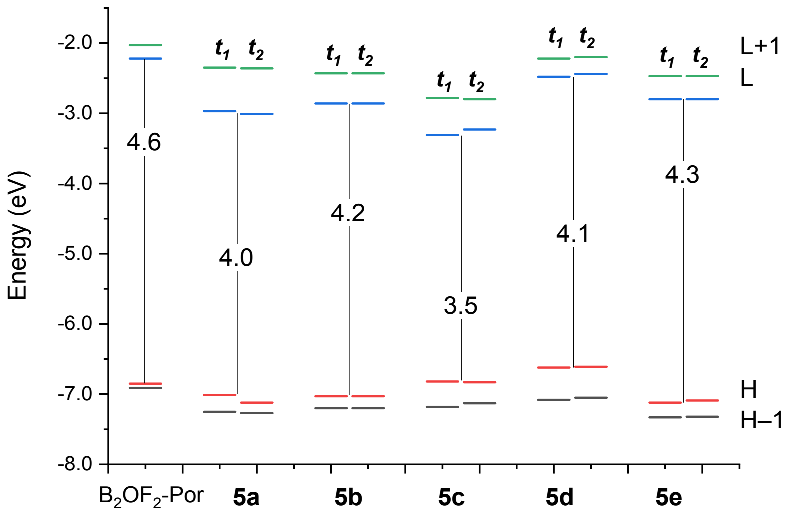

Figure S10: Energy diagrams for the frontier orbitals obtained at CAM-B3LYP/6-31+G** level for push-pull devices 5a – 5e. H refers to HOMO and L to LUMO.

5. Molecular orbitals — spatial visualization

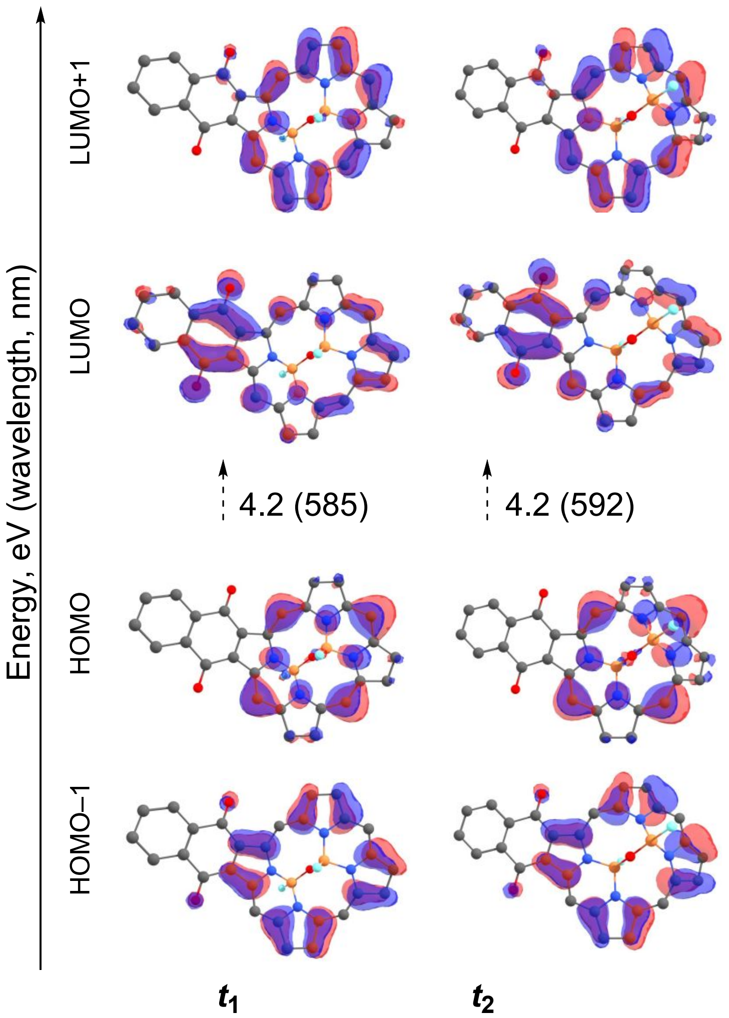

Figure S11: Frontier molecular orbitals (contour value = 0.03) for akamptisomer $\boldsymbol{t_1}$ of for β,β-naphthoquinone **1c**. The intramolecular charge transfer character D → A is evident, where D represents the electron donor unit (porphyrin) and A signifies the acceptor unit (naphtoquinone). Kohn-Sham molecular orbitals were obtained using CAM-B3LYP/6-31+G** in the optimized structure (B3LYP-D3/6-31+G**).

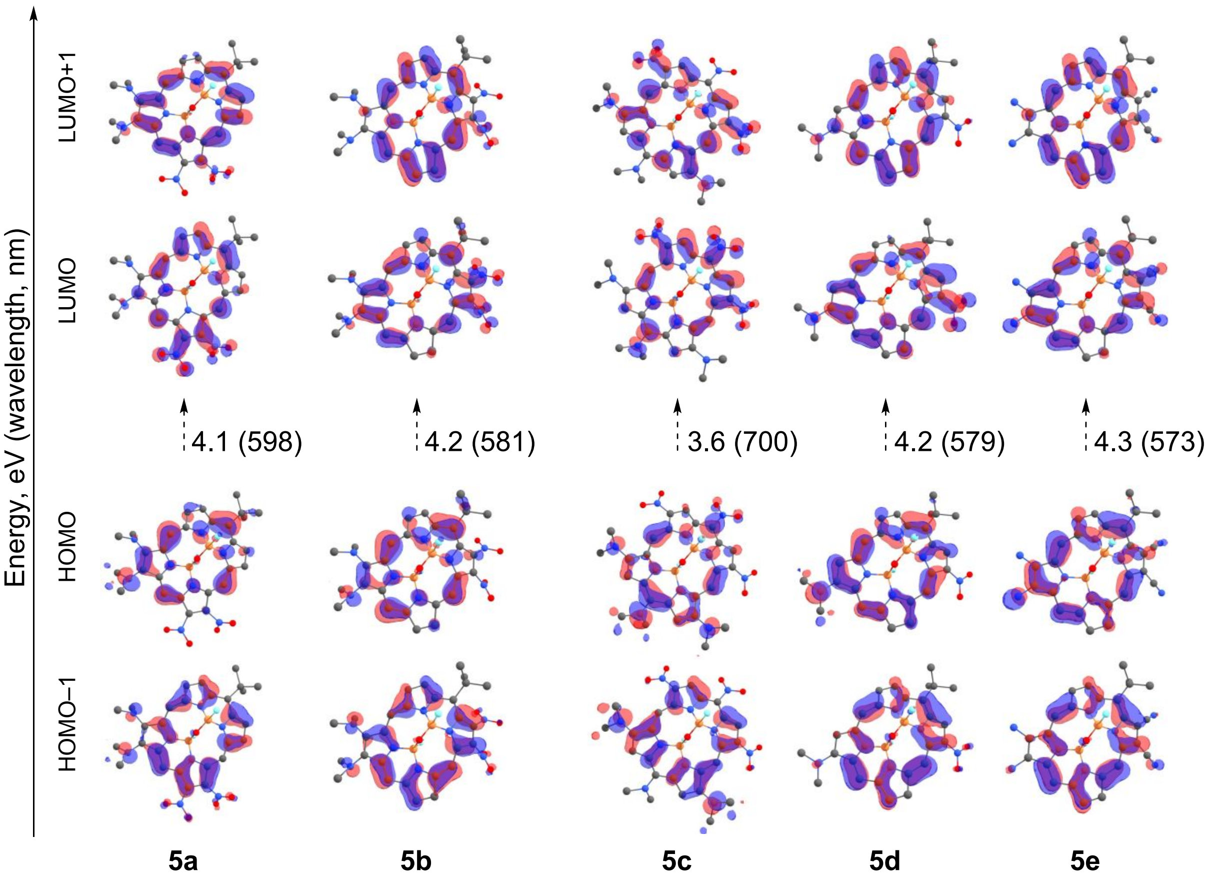

Figure S12: Frontier molecular orbitals (contour value set to 0.03) for akamptisomer t_2 of **5a**–**5e**. The intramolecular charge transfer character D → π → A is evident, where D represents the electron donor unit (-NMe_2, -NH_2, and porphyrin), π denotes the connector (typically the porphyrin framework), and A signifies the acceptor unit (-NO_2 and -CN). Kohn-Sham molecular orbitals were obtained using CAM-B3LYP/6-31+G** in the optimized structure (B3LYP-D3/6-31+G**).

6. Matrices of optimized structures

B_2OF_2-Por – referential system

9	1.868916000	-0.000366000	2.222755000
5	-0.943027000	0.000044000	-0.074131000
5	1.188197000	0.000004000	0.993092000
8	-0.181948000	0.000302000	1.109839000
9	-0.219678000	0.000277000	-1.299169000
6	-2.573164000	-3.383805000	0.001296000
6	-1.396560000	-2.534625000	0.017385000
6	-0.079947000	-3.031761000	0.046619000
6	1.232317000	-2.508782000	0.000353000

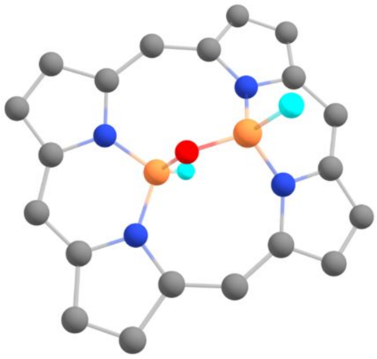

6	2.344844000	-3.347759000	-0.416513000
6	3.450831000	-2.555036000	-0.562272000
6	3.047346000	-1.218790000	-0.212520000
6	3.728358000	0.000360000	-0.337077000
6	3.047234000	1.219446000	-0.212296000
6	3.450268000	2.555712000	-0.562477000
6	2.344047000	3.348120000	-0.416702000
6	1.231841000	2.508920000	0.000557000
6	-0.080576000	3.031629000	0.046847000
6	-1.397072000	2.534295000	0.017630000
6	-2.573879000	3.383306000	0.001373000
6	-3.681539000	2.580965000	-0.083464000
6	-3.211553000	1.218661000	-0.125255000
6	-3.903229000	-0.000348000	-0.169271000
6	-3.211207000	-1.219248000	-0.125242000
6	-3.680966000	-2.581568000	-0.083539000
7	-1.845880000	-1.244884000	-0.072431000
7	1.729954000	-1.241543000	0.167311000
7	1.729964000	1.241886000	0.167824000
7	-1.846322000	1.244560000	-0.072240000
1	-2.546840000	-4.464175000	0.054367000
1	2.259030000	-4.403369000	-0.638459000
1	4.432721000	-2.841675000	-0.913575000
1	4.432015000	2.842583000	-0.913983000
1	2.257879000	4.403662000	-0.638835000
1	-4.718747000	-2.884854000	-0.107215000
1	-0.070077000	-4.118410000	-0.002020000
1	4.766331000	0.000416000	-0.649505000
1	-0.070887000	4.118276000	-0.001868000
1	-4.986098000	-0.000536000	-0.207672000
1	-4.719231000	2.884249000	-0.107239000
1	-2.547869000	4.463679000	0.054374000

E = –1313.8963554 a.u.
H = –1313.8963554 + *0.316059* (thermal correction 6-31+G**) a. u.
G = –1313.8963554 + *0.251260* (thermal correction 6-31+G**) a. u.

STRUCTURAL VARIATION 1

1a– akamptisomer *amplo,parvo* (***t*$_1$**)

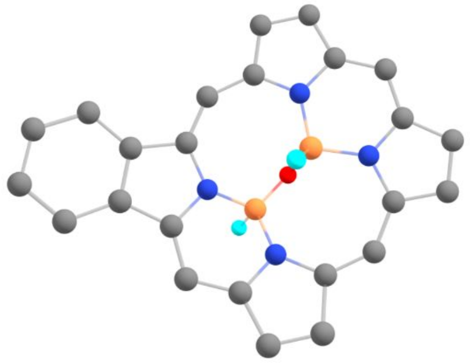

9	0.772541000	-0.047893000	-1.253377000
5	-0.149079000	0.882439000	1.093341000

5	1.435112000	-0.470708000	-0.067383000
8	0.923880000	0.027696000	1.146464000
9	-0.602119000	1.314771000	2.350825000
6	0.927792000	4.196362000	-0.437196000
6	1.305515000	2.851709000	-0.037617000
6	2.651978000	2.441515000	-0.097801000
6	3.371164000	1.229558000	-0.158524000
6	4.811402000	1.159865000	-0.284694000
6	5.171749000	-0.162251000	-0.367985000
6	3.958759000	-0.931984000	-0.299451000
6	3.738574000	-2.319962000	-0.292502000
6	2.450192000	-2.840077000	-0.135081000
6	1.972126000	-4.194037000	-0.013769000
6	0.616815000	-4.123770000	0.178733000
6	0.229893000	-2.724949000	0.182531000
6	-1.107836000	-2.290411000	0.304229000
6	-1.820624000	-1.079099000	0.272485000
6	-3.265340000	-1.056230000	-0.008722000
6	-3.651796000	0.297398000	-0.145437000
6	-2.454768000	1.084702000	0.072249000
6	-2.232465000	2.451932000	-0.083424000
6	-0.928343000	2.976860000	-0.076048000
6	-0.439009000	4.267450000	-0.477931000
7	0.135835000	2.173732000	0.226969000
7	2.912280000	-0.059664000	-0.185568000
7	1.370053000	-2.004516000	-0.022007000
7	-1.422259000	0.231379000	0.371805000
1	1.634038000	4.965309000	-0.721640000
1	5.466001000	2.021212000	-0.300686000
1	6.167996000	-0.573064000	-0.458688000
1	2.589830000	-5.080553000	-0.059699000
1	-0.071216000	-4.947857000	0.313520000
1	-1.052698000	5.101660000	-0.789411000
1	3.315389000	3.295035000	-0.219511000
1	4.582275000	-2.994637000	-0.378387000
1	-1.781392000	-3.142637000	0.335168000
1	-3.064010000	3.101703000	-0.329792000
6	-5.900351000	-0.373928000	-0.627314000
6	-4.967679000	0.647855000	-0.464026000
6	-5.520864000	-1.723699000	-0.493207000
1	-5.257402000	1.688293000	-0.576718000
1	-6.265962000	-2.502179000	-0.626921000
6	-4.204991000	-2.076139000	-0.197337000
1	-3.928872000	-3.122720000	-0.113451000
1	-6.931703000	-0.130220000	-0.863661000

$E = -1467.6159577$ a.u.

$H = -1467.6159577 + 0.365828$ (thermal correction 6-31+G**) a. u.
$G = -1467.6159577 + 0.294837$ (thermal correction 6-31+G**) a. u.

1a – akamptisomer *parvo,amplo* (***t*$_2$**)

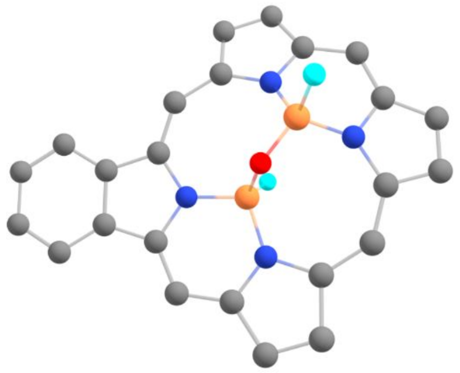

9	-2.199468000	-1.018712000	2.218118000
5	0.031181000	0.687644000	-0.083140000
5	-1.665815000	-0.609984000	0.983579000
8	-0.570723000	0.217168000	1.095964000
9	-0.525379000	0.240536000	-1.311163000
6	-0.751031000	4.363265000	0.009749000
6	-1.165479000	2.974562000	0.019747000
6	-2.509324000	2.570515000	0.055762000
6	-3.235982000	1.357015000	0.008835000
6	-4.629257000	1.347793000	-0.394738000
6	-5.026184000	0.043666000	-0.540660000
6	-3.891719000	-0.769440000	-0.204885000
6	-3.692172000	-2.154381000	-0.333953000
6	-2.414216000	-2.708767000	-0.218200000
6	-1.919013000	-4.010371000	-0.577719000
6	-0.557409000	-3.960653000	-0.445329000
6	-0.187796000	-2.619861000	-0.020613000
6	1.176448000	-2.236271000	0.036328000
6	1.927347000	-1.055600000	0.016344000
6	3.394379000	-1.023492000	0.011571000
6	3.791139000	0.333399000	-0.076509000
6	2.571232000	1.119384000	-0.122954000
6	2.370657000	2.498069000	-0.169251000
6	1.073626000	3.039534000	-0.126689000
6	0.616581000	4.404264000	-0.077156000
7	-0.017895000	2.224945000	-0.078280000
7	-2.854704000	0.048407000	0.164816000
7	-1.349748000	-1.923551000	0.158455000
7	1.516546000	0.250025000	-0.073656000
1	-1.431156000	5.202710000	0.069153000
1	-5.206652000	2.237798000	-0.607910000
1	-5.983317000	-0.325267000	-0.883404000
1	-2.523044000	-4.836045000	-0.928627000
1	0.154306000	-4.741912000	-0.677623000
1	1.254475000	5.277242000	-0.096808000
1	-3.177975000	3.427822000	0.019263000
1	-4.520187000	-2.783145000	-0.641027000
1	1.818543000	-3.111903000	-0.014544000
1	3.225286000	3.162848000	-0.203936000
6	4.358526000	-2.034819000	0.083666000
6	5.704872000	-1.673803000	0.062445000
1	6.466892000	-2.445351000	0.117404000

6	6.094792000	-0.323281000	-0.025648000
1	7.151167000	-0.072641000	-0.038383000
6	5.142562000	0.691657000	-0.094372000
1	5.444630000	1.732681000	-0.159777000
1	4.074759000	-3.079989000	0.159734000

E = –1467.616528 a.u.
H = –1467.616528 + *0.365828* (thermal correction 6-31+G**) a. u.
G = –1467.616528 + *0.294863* (thermal correction 6-31+G**) a. u.

1a – transition state (*TS*)

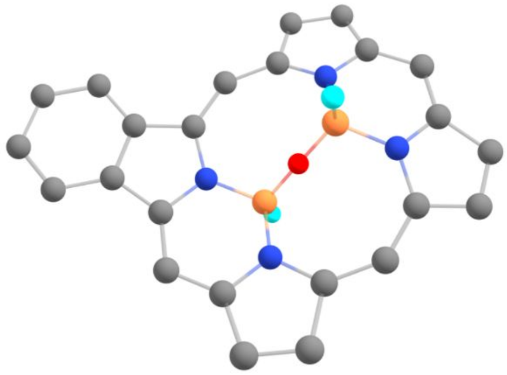

9	1.435792000	-0.505633000	-2.115450000
5	-0.169278000	0.822617000	0.768272000
5	1.588390000	-0.543008000	-0.709833000
8	0.704553000	0.130945000	0.034911000
9	-0.041704000	0.782947000	2.176448000
6	0.678603000	4.305373000	-0.314369000
6	1.092834000	2.938214000	-0.030376000
6	2.450127000	2.516814000	-0.108396000
6	3.265933000	1.339769000	-0.129728000
6	4.701918000	1.376889000	0.094348000
6	5.180386000	0.086595000	0.108497000
6	4.054168000	-0.780968000	-0.134730000
6	3.886052000	-2.184912000	-0.046544000
6	2.605532000	-2.772291000	-0.016106000
6	2.133019000	-4.081072000	0.374434000
6	0.762705000	-4.007832000	0.442311000
6	0.348999000	-2.648167000	0.118427000
6	-1.020044000	-2.224251000	0.181754000
6	-1.840302000	-1.065053000	0.194676000
6	-3.301963000	-1.111199000	0.001161000
6	-3.788572000	0.225284000	0.005590000
6	-2.633691000	1.086046000	0.217366000
6	-2.457933000	2.478466000	0.149596000
6	-1.164899000	3.058796000	0.106584000
6	-0.692146000	4.376784000	-0.249159000
7	-0.073074000	2.271018000	0.273471000
7	2.966009000	0.015492000	-0.320017000
7	1.502478000	-1.992470000	-0.209908000
7	-1.557207000	0.267115000	0.375866000
1	1.356920000	5.102674000	-0.589201000
1	5.268395000	2.280515000	0.278187000
1	6.193782000	-0.235196000	0.306540000
1	2.757360000	-4.931805000	0.611733000
1	0.085090000	-4.796391000	0.742710000
1	-1.315924000	5.234372000	-0.461557000
1	3.092910000	3.394454000	-0.168558000

1	4.756944000	-2.808547000	0.120982000
1	-1.650888000	-3.109381000	0.244013000
1	-3.324866000	3.113384000	0.008969000
6	-6.014473000	-0.569885000	-0.395913000
6	-5.142787000	0.500638000	-0.199945000
6	-5.538229000	-1.893904000	-0.402527000
1	-5.509477000	1.522796000	-0.202794000
1	-6.236239000	-2.710474000	-0.560777000
6	-4.184370000	-2.174008000	-0.216186000
1	-3.832473000	-3.200719000	-0.242932000
1	-7.072941000	-0.381939000	-0.548250000

E= –1467.5734879 a.u.
H= –1467.5734879 + *0.364217* (thermal correction 6-31+G**) a. u.
G= –1467.5734879 + *0.293606* (thermal correction 6-31+G**) a. u.
f = 183.8355i

1a– akamptisomer *amplo, amplo* (c_1)

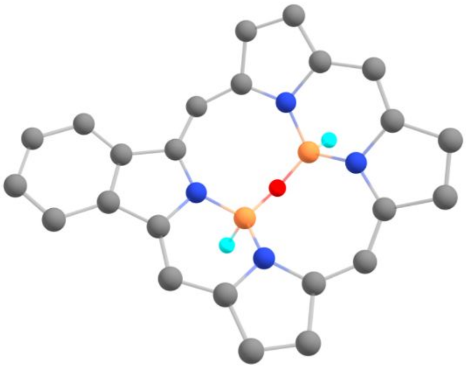

9	2.560873000	-1.283145000	1.899108000
5	-0.205873000	0.941651000	1.007852000
5	1.773544000	-0.708544000	0.874143000
8	0.812450000	0.117300000	1.395776000
9	-0.834648000	1.538851000	2.124271000
6	0.999133000	4.227639000	-0.408979000
6	1.351467000	2.882398000	-0.009287000
6	2.688716000	2.473591000	0.030768000
6	3.330987000	1.236141000	-0.145324000
6	4.675377000	1.164966000	-0.671334000
6	4.923933000	-0.138260000	-1.020094000
6	3.752867000	-0.885996000	-0.676160000
6	3.465173000	-2.245148000	-0.854706000
6	2.206993000	-2.752223000	-0.537795000
6	1.662869000	-4.066333000	-0.721321000
6	0.368852000	-4.022771000	-0.275917000
6	0.088416000	-2.666792000	0.152538000
6	-1.231919000	-2.253311000	0.410703000
6	-1.904573000	-1.035824000	0.304048000
6	-3.344541000	-0.977214000	0.028611000
6	-3.648684000	0.356987000	-0.322484000
6	-2.412871000	1.100307000	-0.208887000
6	-2.152242000	2.441056000	-0.460722000
6	-0.849291000	2.947760000	-0.362984000
6	-0.348660000	4.258589000	-0.653208000
7	0.189623000	2.143232000	0.045729000
7	2.817170000	-0.035414000	-0.125023000
7	1.236331000	-1.936974000	0.017631000
7	-1.414041000	0.242884000	0.204883000

1	1.715443000	5.024345000	-0.561024000
1	5.316144000	2.022995000	-0.826393000
1	5.809496000	-0.544043000	-1.490092000
1	2.188970000	-4.904756000	-1.157400000
1	-0.364837000	-4.817678000	-0.300820000
1	-0.941029000	5.087275000	-1.016608000
1	3.380005000	3.310378000	-0.028702000
1	4.214601000	-2.898557000	-1.286283000
1	-1.909949000	-3.098531000	0.481880000
1	-2.956399000	3.094970000	-0.776431000
6	-4.944808000	0.730846000	-0.696344000
6	-5.936200000	-0.246529000	-0.696595000
1	-6.953191000	0.017123000	-0.970654000
6	-5.637445000	-1.578591000	-0.344874000
1	-6.430344000	-2.320409000	-0.349297000
6	-4.343888000	-1.957985000	0.006615000
1	-4.130165000	-2.989696000	0.268378000
1	-5.174278000	1.755375000	-0.973298000

E= –1467.6048813a.u.
H= –1467.6048813 + 0.365446 (thermal correction 6-31+G**) a. u.
G= –1467.6048813 + 0.293847 (thermal correction 6-31+G**) a. u.

1b – akamptisomer *amplo,parvo* ($\boldsymbol{t}_1$)

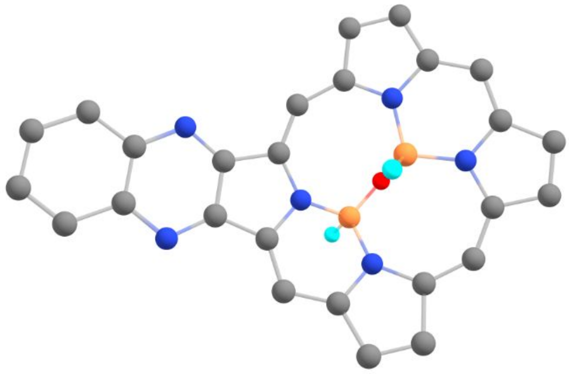

9	-1.480232000	0.096089000	-1.235264000
5	-0.801882000	-0.977135000	1.131524000
5	-2.137884000	0.590168000	-0.074441000
8	-1.749319000	0.014893000	1.151584000
9	-0.458219000	-1.473805000	2.397980000
6	-2.221290000	-4.103282000	-0.504691000
6	-2.439104000	-2.726102000	-0.098592000
6	-3.716441000	-2.142983000	-0.208919000
6	-4.269708000	-0.847223000	-0.276843000
6	-5.682697000	-0.591565000	-0.459506000
6	-5.867095000	0.766614000	-0.534072000
6	-4.569621000	1.372905000	-0.403592000
6	-4.174755000	2.721085000	-0.367526000
6	-2.838299000	3.071023000	-0.152871000
6	-2.195802000	4.352551000	-0.005060000
6	-0.868403000	4.109209000	0.234984000
6	-0.665528000	2.672962000	0.245446000
6	0.598038000	2.066586000	0.413900000
6	1.131514000	0.771573000	0.411688000
6	2.574955000	0.568149000	0.206732000
6	2.800537000	-0.841104000	0.065670000
6	1.503061000	-1.472616000	0.212347000
6	1.120446000	-2.796037000	0.024783000

6	-0.240854000	-3.143943000	-0.035555000
6	-0.875170000	-4.353094000	-0.483257000
7	-1.204596000	-2.209715000	0.231385000
7	-3.648475000	0.372458000	-0.262657000
7	-1.879711000	2.103093000	-0.003958000
7	0.582653000	-0.486743000	0.477997000
7	3.531680000	1.464978000	0.108757000
7	3.980103000	-1.373807000	-0.175100000
6	4.775620000	0.949837000	-0.107817000
6	4.999518000	-0.471981000	-0.252988000
6	5.882424000	1.835381000	-0.202794000
6	6.319785000	-0.941511000	-0.488086000
6	7.148621000	1.343625000	-0.429108000
6	7.368572000	-0.053393000	-0.572910000
1	-3.007518000	-4.769128000	-0.835448000
1	-6.440736000	-1.361448000	-0.517017000
1	-6.797728000	1.303238000	-0.658172000
1	-2.693249000	5.310735000	-0.068220000
1	-0.084307000	4.836326000	0.398720000
1	-0.360780000	-5.256360000	-0.781339000
1	-4.478770000	-2.900950000	-0.375402000
1	-4.921147000	3.498733000	-0.479574000
1	1.397459000	2.803269000	0.459178000
1	1.883218000	-3.534044000	-0.192236000
1	5.691655000	2.897949000	-0.090966000
1	6.464604000	-2.011930000	-0.593680000
1	7.990041000	2.026297000	-0.500121000
1	8.375164000	-0.419413000	-0.751388000

E= –1653.4109305 a.u.
H= –1653.4109305 + *0.390917* (thermal correction 6-31+G**) a. u.
G= –1653.4109305 + *0.314315* (thermal correction 6-31+G**) a. u.

1b – akamptisomer *parvo,amplo* (***t*₂**)

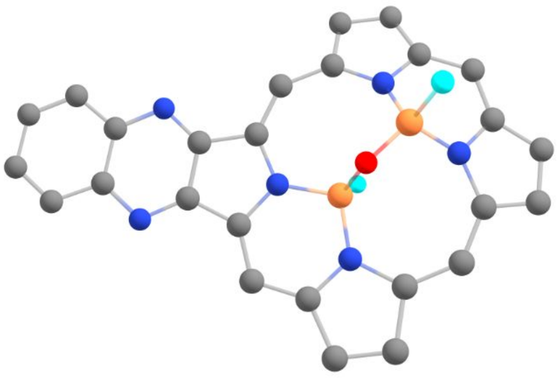

9	-2.863258000	-1.210529000	2.211980000
5	-0.851459000	0.757766000	-0.079177000
5	-2.378867000	-0.742055000	0.979422000
8	-1.391666000	0.212384000	1.095632000
9	-1.341218000	0.250328000	-1.310458000
6	-2.065752000	4.311325000	0.024903000
6	-2.309401000	2.882890000	0.028413000
6	-3.594302000	2.318257000	0.057953000
6	-4.169902000	1.026277000	0.004079000
6	-5.550848000	0.852068000	-0.404036000
6	-5.788838000	-0.489641000	-0.554478000
6	-4.566519000	-1.162445000	-0.216782000
6	-4.204592000	-2.514473000	-0.344018000

6	-2.871412000	-2.915289000	-0.221854000
6	-2.226633000	-4.152120000	-0.574111000
6	-0.880627000	-3.945270000	-0.434375000
6	-0.672530000	-2.569813000	-0.013562000
6	0.634961000	-2.024620000	0.045977000
6	1.223557000	-0.760536000	0.020670000
6	2.676686000	-0.559335000	0.007742000
6	2.919805000	0.854297000	-0.076461000
6	1.615271000	1.494481000	-0.115776000
6	1.259751000	2.838119000	-0.155028000
6	-0.093678000	3.219259000	-0.111680000
6	-0.712861000	4.517712000	-0.058113000
7	-1.079038000	2.277507000	-0.068673000
7	-3.634925000	-0.228020000	0.156569000
7	-1.907330000	-2.010024000	0.156100000
7	0.678053000	0.496418000	-0.067617000
7	3.638630000	-1.454608000	0.059999000
7	4.122051000	1.388761000	-0.104836000
6	4.900021000	-0.938178000	0.030758000
6	5.142351000	0.485934000	-0.050344000
6	6.009216000	-1.824454000	0.081190000
6	6.482519000	0.957180000	-0.076209000
6	7.294472000	-1.331178000	0.053816000
6	7.532673000	0.068111000	-0.025063000
1	-2.842305000	5.062353000	0.085291000
1	-6.229909000	1.667396000	-0.616110000
1	-6.694334000	-0.969255000	-0.900377000
1	-2.729404000	-5.043144000	-0.924806000
1	-0.080409000	-4.638242000	-0.657835000
1	-0.184729000	5.461124000	-0.072973000
1	-4.361361000	3.088805000	0.022631000
1	-4.951889000	-3.236362000	-0.653296000
1	1.398739000	-2.798568000	0.007908000
1	2.041140000	3.587233000	-0.186718000
1	5.804476000	-2.888518000	0.142157000
1	6.640812000	2.029173000	-0.136840000
1	8.137547000	-2.014337000	0.093288000
1	8.554469000	0.434937000	-0.044755000

E= –1653.4109717 a.u.
H= –1653.4109717 + *0.390989* (thermal correction 6-31+G**) a. u.
G= –1653.4109717 + *0.314516* (thermal correction 6-31+G**) a. u.

1b – transition state (*TS*)

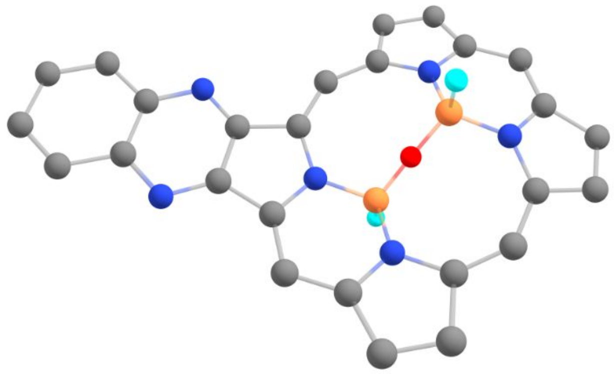

6	2.956829000	-3.051152000	-0.023047000
6	2.307657000	-4.277173000	0.382640000
6	0.964183000	-4.005211000	0.481257000

6	0.747501000	-2.600079000	0.164334000
6	-0.540998000	-1.975257000	0.257841000
6	-1.163509000	-0.706344000	0.296902000
6	-2.623042000	-0.543592000	0.162694000
6	-2.918870000	0.870384000	0.157010000
6	-1.643353000	1.558121000	0.313339000
6	-1.271507000	2.905798000	0.212203000
6	0.092221000	3.283789000	0.119765000
6	0.744778000	4.510579000	-0.274435000
6	2.086579000	4.234342000	-0.378786000
6	2.301933000	2.825100000	-0.081376000
6	3.578343000	2.206083000	-0.187727000
6	4.210608000	0.920643000	-0.206427000
6	5.642808000	0.748107000	-0.027666000
6	5.925932000	-0.598607000	-0.005891000
6	4.676790000	-1.292856000	-0.198953000
6	4.307789000	-2.656576000	-0.091602000
7	1.975417000	-2.117692000	-0.190779000
7	-0.701771000	0.581196000	0.453266000
7	1.059238000	2.342467000	0.267792000
7	3.711759000	-0.346331000	-0.360625000
5	2.258222000	-0.699738000	-0.709042000
8	1.498034000	0.101674000	0.047883000
5	0.769056000	0.932669000	0.793469000
9	2.075844000	-0.650757000	-2.109973000
9	0.926462000	0.893532000	2.196800000
6	-5.888921000	-1.920945000	-0.232881000
6	-7.184026000	-1.472108000	-0.369171000
6	-7.470860000	-0.080412000	-0.381692000
6	-4.820130000	-0.995159000	-0.101132000
6	-5.111990000	0.420227000	-0.112461000
6	-6.459117000	0.845939000	-0.257093000
7	-3.546750000	-1.467973000	0.027354000
7	-4.130086000	1.359718000	0.011568000
1	2.806581000	-5.210116000	0.607361000
1	0.184165000	-4.685294000	0.797285000
1	0.248587000	5.448338000	-0.483894000
1	2.866984000	4.917312000	-0.688285000
1	6.342157000	1.560357000	0.121641000
1	6.887093000	-1.064263000	0.164476000
1	-1.315973000	-2.739147000	0.317296000
1	-2.048937000	3.649762000	0.087288000
1	4.341398000	2.977487000	-0.285495000
1	5.083142000	-3.399945000	0.055758000
1	-5.646817000	-2.978855000	-0.222004000
1	-7.996578000	-2.185377000	-0.469341000
1	-8.498826000	0.251738000	-0.491075000

1	-6.654806000	1.913449000	-0.263557000

E= –1653.3675582 a.u.
H= –1653.3675582 + 0.389293 (thermal correction 6-31+G**) a. u.
G= –1653.3675582 + 0.313061 (thermal correction 6-31+G**) a. u.
f = 183.1798 i

1b– akamptisomer *amplo, amplo* (c_1)

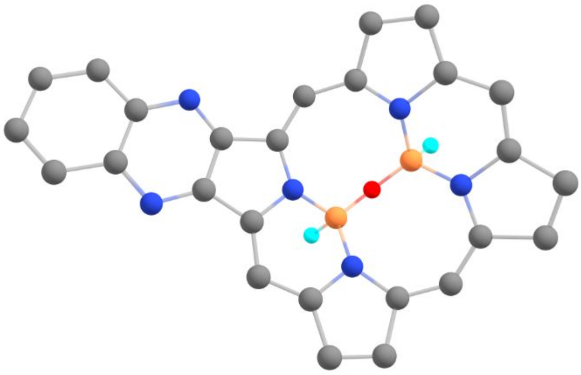

9	3.269969000	-1.491016000	1.846248000
5	0.737009000	1.029048000	1.056632000
5	2.507108000	-0.844359000	0.847562000
8	1.666417000	0.089497000	1.395723000
9	0.228482000	1.688956000	2.196647000
6	2.232562000	4.149676000	-0.449282000
6	2.446951000	2.774413000	-0.055249000
6	3.728472000	2.213715000	-0.077391000
6	4.220403000	0.910538000	-0.265676000
6	5.523674000	0.687792000	-0.849852000
6	5.613465000	-0.638106000	-1.189503000
6	4.385283000	-1.250077000	-0.782619000
6	3.943892000	-2.571523000	-0.930536000
6	2.652807000	-2.935640000	-0.555449000
6	1.956712000	-4.180124000	-0.712831000
6	0.692555000	-3.989182000	-0.223113000
6	0.584973000	-2.610779000	0.210716000
6	-0.668929000	-2.047490000	0.512684000
6	-1.188773000	-0.759856000	0.434862000
6	-2.625700000	-0.546971000	0.220222000
6	-2.806747000	0.831860000	-0.119124000
6	-1.493191000	1.439297000	-0.058677000
6	-1.104449000	2.741777000	-0.325384000
6	0.252149000	3.095965000	-0.293514000
6	0.886243000	4.337300000	-0.622281000
7	1.211123000	2.176002000	0.067972000
7	3.572982000	-0.297458000	-0.203941000
7	1.802609000	-2.015454000	0.032190000
7	-0.585998000	0.468564000	0.319061000
7	-3.609281000	-1.420741000	0.250292000
7	-3.972576000	1.364374000	-0.425638000
6	-4.837866000	-0.900918000	-0.027142000
6	-5.019485000	0.494922000	-0.367039000
6	-5.972835000	-1.755380000	0.008154000
6	-6.328069000	0.969336000	-0.655038000
6	-7.225228000	-1.258869000	-0.274656000
6	-7.403960000	0.112009000	-0.608126000
1	3.027593000	4.856634000	-0.646797000
1	6.245998000	1.469239000	-1.045708000

1	6.427572000	-1.142860000	-1.691700000
1	2.368198000	-5.072877000	-1.163982000
1	-0.128808000	-4.693296000	-0.217725000
1	0.375552000	5.227724000	-0.962576000
1	4.506202000	2.965969000	-0.181999000
1	4.597491000	-3.305518000	-1.387581000
1	-1.458230000	-2.790035000	0.599205000
1	-1.856467000	3.468800000	-0.606715000
1	-5.813842000	-2.797536000	0.266039000
1	-6.441399000	2.018994000	-0.906487000
1	-8.088259000	-1.917088000	-0.244042000
1	-8.401059000	0.481848000	-0.827227000

E= –1653.3989313 a.u.
H= –1653.3989313 + 0.390523 (thermal correction 6-31+G**) a. u.
G= –1653.3989313 + 0.313227 (thermal correction 6-31+G**) a. u.

1c – akamptisomer *amplo,parvo* (***t***$_1$)

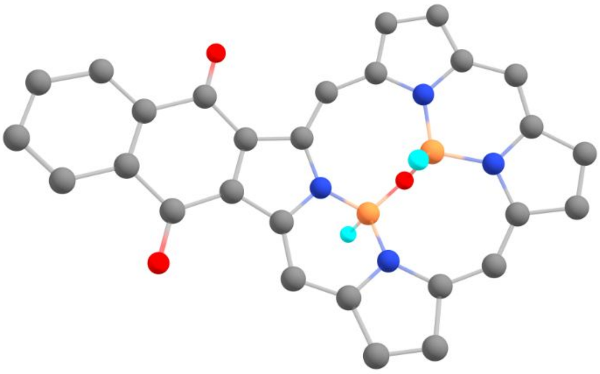

9	1.698703000	-0.064142000	-1.226881000
5	1.061923000	0.957557000	1.174153000
5	2.383288000	-0.570719000	-0.088366000
8	2.027038000	-0.019167000	1.157552000
9	0.756678000	1.449730000	2.451502000
6	2.421279000	4.118237000	-0.467721000
6	2.655605000	2.735322000	-0.075477000
6	3.946278000	2.165775000	-0.206480000
6	4.500126000	0.879919000	-0.313108000
6	5.916997000	0.632266000	-0.530225000
6	6.105287000	-0.718373000	-0.634793000
6	4.808422000	-1.337951000	-0.487071000
6	4.425587000	-2.683930000	-0.459896000
6	3.089614000	-3.041606000	-0.210783000
6	2.464358000	-4.332198000	-0.041937000
6	1.146613000	-4.101755000	0.246868000
6	0.928127000	-2.664808000	0.264177000
6	-0.328289000	-2.086136000	0.483058000
6	-0.892386000	-0.782401000	0.477838000
6	-2.313211000	-0.573021000	0.260601000
6	-2.526542000	0.794469000	0.129098000
6	-1.258785000	1.433386000	0.288517000
6	-0.886929000	2.778827000	0.102485000
6	0.458466000	3.137354000	0.032717000
6	1.077060000	4.358848000	-0.414235000
7	1.443605000	2.206782000	0.273978000
7	3.887220000	-0.343782000	-0.310579000
7	2.135942000	-2.085060000	-0.033199000
7	-0.320882000	0.466360000	0.543574000

1	3.197146000	4.791353000	-0.807827000
1	6.669420000	1.407639000	-0.587357000
1	7.035150000	-1.248746000	-0.788015000
1	2.969212000	-5.284892000	-0.124590000
1	0.373805000	-4.834569000	0.436807000
1	0.548948000	5.260422000	-0.692736000
1	4.699809000	2.934404000	-0.362006000
1	5.171618000	-3.457672000	-0.595622000
1	-1.105626000	-2.837048000	0.579866000
1	-1.665526000	3.502978000	-0.100478000
6	-3.831126000	1.398262000	-0.166066000
8	-3.980555000	2.611076000	-0.312323000
6	-3.413328000	-1.542966000	0.124173000
8	-3.260937000	-2.761180000	0.220013000
6	-7.339734000	0.061134000	-0.619335000
6	-6.256695000	0.934006000	-0.517007000
6	-7.143293000	-1.317613000	-0.478876000
1	-6.383653000	2.006461000	-0.622134000
1	-7.985939000	-1.997971000	-0.558186000
6	-4.971554000	0.437062000	-0.273052000
6	-5.865233000	-1.821922000	-0.237110000
1	-5.692134000	-2.887188000	-0.127123000
6	-4.771298000	-0.954954000	-0.131653000
1	-8.334767000	0.453016000	-0.807739000

E= –1770.6334386 a.u.
H= –1770.6334386 + *0.402895* (thermal correction 6-31+G**) a. u.
G= –1770.6334386 + *0.321485* (thermal correction 6-31+G**) a. u.

1c – akamptisomer *parvo,amplo* (***t*$_2$**)

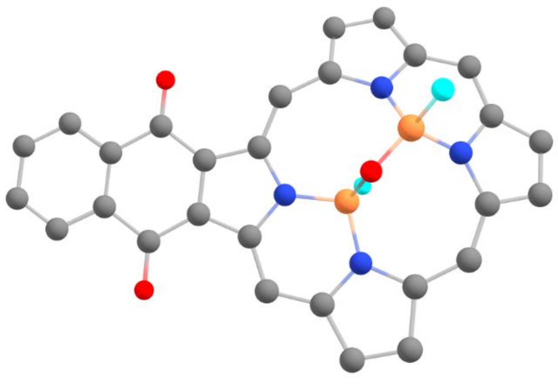

9	-3.144191000	-1.200271000	2.212290000
5	-1.104563000	0.752351000	-0.066667000
5	-2.648612000	-0.726562000	0.987226000
8	-1.654690000	0.217281000	1.110402000
9	-1.613341000	0.258942000	-1.297351000
6	-2.274923000	4.324553000	0.033982000
6	-2.532275000	2.893716000	0.035694000
6	-3.830505000	2.343929000	0.054984000
6	-4.414651000	1.061233000	0.001001000
6	-5.802937000	0.899339000	-0.413780000
6	-6.053181000	-0.434882000	-0.564878000
6	-4.833800000	-1.124338000	-0.222009000
6	-4.486253000	-2.473037000	-0.353498000
6	-3.147182000	-2.881662000	-0.234163000
6	-2.514318000	-4.124672000	-0.596248000
6	-1.169019000	-3.934450000	-0.447626000

6	-0.945022000	-2.559970000	-0.019779000
6	0.361365000	-2.042040000	0.022978000
6	0.979894000	-0.769807000	0.016014000
6	2.412861000	-0.562186000	0.005633000
6	2.646526000	0.807851000	-0.062118000
6	1.370687000	1.455989000	-0.099427000
6	1.024935000	2.820302000	-0.132571000
6	-0.313612000	3.209608000	-0.093047000
6	-0.922714000	4.517134000	-0.041891000
7	-1.317808000	2.276662000	-0.054412000
7	-3.897254000	-0.197807000	0.161264000
7	-2.185235000	-1.991084000	0.153196000
7	0.415488000	0.478963000	-0.059803000
1	-3.045253000	5.082081000	0.091444000
1	-6.471209000	1.722630000	-0.629183000
1	-6.961273000	-0.906223000	-0.915250000
1	-3.026566000	-5.007084000	-0.954567000
1	-0.373995000	-4.633420000	-0.671001000
1	-0.383745000	5.454435000	-0.053634000
1	-4.587128000	3.123523000	0.005399000
1	-5.235977000	-3.189425000	-0.668534000
1	1.108191000	-2.826867000	-0.041578000
1	1.820049000	3.553891000	-0.160859000
6	3.515396000	-1.536627000	0.046107000
8	3.337297000	-2.753820000	0.090948000
6	3.983895000	1.409776000	-0.085288000
8	4.159468000	2.626771000	-0.142312000
6	5.991797000	-1.826709000	0.064433000
6	4.898390000	-0.953893000	0.023126000
6	7.293815000	-1.326106000	0.045402000
1	8.135994000	-2.010831000	0.078268000
6	5.122884000	0.440821000	-0.038353000
6	7.514740000	0.054711000	-0.015442000
1	8.528508000	0.443551000	-0.029902000
6	6.432395000	0.933649000	-0.057328000
1	6.578536000	2.007737000	-0.104949000
1	5.799466000	-2.893360000	0.111630000

E= –1770.6337346 a.u.
H= –1770.6337346 + *0.402951* (thermal correction 6-31+G**) a. u.
G= –1770.6337346 + *0.321679* (thermal correction 6-31+G**) a. u.

1c – transition state (*TS*)

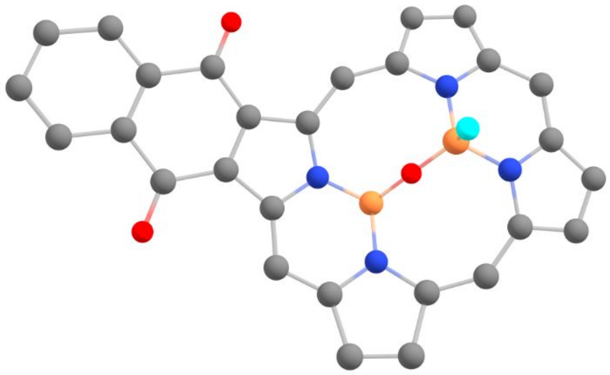

9	2.271156000	-0.627487000	-2.120213000
5	1.030924000	0.927145000	0.831704000
5	2.494809000	-0.675964000	-0.726297000
8	1.758257000	0.120440000	0.056546000

9	1.241357000	0.887005000	2.227529000
6	2.277198000	4.249674000	-0.376176000
6	2.511143000	2.840496000	-0.074447000
6	3.803411000	2.233382000	-0.197167000
6	4.440313000	0.960499000	-0.252132000
6	5.885382000	0.798358000	-0.102295000
6	6.181622000	-0.539747000	-0.100170000
6	4.930247000	-1.250137000	-0.275617000
6	4.581124000	-2.611840000	-0.162863000
6	3.226845000	-3.014065000	-0.051418000
6	2.604669000	-4.247265000	0.384848000
6	1.264449000	-3.991014000	0.521749000
6	1.018631000	-2.586810000	0.203702000
6	-0.262016000	-1.993200000	0.354898000
6	-0.920179000	-0.714620000	0.374098000
6	-2.356785000	-0.547129000	0.220961000
6	-2.639876000	0.819802000	0.205600000
6	-1.397823000	1.515495000	0.378047000
6	-1.041653000	2.885267000	0.265071000
6	0.303507000	3.277959000	0.165043000
6	0.937360000	4.512577000	-0.245650000
7	1.293815000	2.346818000	0.302340000
7	3.955399000	-0.311448000	-0.418428000
7	2.240023000	-2.095779000	-0.199556000
7	-0.436780000	0.563311000	0.524573000
1	3.045061000	4.937319000	-0.705696000
1	6.577572000	1.617180000	0.044401000
1	7.149869000	-0.998207000	0.048082000
1	3.122555000	-5.172349000	0.598344000
1	0.500472000	-4.677132000	0.862972000
1	0.425910000	5.443040000	-0.450853000
1	4.557429000	3.015335000	-0.277804000
1	5.363909000	-3.350019000	-0.032747000
1	-1.011538000	-2.769033000	0.487111000
1	-1.838326000	3.607856000	0.140313000
6	-3.980008000	1.376789000	-0.001449000
8	-4.187642000	2.589432000	-0.045197000
6	-3.418613000	-1.554238000	0.059775000
8	-3.204143000	-2.766401000	0.077838000
6	-7.434735000	-0.092035000	-0.462203000
6	-6.390641000	0.822501000	-0.323966000
6	-7.170864000	-1.466221000	-0.428573000
1	-6.569817000	1.892414000	-0.346952000
1	-7.983187000	-2.178795000	-0.536199000
6	-5.077424000	0.371820000	-0.150420000
6	-5.864348000	-1.924673000	-0.257627000
1	-5.639235000	-2.985603000	-0.230901000

6	-4.809110000	-1.015894000	-0.117150000
1	-8.451879000	0.263946000	-0.595770000

E= –1770.5919936 a.u.
H= –1770.5919936 + *0.401232* (thermal correction 6-31+G**) a. u.
G= –1770.5919936 + *0.320142* (thermal correction 6-31+G**) a. u.
f = 185.1842 i

1c– akamptisomer *amplo, amplo* (c_1)

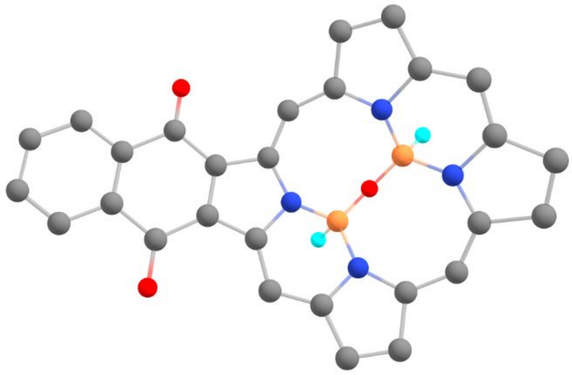

9	3.566061000	-1.532682000	1.771250000
5	0.998790000	0.990700000	1.113085000
5	2.772739000	-0.853450000	0.819643000
8	1.948367000	0.058367000	1.424246000
9	0.509755000	1.634761000	2.269896000
6	2.456230000	4.163256000	-0.347153000
6	2.682398000	2.775923000	0.014184000
6	3.974991000	2.226857000	-0.031779000
6	4.457431000	0.935623000	-0.276341000
6	5.756737000	0.727258000	-0.891045000
6	5.832711000	-0.579921000	-1.282140000
6	4.604098000	-1.206131000	-0.875037000
6	4.163522000	-2.516962000	-1.054511000
6	2.876373000	-2.893181000	-0.652067000
6	2.188891000	-4.144807000	-0.807678000
6	0.947783000	-3.982641000	-0.256084000
6	0.837759000	-2.609683000	0.201369000
6	-0.407047000	-2.075218000	0.539984000
6	-0.947950000	-0.775265000	0.479252000
6	-2.360373000	-0.550105000	0.255385000
6	-2.527066000	0.795531000	-0.048703000
6	-1.239628000	1.404323000	0.023681000
6	-0.856737000	2.730944000	-0.223443000
6	0.485691000	3.089491000	-0.191043000
6	1.109968000	4.346292000	-0.496852000
7	1.463066000	2.166806000	0.140940000
7	3.807532000	-0.270913000	-0.245602000
7	2.047777000	-1.995368000	-0.019080000
7	-0.316627000	0.441195000	0.374041000
6	-4.833288000	-0.895146000	-0.054304000
6	-4.991274000	0.476687000	-0.357798000
6	-5.957154000	-1.729339000	-0.031248000
6	-6.265672000	0.985938000	-0.631306000
6	-7.223997000	-1.212278000	-0.302895000
6	-7.378922000	0.146083000	-0.603152000
1	3.245759000	4.878739000	-0.535369000
1	6.479208000	1.513116000	-1.067694000

1	6.634703000	-1.069798000	-1.817460000
1	2.592703000	-5.022545000	-1.293645000
1	0.135380000	-4.696632000	-0.227582000
1	0.587644000	5.240070000	-0.809614000
1	4.747097000	2.986216000	-0.121889000
1	4.805384000	-3.238530000	-1.545958000
1	-1.178921000	-2.828457000	0.652791000
1	-1.621211000	3.450826000	-0.485400000
1	-5.816272000	-2.779209000	0.202887000
1	-6.360540000	2.041768000	-0.862622000
1	-8.090312000	-1.866631000	-0.280419000
1	-8.365354000	0.547957000	-0.814272000
6	-3.818067000	1.403533000	-0.395717000
8	-3.931420000	2.591793000	-0.694480000
6	-3.489915000	-1.495582000	0.241973000
8	-3.368502000	-2.700507000	0.463610000

E= –1770.6226008 a.u.
H= –1770.6226008 + 0.402502 (thermal correction 6-31+G**) a. u.
G= –1770.6226008 + 0.320368 (thermal correction 6-31+G**) a. u.

1d – akamptisomer *amplo,parvo* (***t***$_1$)

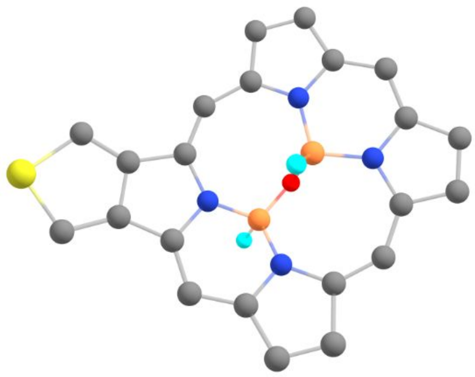

9	0.828725000	-0.058789000	-1.258195000
5	-0.083639000	0.901320000	1.082649000
5	1.479367000	-0.489896000	-0.068489000
8	0.967414000	0.020637000	1.140214000
9	-0.533787000	1.340445000	2.338784000
6	1.065585000	4.190573000	-0.442690000
6	1.415430000	2.839136000	-0.044079000
6	2.750574000	2.401974000	-0.102852000
6	3.448426000	1.175176000	-0.155620000
6	4.885444000	1.079278000	-0.275628000
6	5.222065000	-0.250995000	-0.352785000
6	3.996273000	-0.996370000	-0.287000000
6	3.750560000	-2.382084000	-0.278198000
6	2.455022000	-2.878716000	-0.126690000
6	1.950607000	-4.222981000	-0.007283000
6	0.595824000	-4.127189000	0.178332000
6	0.235995000	-2.721120000	0.179813000
6	-1.095322000	-2.259513000	0.295750000
6	-1.782706000	-1.037289000	0.266575000
6	-3.227902000	-0.989762000	0.004520000
6	-3.604206000	0.389086000	-0.131879000
6	-2.389979000	1.158633000	0.066385000
6	-2.135207000	2.515996000	-0.089170000
6	-0.815824000	3.009616000	-0.085845000
6	-0.299717000	4.289367000	-0.486722000

7	0.228214000	2.184027000	0.217159000
7	2.964787000	-0.105239000	-0.179650000
7	1.387770000	-2.022341000	-0.018863000
7	-1.374256000	0.274412000	0.360400000
1	1.787784000	4.945421000	-0.724910000
1	5.555980000	1.928256000	-0.292262000
1	6.210957000	-0.680283000	-0.438110000
1	2.551660000	-5.121088000	-0.049477000
1	-0.108332000	-4.937903000	0.310555000
1	-0.895733000	5.136183000	-0.798400000
1	3.431105000	3.241485000	-0.227918000
1	4.583104000	-3.071144000	-0.359592000
1	-1.791310000	-3.095004000	0.317207000
1	-2.951780000	3.185791000	-0.331151000
6	-4.273367000	-1.855623000	-0.188895000
16	-5.731484000	-0.970502000	-0.519983000
6	-4.931846000	0.569028000	-0.422623000
1	-4.293008000	-2.936309000	-0.158535000
1	-5.489068000	1.482181000	-0.579276000

E= –1788.3714229 a.u.
H= –1788.3714229 + *0.331779* (thermal correction 6-31+G**) a. u.
G= –1788.3714229 + *0.261191* (thermal correction 6-31+G**) a. u.

1d – akamptisomer *parvo,amplo* (***t*****₂**)

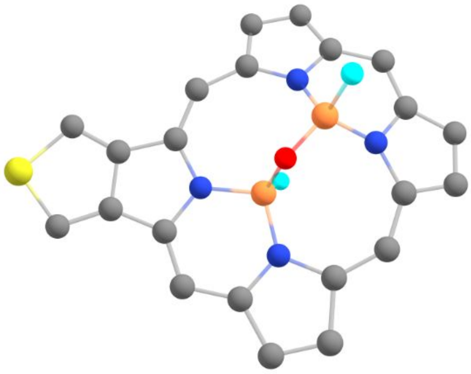

9	-2.232328000	-1.042095000	2.216423000
5	-0.029328000	0.700979000	-0.085302000
5	-1.707343000	-0.628609000	0.979584000
8	-0.624391000	0.216053000	1.090371000
9	-0.568830000	0.239556000	-1.315024000
6	-0.886996000	4.360342000	0.007933000
6	-1.274376000	2.964952000	0.018470000
6	-2.608336000	2.536597000	0.056305000
6	-3.314711000	1.309723000	0.008111000
6	-4.705808000	1.276446000	-0.393570000
6	-5.079820000	-0.035986000	-0.539818000
6	-3.932008000	-0.827327000	-0.205944000
6	-3.708771000	-2.210918000	-0.333460000
6	-2.424784000	-2.743993000	-0.215667000
6	-1.906734000	-4.037608000	-0.570871000
6	-0.545976000	-3.963605000	-0.439706000
6	-0.200575000	-2.615741000	-0.017598000
6	1.158568000	-2.207140000	0.046110000
6	1.886763000	-1.016853000	0.022572000
6	3.351518000	-0.961877000	0.016368000
6	3.737313000	0.420257000	-0.078055000
6	2.504685000	1.189689000	-0.123829000

6	2.273915000	2.559386000	-0.172827000
6	0.962716000	3.073184000	-0.129728000
6	0.479858000	4.428158000	-0.080400000
7	-0.109460000	2.236779000	-0.080478000
7	-2.908110000	0.008059000	0.160835000
7	-1.371191000	-1.938969000	0.158753000
7	1.468103000	0.290726000	-0.070748000
1	-1.583319000	5.186402000	0.067431000
1	-5.299729000	2.155916000	-0.605041000
1	-6.030846000	-0.421720000	-0.881041000
1	-2.496228000	-4.874822000	-0.919220000
1	0.179397000	-4.732804000	-0.669762000
1	1.100838000	5.313188000	-0.100930000
1	-3.292716000	3.381650000	0.024215000
1	-4.527087000	-2.853173000	-0.638847000
1	1.821374000	-3.068552000	0.004345000
1	3.113930000	3.242188000	-0.208995000
6	5.095030000	0.609570000	-0.088938000
16	5.909029000	-0.921463000	0.016157000
6	4.421184000	-1.817073000	0.077275000
1	5.665983000	1.525627000	-0.150371000
1	4.441526000	-2.895167000	0.157823000

E= –1788.3718767 a.u.
H= –1788.3718767 + 0.331779 (thermal correction 6-31+G**) a. u.
G= –1788.3718767 + 0.261195 (thermal correction 6-31+G**) a. u.

1d – transition state (*TS*)

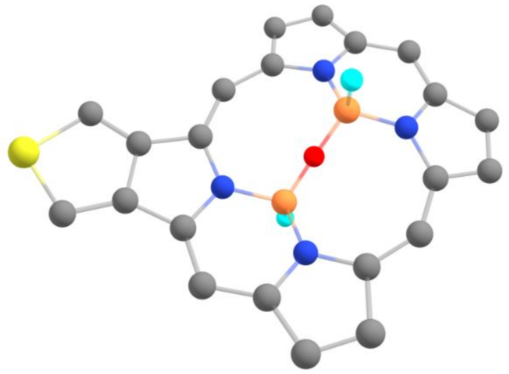

9	1.492088000	-0.521560000	-2.113229000
5	-0.105041000	0.841345000	0.756833000
5	1.632522000	-0.566174000	-0.706366000
8	0.749609000	0.117857000	0.031809000
9	0.011016000	0.801692000	2.166058000
6	0.831123000	4.304941000	-0.312243000
6	1.213498000	2.927941000	-0.035054000
6	2.557895000	2.477680000	-0.110178000
6	3.349122000	1.281558000	-0.121907000
6	4.782642000	1.288336000	0.104561000
6	5.232968000	-0.013734000	0.122310000
6	4.089863000	-0.854994000	-0.120750000
6	3.892535000	-2.257397000	-0.035900000
6	2.603536000	-2.819546000	-0.014759000
6	2.101095000	-4.118491000	0.369974000
6	0.732237000	-4.015481000	0.433474000
6	0.350127000	-2.646598000	0.110536000
6	-1.012452000	-2.191271000	0.165949000

6	-1.804803000	-1.018199000	0.184905000
6	-3.267135000	-1.035530000	0.009255000
6	-2.565315000	1.170880000	0.210964000
6	-2.352376000	2.554134000	0.145900000
6	-1.041487000	3.099695000	0.098391000
6	-0.537824000	4.407799000	-0.249398000
7	0.028251000	2.284660000	0.260335000
7	3.018104000	-0.035827000	-0.305834000
7	1.515678000	-2.015372000	-0.211368000
7	-1.508306000	0.317216000	0.361847000
1	1.528366000	5.088137000	-0.580250000
1	5.368987000	2.179632000	0.286198000
1	6.238985000	-0.357191000	0.321523000
1	2.705723000	-4.983222000	0.607836000
1	0.036408000	-4.789531000	0.729777000
1	-1.141190000	5.281061000	-0.456703000
1	3.220203000	3.340261000	-0.175655000
1	4.750961000	-2.897792000	0.133345000
1	-1.668104000	-3.060148000	0.213504000
1	-3.202227000	3.212835000	0.012533000
6	-3.739713000	0.329870000	0.016030000
6	-5.090939000	0.439330000	-0.181164000
16	-5.798313000	-1.137052000	-0.367900000
6	-4.269751000	-1.946552000	-0.196618000
1	-5.712869000	1.322631000	-0.225801000
1	-4.219622000	-3.024689000	-0.262691000

E= –1788.3297964 a.u.
H= –1788.3297964 + *0.330206* (thermal correction 6-31+G**) a. u.
G= –1788.3297964 + *0.260011* (thermal correction 6-31+G**) a. u.
f = 182.1491 i

1d– akamptisomer *amplo, amplo* (c_1)

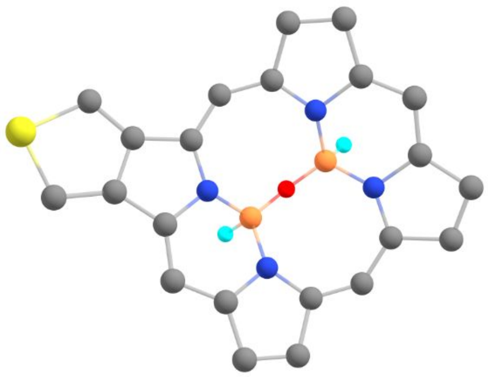

9	2.586538000	-1.298140000	1.911581000
5	-0.143192000	0.961996000	0.996031000
5	1.815047000	-0.722247000	0.875491000
8	0.858436000	0.118445000	1.382688000
9	-0.763408000	1.568493000	2.111987000
6	1.115887000	4.221455000	-0.427446000
6	1.446598000	2.872896000	-0.024280000
6	2.775166000	2.443403000	0.017000000
6	3.403891000	1.195509000	-0.147786000
6	4.746632000	1.105033000	-0.669138000
6	4.981590000	-0.205466000	-1.006291000
6	3.802156000	-0.935085000	-0.660445000
6	3.497013000	-2.293839000	-0.831879000
6	2.233036000	-2.782457000	-0.520034000

6	1.669374000	-4.088537000	-0.702312000
6	0.372023000	-4.022202000	-0.269935000
6	0.110467000	-2.660519000	0.153412000
6	-1.205905000	-2.225970000	0.409878000
6	-1.861407000	-1.002737000	0.303261000
6	-3.302381000	-0.926272000	0.044987000
6	-3.602587000	0.429389000	-0.312890000
6	-2.355294000	1.160152000	-0.215078000
6	-2.069388000	2.490692000	-0.470788000
6	-0.753475000	2.973078000	-0.378135000
6	-0.231597000	4.273745000	-0.672811000
7	0.269381000	2.152202000	0.031155000
7	2.874409000	-0.069748000	-0.119804000
7	1.268296000	-1.949960000	0.025727000
7	-1.369860000	0.278765000	0.198355000
1	1.844963000	5.006058000	-0.581884000
1	5.398015000	1.954025000	-0.829587000
1	5.864036000	-0.625203000	-1.469826000
1	2.185728000	-4.937131000	-1.130368000
1	-0.374084000	-4.805308000	-0.297774000
1	-0.810225000	5.111039000	-1.038502000
1	3.478725000	3.269738000	-0.046267000
1	4.240629000	-2.958684000	-1.256142000
1	-1.901396000	-3.058287000	0.476541000
1	-2.861827000	3.159119000	-0.785247000
6	-4.393954000	-1.755293000	-0.009586000
16	-5.802105000	-0.855938000	-0.483793000
6	-4.920952000	0.632394000	-0.634178000
1	-4.473517000	-2.812331000	0.203715000
1	-5.429251000	1.537469000	-0.936398000

E= –1788.3597342 a.u.
H= –1788.3597342 + 0.331375 (thermal correction 6-31+G**) a. u.
G= –1788.3597342 + 0.260124 (thermal correction 6-31+G**) a. u.

STRUCTURAL VARIATION 2

2a – akamptisomer *amplo,parvo* ($\boldsymbol{t}_1$)

9	-0.537389000	-0.010996000	-1.272551000
5	0.616993000	-0.719548000	1.056656000
5	-1.210096000	0.324337000	-0.064543000
8	-0.588140000	-0.062250000	1.137558000
9	1.164122000	-1.067404000	2.306523000
6	0.059109000	-4.182897000	-0.442613000
6	-0.522900000	-2.921975000	-0.018288000
6	-1.923595000	-2.743996000	-0.023862000

6	-2.837114000	-1.672447000	-0.063335000
6	-4.273745000	-1.844562000	-0.133060000
6	-4.852113000	-0.602668000	-0.214660000
6	-3.781706000	0.359918000	-0.202787000
6	-3.792713000	1.765672000	-0.227299000
6	-2.601131000	2.492079000	-0.132721000
6	-2.349802000	3.910513000	-0.061550000
6	-0.996012000	4.070627000	0.072318000
6	-0.380298000	2.754907000	0.092663000
6	1.011428000	2.552110000	0.178277000
6	1.913163000	1.468734000	0.156158000
6	3.328861000	1.674415000	-0.140842000
6	3.919271000	0.435605000	-0.269578000
6	2.880193000	-0.546524000	-0.034599000
6	2.892961000	-1.929958000	-0.193282000
6	1.695954000	-2.669361000	-0.139745000
6	1.416693000	-4.022223000	-0.534646000
7	0.523158000	-2.057854000	0.208501000
7	-2.603199000	-0.324050000	-0.120055000
7	-1.394530000	1.850142000	-0.047422000
7	1.719381000	0.114924000	0.285008000
1	-0.517707000	-5.059447000	-0.707075000
1	-4.775932000	-2.802825000	-0.115043000
1	-5.905653000	-0.364994000	-0.270418000
1	-3.107979000	4.680867000	-0.099159000
1	-0.450328000	5.000899000	0.160203000
1	2.149367000	-4.740544000	-0.876570000
1	-2.437698000	-3.698478000	-0.115257000
1	-4.737781000	2.291959000	-0.291358000
1	1.540220000	3.502496000	0.154640000
1	3.808707000	-2.433408000	-0.481953000
7	4.032564000	2.885116000	-0.364449000
7	5.214607000	0.176674000	-0.659596000
1	3.860789000	3.589215000	0.345677000
1	3.864387000	3.290088000	-1.281493000
1	5.819731000	0.988241000	-0.605337000
1	5.637017000	-0.666877000	-0.296851000

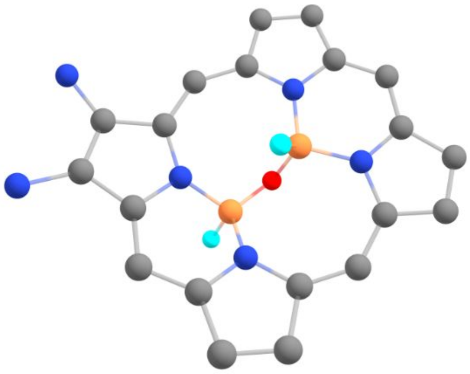

E= –1424.6701822 a.u.
H= –1424.6701822 + *0.352744* (thermal correction 6-31+G**) a. u.
G= –1424.6701822 + *0.281517* (thermal correction 6-31+G**) a. u.

2a – akamptisomer *parvo,amplo* (***t*₂**)

9	-2.021045000	-0.782954000	2.227709000
5	0.429367000	0.571111000	-0.088400000
5	-1.436776000	-0.449865000	0.991702000
8	-0.230644000	0.202167000	1.100494000

9	-0.224088000	0.238465000	-1.308375000
6	0.254523000	4.322030000	0.006815000
6	-0.377435000	3.017886000	0.026487000
6	-1.770168000	2.828742000	0.073461000
6	-2.678959000	1.745516000	0.031588000
6	-4.057979000	1.958534000	-0.370653000
6	-4.656662000	0.735376000	-0.521748000
6	-3.664863000	-0.249988000	-0.189429000
6	-3.683086000	-1.646949000	-0.331863000
6	-2.504473000	-2.394256000	-0.228481000
6	-2.221532000	-3.751299000	-0.614405000
6	-0.869026000	-3.918978000	-0.490105000
6	-0.290655000	-2.661843000	-0.042788000
6	1.114826000	-2.500525000	0.015671000
6	2.031438000	-1.440375000	0.015274000
6	3.475437000	-1.633274000	0.036789000
6	4.071563000	-0.393404000	-0.076667000
6	3.003563000	0.579756000	-0.158787000
6	3.033429000	1.975157000	-0.206037000
6	1.841882000	2.721019000	-0.144841000
6	1.610667000	4.141696000	-0.095572000
7	0.632061000	2.095617000	-0.078880000
7	-2.513537000	0.391739000	0.186099000
7	-1.330732000	-1.793179000	0.156379000
7	1.813290000	-0.092078000	-0.098285000
1	-0.280012000	5.260692000	0.070042000
1	-4.486149000	2.929760000	-0.581364000
1	-5.659582000	0.524436000	-0.867268000
1	-2.949665000	-4.464328000	-0.976662000
1	-0.289028000	-4.797153000	-0.741724000
1	2.379359000	4.901831000	-0.126826000
1	-2.295787000	3.780584000	0.037335000
1	-4.598015000	-2.136849000	-0.645042000
1	1.625067000	-3.457336000	-0.072744000
1	3.978388000	2.503529000	-0.259723000
7	4.101479000	-2.883443000	0.095450000
1	5.089220000	-2.872137000	-0.123026000
1	3.908878000	-3.433367000	0.924375000
7	5.441957000	-0.127730000	-0.130855000
1	5.684115000	0.853155000	-0.080974000
1	5.995046000	-0.661401000	0.531527000

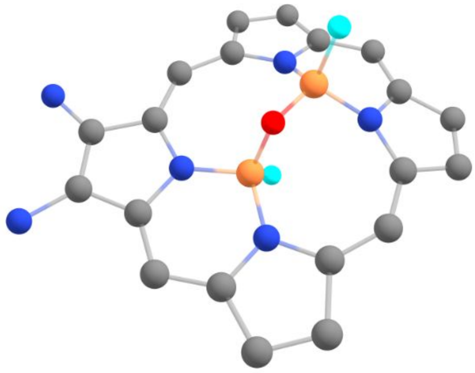

E= –1424.668953 a.u.

H= –1424.668953 + 0.352582 (thermal correction 6-31+G**) a. u.

G= –1424.668953 + 0.280425 (thermal correction 6-31+G**) a. u.

2a – transition state (*TS*)

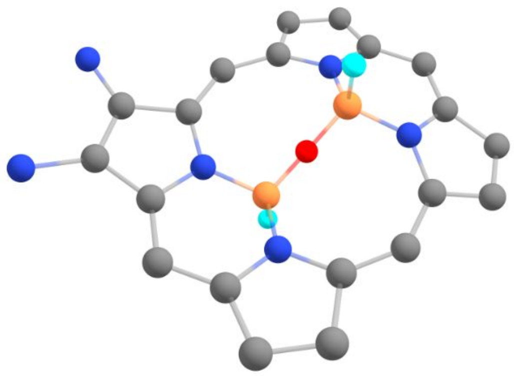

9	1.239578000	-0.341914000	-2.122080000
5	-0.612529000	0.637988000	0.759776000
5	1.384679000	-0.352843000	-0.713770000
8	0.393103000	0.151455000	0.025817000
9	-0.476409000	0.610147000	2.169701000
6	-0.443438000	4.229059000	-0.292883000
6	0.223745000	2.963596000	-0.008773000
6	1.638117000	2.808327000	-0.079213000
6	2.664736000	1.807929000	-0.100861000
6	4.064542000	2.117414000	0.149182000
6	4.781260000	0.943420000	0.164369000
6	3.846181000	-0.122623000	-0.105510000
6	3.943533000	-1.531083000	-0.018466000
6	2.794853000	-2.351815000	-0.006650000
6	2.577149000	-3.725224000	0.382062000
6	1.216248000	-3.916868000	0.429133000
6	0.555322000	-2.664667000	0.092085000
6	-0.871596000	-2.509247000	0.129334000
6	-1.885275000	-1.516901000	0.130240000
6	-3.304862000	-1.819881000	-0.124851000
6	-4.022006000	-0.635579000	-0.096730000
6	-3.074670000	0.420478000	0.172569000
6	-3.172242000	1.827528000	0.123243000
6	-2.017135000	2.646574000	0.104197000
6	-1.802113000	4.035279000	-0.241026000
7	-0.793463000	2.086603000	0.283752000
7	2.629843000	0.453936000	-0.310541000
7	1.566708000	-1.797594000	-0.219993000
7	-1.853044000	-0.159506000	0.339477000
1	0.071503000	5.143011000	-0.559263000
1	4.443409000	3.110826000	0.351263000
1	5.833633000	0.819965000	0.381453000
1	3.349424000	-4.439776000	0.632831000
1	0.698533000	-4.820499000	0.723808000
1	-2.576615000	4.758604000	-0.457550000
1	2.102709000	3.792759000	-0.127351000
1	4.912762000	-1.980666000	0.166036000
1	-1.325767000	-3.497370000	0.200115000
1	-4.144687000	2.285094000	-0.020940000
7	-3.836558000	-3.067140000	-0.368182000
7	-5.409049000	-0.556061000	-0.396510000
1	-5.982812000	-0.383818000	0.424531000
1	-5.620266000	0.152008000	-1.093355000
1	-3.252912000	-3.719371000	-0.873531000
1	-4.788165000	-3.028204000	-0.717929000

E= –1424.6247747 a.u.
H= –1424.6247747 + *0.351092* (thermal correction 6-31+G**) a. u.
G= –1424.6247747 + *0.279778* (thermal correction 6-31+G**) a. u.
f = 185.1677 i

2a– akamptisomer *amplo, amplo* (c_1)

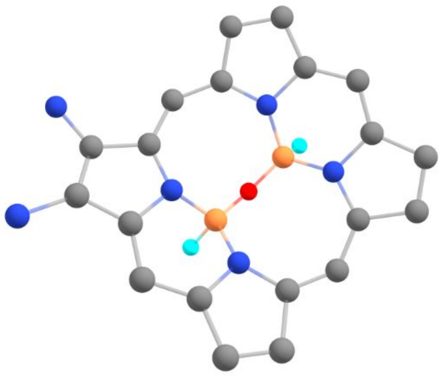

9	2.349276000	-1.040420000	1.933503000
5	-0.674703000	0.776637000	0.947215000
5	1.525504000	-0.554584000	0.889870000
8	0.441387000	0.116936000	1.390969000
9	-1.441832000	1.258842000	2.039065000
6	0.083213000	4.235233000	-0.391857000
6	0.615223000	2.956775000	0.029698000
6	1.996386000	2.752523000	0.142114000
6	2.820279000	1.626825000	-0.017802000
6	4.184719000	1.766415000	-0.477743000
6	4.636020000	0.524978000	-0.844483000
6	3.571934000	-0.395391000	-0.575366000
6	3.493266000	-1.773705000	-0.802350000
6	2.308934000	-2.467711000	-0.556841000
6	1.972608000	-3.837989000	-0.813788000
6	0.669056000	-4.002243000	-0.428581000
6	0.175735000	-2.722829000	0.038532000
6	-1.197023000	-2.522113000	0.267714000
6	-2.023214000	-1.402243000	0.186059000
6	-3.452694000	-1.532428000	-0.062420000
6	-3.921350000	-0.289982000	-0.440613000
6	-2.804612000	0.614627000	-0.364948000
6	-2.746572000	1.982725000	-0.612415000
6	-1.544818000	2.683912000	-0.447464000
6	-1.237580000	4.058995000	-0.711455000
7	-0.417275000	2.049326000	0.023023000
7	2.498725000	0.293027000	-0.049036000
7	1.209283000	-1.826234000	-0.017334000
7	-1.683715000	-0.075109000	0.059412000
1	0.675170000	5.134031000	-0.504637000
1	4.698881000	2.712850000	-0.581967000
1	5.591363000	0.265959000	-1.280512000
1	2.634277000	-4.570621000	-1.255868000
1	0.060640000	-4.892588000	-0.517147000
1	-1.928536000	4.788949000	-1.111128000
1	2.557409000	3.683507000	0.128278000
1	4.345779000	-2.297978000	-1.218440000
1	-1.756254000	-3.453394000	0.272392000
1	-3.620524000	2.518734000	-0.964845000

7	-4.145874000	-2.745609000	-0.079508000
7	-5.205347000	0.019255000	-0.887306000
1	-5.416783000	1.008349000	-0.913372000
1	-5.942781000	-0.485141000	-0.407744000
1	-4.201249000	-3.235625000	0.805211000
1	-5.037130000	-2.729435000	-0.557212000

E= –1424.6603418 a.u.
H= –1424.6603418 + 0.352254 (thermal correction 6-31+G**) a. u.
G= –1424.6603418 + 0.279913 (thermal correction 6-31+G**) a. u.

2b – akamptisomer *amplo,parvo* ($\boldsymbol{t_1}$)

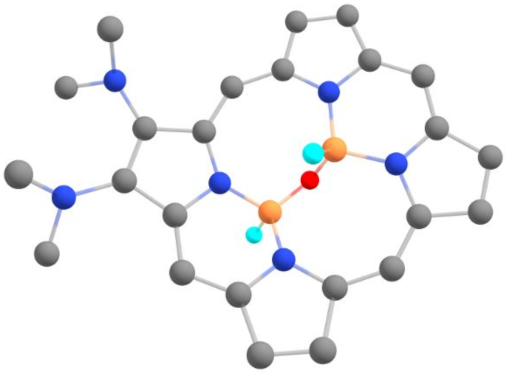

9	-1.125094000	0.033342000	-1.215371000
5	-0.293669000	-0.944671000	1.157263000
5	-1.834226000	0.427761000	-0.045354000
8	-1.374671000	-0.096327000	1.177674000
9	0.085868000	-1.418485000	2.427468000
6	-1.289026000	-4.224585000	-0.509727000
6	-1.691333000	-2.893441000	-0.091798000
6	-3.039448000	-2.484480000	-0.197338000
6	-3.757825000	-1.275109000	-0.256539000
6	-5.194653000	-1.204406000	-0.431198000
6	-5.556575000	0.117625000	-0.486985000
6	-4.348411000	0.889990000	-0.353444000
6	-4.135839000	2.277509000	-0.291475000
6	-2.853471000	2.794269000	-0.071554000
6	-2.386068000	4.144853000	0.127705000
6	-1.039143000	4.071210000	0.367473000
6	-0.643284000	2.674457000	0.320335000
6	0.684505000	2.235465000	0.460069000
6	1.391021000	1.014453000	0.385819000
6	2.828598000	1.012918000	0.113899000
6	3.252906000	-0.309557000	0.049957000
6	2.062407000	-1.119539000	0.257022000
6	1.844909000	-2.487259000	0.062976000
6	0.541171000	-3.009222000	-0.020806000
6	0.079694000	-4.289682000	-0.481982000
7	-0.542356000	-2.219381000	0.246201000
7	-3.304252000	0.017605000	-0.232059000
7	-1.775525000	1.960144000	0.038979000
7	0.994737000	-0.290697000	0.504812000
1	-1.976055000	-4.988032000	-0.850617000
1	-5.845252000	-2.066703000	-0.494243000
1	-6.550787000	0.528326000	-0.598700000
1	-3.006920000	5.030013000	0.099520000
1	-0.359886000	4.889908000	0.565248000

1	0.711903000	-5.113860000	-0.783546000
1	-3.694252000	-3.337082000	-0.364442000
1	-4.979168000	2.952295000	-0.378546000
1	1.378350000	3.067039000	0.559895000
1	2.676402000	-3.146968000	-0.141267000
7	3.568635000	2.198440000	-0.027766000
6	4.916768000	2.218152000	0.528832000
1	5.675011000	1.775160000	-0.135286000
1	5.198625000	3.259504000	0.722715000
1	4.929570000	1.670581000	1.472922000
6	3.433939000	2.944402000	-1.279606000
1	3.682708000	3.997810000	-1.106841000
1	4.102369000	2.565897000	-2.072616000
1	2.406816000	2.884756000	-1.643289000
7	4.544015000	-0.751536000	-0.248294000
6	5.032041000	-1.994388000	0.330283000
1	4.832376000	-2.875228000	-0.303731000
1	6.118835000	-1.927218000	0.461522000
1	4.576767000	-2.154985000	1.309645000
6	5.055699000	-0.491423000	-1.590379000
1	6.151573000	-0.471910000	-1.572260000
1	4.733841000	-1.266066000	-2.308404000
1	4.698778000	0.472911000	-1.949839000

E= –1581.9617256 a.u.
H= –1581.9617256 + 0.470282 (thermal correction 6-31+G**) a. u.
G= –1581.9617256 + 0.386894 (thermal correction 6-31+G**) a. u.

2b – akamptisomer *parvo,amplo* ($\boldsymbol{t_2}$)

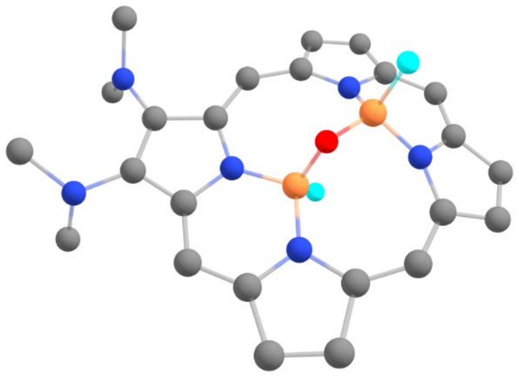

9	-2.614209000	-1.113705000	2.173719000
5	-0.424081000	0.756210000	-0.040231000
5	-2.077412000	-0.640192000	0.962249000
8	-1.024777000	0.231540000	1.119844000
9	-0.963616000	0.333027000	-1.288302000
6	-1.346363000	4.393134000	0.156136000
6	-1.707076000	2.989568000	0.114952000
6	-3.035459000	2.528853000	0.117073000
6	-3.708915000	1.288856000	0.018046000
6	-5.092348000	1.234130000	-0.419487000
6	-5.429354000	-0.079026000	-0.619170000
6	-4.268624000	-0.856182000	-0.282131000
6	-4.004125000	-2.224398000	-0.455777000
6	-2.703163000	-2.724822000	-0.330320000
6	-2.142498000	-3.989983000	-0.724247000
6	-0.788284000	-3.888301000	-0.551568000
6	-0.486929000	-2.550048000	-0.068380000
6	0.854813000	-2.114250000	0.050575000

6	1.551341000	-0.895570000	0.089891000
6	3.014324000	-0.799407000	0.118559000
6	3.346571000	0.545468000	-0.010589000
6	2.094239000	1.278870000	-0.074250000
6	1.845530000	2.653485000	-0.066505000
6	0.530390000	3.144563000	-0.003232000
6	0.020086000	4.488660000	0.088500000
7	-0.532467000	2.289628000	0.008170000
7	-3.278645000	-0.008211000	0.142876000
7	-1.685289000	-1.910627000	0.102560000
7	1.067361000	0.379813000	-0.026836000
1	-2.058521000	5.204034000	0.234439000
1	-5.701801000	2.106004000	-0.618137000
1	-6.361133000	-0.475458000	-0.999201000
1	-2.701221000	-4.826743000	-1.121137000
1	-0.037563000	-4.629882000	-0.791414000
1	0.623903000	5.385716000	0.105169000
1	-3.738574000	3.358694000	0.094010000
1	-4.795086000	-2.878037000	-0.805490000
1	1.537281000	-2.955512000	-0.007210000
1	2.677519000	3.346358000	-0.056886000
7	3.927043000	-1.858992000	0.195331000
6	3.808710000	-2.878960000	1.225894000
1	3.338659000	-3.810789000	0.868701000
1	4.807902000	-3.141128000	1.600768000
1	3.217769000	-2.496035000	2.060245000
6	4.595971000	-2.284091000	-1.024774000
1	5.583298000	-2.699516000	-0.784873000
1	4.024376000	-3.054591000	-1.572622000
1	4.735235000	-1.428622000	-1.688755000
7	4.590615000	1.176629000	0.000168000
6	5.690980000	0.526967000	0.702843000
1	6.174574000	-0.268858000	0.115801000
1	6.444261000	1.286864000	0.938855000
1	5.327292000	0.092005000	1.634747000
6	5.001844000	1.858657000	-1.227471000
1	5.745218000	2.627406000	-0.990532000
1	5.448879000	1.159748000	-1.955989000
1	4.143539000	2.337322000	-1.701370000

E=–1581.9604042 a.u.
H= –1581.9604042 + 0.469879 (thermal correction 6-31+G**) a. u.
G= –1581.9604042 + 0.384564 (thermal correction 6-31+G**) a. u.

2b – transition state (*TS*)

9	-1.763347000	-0.530566000	2.087261000
5	-0.276061000	0.893528000	-0.820121000

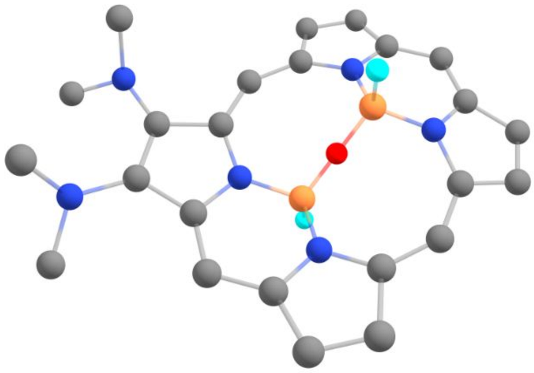

5	-1.971607000	-0.521270000	0.685759000
8	-1.123366000	0.184757000	-0.068328000
9	-0.476941000	0.902821000	-2.222629000
6	-1.080280000	4.340210000	0.400361000
6	-1.505401000	2.983049000	0.090098000
6	-2.863144000	2.553457000	0.199490000
6	-3.671122000	1.375359000	0.220980000
6	-5.115241000	1.413530000	0.040045000
6	-5.590534000	0.124227000	-0.000873000
6	-4.454686000	-0.748819000	0.183099000
6	-4.287289000	-2.147191000	0.037818000
6	-3.004068000	-2.725974000	-0.057812000
6	-2.541742000	-4.016058000	-0.522285000
6	-1.175407000	-3.936116000	-0.631748000
6	-0.751475000	-2.590796000	-0.260010000
6	0.606017000	-2.159687000	-0.342463000
6	1.415146000	-0.984592000	-0.307303000
6	2.867038000	-1.042050000	-0.121477000
6	3.367306000	0.262625000	-0.157016000
6	2.215673000	1.137662000	-0.362499000
6	2.035526000	2.532360000	-0.251353000
6	0.744512000	3.107288000	-0.131635000
6	0.288460000	4.413319000	0.282831000
7	-0.356376000	2.327051000	-0.277101000
7	-3.365018000	0.044406000	0.367373000
7	-1.899340000	-1.953832000	0.137264000
7	1.118495000	0.339765000	-0.492834000
1	-1.745845000	5.128788000	0.726827000
1	-5.688306000	2.320708000	-0.101427000
1	-6.608078000	-0.194725000	-0.181783000
1	-3.173072000	-4.856659000	-0.776876000
1	-0.505710000	-4.707544000	-0.989114000
1	0.920640000	5.264331000	0.497631000
1	-3.506447000	3.427764000	0.294316000
1	-5.160632000	-2.767367000	-0.128869000
1	1.258507000	-3.021661000	-0.480191000
1	2.895106000	3.179229000	-0.143814000
7	3.556496000	-2.252473000	0.033568000
7	4.694714000	0.638257000	0.063624000
6	3.414611000	-2.974368000	1.298693000
1	3.588971000	-4.043616000	1.132952000
1	4.132732000	-2.629257000	2.062924000
1	2.406345000	-2.844176000	1.694499000
6	4.876885000	-2.368300000	-0.573188000
1	5.686924000	-1.959670000	0.050545000
1	5.086305000	-3.429335000	-0.751517000
1	4.886492000	-1.843506000	-1.529990000

6	5.204412000	1.848448000	-0.563125000
1	4.736332000	1.988798000	-1.539756000
1	5.037167000	2.754064000	0.045353000
1	6.286250000	1.748794000	-0.710254000
6	5.243988000	0.399974000	1.396618000
1	6.336374000	0.332603000	1.340134000
1	4.980180000	1.211036000	2.097706000
1	4.859700000	-0.535790000	1.800702000

E= –1581.9185566 a.u.
H= –1581.9185566 + 0.468725 (thermal correction 6-31+G**) a. u.
G= –1581.9185566 + 0.385756 (thermal correction 6-31+G**) a. u.
f = 183.9649 i

2b– akamptisomer *amplo, amplo* (c_1)

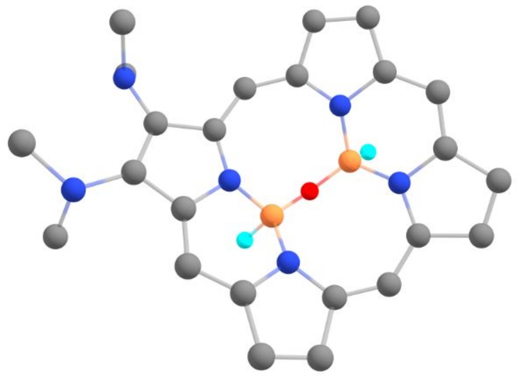

9	2.902001000	-1.311682000	1.914010000
5	0.219146000	1.008800000	1.005001000
5	2.148046000	-0.701770000	0.882456000
8	1.213166000	0.155480000	1.399493000
9	-0.382944000	1.639991000	2.122292000
6	1.534429000	4.242948000	-0.469738000
6	1.840743000	2.900176000	-0.027963000
6	3.168351000	2.453084000	0.035919000
6	3.777192000	1.198897000	-0.115312000
6	5.129441000	1.085700000	-0.617852000
6	5.345174000	-0.223948000	-0.959828000
6	4.145560000	-0.937042000	-0.634796000
6	3.818347000	-2.285476000	-0.823010000
6	2.536000000	-2.749109000	-0.533570000
6	1.951635000	-4.043990000	-0.739471000
6	0.651505000	-3.960187000	-0.321776000
6	0.405729000	-2.597500000	0.111847000
6	-0.902587000	-2.146805000	0.341757000
6	-1.535458000	-0.899884000	0.249720000
6	-2.971632000	-0.795581000	-0.012169000
6	-3.250147000	0.535610000	-0.279292000
6	-1.981817000	1.238975000	-0.184143000
6	-1.674709000	2.561278000	-0.478163000
6	-0.353398000	3.023126000	-0.398236000
6	0.189210000	4.307337000	-0.725916000
7	0.659275000	2.199772000	0.040171000
7	3.227885000	-0.059294000	-0.098503000
7	1.583871000	-1.906172000	0.007847000
7	-0.992450000	0.355871000	0.197584000
1	2.276219000	5.012317000	-0.639311000
1	5.797225000	1.924446000	-0.764702000

1	6.225692000	-0.656690000	-1.415101000
1	2.459291000	-4.896023000	-1.171134000
1	-0.108322000	-4.729238000	-0.369019000
1	-0.375170000	5.142660000	-1.117801000
1	3.882781000	3.269881000	-0.028547000
1	4.553205000	-2.962526000	-1.242732000
1	-1.609564000	-2.966572000	0.382555000
1	-2.450814000	3.229093000	-0.830347000
7	-3.916035000	-1.849904000	-0.012569000
6	-3.897938000	-2.788308000	-1.128837000
1	-3.644944000	-2.258975000	-2.050702000
1	-3.176574000	-3.615170000	-1.006589000
1	-4.894396000	-3.232300000	-1.247172000
6	-4.225661000	-2.452394000	1.280055000
1	-5.204278000	-2.944890000	1.221977000
1	-3.488388000	-3.205166000	1.610335000
1	-4.277474000	-1.672101000	2.042944000
7	-4.436474000	1.167751000	-0.637470000
6	-5.518171000	0.371286000	-1.209250000
1	-5.119313000	-0.320361000	-1.951824000
1	-6.069920000	-0.209294000	-0.456229000
1	-6.214080000	1.053948000	-1.709515000
6	-4.927550000	2.211405000	0.270277000
1	-5.545877000	2.923706000	-0.286211000
1	-5.538682000	1.777613000	1.079391000
1	-4.097428000	2.748121000	0.728849000

E= –1581.9498308 a.u.
H= –1581.9498308 + 0.469866 (thermal correction 6-31+G**) a. u.
G= –1581.9498308 + 0.385248 (thermal correction 6-31+G**) a. u.

2c – akamptisomer *amplo,parvo* ($\boldsymbol{t_1}$)

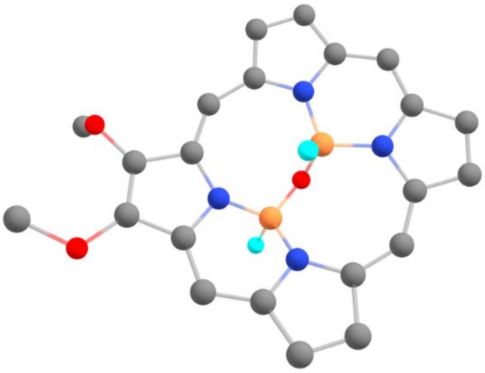

9	0.897857000	0.018027000	-1.264117000
5	-0.054664000	0.896206000	1.093323000
5	1.505526000	-0.473902000	-0.075403000
8	0.989390000	0.003497000	1.144404000
9	-0.512391000	1.315112000	2.356109000
6	1.169066000	4.208658000	-0.341411000
6	1.489870000	2.845927000	0.047945000
6	2.825379000	2.388136000	0.017316000
6	3.501418000	1.154191000	-0.061016000
6	4.942711000	1.034401000	-0.154834000
6	5.256938000	-0.295565000	-0.275628000
6	4.014164000	-1.023037000	-0.264149000
6	3.740516000	-2.399564000	-0.323167000
6	2.427242000	-2.874607000	-0.224263000
6	1.895400000	-4.214243000	-0.198458000

6	0.537644000	-4.102681000	-0.049182000
6	0.202154000	-2.692746000	0.028166000
6	-1.119450000	-2.215198000	0.140175000
6	-1.770420000	-0.968249000	0.172357000
6	-3.201422000	-0.876964000	-0.121508000
6	-3.524331000	0.456505000	-0.250499000
6	-2.312778000	1.199996000	0.015842000
6	-2.055999000	2.563960000	-0.113554000
6	-0.736748000	3.046314000	-0.052044000
6	-0.193203000	4.325591000	-0.419481000
7	0.294733000	2.204824000	0.270550000
7	2.999246000	-0.117008000	-0.142490000
7	1.377527000	-2.005828000	-0.093967000
7	-1.311354000	0.315586000	0.319347000
1	1.908062000	4.957591000	-0.594296000
1	5.628322000	1.870995000	-0.125942000
1	6.239776000	-0.739188000	-0.357960000
1	2.481453000	-5.119717000	-0.277810000
1	-0.185395000	-4.905368000	0.010645000
1	-0.770015000	5.183893000	-0.735978000
1	3.521650000	3.220314000	-0.061751000
1	4.558615000	-3.103369000	-0.421429000
1	-1.839354000	-3.026892000	0.072501000
1	-2.866871000	3.232292000	-0.376645000
8	-4.006382000	-1.962897000	-0.340385000
6	-4.560361000	-2.533800000	0.858166000
1	-3.770239000	-2.816600000	1.563630000
1	-5.238021000	-1.823642000	1.348603000
1	-5.115819000	-3.420401000	0.546587000
8	-4.657893000	1.100156000	-0.589744000
6	-5.747055000	0.316599000	-1.099425000
1	-5.421009000	-0.296527000	-1.944386000
1	-6.161039000	-0.331460000	-0.320475000
1	-6.500005000	1.037484000	-1.419185000

E= –1543.0512701 a.u.
H= –1543.0512701 + 0.386534 (thermal correction 6-31+G**) a. u.
G= –1543.0512701 + 0.308945 (thermal correction 6-31+G**) a. u.

2c – akamptisomer *parvo,amplo* ($\boldsymbol{t_2}$)

9	-2.309753000	-0.959007000	2.246239000
5	-0.077361000	0.733811000	-0.062354000
5	-1.755280000	-0.571969000	1.013450000
8	-0.691384000	0.292344000	1.124087000
9	-0.620864000	0.246109000	-1.284030000
6	-0.923800000	4.390636000	-0.094314000
6	-1.309727000	2.993834000	-0.057964000

6	-2.646313000	2.553994000	-0.047008000
6	-3.340485000	1.321946000	-0.071797000
6	-4.721918000	1.263961000	-0.517754000
6	-5.083919000	-0.052413000	-0.630013000
6	-3.941336000	-0.827566000	-0.229707000
6	-3.704846000	-2.210092000	-0.301578000
6	-2.415779000	-2.728301000	-0.130482000
6	-1.884647000	-4.035262000	-0.414727000
6	-0.529366000	-3.953108000	-0.241222000
6	-0.197220000	-2.585816000	0.127070000
6	1.153036000	-2.166030000	0.186992000
6	1.857467000	-0.956140000	0.115130000
6	3.314257000	-0.885731000	0.110646000
6	3.676038000	0.442230000	0.012552000
6	2.448493000	1.206386000	-0.071743000
6	2.239620000	2.583286000	-0.145358000
6	0.932479000	3.102424000	-0.146152000
6	0.445351000	4.457834000	-0.144161000
7	-0.147596000	2.268503000	-0.104966000
7	-2.937964000	0.027464000	0.144404000
7	-1.380474000	-1.905163000	0.239811000
7	1.404336000	0.326788000	-0.024458000
1	-1.621579000	5.217398000	-0.075914000
1	-5.312468000	2.131949000	-0.780218000
1	-6.020808000	-0.455572000	-0.989780000
1	-2.462182000	-4.888362000	-0.744095000
1	0.203908000	-4.730244000	-0.412466000
1	1.065164000	5.343616000	-0.169647000
1	-3.334850000	3.391757000	-0.134088000
1	-4.508300000	-2.871111000	-0.605984000
1	1.835182000	-3.012467000	0.213523000
1	3.092529000	3.249992000	-0.172331000
8	4.127747000	-1.981571000	0.212201000
6	4.457629000	-2.586928000	-1.049993000
1	3.551700000	-2.882059000	-1.592428000
1	5.038223000	-1.895998000	-1.674264000
1	5.057017000	-3.469540000	-0.819152000
8	4.871580000	1.065597000	-0.005784000
6	6.010500000	0.330417000	0.467884000
1	5.806305000	-0.103295000	1.451519000
1	6.283367000	-0.467771000	-0.228906000
1	6.820802000	1.057109000	0.533507000

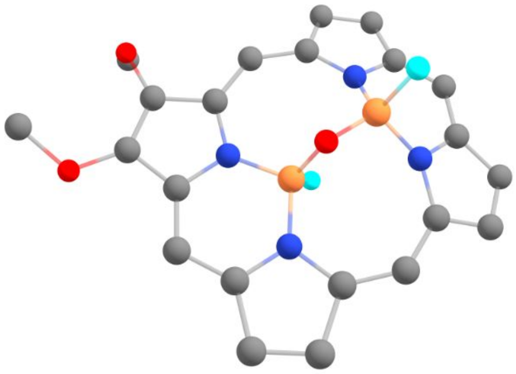

E= –1543.050475 a.u.
H= –1543.050475 + 0.386444 (thermal correction 6-31+G**) a. u.
G= –1543.050475 + 0.308574 (thermal correction 6-31+G**) a. u.

2c – transition state (*TS*)

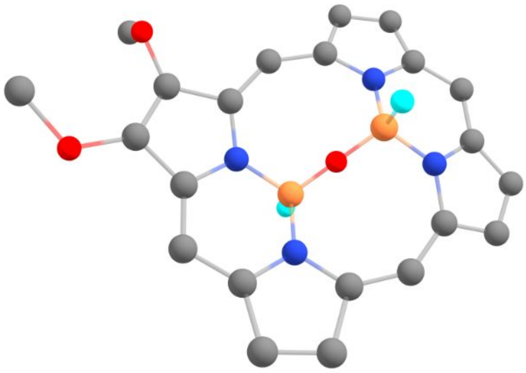

9	1.535892000	-0.434225000	-2.129878000
5	-0.062175000	0.853902000	0.784344000
5	1.664951000	-0.517502000	-0.722820000
8	0.801147000	0.170412000	0.028832000
9	0.071586000	0.770198000	2.191543000
6	0.887954000	4.332368000	-0.225582000
6	1.259169000	2.951616000	0.053159000
6	2.608217000	2.488731000	-0.010181000
6	3.387598000	1.289423000	-0.055569000
6	4.822185000	1.272945000	0.192368000
6	5.260425000	-0.030496000	0.170671000
6	4.111384000	-0.855642000	-0.120822000
6	3.896149000	-2.255055000	-0.090321000
6	2.594762000	-2.801011000	-0.102582000
6	2.074235000	-4.115295000	0.201670000
6	0.705113000	-4.005615000	0.237589000
6	0.338476000	-2.617029000	-0.013344000
6	-1.014450000	-2.152275000	0.046931000
6	-1.782615000	-0.957893000	0.119738000
6	-3.232499000	-0.947208000	-0.096019000
6	-3.667339000	0.366761000	-0.082688000
6	-2.504629000	1.192913000	0.191827000
6	-2.304827000	2.582995000	0.141988000
6	-0.997262000	3.131315000	0.145867000
6	-0.481937000	4.439201000	-0.188166000
7	0.073283000	2.315183000	0.332203000
7	3.053606000	-0.019091000	-0.296778000
7	1.521275000	-1.978440000	-0.272296000
7	-1.450958000	0.350737000	0.363843000
1	1.592283000	5.113574000	-0.480470000
1	5.413069000	2.151647000	0.416137000
1	6.260075000	-0.389690000	0.374364000
1	2.667249000	-4.997149000	0.403147000
1	-0.002672000	-4.790049000	0.472024000
1	-1.078390000	5.314403000	-0.407213000
1	3.278078000	3.347889000	-0.032455000
1	4.743203000	-2.914669000	0.060486000
1	-1.689520000	-3.005835000	0.011427000
1	-3.158406000	3.229946000	-0.020639000
8	-3.972259000	-2.069322000	-0.357376000
8	-4.870104000	0.931490000	-0.306605000
6	-4.463020000	-2.735743000	0.818492000
1	-3.640335000	-3.010685000	1.489344000
1	-5.167825000	-2.095349000	1.363881000
1	-4.974232000	-3.634983000	0.469720000

6	-5.885409000	0.127054000	-0.926139000
1	-6.236411000	-0.655314000	-0.246545000
1	-6.701736000	0.813069000	-1.153997000
1	-5.506389000	-0.334297000	-1.842735000

E= –1543.0058519 a.u.
H= –1543.0058519 + 0.384814 (thermal correction 6-31+G**) a. u.
G= –1543.0058519 + 0.307324 (thermal correction 6-31+G**) a. u.
f = 186.7578 i

2c– akamptisomer *amplo, amplo* (c_1)

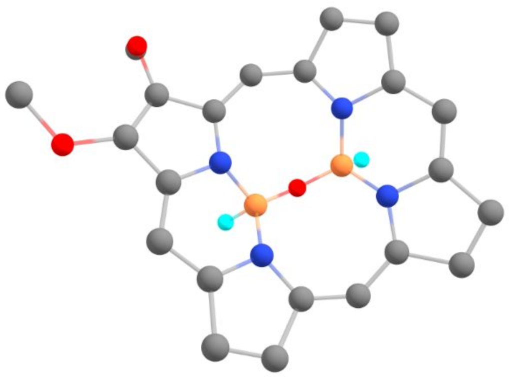

9	2.541072000	-1.406003000	1.878243000
5	-0.126000000	0.939633000	0.986232000
5	1.805201000	-0.757824000	0.857834000
8	0.861054000	0.080847000	1.389351000
9	-0.788032000	1.508995000	2.101522000
6	1.209510000	4.248382000	-0.275662000
6	1.511547000	2.883720000	0.100620000
6	2.836775000	2.433972000	0.178243000
6	3.448121000	1.186527000	-0.022104000
6	4.810901000	1.097700000	-0.500710000
6	5.036222000	-0.194372000	-0.898496000
6	3.831740000	-0.922822000	-0.631227000
6	3.512920000	-2.261964000	-0.883189000
6	2.228297000	-2.744795000	-0.636241000
6	1.656988000	-4.031066000	-0.914705000
6	0.348168000	-3.974795000	-0.518514000
6	0.087006000	-2.637508000	-0.024471000
6	-1.227609000	-2.202094000	0.209788000
6	-1.839919000	-0.948733000	0.157693000
6	-3.264481000	-0.824422000	-0.126177000
6	-3.507649000	0.490408000	-0.464103000
6	-2.257242000	1.192424000	-0.312154000
6	-1.976978000	2.538165000	-0.510673000
6	-0.670500000	3.014717000	-0.338254000
6	-0.126899000	4.320156000	-0.570405000
7	0.334195000	2.175472000	0.092791000
7	2.900415000	-0.071195000	-0.075754000
7	1.261188000	-1.933117000	-0.072962000
7	-1.283199000	0.303578000	0.092599000
1	1.950615000	5.030186000	-0.377979000
1	5.480187000	1.942855000	-0.594061000
1	5.927049000	-0.604779000	-1.354373000
1	2.178194000	-4.858077000	-1.377663000
1	-0.406282000	-4.744042000	-0.617802000
1	-0.683954000	5.172525000	-0.934878000
1	3.553111000	3.251564000	0.174244000

1	4.257602000	-2.917115000	-1.320148000
1	-1.947736000	-3.015145000	0.189224000
1	-2.765866000	3.205919000	-0.834216000
8	-4.128826000	-1.882258000	-0.187795000
6	-4.606547000	-2.333160000	1.093448000
1	-3.774668000	-2.575469000	1.764074000
1	-5.231933000	-1.563618000	1.563000000
1	-5.201889000	-3.227197000	0.899903000
8	-4.604214000	1.144551000	-0.889648000
6	-5.814888000	0.392141000	-1.057120000
1	-5.667762000	-0.432664000	-1.759315000
1	-6.159363000	-0.005329000	-0.096496000
1	-6.545880000	1.100641000	-1.447246000

E= –1543.042268 a.u.
H= –1543.042268 + 0.386182 (thermal correction 6-31+G**) a. u.
G= –1543.042268 + 0.307879 (thermal correction 6-31+G**) a. u.

2d – akamptisomer *amplo,parvo* (***t***$_1$)

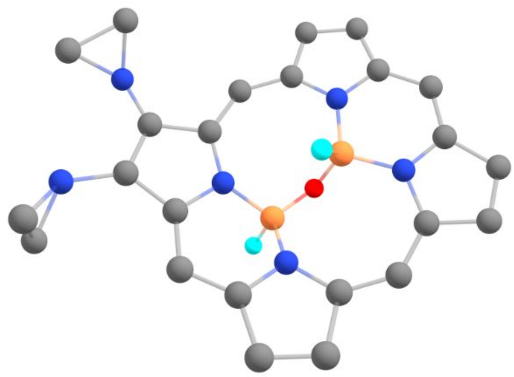

9	-1.047550000	0.026537000	-1.219765000
5	-0.161155000	-0.919880000	1.143816000
5	-1.771890000	0.377743000	-0.045904000
8	-1.282061000	-0.125182000	1.173555000
9	0.250701000	-1.373668000	2.410218000
6	-0.994002000	-4.243844000	-0.516218000
6	-1.464415000	-2.932076000	-0.101760000
6	-2.829545000	-2.593017000	-0.207138000
6	-3.608253000	-1.419047000	-0.262517000
6	-5.046195000	-1.420716000	-0.442038000
6	-5.473750000	-0.118383000	-0.497867000
6	-4.306547000	0.712955000	-0.358803000
6	-4.163342000	2.108068000	-0.297043000
6	-2.909767000	2.690875000	-0.071899000
6	-2.513132000	4.061517000	0.130064000
6	-1.164614000	4.054852000	0.379121000
6	-0.701070000	2.680817000	0.335688000
6	0.650544000	2.310504000	0.493190000
6	1.422145000	1.134817000	0.435446000
6	2.868889000	1.202216000	0.192929000
6	3.349213000	-0.087963000	0.073398000
6	2.200408000	-0.963501000	0.226613000
6	2.045206000	-2.344838000	0.035690000
6	0.771738000	-2.932810000	-0.035648000
6	0.374848000	-4.239301000	-0.490068000
7	-0.350235000	-2.200102000	0.232716000
7	-3.219658000	-0.107195000	-0.232692000
7	-1.791770000	1.911078000	0.046682000

7	1.090806000	-0.194806000	0.486251000
1	-1.641894000	-5.042546000	-0.852856000
1	-5.652377000	-2.314494000	-0.508647000
1	-6.486860000	0.242052000	-0.613063000
1	-3.177036000	4.914671000	0.098906000
1	-0.529422000	4.906658000	0.583359000
1	1.047983000	-5.031192000	-0.789539000
1	-3.440753000	-3.476399000	-0.378754000
1	-5.039321000	2.739378000	-0.388881000
1	1.282651000	3.186229000	0.606308000
1	2.902376000	-2.971023000	-0.169775000
7	3.582351000	2.395410000	0.141310000
6	3.387395000	3.356912000	-0.955860000
6	4.662293000	2.594388000	-0.820819000
1	3.332410000	4.399534000	-0.653447000
1	2.706054000	3.065806000	-1.753580000
1	5.549040000	3.077047000	-0.419134000
1	4.853984000	1.794723000	-1.529776000
7	4.713624000	-0.377672000	-0.171224000
6	5.327712000	-1.592367000	0.380006000
6	5.103884000	-1.460934000	-1.088454000
1	4.696590000	-2.275932000	0.943290000
1	6.323522000	-1.448551000	0.792908000
1	4.316230000	-2.051111000	-1.550508000
1	5.936481000	-1.220268000	-1.746252000

E= –1579.4657182 a.u.
H= –1579.4657182 + 0.423446 (thermal correction 6-31+G**) a. u.
G= –1579.4657182 + 0.344291 (thermal correction 6-31+G**) a. u.

2d – akamptisomer *parvo,amplo* (t_2)

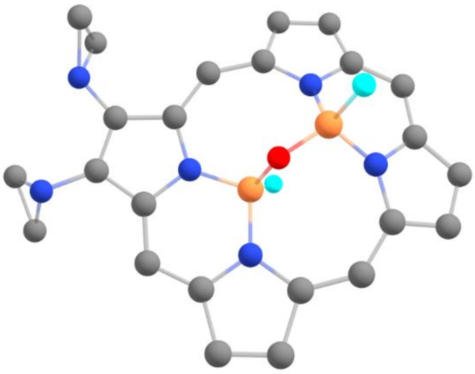

9	-2.571558000	-1.080520000	2.160394000
5	-0.319457000	0.753683000	-0.020657000
5	-2.010507000	-0.616828000	0.956814000
8	-0.948958000	0.240920000	1.128331000
9	-0.837082000	0.331068000	-1.276959000
6	-1.223844000	4.401570000	0.147352000
6	-1.591411000	3.000259000	0.100654000
6	-2.924704000	2.553744000	0.076042000
6	-3.609615000	1.321891000	-0.024705000
6	-4.987896000	1.277779000	-0.480200000
6	-5.335559000	-0.033304000	-0.672531000
6	-4.186752000	-0.819104000	-0.314022000
6	-3.935098000	-2.191693000	-0.472222000
6	-2.640477000	-2.702672000	-0.328599000
6	-2.086445000	-3.975650000	-0.706515000
6	-0.733468000	-3.881650000	-0.524537000

6	-0.425372000	-2.539639000	-0.052163000
6	0.923390000	-2.121223000	0.057183000
6	1.654262000	-0.918968000	0.097077000
6	3.120611000	-0.815681000	0.142721000
6	3.439043000	0.530151000	0.093698000
6	2.195748000	1.267582000	0.032216000
6	1.959499000	2.642415000	0.024952000
6	0.646915000	3.142170000	0.039879000
6	0.144103000	4.489153000	0.114116000
7	-0.419431000	2.291343000	0.022988000
7	-3.193487000	0.022073000	0.115887000
7	-1.620132000	-1.892449000	0.106078000
7	1.171723000	0.364477000	0.021506000
1	-1.933360000	5.216345000	0.206663000
1	-5.585780000	2.154109000	-0.693723000
1	-6.266378000	-0.424041000	-1.060657000
1	-2.647988000	-4.811935000	-1.100279000
1	0.012800000	-4.631702000	-0.752160000
1	0.753285000	5.382065000	0.146515000
1	-3.617549000	3.391334000	0.033201000
1	-4.728713000	-2.840551000	-0.824656000
1	1.570101000	-2.986071000	-0.002181000
1	2.796842000	3.328402000	0.051145000
7	4.142325000	-1.787823000	0.265111000
6	3.995225000	-3.009209000	1.061770000
6	4.121837000	-3.083003000	-0.425003000
1	3.027941000	-3.221982000	1.511353000
1	4.850255000	-3.245315000	1.691576000
1	3.246526000	-3.351148000	-1.012052000
1	5.070135000	-3.369392000	-0.875595000
7	4.684652000	1.132694000	0.211442000
6	5.823466000	0.664060000	-0.567089000
6	5.207592000	1.996348000	-0.854324000
1	5.643624000	-0.140631000	-1.274916000
1	6.761042000	0.600358000	-0.021986000
1	4.609999000	2.090032000	-1.760017000
1	5.690582000	2.909397000	-0.516173000

E= –1579.4635557 a.u.
H= –1579.4635557 + 0.423390 (thermal correction 6-31+G**) a. u.
G= –1579.4635557 + 0.344064 (thermal correction 6-31+G**) a. u.

2d – transition state (*TS*)

9	1.799880000	-0.503905000	-2.102224000
5	0.165040000	0.913814000	0.725949000
5	1.920998000	-0.515262000	-0.692376000
8	1.048890000	0.204129000	0.020539000

9	0.271125000	0.894507000	2.138795000
6	1.135783000	4.354344000	-0.403399000
6	1.501840000	2.983947000	-0.074264000
6	2.852841000	2.523541000	-0.106398000
6	3.635248000	1.327712000	-0.097476000
6	5.067235000	1.327939000	0.165019000
6	5.508809000	0.026615000	0.210032000
6	4.364482000	-0.814712000	-0.052156000
6	4.152714000	-2.210343000	0.058939000
6	2.851905000	-2.755864000	0.074285000
6	2.331650000	-4.039417000	0.490374000
6	0.964184000	-3.922670000	0.528598000
6	0.596613000	-2.559410000	0.159559000
6	-0.759859000	-2.105185000	0.192722000
6	-1.568943000	-0.926731000	0.146220000
6	-3.028163000	-0.913805000	-0.086111000
6	-3.438471000	0.412888000	-0.118127000
6	-2.266419000	1.236942000	0.104712000
6	-2.059046000	2.623527000	0.008179000
6	-0.752555000	3.170603000	-0.005264000
6	-0.234189000	4.465025000	-0.380216000
7	0.312941000	2.357330000	0.214551000
7	3.306425000	0.008221000	-0.282131000
7	1.779178000	-1.950032000	-0.165341000
7	-1.219382000	0.392702000	0.305745000
1	1.844027000	5.124937000	-0.678931000
1	5.653025000	2.218304000	0.353246000
1	6.506782000	-0.320062000	0.441676000
1	2.924998000	-4.901690000	0.762973000
1	0.257415000	-4.682639000	0.836153000
1	-0.827522000	5.332892000	-0.634169000
1	3.520022000	3.383740000	-0.153468000
1	4.998793000	-2.856935000	0.262010000
1	-1.390915000	-2.979435000	0.296634000
1	-2.907316000	3.270022000	-0.181883000
7	-3.959887000	-1.942958000	-0.363418000
6	-3.637898000	-3.122325000	-1.172249000
6	-3.943386000	-3.258057000	0.284210000
1	-2.609022000	-3.257556000	-1.498401000
1	-4.390326000	-3.386069000	-1.912407000
1	-3.134234000	-3.499803000	0.969552000
1	-4.920515000	-3.615612000	0.603341000
7	-4.683540000	0.922314000	-0.458350000
6	-5.902643000	0.401166000	0.144231000
6	-5.424951000	1.780406000	0.473427000
1	-5.791338000	-0.368865000	0.902890000
1	-6.733412000	0.259078000	-0.541315000

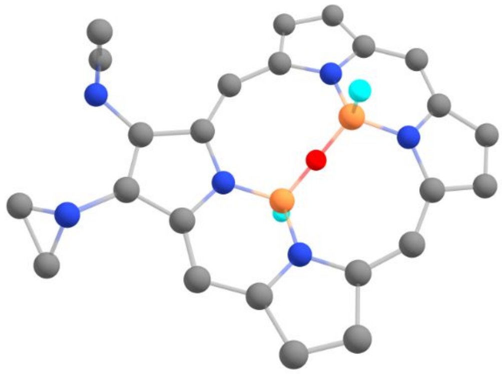

1	-4.989551000	1.940533000	1.458824000
1	-5.902087000	2.648105000	0.025494000

E= –1579.4191075 a.u.
H= –1579.4191075 + 0.421788 (thermal correction 6-31+G**) a. u.
G= –1579.4191075 + 0.342778 (thermal correction 6-31+G**) a. u.
f = 184.8487 i

2d– akamptisomer *amplo, amplo* (c_1)

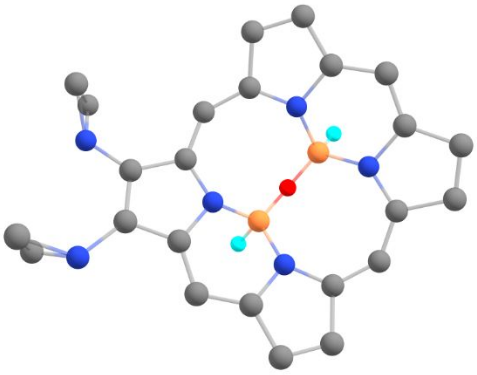

9	2.812476000	-1.453008000	1.848009000
5	0.221902000	1.003631000	1.036422000
5	2.066575000	-0.774856000	0.854427000
8	1.185509000	0.109538000	1.419098000
9	-0.367714000	1.625272000	2.162402000
6	1.678819000	4.228853000	-0.309868000
6	1.925345000	2.852336000	0.068765000
6	3.231390000	2.344285000	0.105477000
6	3.770489000	1.067854000	-0.112974000
6	5.105106000	0.902790000	-0.649502000
6	5.241302000	-0.400868000	-1.048147000
6	4.011354000	-1.062085000	-0.723296000
6	3.611823000	-2.383304000	-0.945029000
6	2.315906000	-2.795237000	-0.629321000
6	1.672557000	-4.060613000	-0.829263000
6	0.395417000	-3.932909000	-0.350428000
6	0.223326000	-2.569164000	0.107439000
6	-1.058196000	-2.071953000	0.403332000
6	-1.645728000	-0.802985000	0.319343000
6	-3.079398000	-0.587745000	0.066082000
6	-3.233647000	0.740301000	-0.287197000
6	-1.937181000	1.349241000	-0.191015000
6	-1.588256000	2.677112000	-0.423725000
6	-0.259066000	3.087831000	-0.302585000
6	0.340420000	4.363709000	-0.564718000
7	0.716657000	2.201587000	0.104622000
7	3.153326000	-0.156571000	-0.133551000
7	1.420740000	-1.922105000	-0.040726000
7	-1.009817000	0.410947000	0.200510000
1	2.454490000	4.971907000	-0.440850000
1	5.814711000	1.709664000	-0.777271000
1	6.087395000	-0.861583000	-1.539832000
1	2.126147000	-4.926095000	-1.293071000
1	-0.393589000	-4.673472000	-0.372490000
1	-0.186503000	5.239048000	-0.919336000
1	3.984808000	3.126914000	0.070586000
1	4.301596000	-3.083452000	-1.401802000

1	-1.769470000	-2.879188000	0.503526000
1	-2.361174000	3.370664000	-0.730729000
7	-4.187111000	-1.469376000	0.078439000
6	-4.183528000	-2.803638000	-0.538108000
6	-4.291489000	-2.645908000	0.943297000
1	-3.250206000	-3.175687000	-0.954048000
1	-5.069121000	-3.035106000	-1.126543000
1	-3.443335000	-2.920954000	1.565184000
1	-5.258924000	-2.759640000	1.427988000
7	-4.344127000	1.516181000	-0.685825000
6	-5.438063000	0.908576000	-1.469774000
6	-5.660148000	1.291321000	-0.051836000
1	-5.370432000	-0.153208000	-1.687506000
1	-5.807574000	1.540017000	-2.274357000
1	-5.733470000	0.482191000	0.668946000
1	-6.193667000	2.205992000	0.194898000

E= –1579.4636844 a.u.
H= –1579.4636844 + 0.422618 (thermal correction 6-31+G**) a. u.
G= –1579.4636844 + 0.342764 (thermal correction 6-31+G**) a. u.

2e – akamptisomer *amplo,parvo* ($\boldsymbol{t}_1$)

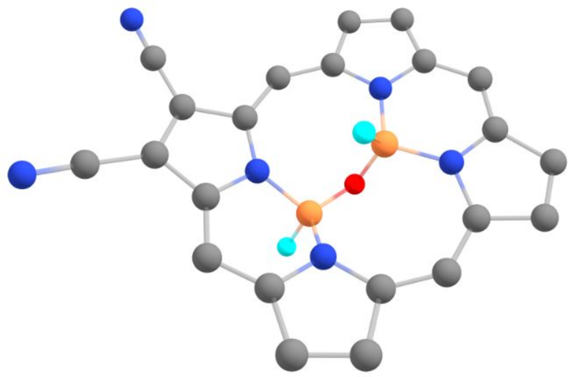

9	-0.676048000	0.010442000	-1.236992000
5	0.279947000	-0.845416000	1.120998000
5	-1.374430000	0.395830000	-0.061021000
8	-0.845080000	-0.061268000	1.161739000
9	0.758067000	-1.248525000	2.372935000
6	-0.556507000	-4.226810000	-0.422109000
6	-1.022196000	-2.906123000	-0.022173000
6	-2.400714000	-2.588266000	-0.075882000
6	-3.191157000	-1.429035000	-0.149459000
6	-4.639645000	-1.454370000	-0.278076000
6	-5.084152000	-0.164432000	-0.372596000
6	-3.919929000	0.688262000	-0.307753000
6	-3.795583000	2.082796000	-0.309943000
6	-2.538955000	2.689114000	-0.150909000
6	-2.157626000	4.077577000	-0.042711000
6	-0.803566000	4.105401000	0.151520000
6	-0.319282000	2.735018000	0.170215000
6	1.034353000	2.397994000	0.299561000
6	1.817688000	1.215889000	0.272512000
6	3.235828000	1.279030000	-0.042146000
6	3.704682000	-0.025574000	-0.173601000
6	2.586188000	-0.886316000	0.082366000
6	2.468656000	-2.279202000	-0.070913000
6	1.213273000	-2.887522000	-0.051903000
6	0.809541000	-4.210683000	-0.453082000

7	0.088758000	-2.154182000	0.248287000
7	-2.819236000	-0.112697000	-0.187069000
7	-1.412433000	1.931504000	-0.028121000
7	1.504317000	-0.111361000	0.392873000
1	-1.210745000	-5.039192000	-0.709394000
1	-5.235236000	-2.357493000	-0.287216000
1	-6.104511000	0.180468000	-0.468224000
1	-2.836456000	4.917162000	-0.100977000
1	-0.173180000	4.975832000	0.275425000
1	1.478579000	-5.002420000	-0.760931000
1	-3.005652000	-3.485489000	-0.183643000
1	-4.680755000	2.700413000	-0.402961000
1	1.661177000	3.285833000	0.332147000
1	3.349392000	-2.859329000	-0.320684000
6	3.971559000	2.473267000	-0.249897000
7	4.547919000	3.473102000	-0.405807000
6	5.002973000	-0.459433000	-0.542085000
7	6.056346000	-0.854144000	-0.842514000

E= –1498.4594096 a.u.
H= –1498.4594096 + *0.316920* (thermal correction 6-31+G**) a. u.
G= –1498.4594096 + *0.243055* (thermal correction 6-31+G**) a. u.

2e – akamptisomer *parvo,amplo* (***t*₂**)

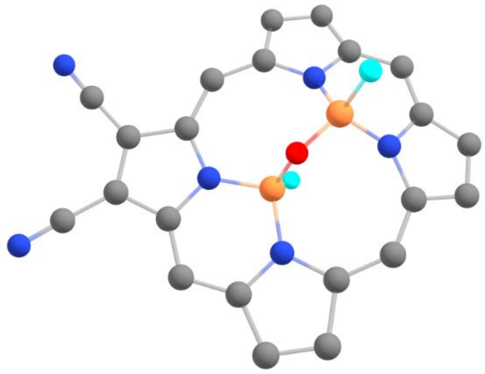

9	-2.188005000	-0.910300000	2.214378000
5	0.142936000	0.664949000	-0.072529000
5	-1.621438000	-0.530467000	0.990248000
8	-0.481634000	0.231978000	1.107503000
9	-0.441512000	0.259464000	-1.299384000
6	-0.393070000	4.382841000	0.023357000
6	-0.892027000	3.017508000	0.027101000
6	-2.263720000	2.694877000	0.048119000
6	-3.057057000	1.528556000	-0.003538000
6	-4.452062000	1.603668000	-0.420168000
6	-4.925290000	0.330903000	-0.567365000
6	-3.841113000	-0.554042000	-0.219880000
6	-3.727666000	-1.943269000	-0.346486000
6	-2.479061000	-2.574711000	-0.223841000
6	-2.068897000	-3.910591000	-0.576360000
6	-0.711233000	-3.953579000	-0.427277000
6	-0.255841000	-2.634398000	-0.008658000
6	1.118868000	-2.339999000	0.033708000
6	1.933619000	-1.186205000	0.017256000
6	3.382310000	-1.234881000	0.006275000
6	3.855458000	0.075526000	-0.067444000
6	2.701689000	0.930582000	-0.108202000
6	2.599530000	2.332516000	-0.145207000

6	1.347550000	2.948091000	-0.105458000
6	0.971896000	4.340949000	-0.055188000
7	0.199387000	2.201116000	-0.064368000
7	-2.760872000	0.200571000	0.161263000
7	-1.378183000	-1.859182000	0.158489000
7	1.596337000	0.135312000	-0.066209000
1	-1.021374000	5.261600000	0.081477000
1	-4.970736000	2.527624000	-0.639049000
1	-5.900011000	0.019529000	-0.917169000
1	-2.724292000	-4.695041000	-0.929024000
1	-0.049974000	-4.781523000	-0.646158000
1	1.662414000	5.172883000	-0.068783000
1	-2.878040000	3.590811000	-0.002011000
1	-4.588674000	-2.522527000	-0.659015000
1	1.718386000	-3.245149000	-0.027670000
1	3.502201000	2.930867000	-0.174996000
6	4.156373000	-2.421540000	0.061002000
7	4.760759000	-3.416042000	0.100262000
6	5.200059000	0.522976000	-0.092714000
7	6.291065000	0.929018000	-0.117343000

E= –1498.4586461 a.u.
H= –1498.4586461 + 0.316909 (thermal correction 6-31+G**) a. u.
G= –1498.4586461 + 0.243015 (thermal correction 6-31+G**) a. u.

2e – transition state (*TS*)

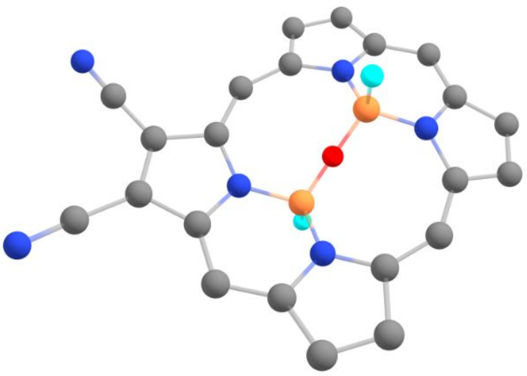

9	1.372566000	-0.418866000	-2.109640000
5	-0.296305000	0.784476000	0.795847000
5	1.534693000	-0.449021000	-0.708940000
8	0.608493000	0.155480000	0.044335000
9	-0.145772000	0.760143000	2.196695000
6	0.271106000	4.314669000	-0.322916000
6	0.783757000	2.980977000	-0.022917000
6	2.177354000	2.659977000	-0.088741000
6	3.069318000	1.548330000	-0.120409000
6	4.507231000	1.688459000	0.100970000
6	5.076163000	0.441549000	0.106022000
6	4.010764000	-0.509832000	-0.139519000
6	3.950099000	-1.917396000	-0.060819000
6	2.708679000	-2.597761000	-0.026178000
6	2.339243000	-3.946430000	0.351865000
6	0.970120000	-3.981522000	0.417909000
6	0.451487000	-2.650537000	0.112310000
6	-0.930859000	-2.333828000	0.200302000
6	-1.830370000	-1.214953000	0.213368000
6	-3.261405000	-1.357821000	-0.012308000
6	-3.836974000	-0.082010000	-0.021279000

6	-2.772102000	0.854717000	0.227577000
6	-2.710756000	2.269116000	0.145586000
6	-1.474639000	2.938626000	0.113004000
6	-1.098322000	4.286985000	-0.255492000
7	-0.318667000	2.234513000	0.290260000
7	2.868667000	0.207130000	-0.321295000
7	1.558471000	-1.902151000	-0.210532000
7	-1.642822000	0.126418000	0.413057000
1	0.889827000	5.155217000	-0.608391000
1	5.005549000	2.630435000	0.288954000
1	6.110702000	0.191997000	0.298207000
1	3.028484000	-4.748749000	0.576757000
1	0.353905000	-4.823532000	0.704661000
1	-1.784391000	5.092943000	-0.477039000
1	2.753752000	3.583205000	-0.129492000
1	4.863986000	-2.477681000	0.098857000
1	-1.505907000	-3.255480000	0.277991000
1	-3.628488000	2.824202000	-0.010521000
6	-3.929005000	-2.588080000	-0.240572000
7	-4.446084000	-3.617326000	-0.411952000
6	-5.188249000	0.262501000	-0.274468000
7	-6.288750000	0.585761000	-0.475607000

E= –1498.4141579 a.u.
H= –1498.4141579 + 0.315212 (thermal correction 6-31+G**) a. u.
G= –1498.4141579 + 0.241589 (thermal correction 6-31+G**) a. u.
f = 185.7029 i

2e– akamptisomer *amplo, amplo* (c_1)

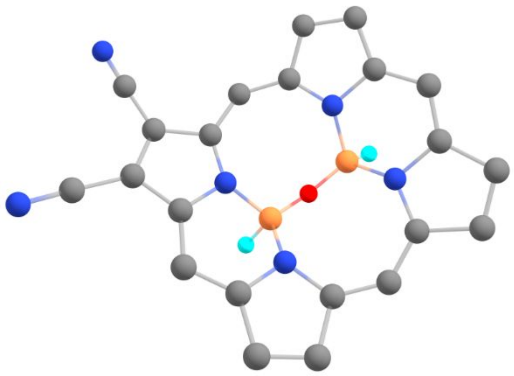

9	2.561586000	-1.183253000	1.870272000
5	-0.327929000	0.903018000	1.048439000
5	1.727372000	-0.640025000	0.870952000
8	0.734068000	0.128797000	1.417825000
9	-0.986597000	1.453249000	2.164913000
6	0.680440000	4.266122000	-0.353726000
6	1.105070000	2.932443000	0.029198000
6	2.467942000	2.594754000	0.058986000
6	3.162866000	1.396013000	-0.145741000
6	4.511867000	1.398982000	-0.683403000
6	4.819269000	0.120618000	-1.055786000
6	3.685531000	-0.693933000	-0.712506000
6	3.473345000	-2.059631000	-0.901572000
6	2.243570000	-2.639360000	-0.570354000
6	1.778942000	-3.986270000	-0.754240000
6	0.496052000	-4.027397000	-0.283187000
6	0.138298000	-2.689641000	0.151826000

6	-1.193662000	-2.354352000	0.403802000
6	-1.918359000	-1.151988000	0.301045000
6	-3.333479000	-1.163472000	-0.011838000
6	-3.704730000	0.140070000	-0.331319000
6	-2.532535000	0.946414000	-0.177838000
6	-2.360746000	2.317574000	-0.408197000
6	-1.098046000	2.890694000	-0.300814000
6	-0.667241000	4.231999000	-0.579700000
7	-0.007094000	2.135623000	0.092557000
7	2.713405000	0.101034000	-0.140565000
7	1.243170000	-1.886908000	0.002487000
7	-1.491640000	0.146537000	0.230047000
1	1.353948000	5.099641000	-0.502488000
1	5.108180000	2.290399000	-0.826114000
1	5.719242000	-0.234600000	-1.538901000
1	2.348541000	-4.787301000	-1.205152000
1	-0.188700000	-4.864950000	-0.299281000
1	-1.306087000	5.032070000	-0.927844000
1	3.114017000	3.466867000	0.006756000
1	4.250962000	-2.668099000	-1.347910000
1	-1.838899000	-3.225674000	0.473721000
1	-3.209196000	2.917438000	-0.715582000
6	-4.146113000	-2.323950000	-0.072522000
7	-4.785370000	-3.296440000	-0.112503000
6	-4.969066000	0.612385000	-0.765863000
7	-5.991323000	1.035937000	-1.127872000

E= –1498.4481009 a.u.
H= –1498.4481009 + 0.316521 (thermal correction 6-31+G**) a. u.
G= –1498.4481009 + 0.242001 (thermal correction 6-31+G**) a. u.

2f – akamptisomer *amplo,parvo* (***t***$_1$)

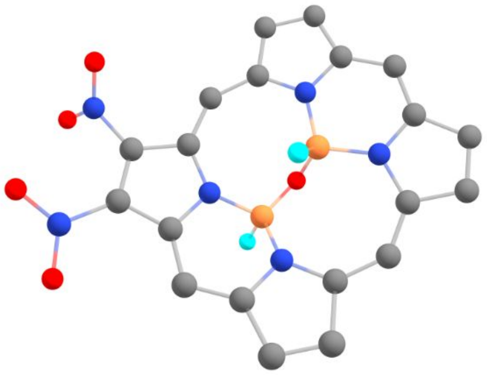

9	1.049657000	-0.009109000	-1.227101000
5	0.191731000	0.932420000	1.138453000
5	1.724548000	-0.447201000	-0.056954000
8	1.241427000	0.049868000	1.169210000
9	-0.236089000	1.375700000	2.393862000
6	1.296045000	4.228942000	-0.416502000
6	1.654020000	2.872764000	-0.020778000
6	3.000536000	2.441470000	-0.086226000
6	3.688422000	1.219368000	-0.165655000
6	5.133286000	1.122520000	-0.309859000
6	5.466394000	-0.199490000	-0.406835000
6	4.234715000	-0.951681000	-0.327445000
6	3.996011000	-2.329500000	-0.323916000
6	2.693744000	-2.829327000	-0.147337000
6	2.200605000	-4.180549000	-0.021112000

6	0.852190000	-4.093957000	0.193487000
6	0.483906000	-2.687907000	0.205782000
6	-0.832903000	-2.240459000	0.355371000
6	-1.520951000	-0.998183000	0.315060000
6	-2.933141000	-0.933791000	0.034995000
6	-3.301316000	0.389774000	-0.090873000
6	-2.123282000	1.172223000	0.132985000
6	-1.879067000	2.550829000	-0.029103000
6	-0.574830000	3.040286000	-0.027386000
6	-0.065892000	4.326749000	-0.432296000
7	0.488025000	2.215658000	0.260679000
7	3.205902000	-0.060025000	-0.196539000
7	1.637854000	-1.980148000	-0.017628000
7	-1.103218000	0.307342000	0.421734000
1	2.013174000	4.983687000	-0.710691000
1	5.802208000	1.972599000	-0.326910000
1	6.452828000	-0.629507000	-0.512890000
1	2.806345000	-5.074054000	-0.082271000
1	0.153280000	-4.907890000	0.332512000
1	-0.670963000	5.171359000	-0.731711000
1	3.677697000	3.284595000	-0.200217000
1	4.825851000	-3.018766000	-0.423218000
1	-1.521012000	-3.078165000	0.405309000
1	-2.709305000	3.205954000	-0.253212000
7	-3.799822000	-2.096404000	-0.084485000
8	-4.710437000	-2.194776000	0.730081000
8	-3.520978000	-2.911892000	-0.963908000
7	-4.588731000	0.886887000	-0.501473000
8	-4.806651000	2.093618000	-0.346353000
8	-5.381440000	0.079571000	-0.985931000

E= –1723.0776163 a.u.
H= –1723.0776163 + 0.326225 (thermal correction 6-31+G**) a. u.
G= –1723.0776163 + 0.247682 (thermal correction 6-31+G**) a. u.

2f – akamptisomer *parvo,amplo* (***t***$_2$)

9	-2.526261000	-1.016031000	2.202072000
5	-0.303136000	0.732245000	-0.066866000
5	-1.983926000	-0.591780000	0.982231000
8	-0.897925000	0.244920000	1.106753000
9	-0.846353000	0.287301000	-1.298457000
6	-1.114164000	4.401237000	0.046375000
6	-1.512761000	3.002020000	0.043009000
6	-2.858269000	2.584017000	0.060512000
6	-3.563998000	1.363868000	-0.000936000
6	-4.962339000	1.340462000	-0.416580000

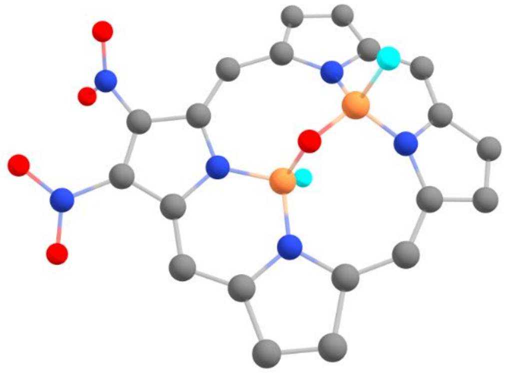

6	-5.341026000	0.038619000	-0.573687000
6	-4.193882000	-0.768190000	-0.233854000
6	-3.981785000	-2.142940000	-0.369698000
6	-2.689119000	-2.683756000	-0.248094000
6	-2.182610000	-3.984488000	-0.607186000
6	-0.826466000	-3.931132000	-0.449793000
6	-0.468966000	-2.584624000	-0.021933000
6	0.878532000	-2.195161000	0.027883000
6	1.615459000	-0.986082000	0.034266000
6	3.050990000	-0.919775000	0.021244000
6	3.432926000	0.406721000	-0.058752000
6	2.234770000	1.191443000	-0.101762000
6	2.020196000	2.584229000	-0.126889000
6	0.725244000	3.096060000	-0.085768000
6	0.249059000	4.458947000	-0.031521000
7	-0.365963000	2.267380000	-0.050426000
7	-3.170613000	0.061249000	0.153291000
7	-1.646669000	-1.893536000	0.143208000
7	1.189828000	0.315190000	-0.055551000
1	-1.805502000	5.231021000	0.107983000
1	-5.547108000	2.226029000	-0.627359000
1	-6.290677000	-0.340769000	-0.925278000
1	-2.778466000	-4.811794000	-0.967308000
1	-0.106547000	-4.708813000	-0.667763000
1	0.878873000	5.337711000	-0.041643000
1	-3.534918000	3.433992000	0.016115000
1	-4.798306000	-2.780612000	-0.687630000
1	1.534460000	-3.058731000	-0.020961000
1	2.873371000	3.246554000	-0.167677000
7	3.918947000	-2.085083000	-0.010478000
8	3.762381000	-2.916795000	0.884543000
8	4.703696000	-2.173482000	-0.948669000
7	4.776105000	0.922282000	-0.007271000
8	5.652063000	0.181992000	0.436873000
8	4.952467000	2.080919000	-0.401783000

E= –1723.0772091 a.u.
H= –1723.0772091 + 0.326196 (thermal correction 6-31+G**) a. u.
G= –1723.0772091 + 0.247707 (thermal correction 6-31+G**) a. u.

2f – transition state (*TS*)

9	1.719848000	-0.463999000	-2.104015000
5	0.167191000	0.882626000	0.800686000
5	1.880080000	-0.509361000	-0.704729000
8	1.001238000	0.160009000	0.051632000
9	0.321676000	0.855202000	2.200417000
6	1.032186000	4.348935000	-0.327790000

6	1.430913000	2.974655000	-0.031622000
6	2.792144000	2.537829000	-0.098969000
6	3.582695000	1.353288000	-0.127692000
6	5.029776000	1.370639000	0.085470000
6	5.489238000	0.080718000	0.092579000
6	4.343897000	-0.777690000	-0.141934000
6	4.167641000	-2.172848000	-0.058536000
6	2.871529000	-2.746975000	-0.015340000
6	2.392315000	-4.055496000	0.381034000
6	1.026069000	-3.973368000	0.455488000
6	0.619857000	-2.606200000	0.137723000
6	-0.726369000	-2.174683000	0.241100000
6	-1.534276000	-0.982983000	0.236369000
6	-2.963295000	-0.993347000	0.037397000
6	-3.432526000	0.312378000	0.032298000
6	-2.304285000	1.169835000	0.259488000
6	-2.111342000	2.575422000	0.165940000
6	-0.821294000	3.125153000	0.118557000
6	-0.332871000	4.438445000	-0.249672000
7	0.272420000	2.323797000	0.284860000
7	3.265059000	0.034366000	-0.319502000
7	1.786149000	-1.959191000	-0.204060000
7	-1.238307000	0.345924000	0.429161000
1	1.719315000	5.133379000	-0.616321000
1	5.607483000	2.267555000	0.266270000
1	6.499764000	-0.256368000	0.278754000
1	3.012786000	-4.910820000	0.610551000
1	0.342529000	-4.757323000	0.753923000
1	-0.950048000	5.300311000	-0.463048000
1	3.445266000	3.408074000	-0.143310000
1	5.031949000	-2.806933000	0.100709000
1	-1.368166000	-3.047195000	0.335651000
1	-2.979086000	3.207051000	0.032645000
7	-3.754898000	-2.205156000	-0.099150000
8	-4.646252000	-2.384005000	0.723911000
8	-3.430771000	-2.981631000	-0.998337000
7	-4.766628000	0.749262000	-0.281907000
8	-5.045963000	1.926458000	-0.024254000
8	-5.532058000	-0.067099000	-0.792999000

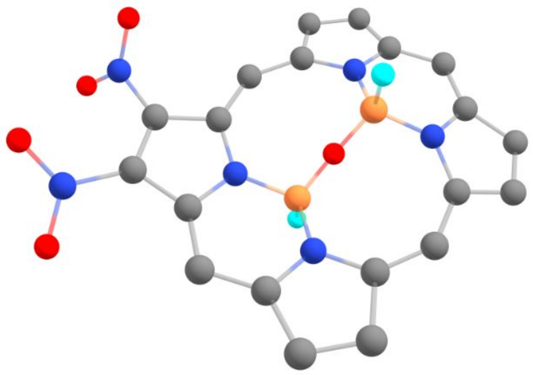

E= –1723.0330964 a.u.
H= –1723.0330964 + 0.324574 (thermal correction 6-31+G**) a. u.
G= –1723.0330964 + 0.246420 (thermal correction 6-31+G**) a. u.
f = 184.6949 i

2f– akamptisomer *amplo, amplo* (c_1)

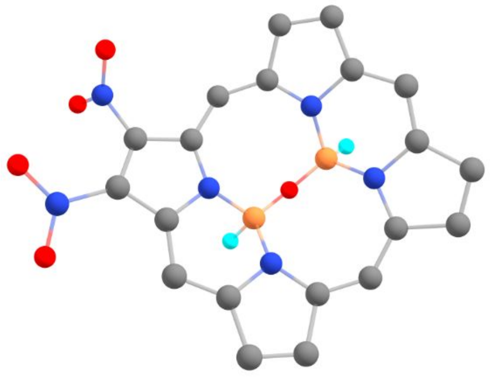

9	2.877427000	-1.326514000	1.858699000
5	0.147571000	0.977459000	1.071959000
5	2.077193000	-0.719572000	0.868419000
8	1.143453000	0.113909000	1.426062000
9	-0.450044000	1.574961000	2.197041000
6	1.400340000	4.254749000	-0.327988000
6	1.726510000	2.889882000	0.045301000
6	3.059808000	2.451635000	0.064850000
6	3.657865000	1.203561000	-0.152099000
6	4.999176000	1.104638000	-0.701925000
6	5.202336000	-0.190884000	-1.083829000
6	4.010826000	-0.917098000	-0.734731000
6	3.691933000	-2.259298000	-0.930282000
6	2.422885000	-2.744640000	-0.587988000
6	1.853665000	-4.050566000	-0.770537000
6	0.578817000	-3.995789000	-0.278555000
6	0.332142000	-2.636866000	0.166692000
6	-0.964556000	-2.203638000	0.443479000
6	-1.602850000	-0.951311000	0.346527000
6	-3.008050000	-0.844159000	0.070819000
6	-3.294389000	0.468507000	-0.239884000
6	-2.071704000	1.201213000	-0.121453000
6	-1.783499000	2.554662000	-0.356904000
6	-0.475902000	3.015416000	-0.265556000
6	0.053055000	4.323043000	-0.542040000
7	0.558210000	2.178368000	0.113566000
7	3.108468000	-0.050933000	-0.148809000
7	1.493339000	-1.920359000	0.000447000
7	-1.087009000	0.320078000	0.263396000
1	2.133897000	5.035637000	-0.478217000
1	5.661990000	1.947722000	-0.844897000
1	6.067339000	-0.612560000	-1.577288000
1	2.352475000	-4.890372000	-1.234506000
1	-0.169415000	-4.777185000	-0.289806000
1	-0.528203000	5.170076000	-0.879684000
1	3.770118000	3.272081000	0.011883000
1	4.415259000	-2.923439000	-1.388332000
1	-1.663847000	-3.029106000	0.518601000
1	-2.583414000	3.223547000	-0.640351000
7	-3.936905000	-1.966095000	0.092771000
8	-4.803994000	-1.949486000	0.956921000
8	-3.746059000	-2.861168000	-0.730843000
7	-4.560345000	0.976431000	-0.702511000
8	-5.417486000	0.151158000	-1.018879000
8	-4.699317000	2.202757000	-0.757803000

E= –1723.0663237 a.u.

H= –1723.0663237 + 0.325831 (thermal correction 6-31+G**) a. u.
G= –1723.0663237 + 0.246531 (thermal correction 6-31+G**) a. u.

2g – akamptisomer *amplo,parvo* (***t***$_1$)

9	0.504743000	0.005168000	-1.263147000
5	-0.644987000	0.702337000	1.069819000
5	1.198872000	-0.300758000	-0.061040000
8	0.573536000	0.072146000	1.143972000
9	-1.203192000	1.029896000	2.314987000
6	-0.208422000	4.178469000	-0.429266000
6	0.414948000	2.930846000	-0.016989000
6	1.820698000	2.793445000	-0.031941000
6	2.762444000	1.747139000	-0.077503000
6	4.195092000	1.963439000	-0.155629000
6	4.810212000	0.741571000	-0.236800000
6	3.768916000	-0.254871000	-0.215623000
6	3.825485000	-1.656287000	-0.231786000
6	2.657081000	-2.423337000	-0.125936000
6	2.455817000	-3.848893000	-0.049420000
6	1.108412000	-4.055324000	0.089604000
6	0.450773000	-2.762149000	0.107299000
6	-0.945983000	-2.602200000	0.190169000
6	-1.859644000	-1.532231000	0.167931000
6	-3.254262000	-1.763282000	-0.163827000
6	-3.879756000	-0.559605000	-0.297982000
6	-2.903555000	0.456068000	-0.028119000
6	-2.968752000	1.844707000	-0.173400000
6	-1.797407000	2.615189000	-0.118820000
6	-1.560167000	3.980266000	-0.506291000
7	-0.599988000	2.036501000	0.215640000
7	2.569146000	0.393361000	-0.130003000
7	1.432888000	-1.821067000	-0.037155000
7	-1.726382000	-0.170668000	0.294950000
1	0.340337000	5.072391000	-0.694805000
1	4.666936000	2.936974000	-0.142467000
1	5.870101000	0.535699000	-0.297155000
1	3.239647000	-4.592803000	-0.089231000
1	0.593630000	-5.002615000	0.179697000
1	-2.317277000	4.678779000	-0.835391000
1	2.307143000	3.761887000	-0.125084000
1	4.786702000	-2.152027000	-0.298657000
1	-1.462031000	-3.559232000	0.172959000
1	-3.909625000	2.309251000	-0.443069000
9	-3.776993000	-2.974013000	-0.396097000
9	-5.141723000	-0.327496000	-0.678021000

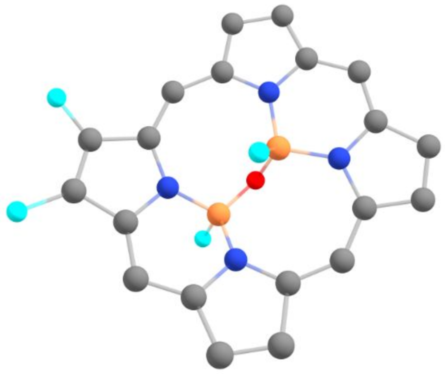

E= –1512.4530043 a.u.

H= –1512.4530043 + 0.301926 (thermal correction 6-31+G**) a. u.
G= –1512.4530043 + 0.232726 (thermal correction 6-31+G**) a. u.

2g – akamptisomer *parvo,amplo* (***t*₂**)

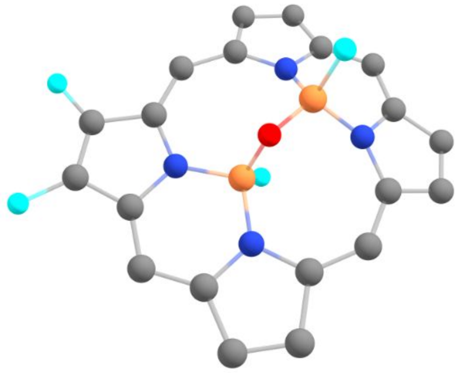

9	-2.045713000	-0.734131000	2.216675000
5	0.451178000	0.563914000	-0.073950000
5	-1.439657000	-0.420627000	0.989672000
8	-0.221808000	0.208185000	1.107854000
9	-0.187981000	0.235944000	-1.299819000
6	0.354845000	4.316027000	0.014313000
6	-0.301437000	3.021922000	0.025025000
6	-1.698915000	2.858890000	0.051405000
6	-2.625926000	1.792197000	0.000430000
6	-3.998323000	2.029740000	-0.417748000
6	-4.618673000	0.818954000	-0.566417000
6	-3.648454000	-0.184683000	-0.217706000
6	-3.695848000	-1.580660000	-0.343837000
6	-2.533685000	-2.353992000	-0.219232000
6	-2.282446000	-3.727439000	-0.567947000
6	-0.936891000	-3.928169000	-0.420589000
6	-0.331785000	-2.673713000	-0.002898000
6	1.074718000	-2.538408000	0.047590000
6	2.002029000	-1.487420000	0.021867000
6	3.436177000	-1.691674000	0.008732000
6	4.051921000	-0.476408000	-0.077471000
6	3.023274000	0.526431000	-0.122138000
6	3.091092000	1.922186000	-0.160845000
6	1.914677000	2.686160000	-0.115014000
6	1.707359000	4.111736000	-0.068232000
7	0.690184000	2.081755000	-0.066318000
7	-2.487803000	0.437400000	0.163730000
7	-1.351146000	-1.772060000	0.161472000
7	1.825370000	-0.131495000	-0.072591000
1	-0.163837000	5.263856000	0.070520000
1	-4.405373000	3.007893000	-0.637541000
1	-5.622686000	0.625134000	-0.918457000
1	-3.024911000	-4.430811000	-0.919443000
1	-0.377763000	-4.828123000	-0.640000000
1	2.490736000	4.856867000	-0.087341000
1	-2.206018000	3.820052000	0.004903000
1	-4.619218000	-2.054936000	-0.656008000
1	1.573679000	-3.503715000	0.002475000
1	4.056646000	2.411464000	-0.196604000
9	4.021329000	-2.893224000	0.076407000
9	5.365380000	-0.220684000	-0.102566000

E= –1512.4523406 a.u.
H= –1512.4523406 + 0.301912 (thermal correction 6-31+G**) a. u.
G= –1512.4523406 + 0.232702 (thermal correction 6-31+G**) a. u.

2g – transition state (*TS*)

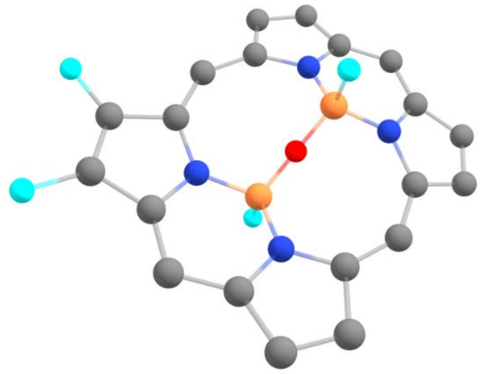

9	1.250395000	-0.315226000	-2.112101000
5	-0.636046000	0.633536000	0.759539000
5	1.378559000	-0.333395000	-0.704745000
8	0.367165000	0.147061000	0.025098000
9	-0.507464000	0.612625000	2.166380000
6	-0.516419000	4.218815000	-0.312162000
6	0.163895000	2.964593000	-0.012700000
6	1.584598000	2.828264000	-0.059225000
6	2.621461000	1.843305000	-0.072643000
6	4.018633000	2.170536000	0.183622000
6	4.749560000	1.007497000	0.198501000
6	3.828189000	-0.071954000	-0.077350000
6	3.948034000	-1.479286000	-0.008304000
6	2.810510000	-2.317493000	-0.012589000
6	2.612405000	-3.706855000	0.336367000
6	1.256324000	-3.922940000	0.364505000
6	0.576347000	-2.669860000	0.061222000
6	-0.846558000	-2.535501000	0.102486000
6	-1.867606000	-1.545427000	0.124057000
6	-3.268702000	-1.859529000	-0.109901000
6	-3.999335000	-0.701626000	-0.107937000
6	-3.089534000	0.384733000	0.152701000
6	-3.216733000	1.786624000	0.084566000
6	-2.072473000	2.618656000	0.077539000
6	-1.872836000	4.007005000	-0.273880000
7	-0.841151000	2.074320000	0.272384000
7	2.605564000	0.488527000	-0.282114000
7	1.577841000	-1.776916000	-0.216527000
7	-1.867089000	-0.187662000	0.336410000
1	-0.011149000	5.138037000	-0.578329000
1	4.383883000	3.168548000	0.387785000
1	5.803058000	0.897210000	0.416737000
1	3.394730000	-4.415747000	0.571064000
1	0.750466000	-4.843322000	0.625311000
1	-2.655070000	4.717602000	-0.503497000
1	2.035860000	3.819209000	-0.095282000
1	4.923913000	-1.916722000	0.169155000
1	-1.306329000	-3.522720000	0.124195000
1	-4.195494000	2.221302000	-0.079654000
9	-3.728389000	-3.090471000	-0.365530000
9	-5.305483000	-0.567575000	-0.368367000

E= –1512.4078573 a.u.
H= –1512.4078573 + 0.300222 (thermal correction 6-31+G**) a. u.
G= –1512.4078573 + 0.231203 (thermal correction 6-31+G**) a. u.
f = 185.5909 i

2g– akamptisomer *amplo, amplo* (c_1)

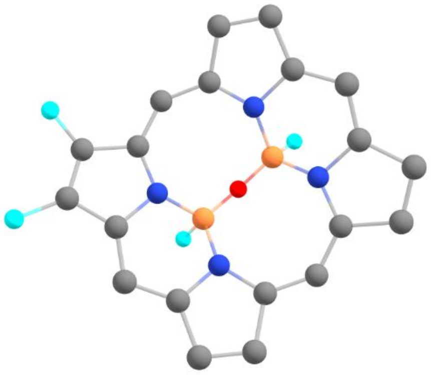

9	2.391214000	-0.943242000	1.924007000
5	-0.703694000	0.767807000	0.979391000
5	1.534810000	-0.503296000	0.890175000
8	0.435755000	0.137633000	1.397649000
9	-1.472134000	1.219857000	2.074574000
6	-0.072650000	4.242503000	-0.380785000
6	0.501830000	2.975359000	0.024125000
6	1.891472000	2.806423000	0.100867000
6	2.740712000	1.702852000	-0.072840000
6	4.093338000	1.878292000	-0.561324000
6	4.576413000	0.647835000	-0.917756000
6	3.546229000	-0.302705000	-0.614288000
6	3.514568000	-1.686339000	-0.809000000
6	2.359736000	-2.418454000	-0.526015000
6	2.074210000	-3.808997000	-0.734568000
6	0.784149000	-4.011870000	-0.324189000
6	0.247181000	-2.736158000	0.103688000
6	-1.130316000	-2.568406000	0.312293000
6	-1.977148000	-1.464081000	0.195521000
6	-3.377172000	-1.631819000	-0.124911000
6	-3.889691000	-0.415688000	-0.469357000
6	-2.839547000	0.543522000	-0.323612000
6	-2.843106000	1.918086000	-0.548411000
6	-1.663024000	2.652119000	-0.395352000
6	-1.396341000	4.035834000	-0.662680000
7	-0.505951000	2.043966000	0.045786000
7	2.460094000	0.360072000	-0.080822000
7	1.245818000	-1.801916000	0.007839000
7	-1.704367000	-0.119972000	0.100603000
1	0.494382000	5.155246000	-0.507512000
1	4.575991000	2.838573000	-0.686473000
1	5.531893000	0.411749000	-1.366100000
1	2.757892000	-4.529573000	-1.162418000
1	0.210233000	-4.927647000	-0.374928000
1	-2.116211000	4.748641000	-1.041185000
1	2.427108000	3.751574000	0.068409000
1	4.379601000	-2.189045000	-1.225615000
1	-1.673814000	-3.508297000	0.354038000
1	-3.751279000	2.406855000	-0.879563000
9	-3.995497000	-2.816845000	-0.185325000

9	-5.122651000	-0.138281000	-0.907696000

$E = -1512.4426991$ a.u.
$H = -1512.4426991 + 0.301539$ (thermal correction 6-31+G**) a. u.
$G = -1512.4426991 + 0.231715$ (thermal correction 6-31+G**) a. u.

STRUCTURAL VARIATION 3

3a – akamptisomer *amplo,parvo* (***t*$_1$**)

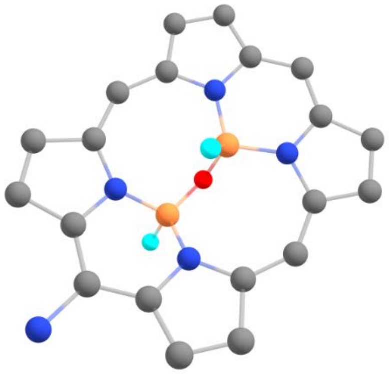

9	0.404837000	0.000026000	-1.291434000
5	-0.966154000	0.000028000	1.021808000
5	1.154107000	-0.000014000	-0.080125000
8	0.403505000	0.000036000	1.114636000
9	-1.630380000	-0.000076000	2.263685000
6	-2.129090000	3.338672000	-0.382022000
6	-1.026523000	2.501778000	0.024013000
6	0.298481000	3.026342000	0.070441000
6	1.604560000	2.534567000	0.013925000
6	2.784900000	3.383022000	-0.011470000
6	3.888903000	2.582209000	-0.117040000
6	3.418860000	1.216280000	-0.160156000
6	4.115455000	-0.000096000	-0.216050000
6	3.418779000	-1.216450000	-0.160258000
6	3.888730000	-2.582404000	-0.117382000
6	2.784672000	-3.383162000	-0.011860000
6	1.604405000	-2.534638000	0.013769000
6	0.298291000	-3.026337000	0.070350000
6	-1.026681000	-2.501737000	0.024068000
6	-2.129371000	-3.338620000	-0.381615000
6	-3.252904000	-2.550637000	-0.493775000
6	-2.855117000	-1.228412000	-0.133577000
6	-3.566108000	0.000110000	-0.196740000
6	-2.855014000	1.228543000	-0.133694000
6	-3.252648000	2.550734000	-0.494259000
7	-1.525959000	1.238831000	0.203851000
7	2.057345000	1.240672000	-0.090951000
7	2.057270000	-1.240751000	-0.090968000
7	-1.526017000	-1.238703000	0.203712000
1	-2.042206000	4.389155000	-0.626058000
1	2.759905000	4.463186000	0.049047000
1	4.926568000	2.884746000	-0.153656000
1	4.926368000	-2.885019000	-0.154113000
1	2.759621000	-4.463333000	0.048510000
1	-4.226440000	2.857418000	-0.851767000
1	0.284002000	4.113919000	0.049004000
1	5.197364000	-0.000130000	-0.267092000
1	0.283768000	-4.113914000	0.048860000

1	-2.042636000	-4.389153000	-0.625497000
1	-4.226799000	-2.857430000	-0.850912000
7	-4.913276000	0.000201000	-0.470179000
1	-5.429259000	-0.846405000	-0.285485000
1	-5.429175000	0.846730000	-0.284872000

E= –1369.2868923 a.u.
H= –1369.2868923 + 0.334462 (thermal correction 6-31+G**) a. u.
G= –1369.2868923 + 0.266459 (thermal correction 6-31+G**) a. u.

3a – akamptisomer *parvo,amplo* (***t*$_2$**)

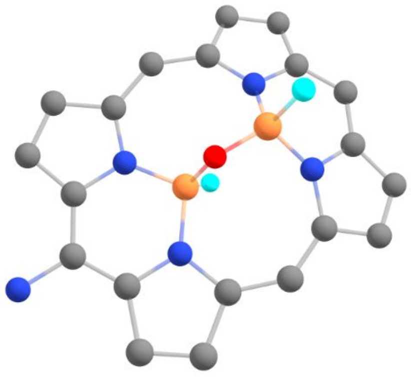

9	-2.094397000	-0.000199000	2.215381000
5	0.734348000	0.000030000	-0.060495000
5	-1.414625000	-0.000013000	0.983233000
8	-0.042287000	0.000038000	1.109438000
9	0.036468000	0.000142000	-1.298305000
6	2.347972000	3.381276000	0.036562000
6	1.186754000	2.529294000	0.036531000
6	-0.144785000	3.025674000	0.036988000
6	-1.446727000	2.509199000	-0.006303000
6	-2.563500000	3.349399000	-0.421973000
6	-3.666155000	2.558088000	-0.574223000
6	-3.263860000	1.216373000	-0.228178000
6	-3.951398000	0.000091000	-0.351357000
6	-3.263858000	-1.216220000	-0.228285000
6	-3.666315000	-2.557965000	-0.574008000
6	-2.563655000	-3.349316000	-0.421940000
6	-1.446832000	-2.509194000	-0.006294000
6	-0.144914000	-3.025683000	0.037026000
6	1.186627000	-2.529312000	0.036548000
6	2.347803000	-3.381325000	0.036487000
6	3.468727000	-2.582581000	-0.030825000
6	3.009358000	-1.229094000	-0.080303000
6	3.717344000	-0.000057000	-0.128517000
6	3.009466000	1.228999000	-0.080013000
6	3.468870000	2.582483000	-0.030556000
7	1.641066000	1.244278000	-0.042451000
7	-1.951329000	1.237141000	0.156702000
7	-1.951300000	-1.237027000	0.156445000
7	1.640975000	-1.244299000	-0.042565000
1	2.318554000	4.461325000	0.091124000
1	-2.477503000	4.405991000	-0.640007000
1	-4.647594000	2.845278000	-0.926463000
1	-4.647819000	-2.845150000	-0.926071000
1	-2.477759000	-4.405923000	-0.639951000
1	4.498360000	2.914409000	-0.022614000
1	-0.149867000	4.111059000	-0.035530000

1	-4.989692000	0.000103000	-0.661170000
1	-0.149991000	-4.111079000	-0.035341000
1	2.318381000	-4.461369000	0.091182000
1	4.498205000	-2.914548000	-0.023138000
7	5.095219000	-0.000154000	-0.152145000
1	5.559963000	0.845557000	-0.445387000
1	5.559830000	-0.845860000	-0.445620000

E= –1369.2847673 a.u.
H= –1369.2847673 + 0.334386 (thermal correction 6-31+G**) a. u.
G= –1369.2847673 + 0.266262 (thermal correction 6-31+G**) a. u.

3a – transition state (*TS*)

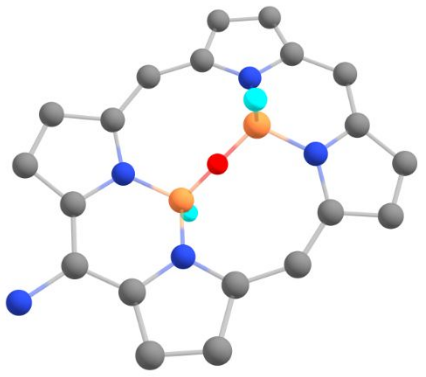

9	1.238840000	0.000005000	-2.134111000
5	-0.913033000	0.000000000	0.702801000
5	1.349237000	0.000001000	-0.722086000
8	0.211903000	-0.000005000	-0.013391000
9	-0.817226000	0.000003000	2.115303000
6	-2.258136000	3.341759000	-0.282180000
6	-1.141334000	2.474082000	0.010643000
6	0.228502000	2.940327000	0.036907000
6	1.563071000	2.477621000	-0.000698000
6	2.703453000	3.342512000	0.299633000
6	3.846567000	2.587713000	0.297329000
6	3.458135000	1.228989000	-0.030631000
6	4.156876000	0.000000000	0.005520000
6	3.458137000	-1.228990000	-0.030638000
6	3.846568000	-2.587714000	0.297322000
6	2.703453000	-3.342513000	0.299629000
6	1.563071000	-2.477620000	-0.000700000
6	0.228502000	-2.940326000	0.036907000
6	-1.141335000	-2.474083000	0.010648000
6	-2.258137000	-3.341760000	-0.282175000
6	-3.418044000	-2.587169000	-0.286213000
6	-3.036328000	-1.245023000	0.034769000
6	-3.745574000	0.000000000	0.022658000
6	-3.036327000	1.245022000	0.034767000
6	-3.418044000	2.587168000	-0.286217000
7	-1.689316000	1.242532000	0.236685000
7	2.120427000	1.240476000	-0.255645000
7	2.120428000	-1.240477000	-0.255650000
7	-1.689318000	-1.242533000	0.236689000
1	-2.174000000	4.394196000	-0.519373000
1	2.620697000	4.394035000	0.542859000
1	4.849652000	2.913942000	0.536726000
1	4.849653000	-2.913946000	0.536716000
1	2.620697000	-4.394035000	0.542855000

1	-4.409118000	2.935727000	-0.544676000
1	0.225755000	4.027923000	0.101589000
1	5.224163000	0.000000000	0.191944000
1	0.225755000	-4.027922000	0.101590000
1	-2.174001000	-4.394197000	-0.519366000
1	-4.409118000	-2.935726000	-0.544677000
7	-5.106225000	-0.000001000	-0.140683000
1	-5.615656000	0.851474000	0.036572000
1	-5.615659000	-0.851466000	0.036611000

E= –1369.2422913 a.u.
H= –1369.2422913 + 0.332732 (thermal correction 6-31+G**) a. u.
G= –1369.2422913 + 0.264897 (thermal correction 6-31+G**) a. u.
f = 180.8447 i

3a– akamptisomer *amplo, amplo* (c_1)

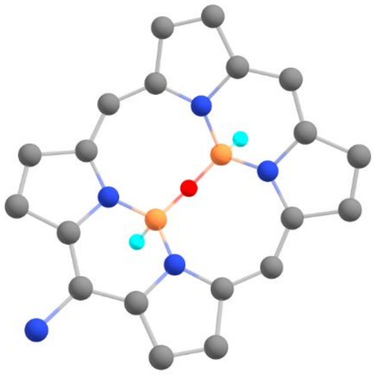

9	-2.472352000	-0.000402000	1.925180000
5	1.059106000	0.000009000	0.968640000
5	-1.526216000	-0.000060000	0.871417000
8	-0.247448000	0.000516000	1.367639000
9	1.938598000	-0.000080000	2.080051000
6	2.188319000	-3.371561000	-0.324833000
6	1.085659000	-2.535885000	0.068937000
6	-0.219982000	-3.070426000	0.170443000
6	-1.497371000	-2.543558000	0.000062000
6	-2.601603000	-3.379199000	-0.436929000
6	-3.630083000	-2.560356000	-0.810513000
6	-3.196862000	-1.210958000	-0.565837000
6	-3.856223000	0.000025000	-0.794277000
6	-3.197032000	1.211103000	-0.565682000
6	-3.630153000	2.560524000	-0.810401000
6	-2.601522000	3.379267000	-0.436973000
6	-1.497408000	2.543534000	0.000167000
6	-0.219936000	3.070300000	0.170399000
6	1.085725000	2.535807000	0.068899000
6	2.188437000	3.371521000	-0.324674000
6	3.262724000	2.556397000	-0.600408000
6	2.835329000	1.222455000	-0.346481000
6	3.533569000	-0.000037000	-0.488730000
6	2.835238000	-1.222462000	-0.346403000
6	3.262653000	-2.556402000	-0.600307000
7	1.520756000	-1.236994000	0.075107000
7	-1.920146000	-1.233955000	-0.050810000
7	-1.920428000	1.234002000	-0.050536000
7	1.520942000	1.236946000	0.075042000
1	2.129155000	-4.444038000	-0.454291000
1	-2.551530000	-4.456947000	-0.521768000

1	-4.584607000	-2.841164000	-1.234678000
1	-4.584642000	2.841432000	-1.234574000
1	-2.551317000	4.457000000	-0.521935000
1	4.225783000	-2.862985000	-0.985803000
1	-0.215012000	-4.157445000	0.169123000
1	-4.865050000	-0.000052000	-1.189237000
1	-0.214930000	4.157327000	0.168991000
1	2.129338000	4.444021000	-0.454010000
1	4.225801000	2.863025000	-0.986009000
7	4.859692000	-0.000027000	-0.865790000
1	5.386660000	0.842678000	-0.692031000
1	5.386686000	-0.842725000	-0.692103000

E= –1369.2754836 a.u.
H= –1369.2754836 + 0.334122 (thermal correction 6-31+G**) a. u.
G= –1369.2754836 + 0.265435 (thermal correction 6-31+G**) a. u.

3b – akamptisomer *amplo,parvo* (***t*$_1$**)

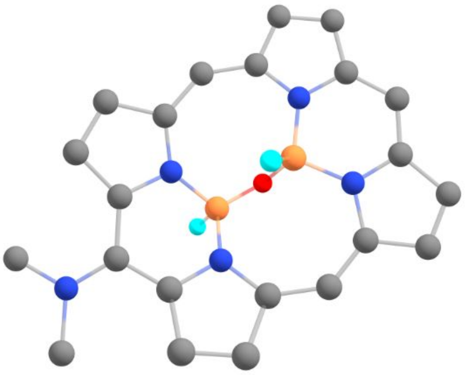

9	0.881949000	-0.000013000	-1.299768000
5	-0.578060000	-0.000033000	0.976065000
5	1.593635000	0.000013000	-0.064136000
8	0.790052000	-0.000009000	1.097334000
9	-1.269092000	-0.000114000	2.206965000
6	-1.648310000	3.282416000	-0.648101000
6	-0.588606000	2.466743000	-0.133191000
6	0.732859000	3.001870000	-0.041189000
6	2.041359000	2.527847000	-0.001409000
6	3.217431000	3.381875000	0.017854000
6	4.327746000	2.583268000	-0.001878000
6	3.865778000	1.213819000	-0.039939000
6	4.568300000	0.000076000	-0.051549000
6	3.865833000	-1.213693000	-0.039880000
6	4.327844000	-2.583123000	-0.001693000
6	3.217552000	-3.381761000	0.018132000
6	2.041453000	-2.527770000	-0.001235000
6	0.732969000	-3.001860000	-0.040877000
6	-0.588544000	-2.466864000	-0.132937000
6	-1.648249000	-3.282843000	-0.647279000
6	-2.796207000	-2.523216000	-0.702088000
6	-2.459613000	-1.231295000	-0.192667000
6	-3.209342000	-0.000084000	-0.100101000
6	-2.459551000	1.231059000	-0.192601000
6	-2.796279000	2.522772000	-0.702382000
7	-1.127998000	1.231643000	0.140967000
7	2.502853000	1.234046000	-0.043991000
7	2.502906000	-1.233965000	-0.043958000
7	-1.127989000	-1.231680000	0.140804000

1	-1.527838000	4.300860000	-0.992557000
1	3.185323000	4.463179000	0.047539000
1	5.365038000	2.889158000	0.011944000
1	5.365145000	-2.888982000	0.012149000
1	3.185481000	-4.463063000	0.047938000
1	-3.737695000	2.832853000	-1.125833000
1	0.708678000	4.087219000	-0.112822000
1	5.651253000	0.000104000	-0.048731000
1	0.708863000	-4.087230000	-0.112213000
7	-4.565297000	0.000172000	0.042919000
6	-5.337005000	-1.235143000	0.153814000
1	-6.265050000	-1.020211000	0.685123000
1	-5.594940000	-1.650452000	-0.828093000
1	-4.777576000	-1.981746000	0.720926000
6	-5.336165000	1.235739000	0.157635000
1	-6.262372000	1.020599000	0.692126000
1	-4.774410000	1.981565000	0.723376000
1	-5.597539000	1.652051000	-0.822959000
1	-1.527746000	-4.301437000	-0.991276000
1	-3.737475000	-2.833662000	-1.125542000

E= –1447.927069 a.u.
H= –1447.927069 + 0.394083 (thermal correction 6-31+G**) a. u.
G= –1447.927069 + 0.319127 (thermal correction 6-31+G**) a. u.

3b – akamptisomer *parvo,amplo* (***t*$_2$**)

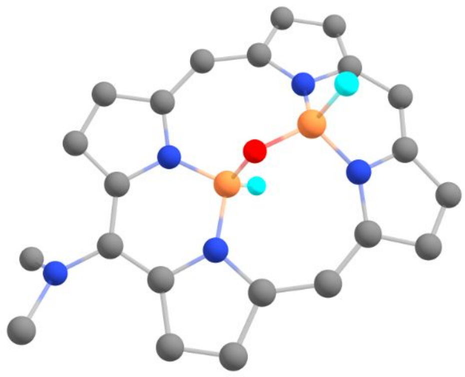

9	-2.565257000	-0.005767000	2.199643000
5	0.303521000	0.008298000	-0.031177000
5	-1.867969000	-0.001337000	0.976438000
8	-0.497968000	-0.010891000	1.123067000
9	-0.375460000	0.055029000	-1.279950000
6	1.907876000	3.384446000	-0.021595000
6	0.755200000	2.525094000	0.052293000
6	-0.574788000	3.018109000	0.028790000
6	-1.880347000	2.506529000	-0.019315000
6	-2.985548000	3.348838000	-0.459248000
6	-4.090887000	2.562320000	-0.619118000
6	-3.699938000	1.222013000	-0.257940000
6	-4.387930000	0.007948000	-0.394906000
6	-3.705453000	-1.210462000	-0.264039000
6	-4.105209000	-2.552566000	-0.606923000
6	-3.006707000	-3.346647000	-0.429340000
6	-1.896742000	-2.505729000	-0.003133000
6	-0.594810000	-3.020765000	0.084191000
6	0.732981000	-2.524044000	0.097938000
6	1.888751000	-3.375891000	0.206822000
6	3.012484000	-2.583634000	0.161500000

6	2.566168000	-1.230570000	-0.000304000
6	3.300339000	-0.008562000	-0.054511000
6	2.591567000	1.228180000	-0.030036000
6	3.032295000	2.594461000	-0.076678000
7	1.221944000	1.240422000	0.042036000
7	-2.392917000	1.240213000	0.149046000
7	-2.397385000	-1.231707000	0.136814000
7	1.197509000	-1.243422000	-0.036451000
1	1.869702000	4.465604000	-0.036641000
1	-2.890347000	4.402810000	-0.685853000
1	-5.065510000	2.851269000	-0.988435000
1	-5.081662000	-2.840590000	-0.972162000
1	-2.920544000	-4.405428000	-0.636334000
1	4.051512000	2.938657000	-0.149005000
1	-0.577433000	4.100948000	-0.074801000
1	-5.421439000	0.011565000	-0.720547000
1	-0.598627000	-4.108279000	0.051959000
1	4.038250000	-2.906126000	0.256419000
1	1.850877000	-4.450555000	0.326099000
7	4.684943000	-0.056760000	-0.103017000
6	5.535270000	0.893141000	0.594243000
1	6.386530000	0.352861000	1.027738000
1	5.939962000	1.669384000	-0.073489000
1	4.985953000	1.370453000	1.406426000
6	5.395558000	-0.934781000	-1.024709000
1	6.049156000	-0.334304000	-1.674253000
1	6.025476000	-1.660290000	-0.491528000
1	4.691141000	-1.474808000	-1.656553000

E= –1447.9263085 a.u.
H= –1447.9263085 + 0.393461 (thermal correction 6-31+G**) a. u.
G= –1447.9263085 + 0.318854 (thermal correction 6-31+G**) a. u.

3b – transition state (*TS*)

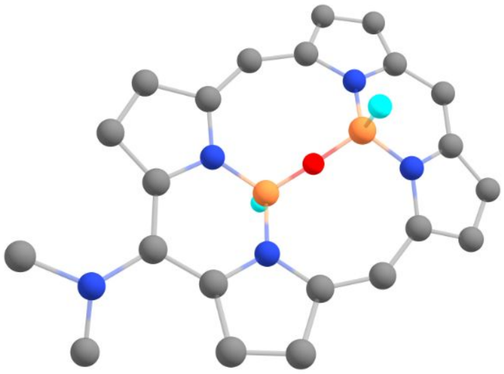

9	1.716598000	-0.005482000	-2.137917000
5	-0.486773000	-0.012927000	0.659356000
5	1.802850000	0.001884000	-0.723354000
8	0.652097000	0.025103000	-0.035894000
9	-0.411208000	-0.091771000	2.073054000
6	-1.794336000	3.320392000	-0.394075000
6	-0.692642000	2.465175000	-0.029528000
6	0.675116000	2.934088000	-0.001578000
6	2.012649000	2.478422000	-0.014317000
6	3.147281000	3.347659000	0.290079000
6	4.291339000	2.592915000	0.310333000
6	3.907862000	1.231693000	-0.010602000

6	4.603277000	0.000713000	0.042340000
6	3.900898000	-1.224801000	0.004284000
6	4.282188000	-2.580908000	0.349559000
6	3.137542000	-3.333105000	0.345702000
6	2.004066000	-2.469548000	0.018157000
6	0.668836000	-2.929305000	0.043401000
6	-0.700263000	-2.469899000	-0.015526000
6	-1.807915000	-3.368688000	-0.208761000
6	-2.979821000	-2.637087000	-0.216134000
6	-2.621454000	-1.257822000	-0.009862000
6	-3.335072000	-0.000777000	0.011192000
6	-2.594991000	1.241039000	0.005556000
6	-2.954793000	2.569264000	-0.399340000
7	-1.255660000	1.245603000	0.244934000
7	2.573531000	1.240127000	-0.250400000
7	2.564895000	-1.236326000	-0.239535000
7	-1.261315000	-1.229642000	0.126397000
1	-1.697989000	4.359536000	-0.680227000
1	3.060887000	4.401413000	0.522217000
1	5.290985000	2.922162000	0.559851000
1	5.281608000	-2.906347000	0.604914000
1	3.048867000	-4.381865000	0.598555000
1	-3.934831000	2.902896000	-0.706762000
1	0.667238000	4.023439000	0.030741000
1	5.668000000	-0.002086000	0.242921000
1	0.665272000	-4.013729000	0.146177000
1	-1.718378000	-4.435822000	-0.365127000
1	-3.963603000	-3.045480000	-0.376451000
7	-4.706959000	0.071123000	-0.045306000
6	-5.458411000	1.087413000	0.691062000
1	-6.004471000	1.754857000	0.012687000
1	-6.188202000	0.589823000	1.343361000
1	-4.790939000	1.681013000	1.313699000
6	-5.532734000	-0.993346000	-0.591599000
1	-6.475296000	-0.556549000	-0.934446000
1	-5.041685000	-1.453456000	-1.450382000
1	-5.766303000	-1.767238000	0.154503000

E= –1447.8853917 a.u.
H= –1447.8853917 + 0.392207 (thermal correction 6-31+G**) a. u.
G= –1447.8853917 + 0.318650 (thermal correction 6-31+G**) a. u.
f = 177.4102 i

3b– akamptisomer *amplo, amplo* (c_1)

9	-2.965780000	-0.000830000	1.870767000
5	0.600207000	0.003249000	1.041736000

5	-1.983000000	0.001518000	0.850602000
8	-0.722363000	0.002950000	1.389475000
9	1.433009000	-0.041374000	2.189530000
6	1.789608000	-3.371134000	-0.067202000
6	0.659248000	-2.518551000	0.180653000
6	-0.644104000	-3.055147000	0.239469000
6	-1.916643000	-2.538160000	-0.003857000
6	-2.997869000	-3.383326000	-0.476050000
6	-4.016021000	-2.572181000	-0.894872000
6	-3.599584000	-1.219256000	-0.643611000
6	-4.253739000	-0.012459000	-0.898700000
6	-3.608148000	1.203428000	-0.649288000
6	-4.032255000	2.548046000	-0.924508000
6	-3.016498000	3.371887000	-0.523611000
6	-1.932327000	2.542065000	-0.033839000
6	-0.658893000	3.072350000	0.173169000
6	0.649198000	2.544179000	0.137023000
6	1.751597000	3.372973000	-0.281947000
6	2.828827000	2.555181000	-0.522021000
6	2.414517000	1.227354000	-0.201221000
6	3.141259000	0.010820000	-0.315572000
6	2.441169000	-1.216705000	-0.191074000
6	2.880480000	-2.573397000	-0.309674000
7	1.090276000	-1.216247000	0.129057000
7	-2.340546000	-1.233704000	-0.083097000
7	-2.352371000	1.233089000	-0.085227000
7	1.101398000	1.251703000	0.208619000
1	1.745964000	-4.451248000	-0.115272000
1	-2.939723000	-4.461480000	-0.549240000
1	-4.952476000	-2.861619000	-1.352272000
1	-4.970129000	2.823846000	-1.387296000
1	-2.962583000	4.448427000	-0.620242000
1	3.872914000	-2.911462000	-0.558498000
1	-0.636583000	-4.141460000	0.278320000
1	-5.246913000	-0.016842000	-1.331762000
1	-0.655921000	4.158997000	0.140498000
1	1.680850000	4.437299000	-0.463110000
1	3.793215000	2.831086000	-0.922626000
7	4.500973000	0.105726000	-0.600212000
6	5.381269000	0.835810000	0.313507000
1	5.973325000	0.130096000	0.917186000
1	6.074712000	1.475026000	-0.246681000
1	4.797705000	1.455422000	0.992854000
6	5.196114000	-0.864854000	-1.426495000
1	6.006991000	-0.352143000	-1.957602000
1	5.649168000	-1.681963000	-0.842051000
1	4.515964000	-1.287077000	-2.168027000

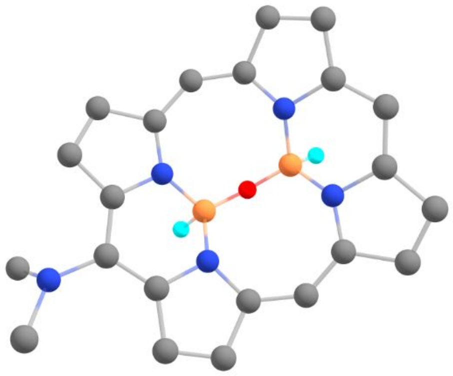

E= –1447.9165271 a.u.

H= –1447.9165271 + 0.393089 (thermal correction 6-31+G**) a. u.

G= –1447.9165271 + 0.318128 (thermal correction 6-31+G**) a. u.

3c – akamptisomer *amplo,parvo* (***t***$_1$)

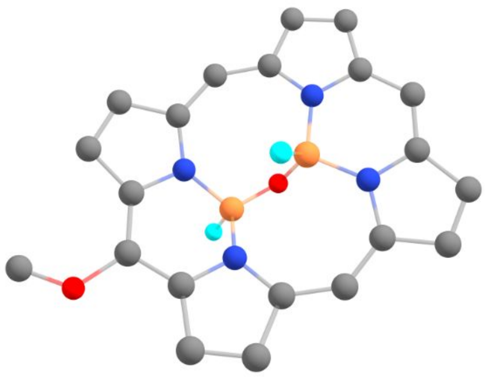

9	0.623139000	-0.012215000	-1.290696000
5	-0.736688000	0.082891000	1.031743000
5	1.374722000	-0.046501000	-0.081990000
8	0.630954000	-0.010825000	1.115247000
9	-1.387965000	0.140095000	2.278553000
6	-1.724010000	3.480526000	-0.350671000
6	-0.654533000	2.582781000	0.026448000
6	0.692307000	3.030779000	0.056072000
6	1.970780000	2.462074000	-0.001510000
6	3.195315000	3.241047000	-0.036687000
6	4.252145000	2.376787000	-0.141593000
6	3.704184000	1.042223000	-0.175377000
6	4.331488000	-0.212957000	-0.229876000
6	3.570766000	-1.387648000	-0.168152000
6	3.959507000	-2.778779000	-0.124018000
6	2.810290000	-3.512657000	-0.012276000
6	1.682798000	-2.594466000	0.017729000
6	0.347269000	-3.005656000	0.080082000
6	-0.945170000	-2.407725000	0.043371000
6	-2.087316000	-3.193533000	-0.357919000
6	-3.174711000	-2.363235000	-0.459756000
6	-2.725135000	-1.048488000	-0.097626000
6	-3.331645000	0.231171000	-0.169928000
6	-2.544544000	1.408663000	-0.111639000
6	-2.886754000	2.754170000	-0.448027000
7	-1.215345000	1.342607000	0.201586000
7	2.345622000	1.144409000	-0.099910000
7	2.209524000	-1.333108000	-0.092198000
7	-1.388214000	-1.126442000	0.234637000
1	-1.586882000	4.529252000	-0.579271000
1	3.232412000	4.321136000	0.017487000
1	5.305303000	2.618880000	-0.183789000
1	4.977154000	-3.142687000	-0.163943000
1	2.721500000	-4.589256000	0.050937000
1	-3.864758000	3.091979000	-0.757360000
1	0.741454000	4.117077000	0.024992000
1	5.411679000	-0.271938000	-0.285869000
1	0.268567000	-4.090189000	0.051593000
1	-2.050340000	-4.247046000	-0.601341000
1	-4.157382000	-2.644157000	-0.801758000
8	-4.639611000	0.467575000	-0.415296000

6	-5.639075000	-0.533625000	-0.211220000
1	-5.725951000	-1.181211000	-1.089487000
1	-5.420970000	-1.131086000	0.679585000
1	-6.574098000	0.011410000	-0.074216000

E= –1428.4725884 a.u.
H= –1428.4725884 + 0.351480 (thermal correction 6-31+G**) a. u.
G= –1428.4725884 + 0.280005 (thermal correction 6-31+G**) a. u.

3c – akamptisomer *parvo,amplo* (***t*$_2$**)

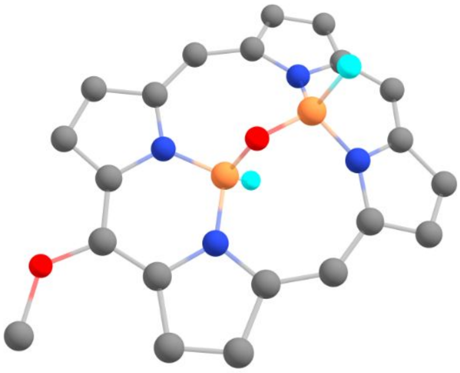

9	2.330846000	-0.090092000	2.209189000
5	-0.509247000	0.057937000	-0.050007000
5	1.645062000	-0.058587000	0.980977000
8	0.275175000	0.005566000	1.114195000
9	0.181262000	0.000820000	-1.290858000
6	-2.297693000	-3.227775000	0.062823000
6	-1.101979000	-2.426731000	0.054849000
6	0.198789000	-2.994326000	0.048309000
6	1.531308000	-2.557110000	-0.008479000
6	2.592662000	-3.463672000	-0.431516000
6	3.741182000	-2.740888000	-0.586156000
6	3.422263000	-1.377964000	-0.236620000
6	4.173438000	-0.203413000	-0.364765000
6	3.555413000	1.051411000	-0.239698000
6	4.033893000	2.364544000	-0.589428000
6	2.979068000	3.220131000	-0.433361000
6	1.820004000	2.447363000	-0.011312000
6	0.547810000	3.039712000	0.039516000
6	-0.803578000	2.618682000	0.041346000
6	-1.921891000	3.531071000	0.041688000
6	-3.081768000	2.793351000	-0.017573000
6	-2.687818000	1.418211000	-0.061107000
6	-3.479130000	0.242137000	-0.092559000
6	-2.877528000	-1.041539000	-0.051964000
6	-3.383553000	-2.389711000	0.001541000
7	-1.501839000	-1.123424000	-0.022240000
7	2.111028000	-1.321770000	0.154323000
7	2.246718000	1.147991000	0.148893000
7	-1.326147000	1.356571000	-0.031892000
1	-2.315084000	-4.308199000	0.114661000
1	2.441435000	-4.512474000	-0.651250000
1	4.701696000	-3.086798000	-0.943288000
1	5.028815000	2.593236000	-0.946547000
1	2.954101000	4.279654000	-0.652360000
1	-4.414366000	-2.700502000	0.003813000
1	0.140554000	-4.078345000	-0.020037000

1	5.208646000	-0.259811000	-0.680103000
1	0.615343000	4.123367000	-0.027291000
1	-4.101176000	3.149288000	-0.023092000
1	-1.833216000	4.608393000	0.087761000
8	-4.805159000	0.509534000	-0.144167000
6	-5.794065000	-0.515721000	-0.182874000
1	-5.660183000	-1.159119000	-1.059215000
1	-6.746999000	0.008684000	-0.257333000
1	-5.777026000	-1.113622000	0.734687000

E= –1428.4709414 a.u.
H= –1428.4709414 + 0.351543 (thermal correction 6-31+G**) a. u.
G= –1428.4709414 + 0.280110 (thermal correction 6-31+G**) a. u.

3c – transition state (*TS*)

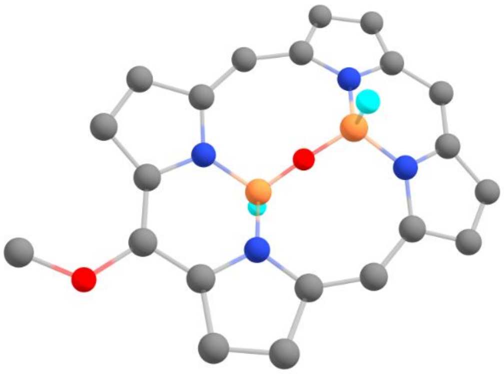

9	1.470469000	-0.026295000	-2.135107000
5	-0.684862000	0.073458000	0.696539000
5	1.576114000	-0.050757000	-0.723391000
8	0.439024000	-0.027137000	-0.015684000
9	-0.589099000	0.077102000	2.108980000
6	-1.847454000	3.489257000	-0.266110000
6	-0.767563000	2.562493000	0.003905000
6	0.616806000	2.954233000	0.021734000
6	1.926612000	2.414333000	-0.001352000
6	3.110909000	3.214113000	0.295790000
6	4.210294000	2.394345000	0.298182000
6	3.745505000	1.061195000	-0.024101000
6	4.379222000	-0.204434000	0.008442000
6	3.621507000	-1.394911000	-0.037175000
6	3.930180000	-2.772430000	0.295940000
6	2.744409000	-3.459200000	0.298937000
6	1.657525000	-2.529167000	-0.006405000
6	0.293675000	-2.911694000	0.031533000
6	-1.047557000	-2.374059000	0.014905000
6	-2.202114000	-3.196945000	-0.262004000
6	-3.328639000	-2.403194000	-0.260967000
6	-2.900008000	-1.059927000	0.047887000
6	-3.505313000	0.237070000	0.029964000
6	-2.717904000	1.431025000	0.037915000
6	-3.041114000	2.795255000	-0.261466000
7	-1.374724000	1.354973000	0.220749000
7	2.407984000	1.145890000	-0.243681000
7	2.285236000	-1.331952000	-0.268129000
7	-1.545078000	-1.120412000	0.240746000
1	-1.712047000	4.539683000	-0.488384000
1	3.088108000	4.269955000	0.532855000
1	5.230428000	2.664226000	0.535514000

1	4.911636000	-3.155651000	0.540457000
1	2.599788000	-4.502837000	0.546827000
1	-4.028463000	3.174967000	-0.479741000
1	0.675384000	4.041082000	0.066846000
1	5.444845000	-0.258128000	0.197551000
1	0.229482000	-3.997790000	0.090186000
1	-2.164894000	-4.254579000	-0.487784000
1	-4.326802000	-2.735984000	-0.494421000
8	-4.827160000	0.483958000	-0.084033000
6	-5.793473000	-0.566261000	-0.071505000
1	-5.761817000	-1.135112000	-1.006656000
1	-5.636760000	-1.232427000	0.783497000
1	-6.759358000	-0.068809000	0.018294000

E= –1428.4285931 a.u.
H= –1428.4285931 + 0.349906 (thermal correction 6-31+G**) a. u.
G= –1428.4285931 + 0.279243 (thermal correction 6-31+G**) a. u.
f = 180.4329 i

3c– akamptisomer *amplo, amplo* (c_1)

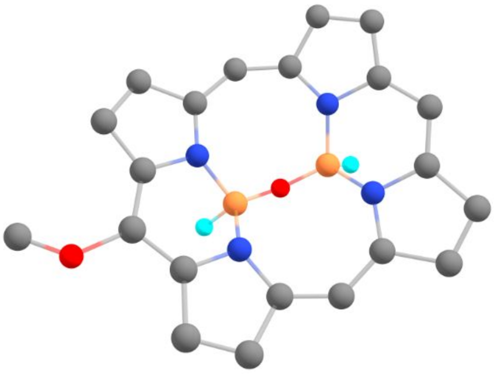

9	2.714463000	-0.108917000	1.909654000
5	-0.823159000	0.087758000	0.993613000
5	1.756478000	-0.063117000	0.868444000
8	0.485309000	-0.002401000	1.377879000
9	-1.685061000	0.175585000	2.114754000
6	-1.787427000	3.503591000	-0.284017000
6	-0.711379000	2.612536000	0.074868000
6	0.615263000	3.077785000	0.162375000
6	1.862709000	2.477109000	-0.018062000
6	3.004541000	3.246584000	-0.473450000
6	3.982522000	2.368045000	-0.851377000
6	3.476882000	1.048832000	-0.592358000
6	4.063694000	-0.199031000	-0.824183000
6	3.342464000	-1.370651000	-0.584522000
6	3.691206000	-2.742386000	-0.835411000
6	2.618967000	-3.498460000	-0.451132000
6	1.573882000	-2.598391000	0.002664000
6	0.268174000	-3.051288000	0.194315000
6	-1.006676000	-2.449520000	0.115005000
6	-2.151586000	-3.233156000	-0.275148000
6	-3.187966000	-2.374217000	-0.540550000
6	-2.703879000	-1.052792000	-0.281413000
6	-3.297683000	0.216699000	-0.441739000
6	-2.525038000	1.388478000	-0.314446000
6	-2.902743000	2.743894000	-0.541687000
7	-1.203573000	1.331052000	0.073119000
7	2.208303000	1.146871000	-0.063230000

7	2.072428000	-1.319079000	-0.052835000
7	-1.385681000	-1.134650000	0.137663000
1	-1.681546000	4.574784000	-0.394495000
1	3.013591000	4.324715000	-0.566233000
1	4.946455000	2.591742000	-1.287975000
1	4.621886000	-3.079753000	-1.271183000
1	2.502906000	-4.571021000	-0.536274000
1	-3.875886000	3.069985000	-0.877855000
1	0.671049000	4.163321000	0.160362000
1	5.065322000	-0.255446000	-1.233501000
1	0.203401000	-4.136281000	0.186329000
1	-2.142043000	-4.306671000	-0.408885000
1	-4.162122000	-2.640355000	-0.918710000
8	-4.585704000	0.426318000	-0.810642000
6	-5.628473000	-0.454323000	-0.371075000
1	-5.821674000	-1.227822000	-1.120752000
1	-5.375443000	-0.914733000	0.588559000
1	-6.519015000	0.167586000	-0.262214000

E= –1428.461082 a.u.
H= –1428.461082 + 0.351021 (thermal correction 6-31+G**) a. u.
G= –1428.461082 + 0.278836 (thermal correction 6-31+G**) a. u.

3d – akamptisomer *amplo,parvo* (t_1)

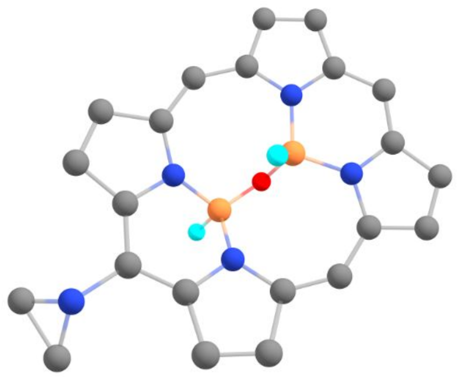

9	0.814004000	-0.000064000	-1.298891000
5	-0.593587000	-0.000015000	0.990462000
5	1.542225000	0.000008000	-0.075673000
8	0.774783000	0.000034000	1.107567000
9	-1.278126000	-0.000160000	2.223027000
6	-1.741365000	3.339611000	-0.428312000
6	-0.638237000	2.502255000	-0.010518000
6	0.681435000	3.026613000	0.047258000
6	1.992111000	2.534198000	0.016731000
6	3.170746000	3.383388000	0.004429000
6	4.277614000	2.582231000	-0.078909000
6	3.808901000	1.217255000	-0.122668000
6	4.504651000	0.000064000	-0.166303000
6	3.808925000	-1.217201000	-0.122737000
6	4.277669000	-2.582143000	-0.079148000
6	3.170791000	-3.383346000	0.003984000
6	1.992179000	-2.534189000	0.016580000
6	0.681491000	-3.026609000	0.047140000
6	-0.638190000	-2.502340000	-0.010404000
6	-1.741414000	-3.339704000	-0.427856000
6	-2.860939000	-2.552850000	-0.550770000
6	-2.464850000	-1.226795000	-0.187953000
6	-3.170114000	0.000013000	-0.267963000

6	-2.464942000	1.226773000	-0.187983000
6	-2.860951000	2.552802000	-0.551028000
7	-1.139943000	1.238397000	0.165379000
7	2.445429000	1.241725000	-0.073304000
7	2.445484000	-1.241669000	-0.073200000
7	-1.139887000	-1.238376000	0.165217000
1	-1.651536000	4.390627000	-0.669444000
1	3.144099000	4.463829000	0.058123000
1	5.315466000	2.885506000	-0.100990000
1	5.315517000	-2.885426000	-0.101293000
1	3.144185000	-4.463799000	0.057455000
1	-3.837184000	2.845347000	-0.909615000
1	0.668059000	4.113823000	0.011681000
1	5.587314000	0.000076000	-0.202244000
1	0.668176000	-4.113818000	0.011411000
7	-4.519117000	-0.000120000	-0.613760000
6	-5.513633000	-0.747944000	0.150240000
1	-6.242448000	-1.294439000	-0.442533000
1	-5.168912000	-1.250294000	1.052354000
6	-5.513627000	0.748161000	0.149709000
1	-5.168955000	1.251146000	1.051493000
1	-6.242492000	1.294236000	-0.443398000
1	-1.651707000	-4.390753000	-0.668927000
1	-3.837210000	-2.845435000	-0.909219000

E= –1446.6868675 a.u.
H= –1446.6868675 + 0.369732 (thermal correction 6-31+G**) a. u.
G= –1446.6868675 + 0.297712 (thermal correction 6-31+G**) a. u.

3d – akamptisomer *parvo,amplo* ($\boldsymbol{t_2}$)

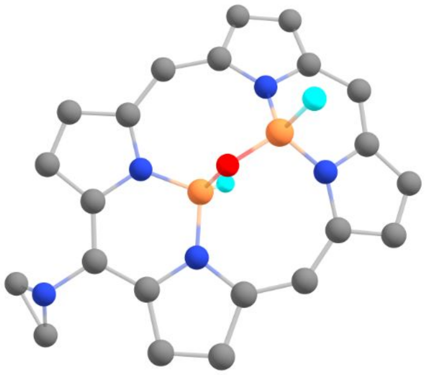

9	-2.599084000	-0.000008000	2.155377000
5	0.347561000	0.000023000	0.032098000
5	-1.852163000	0.000042000	0.962996000
8	-0.489525000	0.000093000	1.161043000
9	-0.287938000	0.000028000	-1.240125000
6	1.954702000	3.380962000	0.247680000
6	0.791599000	2.529555000	0.171748000
6	-0.533144000	3.026464000	0.106256000
6	-1.833594000	2.509164000	-0.019944000
6	-2.922914000	3.350138000	-0.497339000
6	-4.015538000	2.558299000	-0.715319000
6	-3.633313000	1.217269000	-0.349970000
6	-4.310349000	0.000049000	-0.513662000
6	-3.633309000	-1.217221000	-0.349901000
6	-4.015668000	-2.558258000	-0.715041000
6	-2.923020000	-3.350123000	-0.497185000
6	-1.833695000	-2.509210000	-0.019785000

6	-0.533230000	-3.026472000	0.106309000
6	0.791509000	-2.529579000	0.171808000
6	1.954617000	-3.380931000	0.247614000
6	3.075270000	-2.584138000	0.226744000
6	2.618632000	-1.227744000	0.132091000
6	3.325817000	0.000006000	0.110527000
6	2.618780000	1.227730000	0.132313000
6	3.075368000	2.584142000	0.227264000
7	1.250747000	1.243830000	0.098909000
7	-2.343603000	1.238046000	0.108721000
7	-2.343624000	-1.238004000	0.108725000
7	1.250677000	-1.243829000	0.099002000
1	1.921182000	4.460066000	0.318295000
1	-2.825153000	4.407388000	-0.706758000
1	-4.974982000	2.846494000	-1.122946000
1	-4.975158000	-2.846459000	-1.122552000
1	-2.825330000	-4.407381000	-0.706598000
1	4.105659000	2.900486000	0.296789000
1	-0.535808000	4.112895000	0.050746000
1	-5.329456000	0.000028000	-0.882171000
1	-0.535912000	-4.112920000	0.050923000
1	1.921200000	-4.460031000	0.318422000
1	4.105583000	-2.900504000	0.295894000
7	4.718664000	-0.000124000	0.186298000
6	5.555204000	-0.747795000	-0.747626000
1	6.379477000	-1.296087000	-0.299120000
1	5.053486000	-1.248396000	-1.574101000
6	5.555304000	0.747612000	-0.747402000
1	5.053731000	1.248547000	-1.573772000
1	6.379683000	1.295677000	-0.298797000

E= –1446.6846939 a.u.
H= –1446.6846939 + 0.369688 (thermal correction 6-31+G**) a. u.
G= –1446.6846939 + 0.297811   (thermal correction 6-31+G**) a. u.

3d – transition state (*TS*)

9	1.708319000	-0.000005000	-2.129103000
5	-0.536634000	0.000000000	0.636316000
5	1.769357000	-0.000001000	-0.714787000
8	0.611889000	0.000009000	-0.042156000
9	-0.484311000	-0.000004000	2.052506000
6	-1.856498000	3.343456000	-0.383305000
6	-0.741762000	2.474475000	-0.063931000
6	0.620457000	2.940691000	-0.007586000
6	1.961014000	2.477227000	0.007826000
6	3.088090000	3.343499000	0.344294000
6	4.231950000	2.588308000	0.384281000

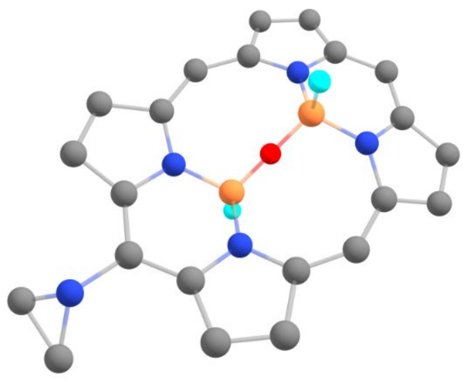

6	3.855314000	1.230434000	0.046448000
6	4.549613000	-0.000001000	0.109371000
6	3.855311000	-1.230434000	0.046451000
6	4.231948000	-2.588308000	0.384284000
6	3.088089000	-3.343500000	0.344298000
6	1.961012000	-2.477228000	0.007829000
6	0.620455000	-2.940692000	-0.007586000
6	-0.741762000	-2.474475000	-0.063934000
6	-1.856499000	-3.343454000	-0.383309000
6	-3.012579000	-2.590096000	-0.416039000
6	-2.637614000	-1.242693000	-0.089306000
6	-3.342652000	0.000001000	-0.128060000
6	-2.637616000	1.242695000	-0.089303000
6	-3.012579000	2.590098000	-0.416036000
7	-1.296868000	1.241634000	0.147863000
7	2.524464000	1.241523000	-0.222814000
7	2.524461000	-1.241524000	-0.222808000
7	-1.296866000	-1.241632000	0.147858000
1	-1.765795000	4.396495000	-0.615824000
1	2.996791000	4.395702000	0.581191000
1	5.225808000	2.915885000	0.657843000
1	5.225806000	-2.915884000	0.657845000
1	2.996790000	-4.395703000	0.581194000
1	-4.002444000	2.925765000	-0.690436000
1	0.617096000	4.029405000	0.034944000
1	5.609752000	-0.000002000	0.333839000
1	0.617094000	-4.029406000	0.034944000
1	-1.765797000	-4.396494000	-0.615827000
1	-4.002444000	-2.925761000	-0.690438000
7	-4.707088000	0.000000000	-0.394001000
6	-5.666477000	-0.748890000	0.409161000
1	-6.420522000	-1.294546000	-0.151861000
1	-5.283778000	-1.252584000	1.295331000
6	-5.666478000	0.748888000	0.409162000
1	-5.283779000	1.252580000	1.295335000
1	-6.420523000	1.294545000	-0.151858000

E= –1446.641803 a.u.
H= –1446.641803 + 0.368014 (thermal correction 6-31+G**) a. u.
G= –1446.641803 + 0.296090 (thermal correction 6-31+G**) a. u.
f = 181.6803 i

3d– akamptisomer *amplo, amplo* (c_1)

9	-0.813985000	-0.000054000	-1.298941000
5	0.593646000	-0.000103000	0.990110000
5	-1.542212000	-0.000024000	-0.075701000
8	-0.774731000	-0.000136000	1.107516000

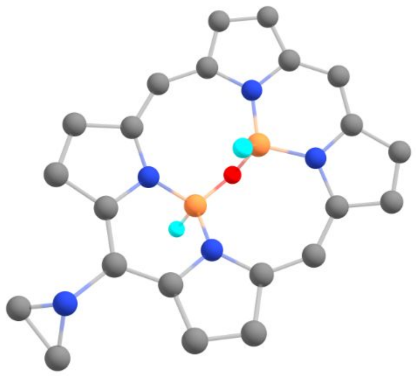

9	1.278358000	-0.000105000	2.222643000
6	1.741029000	-3.339705000	-0.428814000
6	0.638028000	-2.502242000	-0.010745000
6	-0.681641000	-3.026595000	0.047095000
6	-1.992314000	-2.534141000	0.016723000
6	-3.171012000	-3.383230000	0.004630000
6	-4.277820000	-2.581974000	-0.078562000
6	-3.809013000	-1.217034000	-0.122316000
6	-4.504627000	0.000225000	-0.165778000
6	-3.808801000	1.217453000	-0.122378000
6	-4.277420000	2.582411000	-0.078940000
6	-3.170452000	3.383520000	0.004205000
6	-1.991935000	2.534266000	0.016559000
6	-0.681210000	3.026688000	0.046867000
6	0.638409000	2.502444000	-0.010628000
6	1.741717000	3.339534000	-0.428383000
6	2.861151000	2.552468000	-0.551138000
6	2.464639000	1.226471000	-0.188230000
6	3.170142000	-0.000225000	-0.268026000
6	2.464806000	-1.226946000	-0.188333000
6	2.860701000	-2.552918000	-0.551537000
7	1.139850000	-1.238386000	0.164847000
7	-2.445515000	-1.241639000	-0.073260000
7	-2.445331000	1.241795000	-0.073211000
7	1.139851000	1.238244000	0.164856000
1	1.651124000	-4.390723000	-0.669878000
1	-3.144471000	-4.463674000	0.058287000
1	-5.315696000	-2.885186000	-0.100482000
1	-5.315239000	2.885797000	-0.100994000
1	-3.143772000	4.463972000	0.057665000
1	3.836809000	-2.845570000	-0.910382000
1	-0.668246000	-4.113798000	0.011460000
1	-5.587300000	0.000314000	-0.201492000
1	-0.667912000	4.113896000	0.011044000
1	1.651851000	4.390600000	-0.669292000
1	3.837495000	2.844446000	-0.909839000
7	4.519360000	0.000268000	-0.613464000
6	5.513395000	0.747941000	0.151738000
1	6.242415000	1.294524000	-0.440693000
1	5.167649000	1.250372000	1.053396000
6	5.513598000	-0.748133000	0.150684000
1	5.168067000	-1.251824000	1.051710000
1	6.242809000	-1.293622000	-0.442514000

E= –1446.6735688 a.u.

H= –1446.6735688 + 0.369731 (thermal correction 6-31+G**) a. u.

G= –1446.6735688 + 0.297718 (thermal correction 6-31+G**) a. u.

3e – akamptisomer *amplo,parvo* (***t***$_1$)

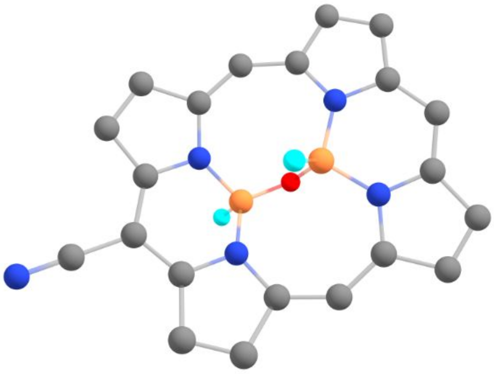

9	-0.508233000	-0.000002000	-1.271631000
5	0.813135000	-0.000005000	1.074589000
5	-1.274454000	0.000003000	-0.075367000
8	-0.560008000	-0.000002000	1.136297000
9	1.448055000	-0.000002000	2.325232000
6	2.024018000	-3.353750000	-0.278984000
6	0.892865000	-2.512464000	0.083131000
6	-0.415922000	-3.031428000	0.070893000
6	-1.734837000	-2.533645000	-0.003810000
6	-2.907990000	-3.383620000	-0.064548000
6	-4.012710000	-2.580912000	-0.187263000
6	-3.541182000	-1.219823000	-0.209348000
6	-4.229192000	0.000015000	-0.277402000
6	-3.541171000	1.219848000	-0.209349000
6	-4.012689000	2.580940000	-0.187262000
6	-2.907963000	3.383640000	-0.064548000
6	-1.734816000	2.533656000	-0.003811000
6	-0.415898000	3.031429000	0.070894000
6	0.892885000	2.512453000	0.083132000
6	2.024046000	3.353730000	-0.278978000
6	3.136013000	2.564550000	-0.379629000
6	2.713065000	1.228893000	-0.051516000
6	3.408411000	-0.000018000	-0.138724000
6	2.713057000	-1.228924000	-0.051513000
6	3.135993000	-2.564582000	-0.379633000
7	1.386750000	-1.240163000	0.266789000
7	-2.177899000	-1.245393000	-0.106374000
7	-2.177887000	1.245407000	-0.106375000
7	1.386757000	1.240147000	0.266783000
1	1.948293000	-4.410275000	-0.499568000
1	-2.883253000	-4.463986000	-0.013070000
1	-5.048823000	-2.884193000	-0.249030000
1	-5.048800000	2.884229000	-0.249027000
1	-2.883217000	4.464005000	-0.013071000
1	4.134399000	-2.845896000	-0.683925000
1	-0.405246000	-4.117600000	0.014378000
1	-5.310069000	0.000020000	-0.355324000
1	-0.405214000	4.117601000	0.014381000
1	1.948332000	4.410256000	-0.499557000
1	4.134422000	2.845855000	-0.683920000
6	4.797396000	-0.000029000	-0.461280000
7	5.931874000	0.000046000	-0.725663000

E= –1406.1781597 a.u.

H= –1406.1781597 + 0.316433 (thermal correction 6-31+G**) a. u.

G= –1406.1781597 + 0.247118 (thermal correction 6-31+G**) a. u.

3e – akamptisomer *parvo,amplo* (***t***$_2$)

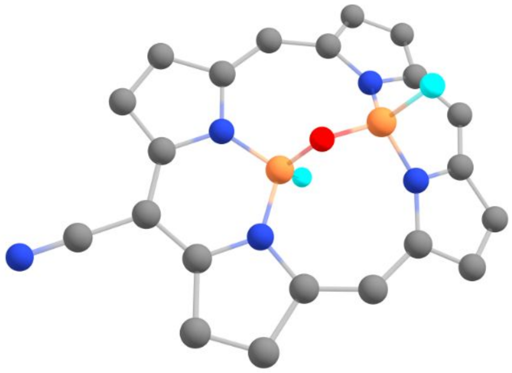

9	-2.244727000	-0.000010000	2.209149000
5	0.595050000	0.000002000	-0.051005000
5	-1.547905000	-0.000001000	0.991327000
8	-0.179367000	0.000003000	1.123003000
9	-0.111574000	0.000011000	-1.283732000
6	2.222748000	3.387573000	0.050423000
6	1.045458000	2.537865000	0.052321000
6	-0.266982000	3.031767000	0.069919000
6	-1.582582000	2.508232000	-0.000051000
6	-2.685716000	3.347977000	-0.435046000
6	-3.789993000	2.554897000	-0.598612000
6	-3.391745000	1.220035000	-0.242009000
6	-4.068726000	0.000005000	-0.378554000
6	-3.391747000	-1.220026000	-0.242017000
6	-3.789992000	-2.554883000	-0.598641000
6	-2.685745000	-3.347984000	-0.434979000
6	-1.582588000	-2.508229000	-0.000062000
6	-0.266989000	-3.031767000	0.069901000
6	1.045451000	-2.537867000	0.052302000
6	2.222740000	-3.387575000	0.050410000
6	3.332093000	-2.588834000	-0.027698000
6	2.858170000	-1.228563000	-0.077106000
6	3.558907000	-0.000003000	-0.118804000
6	2.858174000	1.228561000	-0.077101000
6	3.332100000	2.588830000	-0.027688000
7	1.499851000	1.243770000	-0.037096000
7	-2.079334000	1.242368000	0.157374000
7	-2.079334000	-1.242362000	0.157360000
7	1.499845000	-1.243771000	-0.037108000
1	2.195724000	4.467597000	0.106925000
1	-2.596301000	4.403649000	-0.654622000
1	-4.766111000	2.841699000	-0.965236000
1	-4.766104000	-2.841681000	-0.965284000
1	-2.596345000	-4.403668000	-0.654504000
1	4.371674000	2.884626000	-0.042129000
1	-0.277503000	4.118793000	0.032321000
1	-5.101266000	0.000007000	-0.708840000
1	-0.277512000	-4.118794000	0.032318000
1	2.195714000	-4.467600000	0.106907000
1	4.371667000	-2.884630000	-0.042130000
6	4.982792000	-0.000006000	-0.166878000
7	6.147159000	-0.000013000	-0.209143000

E= –1406.1783038 a.u.

H= –1406.1783038 + 0.316493 (thermal correction 6-31+G**) a. u.
G= –1406.1783038 + 0.247190 (thermal correction 6-31+G**) a. u.

3e – transition state (*TS*)

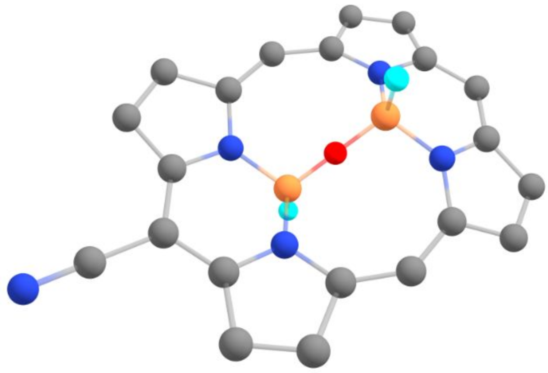

9	1.346504000	-0.000002000	-2.125595000
5	-0.769391000	0.000000000	0.742886000
5	1.467542000	0.000000000	-0.718790000
8	0.349524000	0.000003000	0.012710000
9	-0.647095000	-0.000002000	2.149508000
6	-2.137918000	3.349922000	-0.238554000
6	-0.998219000	2.480797000	0.035755000
6	0.345462000	2.943554000	-0.001940000
6	1.702192000	2.476344000	-0.017911000
6	2.834990000	3.344619000	0.270243000
6	3.982468000	2.587569000	0.267540000
6	3.591254000	1.233932000	-0.050667000
6	4.279281000	0.000000000	-0.009857000
6	3.591254000	-1.233932000	-0.050667000
6	3.982467000	-2.587569000	0.267540000
6	2.834990000	-3.344619000	0.270243000
6	1.702192000	-2.476344000	-0.017910000
6	0.345462000	-2.943554000	-0.001940000
6	-0.998219000	-2.480797000	0.035753000
6	-2.137918000	-3.349922000	-0.238554000
6	-3.284775000	-2.596816000	-0.226458000
6	-2.887048000	-1.242419000	0.086944000
6	-3.588837000	0.000000000	0.063672000
6	-2.887048000	1.242419000	0.086945000
6	-3.284775000	2.596816000	-0.226457000
7	-1.549100000	1.242028000	0.284487000
7	2.245548000	1.244648000	-0.260945000
7	2.245547000	-1.244648000	-0.260943000
7	-1.549099000	-1.242027000	0.284484000
1	-2.058248000	4.403312000	-0.473066000
1	2.753167000	4.397752000	0.505190000
1	4.986023000	2.916326000	0.500688000
1	4.986023000	-2.916327000	0.500687000
1	2.753167000	-4.397753000	0.505190000
1	-4.293045000	2.918559000	-0.446719000
1	0.346567000	4.032833000	-0.021794000
1	5.347655000	0.000000000	0.174772000
1	0.346566000	-4.032834000	-0.021793000
1	-2.058248000	-4.403312000	-0.473066000
1	-4.293045000	-2.918559000	-0.446718000
6	-4.999562000	0.000000000	-0.134841000
7	-6.153519000	0.000000000	-0.297660000

E= –1406.1334284 a.u.
H= –1406.1334284 + 0.314783 (thermal correction 6-31+G**) a. u.
G= –1406.1334284 + 0.245748 (thermal correction 6-31+G**) a. u.
f= 182.7387 i

3e– akamptisomer *amplo, amplo* (c_1)

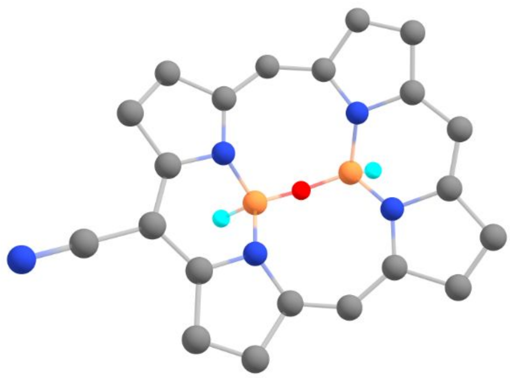

9	-2.669335000	-0.000121000	1.870235000
5	0.895659000	-0.000039000	1.038130000
5	-1.675990000	0.000003000	0.866210000
8	-0.420466000	0.000257000	1.411194000
9	1.750227000	-0.000110000	2.161559000
6	2.087966000	-3.384338000	-0.164642000
6	0.950732000	-2.549148000	0.167808000
6	-0.341090000	-3.081014000	0.219498000
6	-1.620026000	-2.544286000	-0.012057000
6	-2.694917000	-3.378482000	-0.507402000
6	-3.711021000	-2.556837000	-0.917858000
6	-3.289919000	-1.214055000	-0.643886000
6	-3.931777000	0.000032000	-0.900354000
6	-3.289976000	1.214139000	-0.643926000
6	-3.710891000	2.556935000	-0.918192000
6	-2.694789000	3.378529000	-0.507658000
6	-1.620002000	2.544275000	-0.012123000
6	-0.341058000	3.080950000	0.219595000
6	0.950764000	2.549059000	0.167980000
6	2.087997000	3.384321000	-0.164395000
6	3.148509000	2.566439000	-0.443977000
6	2.687724000	1.223441000	-0.241315000
6	3.367369000	-0.000013000	-0.409074000
6	2.687665000	-1.223439000	-0.241374000
6	3.148442000	-2.566430000	-0.444218000
7	1.372141000	-1.238521000	0.151091000
7	-2.033612000	-1.240123000	-0.072444000
7	-2.033751000	1.240187000	-0.072330000
7	1.372200000	1.238462000	0.151094000
1	2.047801000	-4.462149000	-0.250149000
1	-2.638975000	-4.455184000	-0.597850000
1	-4.645536000	-2.836025000	-1.385128000
1	-4.645302000	2.836146000	-1.385658000
1	-2.638722000	4.455214000	-0.598226000
1	4.139759000	-2.840824000	-0.776992000
1	-0.342923000	-4.167832000	0.225019000
1	-4.919275000	-0.000016000	-1.347121000
1	-0.342855000	4.167767000	0.225110000
1	2.047885000	4.462141000	-0.249795000
1	4.139842000	2.840891000	-0.776656000

6	4.735462000	-0.000042000	-0.810759000
7	5.850548000	-0.000024000	-1.147407000

E= –1406.1677941 a.u.
H= –1406.1677941 + 0.316433 (thermal correction 6-31+G**) a. u.
G= –1406.1677941 + 0.247119 (thermal correction 6-31+G**) a. u.

3f – akamptisomer *amplo,parvo* (***t*****1**)

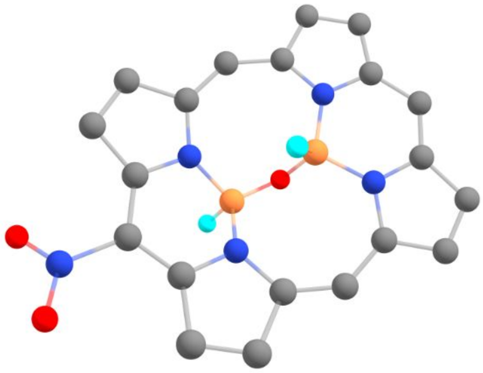

9	-0.744846000	-0.003586000	-1.281227000
5	0.586349000	-0.002000000	1.069309000
5	-1.506352000	0.004526000	-0.083169000
8	-0.787940000	0.005883000	1.124877000
9	1.211366000	-0.010844000	2.324953000
6	1.769505000	-3.376245000	-0.216277000
6	0.646839000	-2.511955000	0.108198000
6	-0.662394000	-3.021256000	0.099066000
6	-1.982799000	-2.521985000	0.015674000
6	-3.157267000	-3.368913000	-0.033997000
6	-4.259719000	-2.563067000	-0.165769000
6	-3.782858000	-1.205247000	-0.204189000
6	-4.464573000	0.017480000	-0.279890000
6	-3.771227000	1.234369000	-0.219962000
6	-4.234442000	2.597392000	-0.205767000
6	-3.123947000	3.394409000	-0.088728000
6	-1.958086000	2.536728000	-0.023253000
6	-0.632313000	3.023940000	0.047325000
6	0.671317000	2.501897000	0.070037000
6	1.802670000	3.344377000	-0.284006000
6	2.920618000	2.566202000	-0.359691000
6	2.505636000	1.223883000	-0.021008000
6	3.182759000	-0.017522000	-0.090161000
6	2.491425000	-1.252087000	-0.041830000
6	2.894013000	-2.610946000	-0.326379000
7	1.157538000	-1.240137000	0.260876000
7	-2.418753000	-1.235261000	-0.103017000
7	-2.406925000	1.252573000	-0.116940000
7	1.171673000	1.231436000	0.267887000
1	1.683649000	-4.437426000	-0.409435000
1	-3.136428000	-4.448591000	0.030725000
1	-5.297022000	-2.863194000	-0.222470000
1	-5.268643000	2.906872000	-0.268539000
1	-3.092214000	4.474826000	-0.043728000
1	3.884839000	-2.919687000	-0.613662000
1	-0.656235000	-4.108149000	0.058413000
1	-5.545658000	0.022166000	-0.356280000
1	-0.614858000	4.109413000	-0.019795000
1	1.725911000	4.398916000	-0.513501000

1	3.920119000	2.858838000	-0.637378000
7	4.619396000	-0.003718000	-0.333575000
8	5.137134000	-0.982605000	-0.881191000
8	5.251611000	0.994259000	0.027369000

E= –1518.4902275 a.u.
H= –1518.4902275 + 0.321156 (thermal correction 6-31+G**) a. u.
G= –1518.4902275 + 0.249825 (thermal correction 6-31+G**) a. u.

3f – akamptisomer *parvo,amplo* ($\boldsymbol{t_2}$)

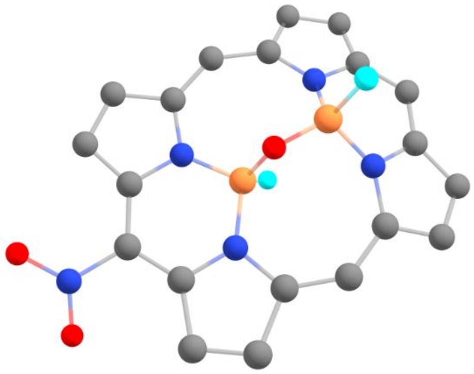

9	-2.492383000	-0.017032000	2.202306000
5	0.368920000	-0.002838000	-0.036509000
5	-1.790387000	-0.007324000	0.987610000
8	-0.422475000	-0.005541000	1.126138000
9	-0.327222000	-0.005148000	-1.275540000
6	1.972055000	3.389816000	0.117100000
6	0.804186000	2.530634000	0.098738000
6	-0.505591000	3.022040000	0.114952000
6	-1.824108000	2.503967000	0.021490000
6	-2.919859000	3.350079000	-0.415368000
6	-4.024915000	2.559916000	-0.594670000
6	-3.631479000	1.222291000	-0.247264000
6	-4.306519000	0.003527000	-0.399780000
6	-3.630694000	-1.217338000	-0.267190000
6	-4.023696000	-2.547834000	-0.641417000
6	-2.918331000	-3.340816000	-0.478391000
6	-1.822833000	-2.503373000	-0.024272000
6	-0.504684000	-3.024299000	0.056871000
6	0.806097000	-2.534884000	0.056608000
6	1.972469000	-3.396136000	0.049652000
6	3.091537000	-2.614305000	-0.007123000
6	2.638150000	-1.239477000	-0.037151000
6	3.320985000	-0.000096000	-0.092909000
6	2.632938000	1.237303000	-0.058886000
6	3.089102000	2.608933000	0.023171000
7	1.272488000	1.240275000	-0.013060000
7	-2.320885000	1.239121000	0.160971000
7	-2.320431000	-1.241781000	0.141926000
7	1.276696000	-1.242845000	-0.009998000
1	1.935623000	4.468348000	0.193618000
1	-2.826935000	4.407599000	-0.623971000
1	-4.997709000	2.851927000	-0.965948000
1	-4.996317000	-2.832384000	-1.018872000
1	-2.824816000	-4.393816000	-0.708502000
1	4.120057000	2.921111000	0.005085000
1	-0.514261000	4.109607000	0.096943000

1	-5.336394000	0.005797000	-0.738553000
1	-0.514553000	-4.111107000	0.012912000
1	1.934303000	-4.476566000	0.090317000
1	4.122289000	-2.926746000	-0.010399000
7	4.773554000	0.007921000	-0.156615000
8	5.328454000	1.002875000	-0.636498000
8	5.384481000	-0.978817000	0.268289000

E= –1518.4907782 a.u.
H= –1518.4907782 + 0.321223 (thermal correction 6-31+G**) a. u.
G= –1518.4907782 + 0.249961 (thermal correction 6-31+G**) a. u.

3f – transition state (*TS*)

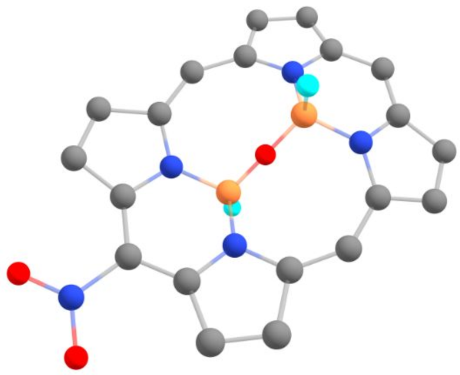

9	1.618363000	-0.005865000	-2.131233000
5	-0.544925000	-0.001520000	0.703044000
5	1.716957000	-0.000562000	-0.723680000
8	0.588650000	0.003041000	-0.007715000
9	-0.440296000	-0.006408000	2.111413000
6	-1.889801000	3.353280000	-0.270368000
6	-0.757199000	2.474380000	-0.004043000
6	0.580353000	2.935260000	-0.034937000
6	1.944300000	2.473827000	-0.025369000
6	3.066403000	3.345578000	0.282403000
6	4.216973000	2.590925000	0.298241000
6	3.834205000	1.238347000	-0.026375000
6	4.520645000	0.005085000	0.032067000
6	3.836349000	-1.229910000	-0.017806000
6	4.222644000	-2.579265000	0.315624000
6	3.074106000	-3.337039000	0.304731000
6	1.949755000	-2.470424000	-0.009072000
6	0.587489000	-2.935954000	-0.016181000
6	-0.751490000	-2.478439000	0.007574000
6	-1.881322000	-3.364169000	-0.245799000
6	-3.036839000	-2.630995000	-0.238825000
6	-2.660609000	-1.256494000	0.050338000
6	-3.354980000	-0.004594000	0.062956000
6	-2.662700000	1.248586000	0.059653000
6	-3.042956000	2.617022000	-0.251880000
7	-1.322020000	1.235171000	0.237865000
7	2.490245000	1.245503000	-0.258285000
7	2.492336000	-1.241933000	-0.249531000
7	-1.319213000	-1.237685000	0.230951000
1	-1.802488000	4.407068000	-0.500443000
1	2.977456000	4.398364000	0.515900000
1	5.215608000	2.922129000	0.548291000
1	5.222193000	-2.906179000	0.567657000
1	2.987937000	-4.388535000	0.544987000

1	-4.046704000	2.952948000	-0.451710000
1	0.579653000	4.024233000	-0.067534000
1	5.585306000	0.006637000	0.238049000
1	0.589919000	-4.025149000	-0.038007000
1	-1.791250000	-4.420781000	-0.461535000
1	-4.038433000	-2.971424000	-0.439657000
7	-4.801325000	0.002390000	-0.039659000
8	-5.389635000	1.084638000	0.076592000
8	-5.385774000	-1.071755000	-0.226554000

E= –1518.4473976 a.u.
H= –1518.4473976 + 0.319546 (thermal correction 6-31+G**) a. u.
G= –1518.4473976 + 0.247755 (thermal correction 6-31+G**) a. u.
f = 178.3102 i

3f– akamptisomer *amplo, amplo* (c_1)

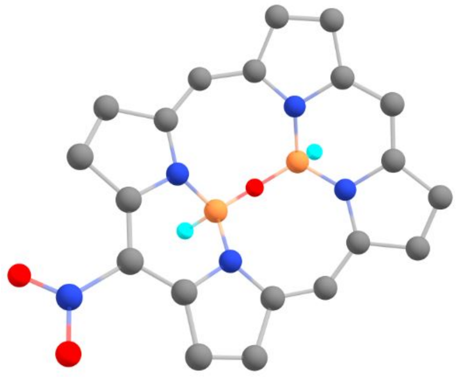

9	-2.922566000	0.035247000	1.850328000
5	0.657268000	0.000380000	1.073771000
5	-1.918521000	0.017982000	0.857209000
8	-0.668392000	0.014930000	1.412919000
9	1.482049000	-0.017985000	2.219158000
6	1.836064000	-3.398590000	-0.040685000
6	0.701933000	-2.543329000	0.237763000
6	-0.591931000	-3.065257000	0.275194000
6	-1.867290000	-2.530440000	0.009635000
6	-2.934313000	-3.367443000	-0.495383000
6	-3.942157000	-2.546916000	-0.930483000
6	-3.521855000	-1.203172000	-0.663187000
6	-4.151566000	0.012005000	-0.943472000
6	-3.506221000	1.225036000	-0.690347000
6	-3.909820000	2.567192000	-0.989267000
6	-2.892209000	3.385122000	-0.572753000
6	-1.836114000	2.547351000	-0.046367000
6	-0.556046000	3.074852000	0.210852000
6	0.733813000	2.541690000	0.198415000
6	1.874445000	3.379939000	-0.109642000
6	2.948660000	2.572078000	-0.350100000
6	2.496521000	1.220558000	-0.142874000
6	3.150011000	-0.017885000	-0.302164000
6	2.467553000	-1.244747000	-0.173228000
6	2.912265000	-2.606358000	-0.322857000
7	1.140203000	-1.236816000	0.191695000
7	-2.274625000	-1.228108000	-0.071577000
7	-2.259124000	1.248341000	-0.098083000
7	1.166898000	1.233612000	0.206257000
7	4.563442000	-0.021604000	-0.661128000
8	4.993380000	-0.965504000	-1.332430000

8	5.258725000	0.924139000	-0.279298000

E= –1518.479437 a.u.
H= –1518.479437 + 0.321156 (thermal correction 6-31+G**) a. u.
G= –1518.479437 + 0.249822 (thermal correction 6-31+G**) a. u.

3g – akamptisomer *amplo,parvo* (***t*$_1$**)

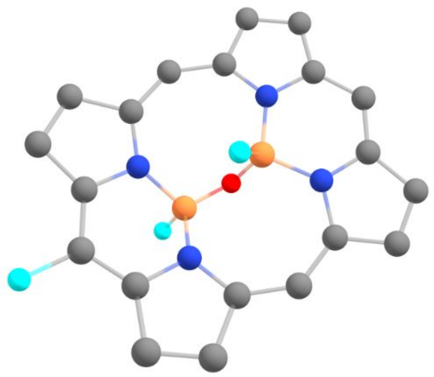

9	-0.385065000	-0.000006000	-1.277505000
5	0.967430000	0.000001000	1.045325000
5	-1.138802000	0.000000000	-0.070942000
8	-0.402737000	-0.000001000	1.130850000
9	1.628500000	0.000009000	2.283698000
6	2.153167000	-3.346800000	-0.351699000
6	1.035567000	-2.510438000	0.046233000
6	-0.282035000	-3.033537000	0.071460000
6	-1.593021000	-2.535739000	0.011895000
6	-2.771499000	-3.383975000	-0.027976000
6	-3.875416000	-2.581497000	-0.136679000
6	-3.403605000	-1.218112000	-0.170263000
6	-4.095913000	0.000006000	-0.229381000
6	-3.403599000	1.218120000	-0.170269000
6	-3.875406000	2.581507000	-0.136693000
6	-2.771487000	3.383981000	-0.027983000
6	-1.593011000	2.535742000	0.011887000
6	-0.282025000	3.033539000	0.071454000
6	1.035576000	2.510436000	0.046230000
6	2.153183000	3.346798000	-0.351680000
6	3.268472000	2.557666000	-0.471843000
6	2.854206000	1.232170000	-0.121340000
6	3.510388000	-0.000006000	-0.221190000
6	2.854204000	-1.232181000	-0.121333000
6	3.268462000	-2.557676000	-0.471848000
7	1.527187000	-1.244647000	0.228269000
7	-2.040879000	-1.243926000	-0.089274000
7	-2.040872000	1.243931000	-0.089279000
7	1.527187000	1.244640000	0.228255000
1	2.072010000	-4.401321000	-0.579975000
1	-2.747187000	-4.464318000	0.026765000
1	-4.912814000	-2.883483000	-0.182188000
1	-4.912803000	2.883495000	-0.182205000
1	-2.747172000	4.464324000	0.026760000
1	4.257119000	-2.840216000	-0.803506000
1	-0.270845000	-4.120425000	0.031410000
1	-5.177591000	0.000009000	-0.290011000
1	-0.270833000	4.120427000	0.031411000
1	2.072035000	4.401322000	-0.579940000
1	4.257131000	2.840203000	-0.803500000

9	4.815910000	-0.000007000	-0.567684000

E= –1413.1769451 a.u.
H= –1413.1769451 + 0.308874 (thermal correction 6-31+G**) a. u.
G= –1413.1769451 + 0.241925 (thermal correction 6-31+G**) a. u.

3g – akamptisomer *parvo,amplo* (***t*$_2$**)

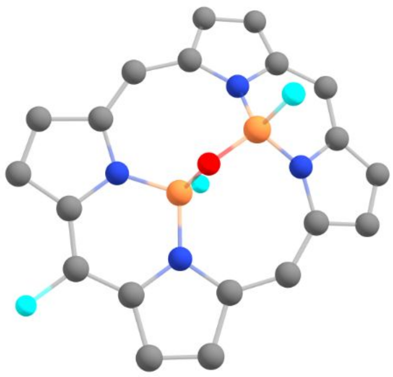

9	-2.086756000	-0.000008000	2.216940000
5	0.735161000	0.000001000	-0.062797000
5	-1.401349000	-0.000001000	0.990726000
8	-0.030529000	0.000002000	1.115273000
9	0.026293000	0.000010000	-1.293507000
6	2.367809000	3.384276000	0.025989000
6	1.193146000	2.536555000	0.032184000
6	-0.128958000	3.033244000	0.048488000
6	-1.436674000	2.510404000	-0.001204000
6	-2.550084000	3.349444000	-0.421549000
6	-3.653318000	2.556554000	-0.574689000
6	-3.250368000	1.218385000	-0.225253000
6	-3.932655000	0.000007000	-0.352473000
6	-3.250372000	-1.218373000	-0.225260000
6	-3.653321000	-2.556537000	-0.574715000
6	-2.550112000	-3.349446000	-0.421494000
6	-1.436682000	-2.510399000	-0.001216000
6	-0.128969000	-3.033245000	0.048472000
6	1.193136000	-2.536559000	0.032168000
6	2.367798000	-3.384282000	0.025980000
6	3.482036000	-2.585086000	-0.048955000
6	3.007328000	-1.231777000	-0.094991000
6	3.670373000	-0.000006000	-0.131763000
6	3.007335000	1.231769000	-0.094988000
6	3.482045000	2.585077000	-0.048949000
7	1.639713000	1.247788000	-0.053610000
7	-1.936372000	1.240904000	0.161729000
7	-1.936373000	-1.240897000	0.161716000
7	1.639705000	-1.247793000	-0.053621000
1	2.342066000	4.464577000	0.078743000
1	-2.464143000	4.405584000	-0.640983000
1	-4.633844000	2.842711000	-0.930074000
1	-4.633842000	-2.842689000	-0.930118000
1	-2.464187000	-4.405596000	-0.640885000
1	4.519989000	2.884451000	-0.063470000
1	-0.137824000	4.119416000	-0.007643000
1	-4.969382000	0.000009000	-0.668415000
1	-0.137838000	-4.119417000	-0.007647000
1	2.342051000	-4.464583000	0.078731000
1	4.519979000	-2.884461000	-0.063468000

9	5.021404000	-0.000010000	-0.173966000

E= –1413.1757291 a.u.
H= –1413.1757291 + 0.308864 (thermal correction 6-31+G**) a. u.
G= –1413.1757291 + 0.241906 (thermal correction 6-31+G**) a. u.

3g – transition state (*TS*)

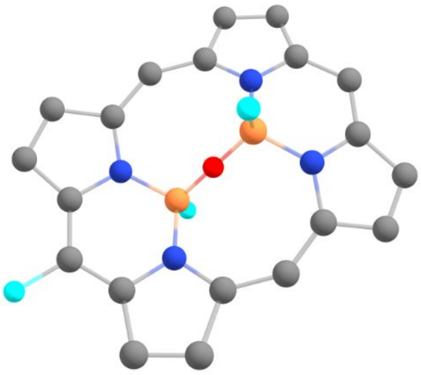

9	1.214637000	-0.000055000	-2.123402000
5	-0.915446000	-0.000002000	0.732675000
5	1.330908000	-0.000005000	-0.714023000
8	0.203437000	0.000083000	0.006107000
9	-0.806290000	-0.000039000	2.141019000
6	-2.277683000	3.343731000	-0.271749000
6	-1.147190000	2.478153000	0.025024000
6	0.210581000	2.944657000	0.019346000
6	1.554806000	2.478067000	-0.006611000
6	2.693297000	3.345302000	0.282060000
6	3.837924000	2.588968000	0.279930000
6	3.446914000	1.231578000	-0.038271000
6	4.140321000	0.000003000	-0.000933000
6	3.446899000	-1.231564000	-0.038227000
6	3.837920000	-2.588952000	0.279967000
6	2.693300000	-3.345297000	0.282099000
6	1.554803000	-2.478071000	-0.006569000
6	0.210585000	-2.944671000	0.019348000
6	-1.147178000	-2.478157000	0.024990000
6	-2.277673000	-3.343728000	-0.271787000
6	-3.430492000	-2.590746000	-0.273103000
6	-3.037755000	-1.246229000	0.055057000
6	-3.699119000	-0.000001000	0.018289000
6	-3.037776000	1.246237000	0.055096000
6	-3.430506000	2.590755000	-0.273067000
7	-1.692422000	1.247004000	0.270713000
7	2.104951000	1.242720000	-0.252394000
7	2.104928000	-1.242714000	-0.252328000
7	-1.692395000	-1.247000000	0.270662000
1	-2.195058000	4.395868000	-0.510946000
1	2.611999000	4.398424000	0.517977000
1	4.841850000	2.916888000	0.513025000
1	4.841851000	-2.916864000	0.513052000
1	2.612012000	-4.398421000	0.518010000
1	-4.432695000	2.915343000	-0.513396000
1	0.209939000	4.033891000	0.038488000
1	5.208776000	-0.000003000	0.180775000
1	0.209942000	-4.033904000	0.038484000
1	-2.195054000	-4.395866000	-0.510980000
1	-4.432684000	-2.915327000	-0.513427000

9	-5.032011000	-0.000011000	-0.199294000

E= –1413.1313318 a.u.
H= –1413.1313318 + 0.307177 (thermal correction 6-31+G**) a. u.
G= –1413.1313318 + 0.240461 (thermal correction 6-31+G**) a. u.
f= 184.0735 i

3g– akamptisomer *amplo, amplo* (c_1)

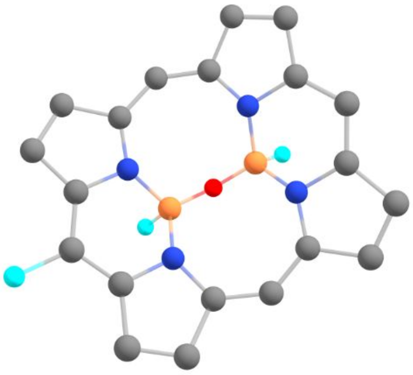

9	-2.478189000	-0.000290000	1.916212000
5	1.058799000	-0.000200000	0.983161000
5	-1.515240000	0.000063000	0.881052000
8	-0.244394000	0.001088000	1.394176000
9	1.947773000	-0.000119000	2.080927000
6	2.213852000	-3.379520000	-0.282844000
6	1.093226000	-2.548097000	0.098123000
6	-0.203809000	-3.083285000	0.196398000
6	-1.482131000	-2.546979000	0.008721000
6	-2.579705000	-3.379857000	-0.444411000
6	-3.602606000	-2.557722000	-0.830198000
6	-3.168713000	-1.212149000	-0.578903000
6	-3.820442000	0.000566000	-0.816691000
6	-3.168594000	1.213251000	-0.578990000
6	-3.601510000	2.558979000	-0.831326000
6	-2.578128000	3.380614000	-0.445752000
6	-1.481272000	2.547179000	0.008309000
6	-0.202701000	3.082887000	0.196325000
6	1.094320000	2.547554000	0.098672000
6	2.215185000	3.378983000	-0.281520000
6	3.274179000	2.560429000	-0.583220000
6	2.821330000	1.225778000	-0.353128000
6	3.461075000	-0.000583000	-0.529376000
6	2.820216000	-1.226296000	-0.353042000
6	3.272774000	-2.560886000	-0.584450000
7	1.510467000	-1.242553000	0.083503000
7	-1.896787000	-1.238332000	-0.047052000
7	-1.896977000	1.238954000	-0.046646000
7	1.511436000	1.241945000	0.082966000
9	4.744899000	-0.001333000	-0.950248000

E= –1413.1662952 a.u.
H= –1413.1662952 + 0.308874 (thermal correction 6-31+G**) a. u.
G= –1413.1662952 + 0.241926 (thermal correction 6-31+G**) a. u.

STRUCTURAL VARIATION 4

4a – akamptisomer *amplo,parvo* (***t_1***)

9	0.412733000	0.000161000	-1.291685000
5	-0.956086000	-0.000069000	1.026480000
5	1.158010000	-0.000009000	-0.079527000
8	0.416345000	0.000021000	1.117087000
9	-1.611407000	-0.000285000	2.272343000
6	-2.133420000	3.346532000	-0.335675000
6	-1.017372000	2.501747000	0.045894000
6	0.298139000	3.025481000	0.073013000
6	1.612598000	2.532425000	0.012214000
6	2.788879000	3.382525000	-0.022369000
6	3.894811000	2.581561000	-0.132909000
6	3.425230000	1.218224000	-0.171413000
6	4.117411000	-0.000136000	-0.229946000
6	3.425071000	-1.218425000	-0.171569000
6	3.894575000	-2.581779000	-0.133133000
6	2.788580000	-3.382696000	-0.022785000
6	1.612368000	-2.532541000	0.011958000
6	0.297877000	-3.025523000	0.072804000
6	-1.017590000	-2.501743000	0.045890000
6	-2.133828000	-3.346460000	-0.335221000
6	-3.247753000	-2.559223000	-0.456786000
6	-2.845650000	-1.216728000	-0.128525000
6	-3.553822000	0.000148000	-0.230592000
6	-2.845579000	1.216989000	-0.128436000
6	-3.247433000	2.559419000	-0.457212000
7	-1.515491000	1.235571000	0.212202000
7	2.061883000	1.242952000	-0.092921000
7	2.061721000	-1.243066000	-0.093096000
7	-1.515484000	-1.235395000	0.211796000
1	-2.050684000	4.403291000	-0.553255000
1	2.763120000	4.462634000	0.036690000
1	4.931804000	2.885552000	-0.175312000
1	4.931546000	-2.885845000	-0.175518000
1	2.762767000	-4.462811000	0.036131000
1	-4.231305000	2.868077000	-0.780001000
1	0.284969000	4.112683000	0.038945000
1	5.199328000	-0.000225000	-0.287845000
1	0.284654000	-4.112728000	0.038792000
1	-2.051337000	-4.403298000	-0.552520000
1	-4.231690000	-2.867900000	-0.779354000
6	-5.033012000	0.000279000	-0.515802000
1	-5.336385000	0.881418000	-1.085677000
1	-5.336963000	-0.882019000	-1.083591000
1	-5.601024000	0.001605000	0.422852000

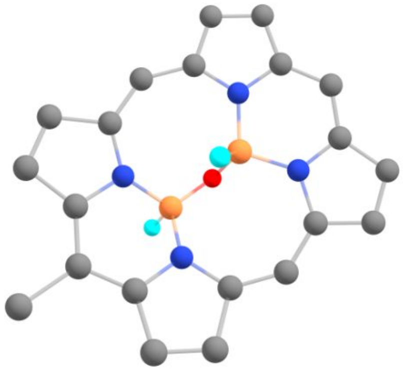

E= –1353.2336532 a.u.
H= –1353.2336532 + 0.345557 (thermal correction 6-31+G**) a. u.

$G = -1353.2336532 + 0.276316$ (thermal correction 6-31+G**) a. u.

4a – akamptisomer *parvo,amplo* (***t*$_2$**)

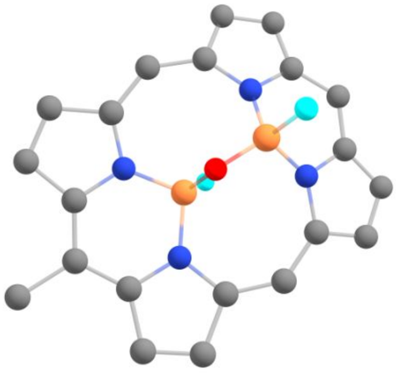

9	-2.105841000	-0.000077000	2.216937000
5	0.724084000	0.000091000	-0.059998000
5	-1.418510000	-0.000032000	0.990099000
8	-0.048744000	0.000088000	1.116549000
9	0.010804000	0.000098000	-1.291522000
6	2.346608000	3.379842000	0.026141000
6	1.175604000	2.527849000	0.034902000
6	-0.143986000	3.026088000	0.051331000
6	-1.454570000	2.507191000	-0.002072000
6	-2.564832000	3.347532000	-0.424820000
6	-3.670007000	2.555949000	-0.577909000
6	-3.269824000	1.218234000	-0.226538000
6	-3.951788000	-0.000165000	-0.354521000
6	-3.269676000	-1.218523000	-0.226513000
6	-3.669760000	-2.556262000	-0.577841000
6	-2.564524000	-3.347772000	-0.424700000
6	-1.454338000	-2.507342000	-0.002001000
6	-0.143707000	-3.026113000	0.051349000
6	1.175824000	-2.527729000	0.034887000
6	2.346968000	-3.379519000	0.026051000
6	3.458515000	-2.580332000	-0.052951000
6	2.996877000	-1.215492000	-0.097194000
6	3.710282000	0.000212000	-0.139843000
6	2.996941000	1.215888000	-0.097157000
6	3.458284000	2.580890000	-0.052835000
7	1.629044000	1.240572000	-0.050209000
7	-1.955570000	1.239833000	0.161486000
7	-1.955434000	-1.239988000	0.161498000
7	1.629048000	-1.240358000	-0.050210000
1	2.317620000	4.460169000	0.078746000
1	-2.476777000	4.403207000	-0.645764000
1	-4.649561000	2.843483000	-0.935000000
1	-4.649293000	-2.843880000	-0.934924000
1	-2.476400000	-4.403451000	-0.645594000
1	4.490932000	2.898960000	-0.068134000
1	-0.150784000	4.112589000	-0.001608000
1	-4.988115000	-0.000232000	-0.672053000
1	-0.150399000	-4.112616000	-0.001579000
1	2.318161000	-4.459854000	0.078609000
1	4.491288000	-2.898028000	-0.068346000
6	5.218475000	-0.000396000	-0.161215000
1	5.627237000	-0.007657000	0.857009000
1	5.609244000	-0.878417000	-0.681103000
1	5.610465000	0.883948000	-0.669144000

E= –1353.2333437 a.u.
H= –1353.2333437 + 0.345537 (thermal correction 6-31+G**) a. u.
G= –1353.2333437 + 0.275903 (thermal correction 6-31+G**) a. u.

4a – transition state (*TS*)

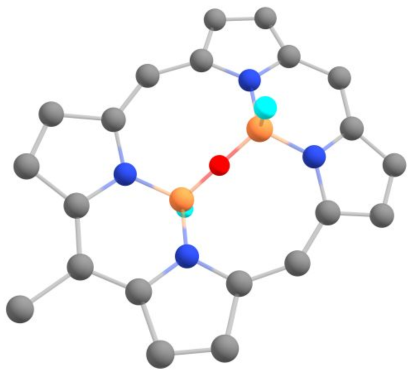

9	1.245844000	0.000001000	-2.132205000
5	-0.904264000	0.000000000	0.709557000
5	1.353178000	0.000000000	-0.721012000
8	0.223981000	-0.000001000	-0.005777000
9	-0.797856000	0.000000000	2.121859000
6	-2.259633000	3.344464000	-0.258873000
6	-1.129551000	2.472096000	0.018754000
6	0.225481000	2.938881000	0.015086000
6	1.573266000	2.475138000	-0.007044000
6	2.707511000	3.342482000	0.292387000
6	3.854168000	2.587561000	0.293228000
6	3.467133000	1.231929000	-0.033047000
6	4.159702000	0.000001000	0.008572000
6	3.467133000	-1.231927000	-0.033047000
6	3.854169000	-2.587559000	0.293228000
6	2.707513000	-3.342481000	0.292386000
6	1.573267000	-2.475138000	-0.007044000
6	0.225482000	-2.938881000	0.015086000
6	-1.129550000	-2.472096000	0.018754000
6	-2.259631000	-3.344466000	-0.258873000
6	-3.409759000	-2.590663000	-0.261733000
6	-3.026574000	-1.230470000	0.042429000
6	-3.739269000	-0.000001000	0.006737000
6	-3.026574000	1.230469000	0.042428000
6	-3.409760000	2.590661000	-0.261734000
7	-1.679623000	1.238575000	0.246183000
7	2.125903000	1.242842000	-0.255928000
7	2.125904000	-1.242841000	-0.255929000
7	-1.679623000	-1.238576000	0.246184000
1	-2.176841000	4.399706000	-0.484469000
1	2.623195000	4.394293000	0.533155000
1	4.856048000	2.916023000	0.534435000
1	4.856049000	-2.916021000	0.534435000
1	2.623196000	-4.394292000	0.533155000
1	-4.408011000	2.936118000	-0.490739000
1	0.223978000	4.028175000	0.037874000
1	5.226753000	0.000001000	0.198958000
1	0.223980000	-4.028175000	0.037874000
1	-2.176839000	-4.399707000	-0.484469000
1	-4.408010000	-2.936122000	-0.490738000

6	-5.237615000	0.000002000	-0.148074000
1	-5.727404000	0.000048000	0.833886000
1	-5.586881000	0.882971000	-0.689017000
1	-5.586893000	-0.883010000	-0.688939000

E= –1353.1888154 a.u.
H= –1353.1888154 + 0.343824 (thermal correction 6-31+G**) a. u.
G= –1353.1888154 + 0.274920 (thermal correction 6-31+G**) a. u.
f = 183.2138 i

4a– akamptisomer *amplo, amplo* (c_1)

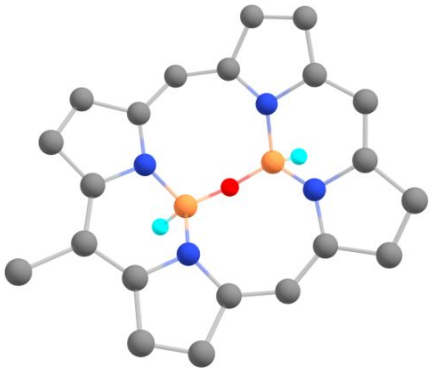

9	-2.494680000	-0.000122000	1.913944000
5	1.045026000	0.000002000	0.973792000
5	-1.533725000	-0.000020000	0.875009000
8	-0.261919000	0.000160000	1.382519000
9	1.924787000	-0.000091000	2.084284000
6	2.194532000	-3.375573000	-0.265195000
6	1.075458000	-2.536816000	0.101405000
6	-0.220043000	-3.072962000	0.194726000
6	-1.501629000	-2.542876000	0.001831000
6	-2.595039000	-3.378584000	-0.453641000
6	-3.621887000	-2.558755000	-0.836852000
6	-3.192733000	-1.212727000	-0.582953000
6	-3.845192000	-0.000024000	-0.819523000
6	-3.192804000	1.212712000	-0.582921000
6	-3.621941000	2.558739000	-0.836878000
6	-2.595081000	3.378561000	-0.453687000
6	-1.501690000	2.542848000	0.001845000
6	-0.220096000	3.072929000	0.194726000
6	1.075432000	2.536829000	0.101437000
6	2.194474000	3.375634000	-0.265167000
6	3.253792000	2.560294000	-0.563757000
6	2.815663000	1.210972000	-0.347425000
6	3.506796000	0.000032000	-0.530730000
6	2.815615000	-1.210893000	-0.347469000
6	3.253807000	-2.560183000	-0.563803000
7	1.501358000	-1.233885000	0.079706000
7	-1.920334000	-1.237119000	-0.051607000
7	-1.920439000	1.237108000	-0.051536000
7	1.501433000	1.233932000	0.079798000
1	2.145853000	-4.452565000	-0.357767000
1	-2.542527000	-4.455897000	-0.540733000
1	-4.570721000	-2.839731000	-1.273442000
1	-4.570762000	2.839721000	-1.273494000
1	-2.542550000	4.455870000	-0.540822000
1	4.228530000	-2.860673000	-0.920364000

1	-0.217115000	-4.159884000	0.202200000
1	-4.847830000	-0.000054000	-1.230759000
1	-0.217185000	4.159853000	0.202148000
1	2.145755000	4.452624000	-0.357745000
1	4.228491000	2.860853000	-0.920317000
6	4.963502000	-0.000054000	-0.917925000
1	5.225200000	-0.880665000	-1.508973000
1	5.225518000	0.880930000	-1.508250000
1	5.597478000	-0.000529000	-0.022687000

E= –1353.2233018 a.u.
H= –1353.2233018 + 0.345223 (thermal correction 6-31+G**) a. u.
G= –1353.2233018 + 0.275432 (thermal correction 6-31+G**) a. u.

4b – akamptisomer *amplo,parvo* (***t***$_1$)

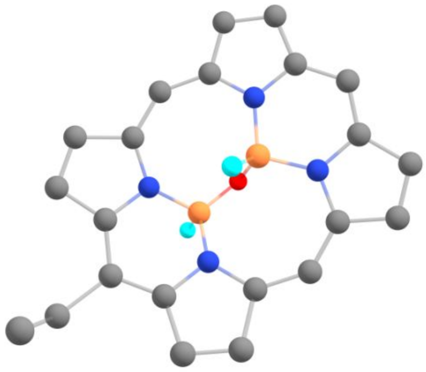

9	0.525589000	0.000041000	-1.231696000
5	-0.681608000	-0.000014000	1.175457000
5	1.351555000	0.000003000	-0.072825000
8	0.693862000	0.000001000	1.171449000
9	-1.249965000	-0.000148000	2.462432000
6	-1.951588000	3.344213000	-0.117463000
6	-0.810864000	2.502222000	0.194396000
6	0.502874000	3.026510000	0.130264000
6	1.810773000	2.533007000	-0.015658000
6	2.981931000	3.382940000	-0.130434000
6	4.078288000	2.581689000	-0.312323000
6	3.607427000	1.218413000	-0.317129000
6	4.294067000	-0.000023000	-0.421273000
6	3.607392000	-1.218446000	-0.317145000
6	4.078242000	-2.581722000	-0.312332000
6	2.981874000	-3.382963000	-0.130436000
6	1.810729000	-2.533020000	-0.015665000
6	0.502822000	-3.026511000	0.130258000
6	-0.810903000	-2.502222000	0.194406000
6	-1.951641000	-3.344189000	-0.117431000
6	-3.070253000	-2.555369000	-0.159713000
6	-2.644423000	-1.215630000	0.147586000
6	-3.357113000	0.000031000	0.087025000
6	-2.644408000	1.215683000	0.147652000
6	-3.070220000	2.555420000	-0.159669000
7	-1.295335000	1.236598000	0.400709000
7	2.252197000	1.243287000	-0.147927000
7	2.252158000	-1.243304000	-0.147970000
7	-1.295324000	-1.236529000	0.400509000
1	-1.884407000	4.400060000	-0.344693000
1	2.960073000	4.463164000	-0.071897000
1	5.110141000	2.885617000	-0.423897000

1	5.110091000	-2.885660000	-0.423908000
1	2.960007000	-4.463186000	-0.071897000
1	-4.075459000	2.856554000	-0.418211000
1	0.486926000	4.113457000	0.089618000
1	5.369646000	-0.000046000	-0.551813000
1	0.486867000	-4.113458000	0.089605000
1	-1.884507000	-4.400059000	-0.344574000
1	-4.075478000	-2.856535000	-0.418273000
6	-4.836482000	0.000116000	-0.223256000
1	-5.304005000	-0.874207000	0.240937000
1	-5.303849000	0.874584000	0.240830000
6	-5.129935000	-0.000039000	-1.737244000
1	-4.697658000	-0.883489000	-2.217449000
1	-4.697579000	0.883218000	-2.217720000
1	-6.209479000	-0.000024000	-1.921768000

E= –1392.5682093 a.u.
H= –1392.5682093 + 0.375563 (thermal correction 6-31+G**) a. u.
G= –1392.5682093 + 0.303875 (thermal correction 6-31+G**) a. u.

4b – akamptisomer *parvo,amplo* (***t***$_2$)

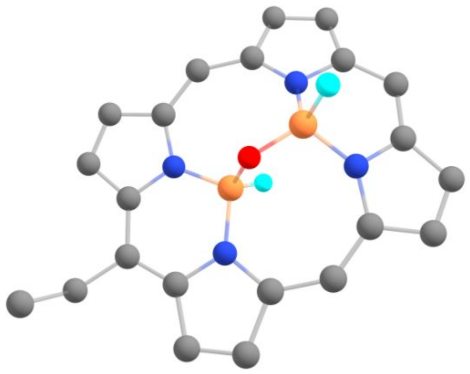

9	-2.235595000	-0.000053000	2.256117000
5	0.496733000	0.000026000	-0.136767000
5	-1.600364000	-0.000021000	1.001603000
8	-0.226330000	0.000019000	1.070716000
9	-0.266177000	0.000021000	-1.337960000
6	2.121947000	3.380060000	-0.119443000
6	0.951761000	2.528473000	-0.060822000
6	-0.365742000	3.026489000	0.010830000
6	-1.677602000	2.507287000	0.011701000
6	-2.804317000	3.347536000	-0.364653000
6	-3.915037000	2.555952000	-0.471519000
6	-3.500769000	1.218402000	-0.136783000
6	-4.187404000	-0.000058000	-0.236440000
6	-3.500725000	-1.218493000	-0.136792000
6	-3.914958000	-2.556059000	-0.471509000
6	-2.804212000	-3.347610000	-0.364660000
6	-1.677520000	-2.507326000	0.011689000
6	-0.365643000	-3.026479000	0.010809000
6	0.951840000	-2.528409000	-0.060847000
6	2.122055000	-3.379956000	-0.119486000
6	3.228938000	-2.580530000	-0.245371000
6	2.765513000	-1.215493000	-0.269467000
6	3.476867000	0.000074000	-0.342258000
6	2.765479000	1.215620000	-0.269451000
6	3.228859000	2.580674000	-0.245331000

7	1.401179000	1.240886000	-0.164653000
7	-2.171368000	1.239974000	0.196108000
7	-2.171319000	-1.240024000	0.196084000
7	1.401212000	-1.240806000	-0.164664000
1	2.095654000	4.460444000	-0.066576000
1	-2.725481000	4.403179000	-0.589241000
1	-4.908525000	2.843552000	-0.787739000
1	-4.908442000	-2.843692000	-0.787715000
1	-2.725347000	-4.403250000	-0.589247000
1	4.260160000	2.897808000	-0.306566000
1	-0.374983000	4.113015000	-0.041399000
1	-5.235977000	-0.000077000	-0.510865000
1	-0.374840000	-4.113005000	-0.041422000
1	2.095799000	-4.460342000	-0.066636000
1	4.260253000	-2.897618000	-0.306608000
6	4.986821000	0.000069000	-0.400623000
1	5.330890000	-0.874072000	-0.962982000
1	5.330909000	0.874380000	-0.962713000
6	5.634043000	-0.000173000	0.998780000
1	5.329587000	-0.884130000	1.568108000
1	5.329530000	0.883531000	1.568470000
1	6.726459000	-0.000117000	0.919558000

E= –1392.5679802 a.u.
H= –1392.5679802 + 0.375534 (thermal correction 6-31+G**) a. u.
G= –1392.5679802 + 0.303206 (thermal correction 6-31+G**) a. u.

4b – transition state (*TS*)

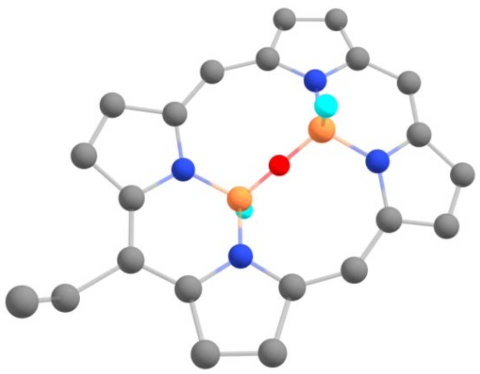

9	1.333846000	0.000000000	-2.127180000
5	-0.647409000	0.000000000	0.834612000
5	1.523052000	0.000000000	-0.724308000
8	0.437358000	0.000000000	0.055087000
9	-0.460010000	0.000000000	2.238042000
6	-2.058289000	3.342975000	-0.065167000
6	-0.912679000	2.472751000	0.153450000
6	0.439417000	2.939977000	0.070270000
6	1.784133000	2.475798000	-0.027346000
6	2.933938000	3.343454000	0.203225000
6	4.078773000	2.588230000	0.138590000
6	3.673368000	1.232114000	-0.161892000
6	4.366983000	0.000000000	-0.161000000
6	3.673368000	-1.232114000	-0.161892000
6	4.078773000	-2.588230000	0.138590000
6	2.933938000	-3.343454000	0.203225000
6	1.784133000	-2.475798000	-0.027346000
6	0.439417000	-2.939977000	0.070270000
6	-0.912679000	-2.472751000	0.153450000

6	-2.058289000	-3.342975000	-0.065167000
6	-3.205676000	-2.587658000	-0.001500000
6	-2.803302000	-1.229356000	0.284226000
6	-3.516670000	0.000000000	0.281229000
6	-2.803302000	1.229356000	0.284226000
6	-3.205676000	2.587658000	-0.001500000
7	-1.447766000	1.239575000	0.416277000
7	2.321249000	1.242913000	-0.305237000
7	2.321249000	-1.242913000	-0.305237000
7	-1.447766000	-1.239575000	0.416277000
1	-1.989249000	4.397698000	-0.297738000
1	2.864086000	4.395791000	0.446322000
1	5.093015000	2.917137000	0.320136000
1	5.093015000	-2.917137000	0.320136000
1	2.864086000	-4.395791000	0.446322000
1	-4.217254000	2.927063000	-0.175292000
1	0.438860000	4.029409000	0.086302000
1	5.443567000	0.000000000	-0.035156000
1	0.438860000	-4.029409000	0.086302000
1	-1.989249000	-4.397698000	-0.297738000
1	-4.217254000	-2.927063000	-0.175292000
6	-5.014748000	0.000000000	0.083346000
1	-5.446536000	-0.875688000	0.579257000
1	-5.446536000	0.875688000	0.579257000
6	-5.418906000	0.000000000	-1.405493000
1	-5.023384000	-0.883533000	-1.916223000
1	-5.023384000	0.883533000	-1.916223000
1	-6.509151000	0.000000000	-1.509657000

E= –1392.5232676 a.u.
H= –1392.5232676 + 0.373859 (thermal correction 6-31+G**) a. u.
G= –1392.5232676 + 0.302487 (thermal correction 6-31+G**) a. u.
f= 185.0745 i

4b– akamptisomer *amplo, amplo* (c_1)

9	-2.838301000	-0.000048000	1.802059000
5	0.766122000	-0.000051000	1.152265000
5	-1.796620000	0.000006000	0.843982000
8	-0.569905000	0.000092000	1.452138000
9	1.552252000	-0.000127000	2.329880000
6	2.012262000	-3.374736000	-0.004395000
6	0.867386000	-2.537498000	0.278527000
6	-0.431195000	-3.073700000	0.266673000
6	-1.693797000	-2.542909000	-0.025344000
6	-2.747175000	-3.378347000	-0.567298000
6	-3.740782000	-2.558449000	-1.029824000

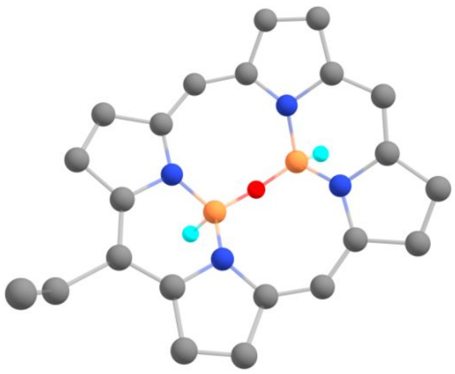

6	-3.333723000	-1.212706000	-0.741274000
6	-3.965247000	0.000083000	-1.028747000
6	-3.333707000	1.212865000	-0.741228000
6	-3.740678000	2.558628000	-1.029796000
6	-2.747013000	3.378462000	-0.567266000
6	-1.693703000	2.542956000	-0.025291000
6	-0.431058000	3.073671000	0.266704000
6	0.867507000	2.537430000	0.278584000
6	2.012425000	3.374626000	-0.004275000
6	3.091971000	2.557966000	-0.212758000
6	2.637457000	1.210059000	-0.023897000
6	3.341357000	-0.000094000	-0.152389000
6	2.637396000	-1.210189000	-0.023940000
6	3.091846000	-2.558120000	-0.212813000
7	1.293711000	-1.234558000	0.297476000
7	-2.107374000	-1.237181000	-0.110521000
7	-2.107387000	1.237259000	-0.110448000
7	1.293802000	1.234470000	0.297528000
1	1.970919000	-4.451220000	-0.106093000
1	-2.687415000	-4.455539000	-0.651172000
1	-4.651926000	-2.839266000	-1.540501000
1	-4.651796000	2.839505000	-1.540487000
1	-2.687179000	4.455648000	-0.651151000
1	4.091911000	-2.854183000	-0.495713000
1	-0.428512000	-4.160647000	0.268377000
1	-4.931781000	0.000094000	-1.518883000
1	-0.428328000	4.160619000	0.268404000
1	1.971128000	4.451116000	-0.105930000
1	4.092047000	2.853984000	-0.495665000
6	4.804774000	-0.000092000	-0.531338000
1	5.295037000	0.872889000	-0.089336000
1	5.294962000	-0.873294000	-0.089691000
6	5.025459000	0.000198000	-2.056959000
1	4.571321000	0.884243000	-2.515966000
1	4.571238000	-0.883626000	-2.516309000
1	6.094722000	0.000193000	-2.293862000

E= –1392.5577072 a.u.
H= –1392.5577072 + 0.375288 (thermal correction 6-31+G**) a. u.
G= –1392.5577072 + 0.302574 (thermal correction 6-31+G**) a. u.

4c – akamptisomer *amplo,parvo* ($\boldsymbol{t}_1$)

9	-0.795828000	-0.000963000	-1.266956000
5	0.504678000	-0.069223000	1.093674000
5	-1.575044000	0.038423000	-0.076902000
8	-0.868478000	0.004558000	1.139765000
9	1.116476000	-0.099662000	2.361524000

6	1.545070000	-3.470798000	-0.238800000
6	0.464009000	-2.568913000	0.109097000
6	-0.876238000	-3.024917000	0.094236000
6	-2.162060000	-2.466369000	-0.001669000
6	-3.378256000	-3.255859000	-0.072519000
6	-4.438410000	-2.399592000	-0.212723000
6	-3.899331000	-1.061883000	-0.234974000
6	-4.526627000	0.189609000	-0.313294000
6	-3.774534000	1.371001000	-0.234681000
6	-4.172827000	2.756927000	-0.210088000
6	-3.029776000	3.499106000	-0.067668000
6	-1.901889000	2.588279000	0.001495000
6	-0.564877000	3.009332000	0.100091000
6	0.721946000	2.418674000	0.112854000
6	1.886552000	3.209713000	-0.232020000
6	2.966464000	2.373579000	-0.311741000
6	2.498322000	1.047505000	0.005428000
6	3.149296000	-0.207896000	-0.059180000
6	2.366168000	-1.382537000	0.010782000
6	2.703891000	-2.746405000	-0.311743000
7	1.024159000	-1.331971000	0.299116000
7	-2.541508000	-1.156054000	-0.116414000
7	-2.414785000	1.324553000	-0.116449000
7	1.156713000	1.130917000	0.296176000
1	1.413157000	-4.520031000	-0.468110000
1	-3.408971000	-4.335923000	-0.015175000
1	-5.487880000	-2.650302000	-0.285592000
1	-5.190745000	3.114943000	-0.282414000
1	-2.949015000	4.576474000	-0.008008000
1	3.677318000	-3.110156000	-0.606696000
1	-0.916816000	-4.111222000	0.053858000
1	-5.604981000	0.244734000	-0.402947000
1	-0.492950000	4.094186000	0.064463000
1	1.861049000	4.268291000	-0.454752000
1	3.969599000	2.643406000	-0.602129000
6	4.646104000	-0.330285000	-0.307874000
1	4.893830000	-1.390500000	-0.201062000
6	5.481506000	0.406518000	0.759037000
1	6.544084000	0.178388000	0.621025000
1	5.361728000	1.491265000	0.700779000
1	5.189571000	0.093949000	1.766236000
6	5.048517000	0.071370000	-1.742541000
1	6.112270000	-0.135106000	-1.904418000
1	4.471691000	-0.492987000	-2.482055000
1	4.880704000	1.134675000	-1.932116000

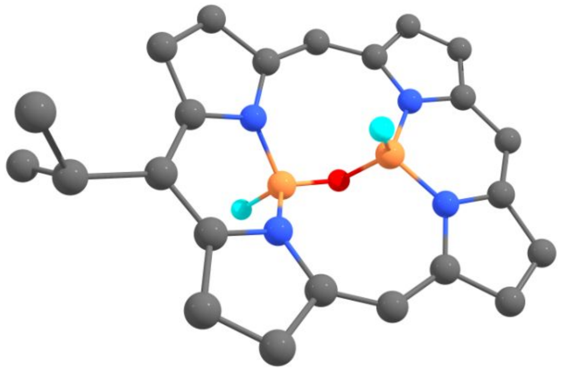

E= –1431.9000721 a.u.

H= –1431.9000721 + 0.405257 (thermal correction 6-31+G**) a. u.
G= –1431.9000721 + 0.331131 (thermal correction 6-31+G**) a. u.

4c – akamptisomer *parvo,amplo* (***t*₂**)

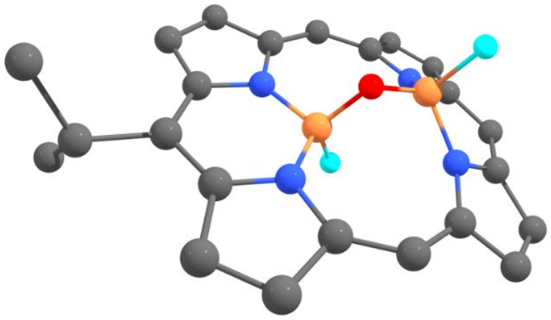

9	2.559668000	0.078568000	2.206841000
5	-0.294885000	-0.058678000	-0.038187000
5	1.861768000	0.048356000	0.985947000
8	0.494871000	-0.019850000	1.126439000
9	0.401890000	-0.017023000	-1.278949000
6	-1.728502000	-3.518757000	0.053904000
6	-0.607348000	-2.604091000	0.054047000
6	0.735753000	-3.033778000	0.053371000
6	2.017452000	-2.449356000	-0.014089000
6	3.164425000	-3.231276000	-0.450818000
6	4.226361000	-2.384145000	-0.611779000
6	3.762257000	-1.069432000	-0.252227000
6	4.380573000	0.181663000	-0.382489000
6	3.638203000	1.363287000	-0.243914000
6	3.963940000	2.720647000	-0.595741000
6	2.819927000	3.452674000	-0.429808000
6	1.760438000	2.554137000	0.002113000
6	0.424704000	3.001102000	0.071522000
6	-0.868085000	2.437180000	0.070434000
6	-2.074262000	3.235184000	0.076406000
6	-3.148697000	2.388302000	0.009151000
6	-2.633117000	1.040123000	-0.044124000
6	-3.285428000	-0.215168000	-0.088298000
6	-2.501300000	-1.390295000	-0.054330000
6	-2.882448000	-2.783040000	-0.010177000
7	-1.132654000	-1.343258000	-0.020442000
7	2.454115000	-1.159034000	0.147903000
7	2.328674000	1.315007000	0.157043000
7	-1.265386000	1.130410000	-0.011314000
1	-1.640213000	-4.596039000	0.102234000
1	3.127904000	-4.289444000	-0.674289000
1	5.215735000	-2.620343000	-0.979306000
1	4.923359000	3.060168000	-0.961746000
1	2.674195000	4.502648000	-0.647287000
1	-3.891930000	-3.166911000	-0.016218000
1	0.796851000	-4.118329000	-0.003857000
1	5.412523000	0.234930000	-0.709661000
1	0.373510000	4.086509000	0.019763000
1	-2.094570000	4.315571000	0.130628000
1	-4.189705000	2.669947000	0.005281000
6	-4.802354000	-0.333163000	-0.143357000
1	-5.032166000	-1.400784000	-0.200702000
6	-5.401069000	0.299054000	-1.417016000

1	-5.269839000	1.383767000	-1.439712000
1	-4.929119000	-0.111770000	-2.315107000
1	-6.475272000	0.089968000	-1.467577000
6	-5.486156000	0.185624000	1.139084000
1	-6.560932000	-0.023504000	1.100019000
1	-5.073335000	-0.304381000	2.026434000
1	-5.359239000	1.263774000	1.266233000

E= –1431.8996633 a.u.
H= –1431.8996633 + 0.405268 (thermal correction 6-31+G**) a. u.
G= –1431.8996633 + 0.331042 (thermal correction 6-31+G**) a. u.

4c – transition state (*TS*)

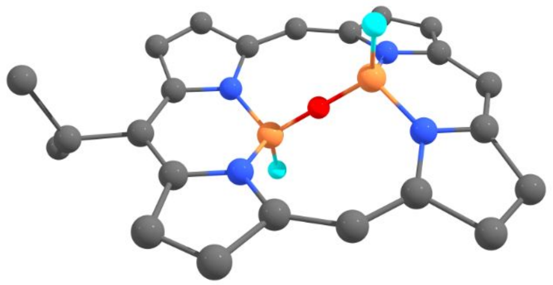

9	1.653450000	-0.027082000	-2.130684000
5	-0.462357000	0.063238000	0.735296000
5	1.776972000	-0.040365000	-0.720613000
8	0.656533000	0.004438000	0.007118000
9	-0.339173000	0.050778000	2.146648000
6	-1.661788000	3.473670000	-0.197326000
6	-0.571969000	2.545152000	0.056288000
6	0.802892000	2.946638000	0.035660000
6	2.126308000	2.418387000	-0.002751000
6	3.305179000	3.229313000	0.281886000
6	4.413694000	2.419655000	0.265706000
6	3.956971000	1.084960000	-0.056574000
6	4.590294000	-0.178169000	-0.028430000
6	3.838781000	-1.375640000	-0.065162000
6	4.162630000	-2.749197000	0.253662000
6	2.979841000	-3.446640000	0.266527000
6	1.886634000	-2.523589000	-0.016683000
6	0.517402000	-2.918692000	0.022350000
6	-0.813490000	-2.388034000	0.044334000
6	-1.983580000	-3.210300000	-0.212066000
6	-3.099234000	-2.408578000	-0.195007000
6	-2.657591000	-1.061347000	0.099940000
6	-3.310672000	0.207391000	0.088077000
6	-2.529157000	1.397242000	0.112809000
6	-2.847657000	2.779408000	-0.177872000
7	-1.179005000	1.339180000	0.286914000
7	2.614059000	1.161418000	-0.259524000
7	2.496268000	-1.319873000	-0.270825000
7	-1.307006000	-1.130796000	0.276845000
1	-1.530862000	4.524387000	-0.421162000
1	3.275423000	4.283544000	0.524925000
1	5.433679000	2.698751000	0.492833000
1	5.150018000	-3.127342000	0.481461000
1	2.846747000	-4.493541000	0.506616000

1	-3.829407000	3.179036000	-0.388250000
1	0.853834000	4.034700000	0.057940000
1	5.658665000	-0.229770000	0.147045000
1	0.462500000	-4.006665000	0.044119000
1	-1.951855000	-4.268496000	-0.436665000
1	-4.111207000	-2.719271000	-0.405068000
6	-4.818939000	0.326416000	-0.070181000
1	-5.063814000	1.386807000	0.047957000
6	-5.588123000	-0.419365000	1.038915000
1	-6.660624000	-0.213747000	0.952321000
1	-5.448260000	-1.501817000	0.976902000
1	-5.255444000	-0.097039000	2.030462000
6	-5.296651000	-0.083696000	-1.479424000
1	-6.368929000	0.114996000	-1.585138000
1	-4.763979000	0.480194000	-2.251554000
1	-5.131177000	-1.147266000	-1.670703000

E= –1431.8557867 a.u.
H= –1431.8557867 + 0.403528 (thermal correction 6-31+G**) a. u.
G= –1431.8557867 + 0.329666 (thermal correction 6-31+G**) a. u.
f = 182.5223 i

4c– akamptisomer *amplo, amplo* (c_1)

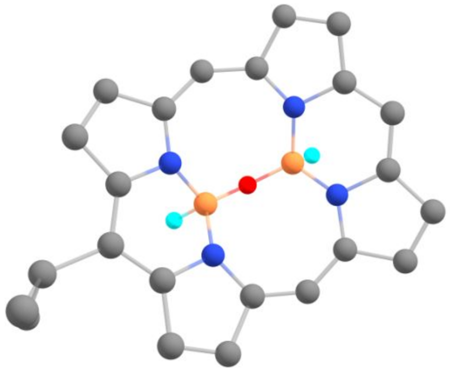

9	-2.998594000	-0.105167000	1.847889000
5	0.579506000	0.083037000	1.083436000
5	-1.992509000	-0.058092000	0.853134000
8	-0.746934000	0.011356000	1.417131000
9	1.393219000	0.134636000	2.242698000
6	1.960692000	-3.231760000	-0.070805000
6	0.786657000	-2.446057000	0.229733000
6	-0.481843000	-3.049823000	0.259289000
6	-1.780735000	-2.595686000	0.000583000
6	-2.801504000	-3.493803000	-0.501527000
6	-3.854077000	-2.734809000	-0.937567000
6	-3.514426000	-1.365878000	-0.671674000
6	-4.220527000	-0.191571000	-0.946836000
6	-3.646188000	1.055384000	-0.688444000
6	-4.132991000	2.375468000	-0.973266000
6	-3.169531000	3.250636000	-0.550180000
6	-2.056555000	2.476308000	-0.036731000
6	-0.815193000	3.073601000	0.213292000
6	0.510838000	2.609094000	0.190861000
6	1.595518000	3.509469000	-0.125662000
6	2.716485000	2.757850000	-0.352367000
6	2.352354000	1.382811000	-0.142229000
6	3.127858000	0.213378000	-0.272220000
6	2.489532000	-1.036671000	-0.127488000

6	2.995165000	-2.369506000	-0.312635000
7	1.153468000	-1.124633000	0.224095000
7	-2.269498000	-1.316758000	-0.081072000
7	-2.403048000	1.150757000	-0.098553000
7	1.015205000	1.334145000	0.208340000
1	1.968617000	-4.309897000	-0.161878000
1	-2.683870000	-4.566654000	-0.579641000
1	-4.763712000	-3.070845000	-1.416613000
1	-5.073820000	2.603263000	-1.455765000
1	-3.169541000	4.328668000	-0.643902000
1	3.996034000	-2.633977000	-0.614847000
1	-0.418613000	-4.134896000	0.272631000
1	-5.200938000	-0.247927000	-1.405074000
1	-0.869892000	4.159169000	0.208189000
1	1.488081000	4.580369000	-0.237081000
1	3.684444000	3.120794000	-0.664781000
6	4.611274000	0.332503000	-0.596572000
1	4.846243000	1.400062000	-0.598395000
6	4.962968000	-0.187043000	-2.006165000
1	6.016940000	0.015270000	-2.226291000
1	4.802565000	-1.263825000	-2.102025000
1	4.352697000	0.308667000	-2.768013000
6	5.503720000	-0.291098000	0.496829000
1	6.554947000	-0.056879000	0.295801000
1	5.243621000	0.104980000	1.483001000
1	5.405221000	-1.378498000	0.540514000

E= –1431.8891876 a.u.
H= –1431.8891876 + 0.404884 (thermal correction 6-31+G**) a. u.
G= –1431.8891876 + 0.329868 (thermal correction 6-31+G**) a. u.

4d – akamptisomer *amplo,parvo* (***t***$_1$)

9	1.039255000	0.004486000	-1.293311000
5	-0.347005000	0.015659000	1.031185000
5	1.780428000	-0.003544000	-0.078453000
8	1.027723000	0.006049000	1.110397000
9	-0.986207000	0.010671000	2.287163000
6	-1.462830000	3.351078000	-0.402909000
6	-0.373649000	2.491869000	0.006743000
6	0.944710000	3.006774000	0.032136000
6	2.260105000	2.515467000	-0.001869000
6	3.439883000	3.360499000	-0.031786000
6	4.542821000	2.552123000	-0.119070000
6	4.066417000	1.190953000	-0.150569000
6	4.748642000	-0.032074000	-0.198361000
6	4.041806000	-1.242988000	-0.150559000
6	4.493504000	-2.611661000	-0.119470000

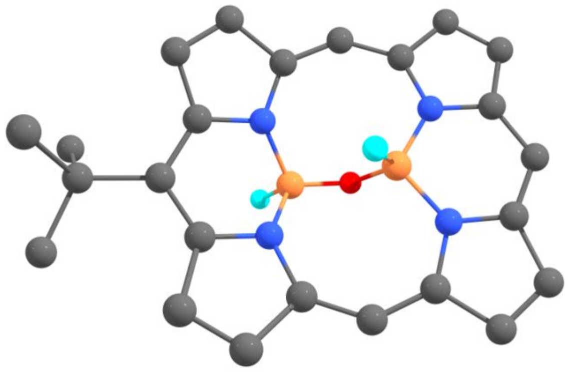

6	3.375539000	-3.400345000	-0.033489000
6	2.212275000	-2.534674000	-0.004666000
6	0.887897000	-3.002412000	0.027673000
6	-0.418643000	-2.461751000	0.001863000
6	-1.530573000	-3.297042000	-0.396587000
6	-2.650685000	-2.518034000	-0.463824000
6	-2.266462000	-1.176738000	-0.086525000
6	-3.004916000	0.043910000	-0.088472000
6	-2.258276000	1.253874000	-0.074556000
6	-2.603110000	2.603426000	-0.466035000
7	-0.911170000	1.247943000	0.224585000
7	2.701894000	1.225111000	-0.087043000
7	2.677256000	-1.251425000	-0.087850000
7	-0.924580000	-1.201135000	0.209403000
1	-1.349892000	4.391071000	-0.679342000
1	3.418899000	4.441270000	0.015246000
1	5.581939000	2.850073000	-0.151678000
1	5.527055000	-2.928388000	-0.151648000
1	3.335002000	-4.480607000	0.012707000
1	-3.560531000	2.949708000	-0.810874000
1	0.933544000	4.092754000	-0.029876000
1	5.831023000	-0.043078000	-0.245530000
1	0.856541000	-4.088222000	-0.031078000
1	-1.445749000	-4.342856000	-0.660809000
1	-3.625403000	-2.839125000	-0.791802000
6	-4.552546000	-0.008579000	-0.142678000
6	-5.058090000	-0.991551000	0.951187000
1	-6.152012000	-1.038475000	0.919631000
1	-4.677513000	-2.005237000	0.839425000
1	-4.758357000	-0.636441000	1.942527000
6	-5.041847000	-0.443058000	-1.547333000
1	-4.682197000	-1.431174000	-1.836784000
1	-6.137428000	-0.462955000	-1.567024000
1	-4.700010000	0.268538000	-2.306315000
6	-5.246348000	1.336980000	0.181462000
1	-6.300780000	1.140598000	0.398799000
1	-4.808294000	1.826059000	1.055849000
1	-5.229729000	2.031376000	-0.660434000

E= –1471.2223524 a.u.
H= –1471.2223524 + 0.434568 (thermal correction 6-31+G**) a. u.
G= –1471.2223524 + 0.358241 (thermal correction 6-31+G**) a. u.

4d – akamptisomer *parvo,amplo* (***t***$_{\mathbf{2}}$)

9	-2.775946000	0.016547000	2.191710000
5	0.113612000	-0.023162000	-0.022725000

5	-2.070546000	0.008001000	0.973681000
8	-0.702937000	-0.015834000	1.125075000
9	-0.567040000	-0.015232000	-1.274065000
6	1.733531000	3.338814000	0.128716000
6	0.570444000	2.486614000	0.103707000
6	-0.741163000	3.001539000	0.087212000
6	-2.059400000	2.511754000	-0.004065000
6	-3.144653000	3.372246000	-0.446154000
6	-4.259285000	2.598334000	-0.625889000
6	-3.887569000	1.253589000	-0.273024000
6	-4.585796000	0.045535000	-0.418759000
6	-3.922240000	-1.179993000	-0.279373000
6	-4.327656000	-2.514144000	-0.639510000
6	-3.233584000	-3.316432000	-0.463604000
6	-2.125900000	-2.485432000	-0.015960000
6	-0.821527000	-3.010435000	0.079527000
6	0.505394000	-2.533672000	0.101476000
6	1.637735000	-3.424750000	0.135891000
6	2.777138000	-2.674239000	0.076534000
6	2.389559000	-1.275707000	-0.001410000
6	3.132437000	-0.062698000	-0.051579000
6	2.403287000	1.165290000	-0.001593000
6	2.844683000	2.544925000	0.066630000
7	1.032142000	1.199012000	0.020872000
7	-2.580951000	1.252794000	0.140005000
7	-2.615676000	-1.216012000	0.134609000
7	1.010349000	-1.264064000	0.017267000
1	1.704947000	4.418516000	0.190756000
1	-3.035273000	4.427484000	-0.659410000
1	-5.226775000	2.902984000	-1.001205000
1	-5.302279000	-2.792236000	-1.017032000
1	-3.150638000	-4.372949000	-0.682317000
1	3.862636000	2.894738000	0.077135000
1	-0.726465000	4.088359000	0.041224000
1	-5.615727000	0.061072000	-0.756055000
1	-0.836735000	-4.097102000	0.031682000
1	1.571661000	-4.502547000	0.203527000
1	3.773573000	-3.071485000	0.100165000
6	4.680759000	0.014190000	-0.147619000
6	5.411596000	-1.339556000	-0.277395000
1	5.319832000	-1.947219000	0.625714000
1	5.070470000	-1.913995000	-1.142597000
1	6.478291000	-1.138967000	-0.418328000
6	5.078990000	0.807578000	-1.424344000
1	6.169430000	0.901436000	-1.473309000
1	4.744711000	0.269369000	-2.317806000
1	4.653856000	1.808375000	-1.471540000

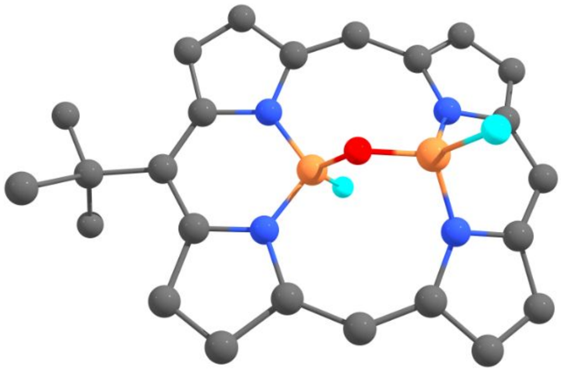

6	5.257018000	0.673072000	1.135893000
1	5.018722000	0.058745000	2.010552000
1	6.347775000	0.742933000	1.057038000
1	4.869192000	1.672496000	1.326506000

E= –1471.2209278 a.u.
H= –1471.2209278 + 0.434898 (thermal correction 6-31+G**) a. u.
G= –1471.2209278 + 0.358772 (thermal correction 6-31+G**) a. u.

4d – transition state (*TS*)

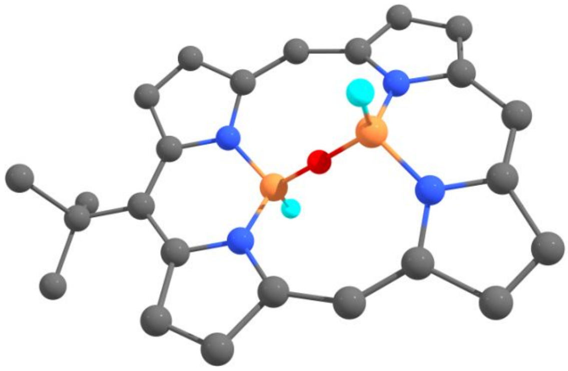

9	1.922820000	-0.010333000	-2.132492000
5	-0.290365000	0.018201000	0.663950000
5	2.000069000	-0.005746000	-0.718860000
8	0.853700000	0.019574000	-0.030041000
9	-0.206546000	0.005508000	2.079138000
6	-1.567706000	3.374112000	-0.315773000
6	-0.467059000	2.474899000	-0.031296000
6	0.891361000	2.927399000	-0.012104000
6	2.237757000	2.459098000	-0.004206000
6	3.371967000	3.318550000	0.316686000
6	4.512372000	2.554144000	0.339285000
6	4.120473000	1.201509000	0.006394000
6	4.797160000	-0.036843000	0.063613000
6	4.089255000	-1.260217000	0.009056000
6	4.454156000	-2.620420000	0.337031000
6	3.298443000	-3.362468000	0.311650000
6	2.182811000	-2.480304000	-0.006237000
6	0.828027000	-2.922294000	-0.018333000
6	-0.517863000	-2.439296000	-0.039647000
6	-1.644803000	-3.309383000	-0.313354000
6	-2.792994000	-2.558744000	-0.303683000
6	-2.421425000	-1.187693000	0.001030000
6	-3.152745000	0.051108000	0.025147000
6	-2.410949000	1.277554000	0.012723000
6	-2.737380000	2.659999000	-0.305206000
7	-1.054408000	1.256225000	0.194902000
7	2.783172000	1.224586000	-0.241714000
7	2.751450000	-1.253788000	-0.235789000
7	-1.070085000	-1.201507000	0.177398000
1	-1.457589000	4.424058000	-0.553915000
1	3.291291000	4.371052000	0.555536000
1	5.512139000	2.874464000	0.599531000
1	5.447630000	-2.961528000	0.594860000
1	3.197045000	-4.414215000	0.545904000
1	-3.706444000	3.059405000	-0.544819000
1	0.895999000	4.016818000	0.005753000
1	5.860395000	-0.050440000	0.273947000

1	0.809917000	-4.011507000	-0.000556000
1	-1.565826000	-4.364319000	-0.541449000
1	-3.781396000	-2.925873000	-0.527914000
6	-4.699134000	-0.008760000	0.015622000
6	-5.393094000	1.348005000	0.270724000
1	-4.977377000	1.866688000	1.139178000
1	-5.344532000	2.007931000	-0.597696000
1	-6.454427000	1.165314000	0.465688000
6	-5.212780000	-0.523087000	-1.354432000
1	-6.308314000	-0.551463000	-1.351421000
1	-4.891066000	0.148306000	-2.157443000
1	-4.850523000	-1.522566000	-1.595754000
6	-5.175261000	-0.937849000	1.167097000
1	-4.873284000	-0.524299000	2.135107000
1	-6.268447000	-1.007580000	1.152584000
1	-4.774224000	-1.948412000	1.103652000

E= –1471.1796147 a.u.
H= –1471.1796147 + 0.432945 (thermal correction 6-31+G**) a. u.
G= –1471.1796147 + 0.357060 (thermal correction 6-31+G**) a. u.
f = 178.0317 i

4d– akamptisomer *amplo, amplo* (c_1)

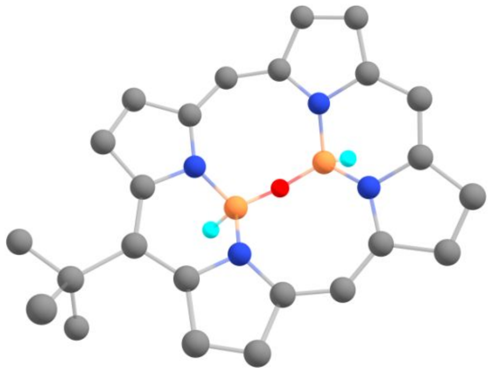

9	-3.157096000	-0.023712000	1.886347000
5	0.412699000	0.022796000	1.051422000
5	-2.187631000	-0.010165000	0.853858000
8	-0.919962000	0.011346000	1.367728000
9	1.214483000	0.020167000	2.221782000
6	1.606621000	-3.328201000	-0.191381000
6	0.483639000	-2.487728000	0.139099000
6	-0.809241000	-3.036466000	0.171561000
6	-2.100806000	-2.539613000	-0.039184000
6	-3.163856000	-3.395424000	-0.526708000
6	-4.207901000	-2.595409000	-0.907544000
6	-3.816407000	-1.243745000	-0.625825000
6	-4.485505000	-0.039927000	-0.868050000
6	-3.848242000	1.179086000	-0.627170000
6	-4.271448000	2.522031000	-0.909506000
6	-3.246749000	3.346025000	-0.529998000
6	-2.162713000	2.515232000	-0.041304000
6	-0.884382000	3.043551000	0.171489000
6	0.424368000	2.530186000	0.138831000
6	1.515744000	3.404038000	-0.209793000
6	2.627450000	2.637921000	-0.406689000
6	2.265830000	1.263724000	-0.146106000
6	3.007334000	0.057152000	-0.232023000

6	2.276238000	-1.162683000	-0.152471000
6	2.691574000	-2.523107000	-0.391171000
7	0.931915000	-1.190345000	0.178476000
7	-2.552693000	-1.247147000	-0.075809000
7	-2.584542000	1.213682000	-0.076901000
7	0.916710000	1.251866000	0.193250000
1	1.551728000	-4.399865000	-0.329618000
1	-3.081736000	-4.469142000	-0.632930000
1	-5.143088000	-2.892947000	-1.362207000
1	-5.213538000	2.797371000	-1.363944000
1	-3.189813000	4.421322000	-0.636679000
1	3.667329000	-2.853226000	-0.704194000
1	-0.784050000	-4.123101000	0.152705000
1	-5.480725000	-0.053267000	-1.296663000
1	-0.886758000	4.130269000	0.146448000
1	1.422071000	4.470624000	-0.365355000
1	3.577545000	3.003974000	-0.746108000
6	4.549419000	-0.012703000	-0.404907000
6	5.292200000	1.339258000	-0.301551000
1	5.131147000	1.971552000	-1.177358000
1	5.023820000	1.892198000	0.602457000
1	6.366484000	1.135216000	-0.258384000
6	5.144308000	-0.875145000	0.745319000
1	6.230880000	-0.945046000	0.624128000
1	4.936944000	-0.402896000	1.711010000
1	4.746503000	-1.887040000	0.789559000
6	4.914955000	-0.589737000	-1.798236000
1	4.528503000	0.063405000	-2.587992000
1	6.004612000	-0.640193000	-1.902820000
1	4.515705000	-1.587516000	-1.976656000

E= –1471.2098275 a.u.
H= –1471.2098275 + 0.434479 (thermal correction 6-31+G**) a. u.
G= –1471.2098275 + 0.357687 (thermal correction 6-31+G**) a. u.

4e – akamptisomer *amplo,parvo* (***t***$_1$)

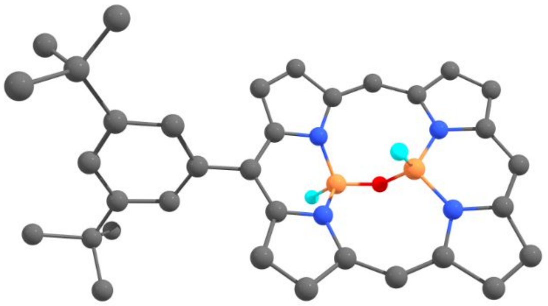

9	2.731771000	0.409015000	1.146960000
5	1.627651000	-0.425284000	-1.159506000
5	3.606166000	-0.002000000	0.102137000
8	3.001399000	-0.436113000	-1.092734000
9	1.111220000	-0.858706000	-2.395636000
6	0.228385000	-3.081048000	1.181502000
6	1.399443000	-2.423239000	0.631045000
6	2.696580000	-2.898454000	0.939012000
6	4.008966000	-2.394832000	0.962222000
6	5.153742000	-3.157029000	1.426100000
6	6.259864000	-2.350505000	1.366596000

6	5.821117000	-1.070426000	0.867852000
6	6.530024000	0.102315000	0.569626000
6	5.876932000	1.209552000	0.008718000
6	6.380655000	2.476858000	-0.459698000
6	5.313062000	3.167231000	-0.970813000
6	4.127766000	2.341557000	-0.831198000
6	2.841129000	2.758314000	-1.216243000
6	1.519337000	2.257896000	-1.153784000
6	0.385206000	3.156494000	-1.263927000
6	-0.755182000	2.447670000	-0.995187000
6	-0.346396000	1.094040000	-0.725291000
6	-1.087459000	-0.020838000	-0.271405000
6	-0.401286000	-1.189768000	0.132071000
6	-0.870620000	-2.315914000	0.895318000
7	0.952726000	-1.313654000	-0.041619000
7	4.474349000	-1.144905000	0.651902000
7	4.530655000	1.182526000	-0.222989000
7	1.014163000	1.010549000	-0.886081000
1	0.262906000	-3.979677000	1.783350000
1	5.109283000	-4.188083000	1.751242000
1	7.278532000	-2.602310000	1.628274000
1	7.413919000	2.792810000	-0.413946000
1	5.320267000	4.154545000	-1.413501000
1	-1.891970000	-2.471477000	1.210802000
1	2.653502000	-3.898951000	1.364011000
1	7.598741000	0.141667000	0.743580000
1	2.853143000	3.783520000	-1.579706000
1	0.465432000	4.216716000	-1.465258000
1	-1.769675000	2.815622000	-0.960140000
6	-2.559111000	0.049453000	-0.102553000
6	-3.151589000	1.060942000	0.675283000
6	-3.368973000	-0.918687000	-0.702327000
1	-2.501969000	1.777748000	1.161133000
1	-2.892814000	-1.684501000	-1.305557000
6	-4.535386000	1.116066000	0.848374000
6	-4.764985000	-0.898294000	-0.550468000
6	-5.318449000	0.125739000	0.223716000
1	-6.391969000	0.162371000	0.357704000
6	-5.222296000	2.198249000	1.700944000
6	-5.973310000	1.525466000	2.875112000
1	-6.735613000	0.824125000	2.522192000
1	-5.277255000	0.971370000	3.514056000
1	-6.474091000	2.282266000	3.489825000
6	-4.217883000	3.207670000	2.288757000
1	-4.753579000	3.956811000	2.880890000
1	-3.491815000	2.722560000	2.949548000
1	-3.668172000	3.738182000	1.503570000

6	-6.232177000	2.979006000	0.825733000
1	-7.009884000	2.326122000	0.418160000
1	-6.726832000	3.755854000	1.419976000
1	-5.725226000	3.462811000	-0.016153000
6	-5.614479000	-1.985334000	-1.232797000
6	-5.173181000	-3.376273000	-0.716653000
1	-4.120539000	-3.577063000	-0.937291000
1	-5.311163000	-3.450214000	0.367673000
1	-5.769094000	-4.163914000	-1.191824000
6	-7.119649000	-1.823075000	-0.947898000
1	-7.675559000	-2.621489000	-1.450334000
1	-7.340559000	-1.889437000	0.123054000
1	-7.504064000	-0.867905000	-1.321722000
6	-5.402075000	-1.914753000	-2.764057000
1	-4.354622000	-2.071203000	-3.037879000
1	-5.997886000	-2.686204000	-3.265469000
1	-5.708135000	-0.938026000	-3.154049000

E= –1859.7385871 a.u.
H= –1859.7385871 + 0.638205 (thermal correction 6-31+G**) a. u.
G= –1859.7385871 + 0.538752 (thermal correction 6-31+G**) a. u.

4e – akamptisomer *parvo,amplo* (***t*₂**)

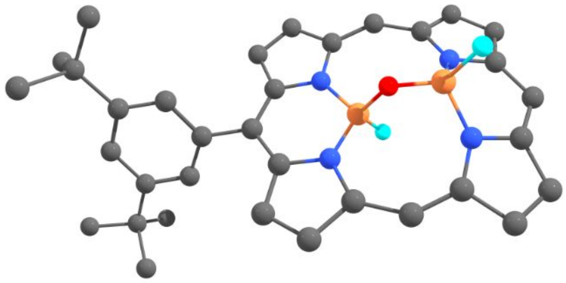

9	4.681966000	-0.735431000	-2.068114000
5	1.798969000	-0.010541000	0.019693000
5	3.967279000	-0.330112000	-0.926634000
8	2.600899000	-0.391266000	-1.072361000
9	2.481035000	0.405539000	1.197381000
6	0.156526000	3.101526000	-1.280713000
6	1.334000000	2.328257000	-0.945196000
6	2.648662000	2.811290000	-1.103754000
6	3.962010000	2.363745000	-0.846486000
6	5.055353000	3.314375000	-0.716072000
6	6.163716000	2.638949000	-0.282772000
6	5.782318000	1.255999000	-0.163746000
6	6.471148000	0.163275000	0.381220000
6	5.801486000	-1.035922000	0.663646000
6	6.203732000	-2.165160000	1.460410000
6	5.106901000	-2.975373000	1.571607000
6	4.000168000	-2.347809000	0.865882000
6	2.694433000	-2.871728000	0.976439000
6	1.370724000	-2.422335000	0.794470000
6	0.207800000	-3.228660000	1.102697000
6	-0.912950000	-2.471482000	0.878020000
6	-0.464646000	-1.180375000	0.418632000
6	-1.187282000	-0.031087000	0.024510000
6	-0.479269000	1.112136000	-0.412766000

6	-0.951058000	2.357231000	-0.966252000
7	0.887632000	1.145449000	-0.418575000
7	4.475904000	1.124708000	-0.557344000
7	4.495828000	-1.208055000	0.283210000
7	0.902377000	-1.197941000	0.400128000
1	0.179320000	4.090363000	-1.719335000
1	4.954171000	4.380623000	-0.870608000
1	7.133065000	3.045828000	-0.028699000
1	7.177737000	-2.298866000	1.911137000
1	5.021553000	-3.891204000	2.141657000
1	-1.988909000	2.618886000	-1.110404000
1	2.645587000	3.848383000	-1.432153000
1	7.500851000	0.285731000	0.696774000
1	2.709629000	-3.869230000	1.410092000
1	0.248470000	-4.251638000	1.452846000
1	-1.944896000	-2.759056000	1.014289000
6	-2.669835000	-0.024786000	0.038008000
6	-3.394131000	-1.026666000	-0.634112000
6	-3.362030000	0.988154000	0.707970000
1	-2.837261000	-1.787028000	-1.166866000
1	-2.787695000	1.739750000	1.239029000
6	-4.789838000	-1.021658000	-0.643069000
6	-4.765353000	1.031165000	0.717946000
6	-5.450029000	0.019833000	0.037543000
1	-6.532283000	0.031996000	0.027249000
6	-5.618899000	-2.090613000	-1.377662000
6	-6.450809000	-1.413624000	-2.493363000
1	-7.136499000	-0.662250000	-2.089676000
1	-5.796589000	-0.916974000	-3.217930000
1	-7.048938000	-2.160782000	-3.027662000
6	-4.736664000	-3.174949000	-2.025499000
1	-5.371712000	-3.916051000	-2.522019000
1	-4.064761000	-2.756940000	-2.782234000
1	-4.131060000	-3.703869000	-1.281456000
6	-6.572325000	-2.784236000	-0.375158000
1	-7.271995000	-2.077373000	0.080907000
1	-7.162743000	-3.553660000	-0.885755000
1	-6.007413000	-3.264660000	0.430977000
6	-5.481078000	2.165312000	1.473143000
6	-5.022968000	3.529689000	0.903826000
1	-3.944723000	3.677420000	1.017813000
1	-5.264216000	3.607157000	-0.161937000
1	-5.527199000	4.348670000	1.429383000
6	-7.014214000	2.083807000	1.344850000
1	-7.472507000	2.913988000	1.892392000
1	-7.339269000	2.156526000	0.301243000
1	-7.409719000	1.153560000	1.766618000

6	-5.116008000	2.087539000	2.974983000
1	-4.038633000	2.188132000	3.136541000
1	-5.614632000	2.891307000	3.528908000
1	-5.431920000	1.129648000	3.402031000

E= –1859.7384204 a.u.
H= –1859.7384204 + 0.638079 (thermal correction 6-31+G**) a. u.
G= –1859.7384204 + 0.537904 (thermal correction 6-31+G**) a. u.

4e – transition state (*TS*)

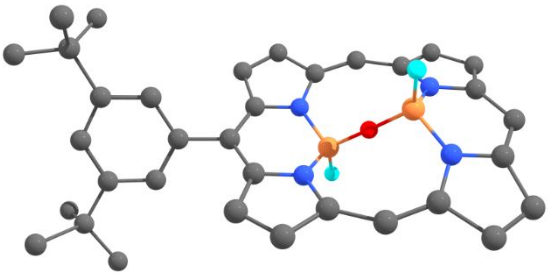

9	3.677923000	0.702496000	2.018304000
5	1.656715000	-0.259453000	-0.752332000
5	3.848118000	0.216537000	0.700045000
8	2.752899000	-0.042744000	-0.020859000
9	1.824484000	-0.701452000	-2.088093000
6	0.202106000	-3.073827000	1.234029000
6	1.358912000	-2.360216000	0.715426000
6	2.703440000	-2.794309000	0.952131000
6	4.058388000	-2.352585000	0.882039000
6	5.188670000	-3.272116000	0.949182000
6	6.347926000	-2.564806000	0.745107000
6	5.971185000	-1.178380000	0.573992000
6	6.683180000	-0.034928000	0.144704000
6	6.008807000	1.136619000	-0.269736000
6	6.433592000	2.296009000	-1.023140000
6	5.301416000	3.007063000	-1.335747000
6	4.140502000	2.298796000	-0.809081000
6	2.803791000	2.730519000	-1.055960000
6	1.443192000	2.295780000	-0.963946000
6	0.314622000	3.200828000	-1.114748000
6	-0.849314000	2.495361000	-0.918236000
6	-0.473414000	1.124850000	-0.650702000
6	-1.206918000	-0.026594000	-0.238330000
6	-0.511155000	-1.202782000	0.170679000
6	-0.932608000	-2.361407000	0.924784000
7	0.839822000	-1.285491000	0.038725000
7	4.621953000	-1.110162000	0.726175000
7	4.658783000	1.225788000	-0.126567000
7	0.883330000	1.064292000	-0.744396000
1	0.257411000	-3.979192000	1.824397000
1	5.096225000	-4.343039000	1.075535000
1	7.353052000	-2.958152000	0.675400000
1	7.450743000	2.519322000	-1.315300000
1	5.246330000	3.911799000	-1.927177000
1	-1.948562000	-2.577392000	1.222364000
1	2.683497000	-3.822922000	1.311246000
1	7.757233000	-0.099508000	0.013609000

1	2.825287000	3.741618000	-1.460917000
1	0.402546000	4.262458000	-1.306029000
1	-1.857699000	2.881627000	-0.936154000
6	-2.682607000	0.011961000	-0.121672000
6	-3.452395000	-1.009323000	-0.709874000
6	-3.326320000	1.036378000	0.579488000
1	-2.931291000	-1.785867000	-1.255488000
1	-2.719259000	1.791969000	1.065716000
6	-4.844664000	-1.008144000	-0.614589000
6	-4.724757000	1.074520000	0.694942000
6	-5.455691000	0.047062000	0.090174000
1	-6.535481000	0.054625000	0.166271000
6	-5.719666000	-2.105879000	-1.246522000
6	-6.702559000	-1.466276000	-2.256297000
1	-7.367134000	-0.742381000	-1.775198000
1	-6.158704000	-0.946985000	-3.052525000
1	-7.328733000	-2.239252000	-2.716536000
6	-4.885283000	-3.163710000	-1.993571000
1	-5.551272000	-3.919553000	-2.422835000
1	-4.311752000	-2.722462000	-2.815514000
1	-4.187855000	-3.679354000	-1.324813000
6	-6.521204000	-2.821766000	-0.132530000
1	-7.174695000	-2.129738000	0.407181000
1	-7.149917000	-3.609468000	-0.563316000
1	-5.845981000	-3.283696000	0.596030000
6	-5.385565000	2.220335000	1.482513000
6	-4.987361000	3.574451000	0.847600000
1	-3.904385000	3.730525000	0.867470000
1	-5.317578000	3.626993000	-0.195629000
1	-5.451509000	4.402017000	1.396127000
6	-6.923117000	2.126053000	1.483339000
1	-7.341276000	2.966168000	2.047551000
1	-7.333863000	2.171565000	0.468907000
1	-7.273623000	1.203611000	1.958604000
6	-4.898243000	2.178800000	2.950825000
1	-3.812263000	2.290723000	3.022795000
1	-5.356948000	2.991279000	3.526142000
1	-5.170101000	1.228781000	3.423281000

E= –1859.6963096 a.u.
H= –1859.6963096 + 0.636408 (thermal correction 6-31+G**) a. u.
G= –1859.6963096 + 0.536517 (thermal correction 6-31+G**) a. u.
f = 184.2407 i

4e– akamptisomer *amplo, amplo* (c_1)

9	5.229128000	-0.688973000	-1.501734000
5	1.585153000	-0.508365000	-1.178677000

5	4.114797000	-0.339262000	-0.702571000
8	2.939589000	-0.603016000	-1.354718000
9	0.890823000	-0.959363000	-2.328776000
6	0.215290000	-3.193717000	1.112656000
6	1.386982000	-2.517520000	0.599503000
6	2.675021000	-2.974641000	0.923294000
6	3.915957000	-2.343996000	1.080822000
6	4.911637000	-2.876732000	1.989472000
6	5.873121000	-1.919520000	2.171341000
6	5.506035000	-0.803766000	1.347053000
6	6.123997000	0.439360000	1.187760000
6	5.531443000	1.429330000	0.399971000
6	5.929237000	2.788842000	0.169966000
6	4.986247000	3.341102000	-0.654551000
6	3.970894000	2.338433000	-0.907162000
6	2.742365000	2.684707000	-1.484880000
6	1.444419000	2.159907000	-1.382892000
6	0.285218000	3.001561000	-1.578273000
6	-0.817429000	2.310770000	-1.150268000
6	-0.356185000	1.024715000	-0.711771000
6	-1.080954000	-0.059860000	-0.181426000
6	-0.385254000	-1.217032000	0.219093000
6	-0.867514000	-2.381065000	0.905126000
7	0.975557000	-1.339429000	0.029682000
7	4.335990000	-1.100604000	0.679604000
7	4.360339000	1.179155000	-0.284953000
7	1.011564000	0.951968000	-0.894065000
1	0.239819000	-4.135634000	1.644680000
1	4.832925000	-3.835617000	2.484702000
1	6.735846000	-1.957586000	2.822582000
1	6.798869000	3.266303000	0.600815000
1	4.932176000	4.363760000	-1.004109000
1	-1.889467000	-2.537917000	1.217935000
1	2.661699000	-3.974066000	1.350480000
1	7.046210000	0.652758000	1.715446000
1	2.753812000	3.679259000	-1.923280000
1	0.325014000	4.021958000	-1.936326000
1	-1.843532000	2.646619000	-1.125020000
6	-2.552591000	0.019616000	-0.010102000
6	-3.376779000	-0.948217000	-0.611779000
6	-3.131729000	1.045304000	0.741168000
1	-2.902334000	-1.721471000	-1.202940000
1	-2.478739000	1.770107000	1.216116000
6	-4.764874000	-0.895863000	-0.473256000
6	-4.523968000	1.133101000	0.903081000
6	-5.311869000	0.154525000	0.289162000
1	-6.387836000	0.200748000	0.398846000

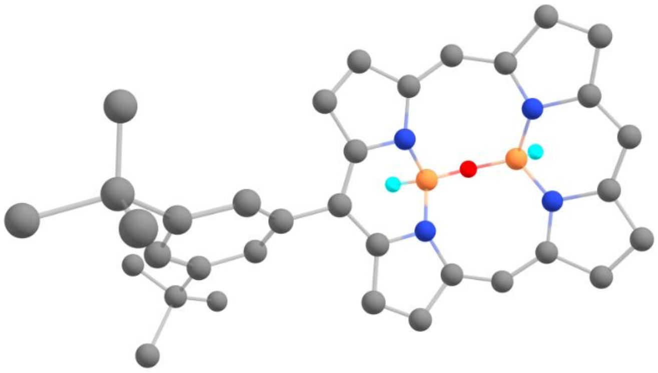

6	-5.702058000	-1.929390000	-1.123856000
6	-6.678779000	-1.206815000	-2.082554000
1	-7.297082000	-0.472302000	-1.557623000
1	-6.130715000	-0.682483000	-2.872567000
1	-7.351465000	-1.931655000	-2.555452000
6	-4.933407000	-2.990648000	-1.933527000
1	-5.642043000	-3.700073000	-2.373345000
1	-4.362204000	-2.541956000	-2.752924000
1	-4.241901000	-3.561585000	-1.304688000
6	-6.508544000	-2.654397000	-0.019402000
1	-7.112316000	-1.956736000	0.568893000
1	-7.187019000	-3.390109000	-0.466140000
1	-5.838268000	-3.181360000	0.668504000
6	-5.117136000	2.282296000	1.738097000
6	-4.698903000	3.635566000	1.114043000
1	-3.611329000	3.755485000	1.093511000
1	-5.067646000	3.720271000	0.085948000
1	-5.113854000	4.466682000	1.695776000
6	-6.655832000	2.236594000	1.794997000
1	-7.026103000	3.076357000	2.392268000
1	-7.102079000	2.317765000	0.797973000
1	-7.017759000	1.314584000	2.262536000
6	-4.578044000	2.196746000	3.186017000
1	-3.487084000	2.273373000	3.219149000
1	-4.989212000	3.011787000	3.792754000
1	-4.862634000	1.246606000	3.650662000

E= –1859.7283748 a.u.
H= –1859.7283748 + 0.637659 (thermal correction 6-31+G**) a. u.
G= –1859.7283748 + 0.536853 (thermal correction 6-31+G**) a. u.

STRUCTURAL VARIATION 5

5a – akamptisomer *amplo, parvo* (***t*$_1$**)

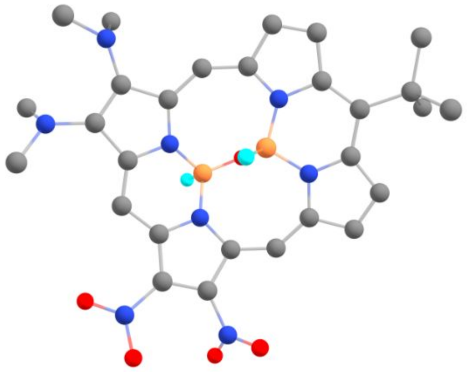

9	0.704950000	0.045413000	-1.094205000
5	-0.650432000	0.081624000	1.246125000
5	1.451787000	-0.115860000	0.105687000
8	0.712536000	-0.080119000	1.305436000
9	-1.278100000	0.108427000	2.502526000
6	-1.418327000	3.594685000	0.049113000
6	-0.412443000	2.624487000	0.382180000
6	0.961423000	2.966409000	0.460059000
6	2.195383000	2.315607000	0.359683000
6	3.448336000	3.033037000	0.363503000
6	4.453624000	2.131401000	0.161091000
6	3.851745000	0.816906000	0.024922000
6	4.434331000	-0.481056000	-0.174443000

6	3.568302000	-1.600422000	-0.124358000
6	3.803125000	-3.038504000	-0.150014000
6	2.600289000	-3.663356000	-0.014006000
6	1.571275000	-2.653349000	0.103275000
6	0.209606000	-2.996093000	0.213592000
6	-1.040590000	-2.351693000	0.261184000
6	-2.277034000	-3.092123000	-0.015100000
6	-3.301656000	-2.158858000	-0.157441000
6	-2.698419000	-0.853352000	0.084749000
6	-3.229900000	0.431120000	0.031697000
6	-2.394403000	1.559665000	0.179064000
6	-2.632396000	2.945023000	-0.090398000
7	-1.075933000	1.425660000	0.503610000
7	2.504928000	0.991203000	0.154082000
7	2.203344000	-1.444619000	0.027280000
7	-1.366378000	-1.030372000	0.386518000
1	3.544525000	4.101863000	0.499327000
1	5.503350000	2.365360000	0.119086000
1	4.749093000	-3.536953000	-0.238814000
1	2.419470000	-4.729536000	0.014423000
1	1.101964000	4.042014000	0.493232000
1	0.079173000	-4.070459000	0.125396000
1	-4.266171000	0.605891000	-0.201671000
6	5.963999000	-0.559422000	-0.422023000
6	6.326102000	0.299577000	-1.667005000
6	6.729338000	-0.082385000	0.842483000
6	6.517983000	-1.965965000	-0.736171000
1	5.852406000	-0.119577000	-2.561072000
1	6.014514000	1.339830000	-1.592770000
1	7.410625000	0.284190000	-1.819450000
1	6.507251000	-0.743781000	1.686507000
1	7.808322000	-0.120241000	0.656178000
1	6.478086000	0.931666000	1.149871000
1	7.577821000	-1.865772000	-0.989373000
1	6.461770000	-2.636336000	0.124632000
1	6.019776000	-2.426590000	-1.593528000
7	-2.302148000	-4.469110000	-0.243110000
7	-4.629412000	-2.447919000	-0.452289000
7	-1.171400000	5.020455000	-0.086701000
8	-0.329957000	5.368478000	-0.917040000
8	-1.786471000	5.765153000	0.669296000
7	-3.861203000	3.517807000	-0.564426000
8	-4.893917000	2.847986000	-0.428716000
8	-3.809118000	4.632544000	-1.085619000
6	-2.174896000	-5.368242000	0.901821000
1	-1.721008000	-6.313699000	0.582471000
1	-1.538756000	-4.915466000	1.665082000

1	-3.148459000	-5.601518000	1.365947000
6	-3.105421000	-4.986877000	-1.343351000
1	-3.087731000	-4.278587000	-2.173228000
1	-2.667474000	-5.933142000	-1.681850000
1	-4.155515000	-5.175625000	-1.072852000
6	-5.357336000	-3.291768000	0.495165000
1	-6.150293000	-3.837801000	-0.027316000
1	-4.683874000	-4.012037000	0.956872000
1	-5.816181000	-2.688678000	1.295928000
6	-5.472939000	-1.474636000	-1.130837000
1	-4.892648000	-0.926308000	-1.875466000
1	-6.274737000	-2.009508000	-1.651550000
1	-5.945505000	-0.755518000	-0.442195000

E= –2148.4714 a.u.
H= –2148.4714 + *0.599464* (thermal correction 6-31+G**) a. u.
G= –2148.4714 + *0.491164* (thermal correction 6-31+G**) a. u.

5a – akamptisomer *parvo, amplo* (***t*₂**)

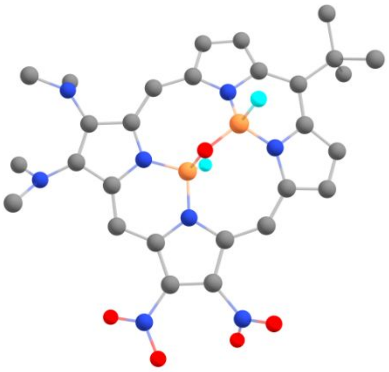

9	-2.403021000	-0.151798000	2.290693000
5	0.373370000	0.104880000	-0.042665000
5	-1.757665000	-0.095596000	1.042541000
8	-0.389733000	0.049588000	1.133440000
9	-0.343366000	0.041000000	-1.266703000
6	1.649753000	3.614267000	-0.020939000
6	0.574244000	2.668817000	0.021559000
6	-0.798195000	3.014268000	0.012284000
6	-2.039075000	2.361984000	-0.006937000
6	-3.219422000	3.092399000	-0.423404000
6	-4.261579000	2.215304000	-0.497550000
6	-3.761523000	0.917532000	-0.096505000
6	-4.396082000	-0.369961000	-0.086215000
6	-3.547014000	-1.499442000	-0.064298000
6	-3.772029000	-2.883480000	-0.441020000
6	-2.572090000	-3.522440000	-0.382549000
6	-1.557018000	-2.561942000	0.013475000
6	-0.202195000	-2.960612000	0.027077000
6	1.065283000	-2.359782000	-0.004970000
6	2.311373000	-3.132022000	-0.032082000
6	3.363122000	-2.220663000	-0.045319000
6	2.754969000	-0.888975000	-0.055171000
6	3.337881000	0.366682000	-0.126579000
6	2.525630000	1.523719000	-0.118183000
6	2.847327000	2.917344000	-0.104882000
7	1.167862000	1.432941000	-0.050965000
7	-2.438334000	1.058526000	0.211767000
7	-2.200045000	-1.372304000	0.228326000

7	1.395266000	-1.039475000	-0.033878000
1	-3.225170000	4.140389000	-0.691988000
1	-5.258139000	2.442708000	-0.837146000
1	-4.698197000	-3.315039000	-0.774610000
1	-2.365098000	-4.549545000	-0.651866000
1	-0.930044000	4.089067000	-0.062047000
1	-0.107062000	-4.041793000	-0.030164000
1	4.408976000	0.479784000	-0.199033000
6	-5.941202000	-0.448895000	-0.138707000
6	-6.467363000	-0.074612000	-1.547648000
6	-6.516792000	-1.845013000	0.201905000
6	-6.525132000	0.501332000	0.944954000
1	-6.194070000	0.936029000	-1.853691000
1	-6.069720000	-0.765791000	-2.298202000
1	-7.560628000	-0.145981000	-1.563683000
1	-6.041020000	-2.282893000	1.083929000
1	-7.584609000	-1.737635000	0.414829000
1	-6.438919000	-2.546179000	-0.630869000
1	-7.618901000	0.454011000	0.916571000
1	-6.194314000	0.186728000	1.940018000
1	-6.234691000	1.542919000	0.818538000
7	2.310130000	-4.531847000	0.060571000
7	4.731077000	-2.409872000	-0.130138000
7	4.173117000	3.468396000	-0.058594000
8	5.101599000	2.747914000	-0.449084000
8	4.303045000	4.610644000	0.381224000
7	1.469892000	5.052415000	-0.092458000
8	0.738985000	5.566877000	0.757415000
8	2.018589000	5.642423000	-1.018139000
6	3.035337000	-5.169921000	1.149945000
1	3.048340000	-4.504325000	2.015984000
1	2.518113000	-6.094304000	1.436670000
1	4.073788000	-5.435702000	0.896368000
6	2.166130000	-5.321880000	-1.154846000
1	3.130185000	-5.535422000	-1.647760000
1	1.692505000	-6.282451000	-0.918285000
1	1.530179000	-4.790057000	-1.866981000
6	5.267041000	-3.545293000	-0.863420000
1	5.418040000	-4.436740000	-0.237377000
1	4.600127000	-3.799504000	-1.688419000
1	6.239669000	-3.263745000	-1.283375000
6	5.603933000	-1.897277000	0.924719000
1	5.125041000	-1.069318000	1.447628000
1	5.832629000	-2.681419000	1.664785000
1	6.547443000	-1.542684000	0.495445000

E= –2148.47046 a.u.

H= –2148.47046 + *0.598931* (thermal correction 6-31+G**) a. u.
G= –2148.47046 + *0.488867* (thermal correction 6-31+G**) a. u.

5a – transition state (*TS*)

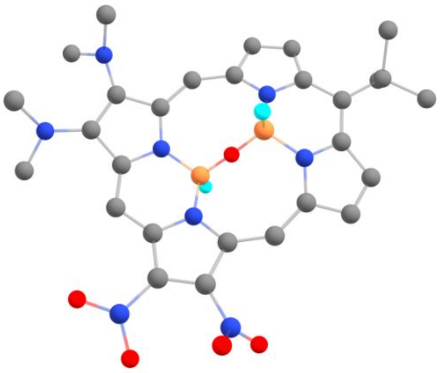

9	-1.433299000	-0.068944000	1.953458000
5	0.566233000	0.109662000	-0.983210000
5	-1.623101000	-0.020045000	0.552397000
8	-0.530505000	0.079595000	-0.220383000
9	0.406141000	0.144561000	-2.385374000
6	1.800460000	3.477208000	0.011864000
6	0.705837000	2.583112000	-0.245638000
6	-0.678051000	2.972996000	-0.184859000
6	-1.986390000	2.438885000	-0.096153000
6	-3.157090000	3.269712000	-0.319579000
6	-4.275226000	2.486101000	-0.246575000
6	-3.844904000	1.126159000	0.054191000
6	-4.520638000	-0.144598000	0.085781000
6	-3.721738000	-1.324826000	-0.028438000
6	-4.034057000	-2.704343000	-0.387909000
6	-2.852779000	-3.380713000	-0.506078000
6	-1.756580000	-2.458599000	-0.241152000
6	-0.398155000	-2.864080000	-0.307353000
6	0.933483000	-2.342708000	-0.301879000
6	2.097117000	-3.193544000	-0.066559000
6	3.247078000	-2.400600000	-0.167541000
6	2.779957000	-1.041753000	-0.465031000
6	3.417703000	0.203321000	-0.441280000
6	2.661956000	1.410769000	-0.380987000
6	2.995657000	2.766415000	-0.054861000
7	1.316671000	1.382496000	-0.525902000
7	-2.489801000	1.181156000	0.169970000
7	-2.363387000	-1.268579000	0.077680000
7	1.417276000	-1.095727000	-0.572560000
1	-5.013594000	-3.111787000	-0.578280000
1	-2.724070000	-4.414314000	-0.799288000
1	-0.740848000	4.057227000	-0.233704000
1	-0.338565000	-3.949613000	-0.379160000
1	4.488283000	0.283802000	-0.346642000
6	-6.058657000	-0.288305000	0.178439000
6	-6.405176000	-1.303218000	1.303066000
6	-6.792641000	1.016798000	0.565838000
6	-6.645780000	-0.750670000	-1.179559000
1	-5.975775000	-2.291882000	1.145255000
1	-6.041765000	-0.936729000	2.268894000
1	-7.492422000	-1.417706000	1.369922000
1	-6.862394000	1.717414000	-0.268189000
1	-7.819720000	0.767956000	0.849555000

1	-6.322642000	1.519026000	1.416284000
1	-7.736030000	-0.830880000	-1.104316000
1	-6.410942000	-0.023019000	-1.963301000
1	-6.259219000	-1.718462000	-1.501952000
7	1.983098000	-4.571813000	0.156891000
6	2.960097000	-5.451201000	-0.473563000
1	2.509687000	-6.442492000	-0.598338000
1	3.219741000	-5.060296000	-1.459178000
1	3.888432000	-5.570908000	0.105529000
6	1.483630000	-5.037364000	1.450378000
1	0.743603000	-4.335785000	1.839156000
1	1.005880000	-6.016094000	1.328538000
1	2.284220000	-5.144409000	2.202347000
7	4.552833000	-2.805233000	0.057211000
6	5.658602000	-2.082762000	-0.557101000
1	6.498678000	-2.772253000	-0.695415000
1	5.361739000	-1.704679000	-1.537218000
1	6.014547000	-1.243089000	0.060942000
6	4.886114000	-3.383261000	1.358688000
1	4.032690000	-3.921505000	1.765663000
1	5.723855000	-4.080776000	1.252616000
1	5.173185000	-2.598830000	2.078140000
7	4.318973000	3.286424000	0.132115000
8	4.466370000	4.508174000	0.088949000
8	5.232864000	2.467538000	0.305203000
7	1.635295000	4.858603000	0.441349000
8	2.053307000	5.159520000	1.552864000
8	1.044022000	5.610632000	-0.336045000
1	-3.121696000	4.323702000	-0.562177000
1	-5.280264000	2.819172000	-0.433247000

E= –2148.4329654 a.u.
H= –2148.4329654 + 0.597764 (thermal correction 6-31+G**) a. u.
G= –2148.4329654 + 0.490194 (thermal correction 6-31+G**) a. u.
f = 175.2249 i

5a – akamptisomer *amplo, amplo* (c_1)

9	2.740299000	-0.406676000	2.228090000
5	-0.748442000	0.082975000	1.212490000
5	1.832191000	-0.227721000	1.155755000
8	0.547424000	-0.082878000	1.617480000
9	-1.629293000	0.140974000	2.317063000
6	-1.443182000	3.618681000	0.102931000
6	-0.448866000	2.652492000	0.460514000
6	0.904104000	3.000403000	0.628398000
6	2.109175000	2.324250000	0.436327000

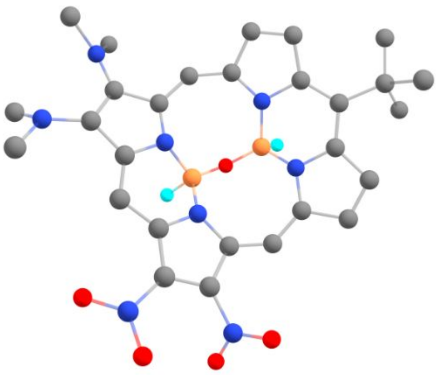

6	3.287991000	3.063609000	0.057874000
6	4.233236000	2.164648000	-0.343940000
6	3.679428000	0.842205000	-0.175301000
6	4.246553000	-0.444468000	-0.441164000
6	3.383523000	-1.558299000	-0.346294000
6	3.551787000	-2.945878000	-0.725525000
6	2.401629000	-3.604542000	-0.412386000
6	1.467307000	-2.645408000	0.128077000
6	0.139388000	-3.036776000	0.376088000
6	-1.092640000	-2.380708000	0.346846000
6	-2.338431000	-3.096709000	0.097769000
6	-3.286000000	-2.142381000	-0.298706000
6	-2.636545000	-0.850954000	-0.172046000
6	-3.148593000	0.431181000	-0.323252000
6	-2.337373000	1.551499000	-0.076569000
6	-2.599437000	2.946712000	-0.250613000
7	-1.050669000	1.419208000	0.380582000
7	2.397893000	0.983425000	0.311689000
7	2.092704000	-1.430978000	0.167927000
7	-1.327729000	-1.031642000	0.246669000
1	1.053416000	4.072656000	0.692306000
1	0.040292000	-4.115121000	0.360289000
6	5.754688000	-0.525274000	-0.799640000
6	6.338299000	-1.952451000	-0.910075000
1	6.127204000	-2.558087000	-0.024781000
1	5.992093000	-2.476307000	-1.803847000
1	7.425601000	-1.868997000	-0.999776000
6	6.024124000	0.146807000	-2.172645000
1	7.093976000	0.096626000	-2.402851000
1	5.482359000	-0.383463000	-2.963133000
1	5.722918000	1.192600000	-2.216848000
6	6.573996000	0.146762000	0.339895000
1	6.421370000	-0.395648000	1.278544000
1	7.640321000	0.109606000	0.092304000
1	6.309206000	1.186418000	0.521772000
1	2.172188000	-4.644963000	-0.600475000
1	4.402675000	-3.383723000	-1.211623000
1	-4.161645000	0.608551000	-0.637910000
7	-2.525847000	-4.470002000	0.109243000
6	-2.948554000	-5.152874000	-1.105747000
1	-3.594294000	-6.002864000	-0.853552000
1	-2.086321000	-5.533778000	-1.676538000
6	-1.999079000	-5.316343000	1.168099000
1	-1.718752000	-4.705508000	2.028224000
1	-2.771168000	-6.029447000	1.488184000
1	-1.122321000	-5.903754000	0.851618000
1	-3.513626000	-4.468718000	-1.741968000

7	-4.576927000	-2.414298000	-0.721868000
6	-5.410494000	-3.259367000	0.136092000
1	-6.185548000	-3.740115000	-0.469091000
1	-5.897360000	-2.662171000	0.922413000
6	-5.329396000	-1.460795000	-1.523985000
1	-4.668538000	-0.959756000	-2.233942000
1	-6.088596000	-2.008190000	-2.092180000
1	-5.848056000	-0.702106000	-0.917331000
1	-4.803463000	-4.030648000	0.610119000
1	5.201554000	2.411555000	-0.743502000
1	3.347970000	4.142974000	0.016784000
7	-1.228382000	5.058061000	0.103090000
8	-1.889409000	5.717721000	0.895935000
8	-0.367338000	5.494641000	-0.662441000
7	-3.786219000	3.532161000	-0.808180000
8	-4.788895000	2.813492000	-0.914848000
8	-3.728637000	4.713240000	-1.155001000

E= –2148.460972 a.u.
H= –2148.460972 + 0.599112 (thermal correction 6-31+G**) a. u.
G= –2148.460972 + 0.489178 (thermal correction 6-31+G**) a. u.

5b – akamptisomer *amplo, parvo* (***t***$_1$)

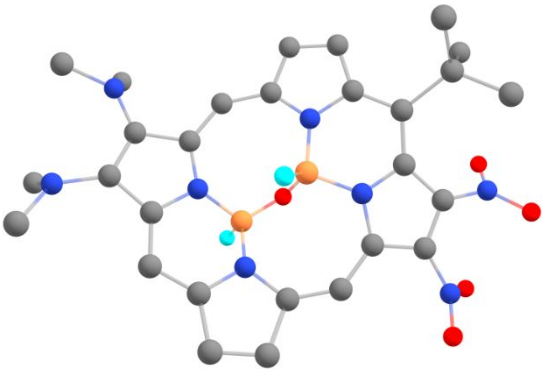

9	-0.228553000	0.122808000	-1.305179000
5	-1.347160000	1.139621000	0.963476000
5	0.392923000	-0.151255000	-0.062377000
8	-0.191811000	0.399635000	1.091269000
9	-1.851397000	1.621118000	2.185560000
6	-0.504744000	4.394621000	-0.785243000
6	-0.035007000	3.129696000	-0.234244000
6	1.332192000	2.849004000	-0.133349000
6	2.160091000	1.700072000	-0.027361000
6	3.581790000	1.746648000	0.137856000
6	4.068722000	0.452153000	0.131108000
6	2.967766000	-0.438493000	-0.099401000
6	2.860633000	-1.862911000	-0.275550000
6	1.595927000	-2.432699000	-0.007133000
6	1.195389000	-3.806710000	0.229716000
6	-0.143165000	-3.807060000	0.497187000
6	-0.622791000	-2.443216000	0.418477000
6	-1.995861000	-2.124917000	0.531536000
6	-2.821236000	-0.998954000	0.402665000
6	-4.268515000	-1.140258000	0.176973000
6	-4.754128000	0.105373000	-0.207810000
6	-3.624020000	1.022170000	-0.146285000
6	-3.512062000	2.384943000	-0.450381000
6	-2.260991000	3.026691000	-0.430324000

6	-1.862635000	4.323263000	-0.923790000
7	-1.153261000	2.368337000	0.009758000
7	1.852738000	0.365790000	-0.130718000
7	0.462213000	-1.665432000	0.134788000
7	-2.515420000	0.329337000	0.271355000
1	1.847668000	-4.664093000	0.246591000
1	-0.761718000	-4.660743000	0.739709000
1	1.930445000	3.742739000	-0.273201000
1	-2.590226000	-3.029244000	0.622781000
1	-4.369676000	2.942129000	-0.796499000
6	3.982638000	-2.763334000	-0.866591000
6	5.134917000	-1.976532000	-1.542875000
6	4.598558000	-3.691142000	0.208391000
6	3.365609000	-3.607486000	-2.022895000
1	4.775015000	-1.108979000	-2.103522000
1	5.906006000	-1.662341000	-0.845199000
1	5.632013000	-2.638745000	-2.256871000
1	3.863522000	-4.369616000	0.646315000
1	5.387670000	-4.299517000	-0.246983000
1	5.040318000	-3.102073000	1.014767000
1	4.148134000	-4.236763000	-2.458750000
1	2.552930000	-4.260926000	-1.711642000
1	2.980068000	-2.951142000	-2.810540000
7	-4.925957000	-2.368207000	0.216749000
7	-6.082448000	0.389528000	-0.535364000
6	-5.158538000	-2.982663000	1.523620000
1	-5.214583000	-4.071922000	1.416823000
1	-4.341152000	-2.738528000	2.204761000
1	-6.099724000	-2.637566000	1.984124000
6	-5.950599000	-2.654987000	-0.779621000
1	-5.666652000	-2.210968000	-1.734789000
1	-6.020247000	-3.741697000	-0.904446000
1	-6.947448000	-2.277055000	-0.506485000
6	-7.064279000	0.268667000	0.541657000
1	-8.057833000	0.090249000	0.115835000
1	-6.809724000	-0.568999000	1.190608000
1	-7.106444000	1.181626000	1.159592000
6	-6.392654000	1.440282000	-1.491704000
1	-5.635060000	1.466898000	-2.277900000
1	-7.360328000	1.221411000	-1.957374000
1	-6.467876000	2.440211000	-1.030686000
1	-2.526592000	5.062278000	-1.350988000
1	0.148147000	5.202324000	-1.088629000
7	5.425533000	0.113290000	0.496922000
8	5.565040000	-0.759560000	1.355834000
8	6.336373000	0.717381000	-0.063709000
7	4.355601000	2.935114000	0.440219000

8	5.142529000	2.873723000	1.380395000
8	4.158252000	3.930852000	-0.262547000

E= –2148.4584874 a.u.
H= –2148.4584874 + *0.598848* (thermal correction 6-31+G**) a. u.
G= –2148.4584874 + *0.491845* (thermal correction 6-31+G**) a. u.

5b – akamptisomer *parvo, amplo* (t_2)

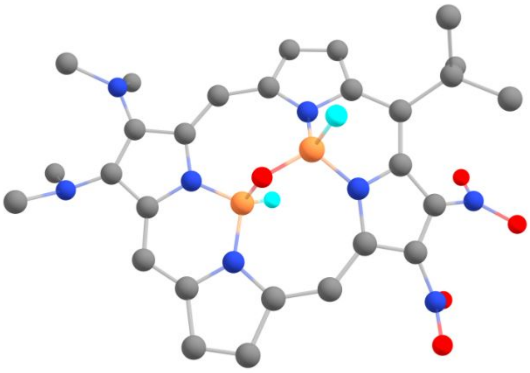

9	-1.091088000	-0.850891000	2.445953000
5	1.107192000	0.870255000	0.106168000
5	-0.613647000	-0.387299000	1.208983000
8	0.527173000	0.371893000	1.290748000
9	0.418373000	0.565338000	-1.099151000
6	0.633304000	4.566525000	0.525315000
6	0.117175000	3.209425000	0.441257000
6	-1.238027000	2.892858000	0.496558000
6	-2.056219000	1.731996000	0.356022000
6	-3.403295000	1.836901000	-0.125044000
6	-3.911902000	0.563962000	-0.296621000
6	-2.920013000	-0.365488000	0.144554000
6	-2.877913000	-1.805252000	0.179627000
6	-1.597727000	-2.381966000	0.046166000
6	-1.193206000	-3.700226000	-0.387109000
6	0.169508000	-3.700594000	-0.452319000
6	0.649256000	-2.390997000	-0.053562000
6	2.038118000	-2.110130000	-0.105045000
6	2.867632000	-0.987369000	-0.119819000
6	4.330608000	-1.069171000	-0.243745000
6	4.822134000	0.228302000	-0.192605000
6	3.665448000	1.105180000	-0.053628000
6	3.590686000	2.496464000	0.019323000
6	2.343844000	3.127547000	0.178390000
6	1.990793000	4.517026000	0.363234000
7	1.199741000	2.394850000	0.222532000
7	-1.825799000	0.383217000	0.511075000
7	-0.452220000	-1.640813000	0.258902000
7	2.540929000	0.335016000	-0.026082000
1	-1.850520000	-4.503071000	-0.678703000
1	0.807755000	-4.502615000	-0.798455000
1	-1.848696000	3.785178000	0.581044000
1	2.622016000	-3.017554000	-0.235986000
1	4.497974000	3.084739000	-0.031406000
6	-4.101553000	-2.711201000	0.472677000
6	-4.573834000	-3.508702000	-0.766180000
6	-3.680630000	-3.693383000	1.607345000
6	-5.310423000	-1.929699000	1.044452000
1	-4.882628000	-2.836653000	-1.568028000

1	-3.798296000	-4.170632000	-1.156264000
1	-5.430950000	-4.130577000	-0.485201000
1	-3.341916000	-3.140105000	2.488986000
1	-4.545486000	-4.301856000	1.890943000
1	-2.879216000	-4.371049000	1.313036000
1	-5.975965000	-2.638839000	1.544900000
1	-5.004358000	-1.187775000	1.787951000
1	-5.913529000	-1.445112000	0.280763000
7	4.995425000	-2.301997000	-0.300046000
7	6.114784000	0.738910000	-0.326412000
6	6.072786000	-2.560605000	0.645789000
1	5.864645000	-2.040107000	1.583253000
1	6.116986000	-3.636723000	0.853484000
1	7.065686000	-2.250018000	0.285675000
6	5.125634000	-2.958273000	-1.596672000
1	5.995233000	-2.597429000	-2.171564000
1	5.239803000	-4.038923000	-1.452849000
1	4.226123000	-2.781836000	-2.191484000
6	7.022025000	0.117596000	-1.281335000
1	7.585612000	-0.732715000	-0.869383000
1	6.457117000	-0.224014000	-2.151010000
1	7.748412000	0.867091000	-1.616210000
6	6.760547000	1.271877000	0.871841000
1	6.024594000	1.777689000	1.500077000
1	7.236921000	0.478341000	1.472127000
1	7.531157000	1.995473000	0.584671000
1	2.690591000	5.341256000	0.374308000
1	0.019072000	5.442428000	0.686454000
7	-5.147693000	0.287228000	-0.992348000
8	-5.101862000	-0.546019000	-1.898881000
8	-6.150185000	0.902789000	-0.636665000
7	-4.041525000	3.056724000	-0.585439000
8	-4.558568000	3.041444000	-1.699246000
8	-4.012239000	4.030273000	0.173222000

E= –2148.4620453 a.u.
H= –2148.4620453 + *0.598598* (thermal correction 6-31+G**) a. u.
G= –2148.4620453 + *0.489578* (thermal correction 6-31+G**) a. u.

5b – transition state (*TS*)

9	-0.269772000	-0.507447000	2.067896000
5	1.288027000	1.046934000	-0.735964000
5	-0.502094000	-0.346198000	0.684945000
8	0.352586000	0.393799000	-0.029347000
9	1.091345000	1.164495000	-2.130016000
6	0.785725000	4.426778000	0.795664000
6	0.250527000	3.138124000	0.362262000

6	-1.119117000	2.808584000	0.463502000
6	-2.033628000	1.697280000	0.345772000
6	-3.434006000	1.859090000	0.069112000
6	-4.018769000	0.606639000	-0.054823000
6	-3.015303000	-0.383783000	0.214375000
6	-2.973554000	-1.834559000	0.173632000
6	-1.715082000	-2.429008000	-0.111444000
6	-1.331916000	-3.728426000	-0.640298000
6	0.020085000	-3.704297000	-0.850124000
6	0.532736000	-2.401038000	-0.456380000
6	1.924691000	-2.084525000	-0.512598000
6	2.824936000	-0.995021000	-0.392528000
6	4.272200000	-1.185432000	-0.244703000
6	4.875322000	0.072719000	-0.188567000
6	3.791092000	1.052486000	-0.290369000
6	3.738280000	2.442455000	-0.071568000
6	2.497801000	3.107578000	0.114310000
6	2.150317000	4.404435000	0.660154000
7	1.344173000	2.436313000	-0.091721000
7	-1.875083000	0.332198000	0.449492000
7	-0.557508000	-1.707509000	-0.008551000
7	2.628545000	0.358437000	-0.452826000
1	-1.999725000	-4.536687000	-0.892567000
1	0.622763000	-4.492748000	-1.281317000
1	-1.687521000	3.702002000	0.704510000
1	2.498409000	-2.990338000	-0.703058000
1	4.650287000	3.003957000	0.072050000
6	-4.178922000	-2.749406000	0.506298000
6	-3.692309000	-3.829693000	1.517593000
6	-5.328842000	-2.008574000	1.232890000
6	-4.752910000	-3.433061000	-0.758526000
1	-2.905390000	-4.470604000	1.121610000
1	-3.311987000	-3.358514000	2.430021000
1	-4.537604000	-4.469695000	1.790596000
1	-6.000494000	-1.488578000	0.555001000
1	-5.943709000	-2.747145000	1.755229000
1	-4.957043000	-1.304606000	1.983524000
1	-5.603302000	-4.062637000	-0.474239000
1	-5.099738000	-2.688835000	-1.477517000
1	-4.016705000	-4.069345000	-1.254232000
1	2.851196000	5.179055000	0.939979000
1	0.184112000	5.228615000	1.203200000
7	4.863236000	-2.452729000	-0.213922000
6	6.148353000	-2.627230000	-0.880311000
1	6.254709000	-3.681435000	-1.159940000
1	6.175381000	-2.020757000	-1.787181000
1	7.010754000	-2.351377000	-0.254554000

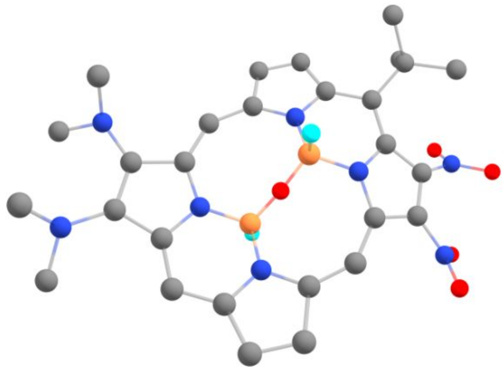

6	4.683342000	-3.283293000	0.977918000
1	3.705666000	-3.094499000	1.423698000
1	4.742845000	-4.341941000	0.701234000
1	5.454009000	-3.090311000	1.743895000
7	6.227499000	0.325981000	0.026388000
6	6.828608000	1.541223000	-0.502042000
1	7.889704000	1.357034000	-0.705921000
1	6.343830000	1.822117000	-1.439207000
1	6.770403000	2.389859000	0.200374000
6	6.807698000	-0.111047000	1.294715000
1	6.340522000	-1.038218000	1.623129000
1	7.882336000	-0.285307000	1.171380000
1	6.665531000	0.644646000	2.086105000
7	-4.093817000	3.112823000	-0.245118000
8	-3.860580000	4.074238000	0.493794000
8	-4.833023000	3.136776000	-1.225528000
7	-5.359196000	0.403702000	-0.559799000
8	-5.478435000	-0.345495000	-1.530414000
8	-6.277062000	0.985765000	0.012403000

E= – 2148.4225127 a.u.
H=– 2148.4225127 + 0.597169 (thermal correction 6-31+G**) a. u.
G=– 2148.4225127 + 0.490209 (thermal correction 6-31+G**) a. u.
f = 173.5774 i

5b– akamptisomer *amplo, amplo* (c_1)

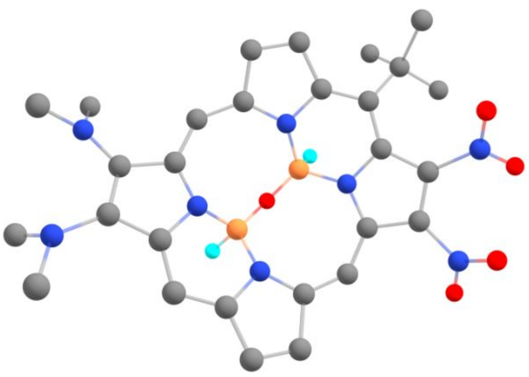

9	1.339249000	-1.076197000	2.351246000
5	-1.477877000	0.982515000	1.198749000
5	0.681041000	-0.476014000	1.253437000
8	-0.412182000	0.242206000	1.646216000
9	-2.297086000	1.386097000	2.282757000
6	-0.451798000	4.487170000	0.257861000
6	-0.017820000	3.136232000	0.573815000
6	1.330837000	2.839710000	0.717128000
6	2.122806000	1.685369000	0.498193000
6	3.450566000	1.806881000	-0.023048000
6	3.891399000	0.550232000	-0.387320000
6	2.886075000	-0.390567000	-0.013417000
6	2.827314000	-1.820992000	-0.090635000
6	1.533238000	-2.370091000	-0.154486000
6	1.096155000	-3.679190000	-0.566422000
6	-0.260744000	-3.716025000	-0.429850000
6	-0.698422000	-2.430707000	0.063534000
6	-2.076946000	-2.176295000	0.215334000
6	-2.870392000	-1.034080000	0.188728000
6	-4.293666000	-1.087689000	-0.149957000

6	-4.671134000	0.208650000	-0.518563000
6	-3.518375000	1.051524000	-0.282595000
6	-3.371319000	2.437535000	-0.375431000
6	-2.153657000	3.045760000	-0.048888000
6	-1.753345000	4.423945000	-0.153445000
7	-1.101209000	2.305047000	0.422742000
7	1.823852000	0.344380000	0.483937000
7	0.407600000	-1.638926000	0.212232000
7	-2.463586000	0.277177000	0.167619000
1	0.198369000	5.351992000	0.269775000
1	-2.375924000	5.229367000	-0.518506000
1	1.938812000	3.732124000	0.812545000
1	-2.651575000	-3.090324000	0.130990000
6	4.055091000	-2.762371000	0.056407000
6	3.706645000	-3.805124000	1.162352000
1	3.443415000	-3.298522000	2.096129000
1	2.877160000	-4.459077000	0.896312000
1	4.582505000	-4.436054000	1.344132000
6	4.417049000	-3.482394000	-1.264078000
1	5.283390000	-4.130885000	-1.093573000
1	3.603841000	-4.107916000	-1.637876000
1	4.675550000	-2.759971000	-2.040319000
6	5.323608000	-2.044650000	0.582565000
1	5.094658000	-1.338776000	1.386005000
1	5.998779000	-2.799412000	0.995119000
1	5.888313000	-1.537438000	-0.195083000
1	-0.925811000	-4.526521000	-0.696752000
1	1.722647000	-4.458769000	-0.967387000
1	-4.180352000	3.061216000	-0.723271000
7	5.066820000	0.324409000	-1.195339000
8	6.105181000	0.889427000	-0.860123000
8	4.935970000	-0.414307000	-2.173952000
7	4.099144000	3.058789000	-0.366425000
8	4.162761000	3.918450000	0.516392000
8	4.522150000	3.184284000	-1.513178000
7	-5.086951000	-2.218015000	-0.215614000
6	-5.783609000	-2.556006000	-1.449450000
1	-6.760686000	-2.998381000	-1.220166000
1	-5.209213000	-3.281740000	-2.046632000
6	-5.046896000	-3.259321000	0.799220000
1	-4.530925000	-2.895076000	1.689316000
1	-6.071783000	-3.535018000	1.083126000
1	-4.545969000	-4.175919000	0.449782000
1	-5.942953000	-1.656881000	-2.047076000
7	-5.928877000	0.571206000	-0.993633000
6	-7.078444000	0.250224000	-0.146906000
1	-7.982506000	0.201645000	-0.762699000

1	-7.225701000	1.011921000	0.635288000
6	-6.109176000	1.755756000	-1.815990000
1	-5.250129000	1.888932000	-2.476994000
1	-7.000687000	1.619810000	-2.438148000
1	-6.255993000	2.675414000	-1.225208000
1	-6.931346000	-0.717214000	0.334808000

E= –2148.4511578 a.u.
H= –2148.4511578 + 0.598487 (thermal correction 6-31+G**) a. u.
G= –2148.4511578 + 0.489912 (thermal correction 6-31+G**) a. u.

5c– akamptisomer *amplo, parvo* (***t***$_1$)

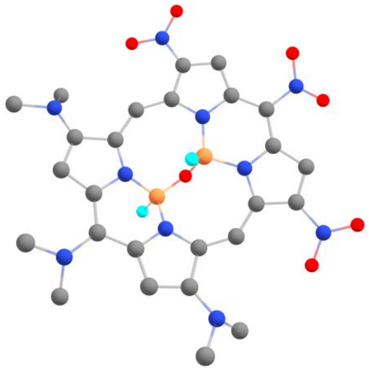

9	0.084808000	-0.263318000	-1.211213000
5	-0.092279000	1.123760000	1.106302000
5	0.067022000	-0.997836000	0.002738000
8	-0.015547000	-0.249367000	1.187736000
9	-0.218738000	1.760360000	2.355436000
6	3.245408000	2.512409000	-0.131227000
6	2.435873000	1.331256000	0.251364000
6	3.006333000	0.049802000	0.229174000
6	2.608144000	-1.297830000	0.159906000
6	3.516320000	-2.430023000	0.072111000
6	2.799854000	-3.591972000	-0.092361000
6	1.424508000	-3.207879000	-0.106600000
6	0.230495000	-3.953220000	-0.259538000
6	-1.040263000	-3.331899000	-0.211795000
6	-2.361971000	-3.873155000	-0.213881000
6	-3.210155000	-2.804672000	-0.048347000
6	-2.440398000	-1.573909000	0.053890000
6	-2.995876000	-0.286375000	0.161330000
6	-2.571602000	1.050768000	0.193152000
6	-3.527991000	2.157621000	-0.017621000
6	-2.776349000	3.301151000	-0.244397000
6	-1.405332000	2.944339000	-0.083103000
6	-0.209596000	3.720456000	-0.171396000
6	1.055859000	3.065815000	-0.039844000
6	2.354260000	3.544451000	-0.377704000
7	1.140699000	1.768582000	0.377072000
7	1.356217000	-1.846657000	0.042206000
7	-1.130627000	-1.972774000	-0.055366000
7	-1.319233000	1.609916000	0.223265000
1	-2.638360000	-4.908173000	-0.313956000
1	4.081021000	0.103488000	0.143328000
1	-4.072056000	-0.357836000	0.117074000
7	-4.895807000	2.036386000	-0.084112000
6	-5.636402000	1.382882000	1.006534000

1	-6.103546000	2.144160000	1.648433000
1	-6.414982000	0.729404000	0.602700000
1	-4.970165000	0.786930000	1.627509000
6	-5.634609000	3.123222000	-0.711780000
1	-5.169182000	3.390819000	-1.663829000
1	-6.656170000	2.787337000	-0.908644000
1	-5.684952000	4.020200000	-0.071856000
1	2.586602000	4.524025000	-0.761593000
7	4.959529000	-2.426540000	0.156184000
8	5.537374000	-1.378571000	0.481112000
8	5.542457000	-3.481490000	-0.097675000
1	-3.168088000	4.280503000	-0.456336000
7	-0.218240000	5.068043000	-0.473429000
6	-1.187430000	5.682561000	-1.367150000
1	-0.677304000	6.457247000	-1.951608000
1	-1.589440000	4.943080000	-2.060246000
1	-2.014902000	6.164113000	-0.825131000
6	0.596387000	6.023101000	0.274961000
1	1.162879000	5.514779000	1.054044000
1	1.291153000	6.561335000	-0.382733000
1	-0.060759000	6.762274000	0.754186000
7	4.607353000	2.517873000	-0.332149000
6	5.153828000	3.616622000	-1.116484000
1	6.188688000	3.381802000	-1.378894000
1	4.581709000	3.737570000	-2.039706000
1	5.147227000	4.570813000	-0.562447000
6	5.505002000	2.075176000	0.746383000
1	6.354934000	1.527095000	0.330411000
1	5.878202000	2.945737000	1.306829000
1	4.984590000	1.424739000	1.446764000
1	3.195384000	-4.589432000	-0.168634000
7	0.306448000	-5.389266000	-0.451735000
8	1.332757000	-5.975728000	-0.087973000
8	-0.659791000	-5.960098000	-0.972062000
7	-4.644839000	-2.974023000	0.022597000
8	-5.348320000	-2.002533000	0.336883000
8	-5.093568000	-4.093460000	-0.227282000

E= –2329.7922436 a.u.
H= –2329.7922436 + 0.563672 (thermal correction 6-31+G**) a. u.
G= –2329.7922436 + 0.448539 (thermal correction 6-31+G**) a. u.

5c– akamptisomer *parvo, amplo* (***t*₂**)

9	-0.240379000	-1.926695000	2.376880000
5	0.091893000	0.820917000	0.017602000
5	-0.158179000	-1.291105000	1.130111000
8	-0.024499000	0.074727000	1.203343000

9	0.095819000	0.082723000	-1.196259000
6	-3.107868000	2.790340000	0.208020000
6	-2.372640000	1.515971000	0.167710000
6	-3.000814000	0.263261000	0.185348000
6	-2.641390000	-1.097245000	0.117985000
6	-3.573008000	-2.149810000	-0.272379000
6	-2.910218000	-3.349319000	-0.360782000
6	-1.559176000	-3.073689000	0.000961000
6	-0.398635000	-3.885473000	-0.045244000
6	0.897118000	-3.321810000	0.024123000
6	2.186736000	-3.864632000	-0.262463000
6	3.067403000	-2.821019000	-0.137917000
6	2.345462000	-1.597791000	0.204518000
6	2.968562000	-0.336238000	0.233528000
6	2.621732000	1.024875000	0.204486000
6	3.623880000	2.093732000	0.083417000
6	2.937199000	3.265818000	-0.207047000
6	1.541657000	2.959089000	-0.183947000
6	0.380731000	3.770765000	-0.363430000
6	-0.913189000	3.181017000	-0.199893000
6	-2.188597000	3.799447000	-0.060631000
7	-1.058280000	1.833522000	-0.045989000
7	-1.438157000	-1.741118000	0.304493000
7	1.032118000	-1.990969000	0.341886000
7	1.400354000	1.623347000	0.085027000
1	2.416796000	-4.874825000	-0.550819000
1	-4.075719000	0.360716000	0.128245000
1	4.038502000	-0.460821000	0.144881000
7	4.987531000	1.921634000	0.168532000
6	5.819802000	3.001765000	-0.342158000
1	5.811455000	3.885358000	0.318856000
1	6.849796000	2.645076000	-0.422616000
1	5.478352000	3.298681000	-1.337090000
6	5.576922000	1.236359000	1.328694000
1	6.413382000	0.608234000	1.009756000
1	5.940219000	1.975512000	2.058906000
1	4.840732000	0.607150000	1.824982000
1	-2.380212000	4.859802000	-0.069737000
7	-4.982068000	-2.039746000	-0.586003000
8	-5.572676000	-0.975722000	-0.348254000
8	-5.532175000	-3.034185000	-1.061142000
1	3.381321000	4.231248000	-0.373795000
7	0.463735000	5.119548000	-0.651558000
6	-0.371490000	5.743502000	-1.673841000
1	0.272410000	6.215540000	-2.428780000
1	-0.990837000	4.998281000	-2.171252000
1	-1.019278000	6.520715000	-1.247033000

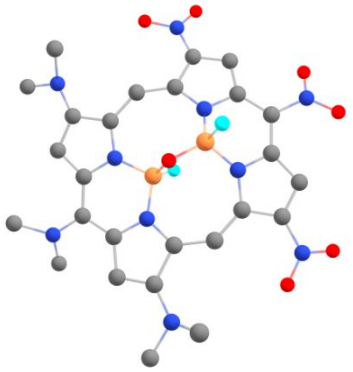

6	1.496203000	5.989956000	-0.110694000
1	1.910961000	5.566003000	0.804178000
1	2.309241000	6.176653000	-0.827857000
1	1.042139000	6.957049000	0.136776000
7	-4.456048000	2.932143000	0.421679000
6	-5.142645000	2.218904000	1.508586000
1	-5.442712000	2.934402000	2.286952000
1	-6.034438000	1.706992000	1.134220000
1	-4.482495000	1.484546000	1.967319000
6	-5.054953000	4.217935000	0.093360000
1	-4.813992000	4.992550000	0.840198000
1	-4.710225000	4.552109000	-0.888600000
1	-6.141236000	4.102607000	0.056387000
1	-3.324498000	-4.296721000	-0.659221000
7	-0.559178000	-5.311944000	-0.272567000
8	0.394506000	-5.950827000	-0.731375000
8	-1.649268000	-5.820848000	0.012722000
7	4.486119000	-3.000046000	-0.372060000
8	5.269307000	-2.096341000	-0.044877000
8	4.844459000	-4.069182000	-0.867284000

E= –2329.7911918 a.u.
H= –2329.7911918 + 0.563813 (thermal correction 6-31+G**) a. u.
G= –2329.7911918+ 0.448559 (thermal correction 6-31+G**) a. u.

5c – transition state (*TS*)

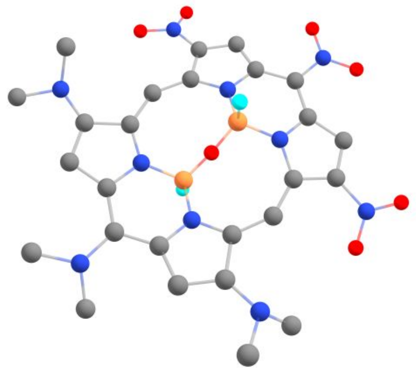

9	1.083715000	0.125252000	-2.138587000
5	-1.018492000	-0.194839000	0.718626000
5	1.193792000	0.180121000	-0.732530000
8	0.072542000	0.055744000	-0.013240000
9	-0.877387000	-0.296298000	2.120269000
6	-2.942545000	2.907714000	-0.196795000
6	-1.656155000	2.226650000	0.071586000
6	-0.402893000	2.877569000	-0.077343000
6	1.008820000	2.668569000	-0.110914000
6	2.009477000	3.721818000	0.033523000
6	3.281299000	3.185003000	-0.009122000
6	3.110910000	1.779534000	-0.209254000
6	3.997944000	0.652750000	-0.208076000
6	3.504375000	-0.683957000	-0.119755000
6	4.118363000	-1.935303000	0.230260000
6	3.099270000	-2.852541000	0.316313000
6	1.810349000	-2.211322000	0.038992000
6	0.559094000	-2.866080000	0.075515000
6	-0.850102000	-2.631410000	-0.006314000
6	-1.813463000	-3.721721000	-0.190025000

6	-3.092968000	-3.183113000	-0.073988000
6	-2.944294000	-1.772608000	0.135269000
6	-3.848765000	-0.654729000	0.225985000
6	-3.320519000	0.698740000	0.171552000
6	-3.939100000	1.932690000	-0.189102000
7	-2.002002000	0.925538000	0.364659000
7	1.775028000	1.539089000	-0.304033000
7	2.168639000	-0.907881000	-0.252631000
7	-1.603812000	-1.507495000	0.170269000
1	5.163539000	-2.115461000	0.411247000
1	-0.572077000	3.937882000	-0.202856000
1	0.727810000	-3.927576000	0.214317000
7	-1.477980000	-5.047268000	-0.406724000
6	-0.619287000	-5.394818000	-1.550232000
1	-1.233525000	-5.619834000	-2.436756000
1	0.048661000	-4.573070000	-1.800284000
1	-0.013674000	-6.271199000	-1.303878000
6	-2.535253000	-6.025095000	-0.186445000
1	-3.019749000	-5.840714000	0.775685000
1	-3.299100000	-6.005477000	-0.983328000
1	-2.092022000	-7.024114000	-0.165010000
1	4.207192000	3.715773000	0.125514000
1	-4.007131000	-3.737314000	-0.189368000
7	-5.211578000	-0.800476000	0.263201000
6	-5.900879000	-1.978981000	-0.243228000
1	-6.918318000	-1.687597000	-0.519155000
1	-5.402545000	-2.356069000	-1.136854000
1	-5.966300000	-2.778230000	0.508454000
6	-6.059598000	0.075772000	1.075314000
1	-5.454228000	0.778898000	1.644388000
1	-6.770315000	0.632296000	0.452788000
1	-6.630425000	-0.539611000	1.782485000
7	-3.098535000	4.243768000	-0.507837000
6	-4.343088000	4.618671000	-1.165746000
1	-4.248316000	5.640838000	-1.541374000
1	-4.533396000	3.954579000	-2.012532000
1	-5.207771000	4.584109000	-0.480414000
6	-2.634404000	5.261462000	0.449494000
1	-1.814283000	4.888898000	1.058587000
1	-2.285819000	6.148261000	-0.086481000
1	-3.457244000	5.549932000	1.122061000
1	-4.979942000	2.079161000	-0.426759000
7	3.359247000	-4.236964000	0.658587000
8	4.499418000	-4.523941000	1.024112000
8	2.440785000	-5.061006000	0.550050000
7	5.423939000	0.878409000	-0.198356000
8	6.177496000	-0.104460000	-0.231952000

8	5.832696000	2.047008000	-0.167216000
7	1.794365000	5.129931000	0.276301000
8	2.778737000	5.869061000	0.234814000
8	0.642681000	5.528907000	0.510313000

E= –2329.7511207 a.u.
H= –2329.7511207 + 0.562346 (thermal correction 6-31+G**) a. u.
G= –2329.7511207 + 0.447971 (thermal correction 6-31+G**) a. u.
f= 170.2087 *i*

5c– akamptisomer *amplo, amplo* (c_1)

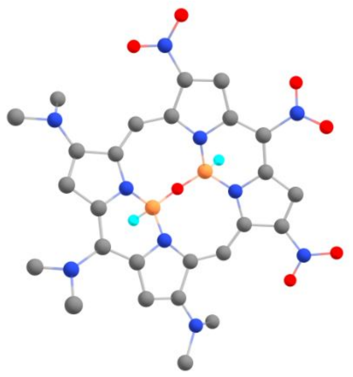

9	-0.032345000	-2.303239000	2.206181000
5	0.088394000	1.197191000	1.110513000
5	-0.036912000	-1.375881000	1.145292000
8	0.022957000	-0.085777000	1.598037000
9	0.230038000	2.134896000	2.165431000
6	3.491063000	2.210519000	0.089249000
6	2.587333000	1.072844000	0.346230000
6	3.060336000	-0.225646000	0.490189000
6	2.483198000	-1.487510000	0.268536000
6	3.244889000	-2.649634000	-0.150002000
6	2.382568000	-3.648121000	-0.537250000
6	1.075549000	-3.138696000	-0.313110000
6	-0.186860000	-3.741774000	-0.473607000
6	-1.381213000	-3.045207000	-0.210941000
6	-2.736254000	-3.415842000	-0.423826000
6	-3.484624000	-2.329135000	-0.041477000
6	-2.600365000	-1.248142000	0.364735000
6	-3.051077000	0.067701000	0.556156000
6	-2.474347000	1.327265000	0.413941000
6	-3.291308000	2.489581000	-0.007639000
6	-2.404825000	3.410523000	-0.551971000
6	-1.093620000	2.912291000	-0.331611000
6	0.157878000	3.511228000	-0.638132000
6	1.352786000	2.787296000	-0.376673000
6	2.699557000	3.227181000	-0.436442000
7	1.298394000	1.497597000	0.118455000
7	1.161040000	-1.848368000	0.188481000
7	-1.325903000	-1.750298000	0.275714000
7	-1.157682000	1.678989000	0.276847000
1	2.646571000	-4.607947000	-0.946138000
1	-3.103901000	-4.352831000	-0.805660000
1	3.037098000	4.198977000	-0.755092000
1	4.133919000	-0.298356000	0.561076000
1	-4.128364000	0.093115000	0.587914000
1	-2.651610000	4.340684000	-1.036297000
7	0.182524000	4.780948000	-1.199277000

6	-0.506723000	5.907020000	-0.577835000
1	0.225499000	6.676133000	-0.291003000
1	-1.228258000	6.367912000	-1.266061000
1	-1.028150000	5.583796000	0.322475000
6	1.093808000	5.145318000	-2.271522000
1	0.544602000	5.723894000	-3.025678000
1	1.930471000	5.768946000	-1.920525000
1	1.493392000	4.250688000	-2.750159000
7	-4.654067000	2.562908000	0.005548000
6	-5.444734000	2.094269000	1.150544000
1	-4.800550000	1.644840000	1.905791000
1	-5.956336000	2.947691000	1.614448000
1	-6.192797000	1.358595000	0.837648000
6	-5.293380000	3.597867000	-0.794252000
1	-5.191899000	4.595742000	-0.337909000
1	-4.858944000	3.620468000	-1.797061000
1	-6.357395000	3.367045000	-0.886580000
7	4.846448000	2.217152000	0.235303000
6	5.608255000	3.278566000	-0.407843000
1	5.533960000	4.231525000	0.139505000
1	6.660060000	2.984794000	-0.448396000
1	5.254314000	3.427866000	-1.431150000
6	5.511177000	1.590732000	1.384724000
1	4.778603000	1.141962000	2.055088000
1	6.219757000	0.820927000	1.061199000
1	6.054910000	2.357722000	1.950569000
7	4.679983000	-2.795977000	-0.249465000
8	5.105600000	-3.813532000	-0.798633000
8	5.409804000	-1.916949000	0.233060000
7	-0.267957000	-5.111134000	-0.966576000
8	-1.240035000	-5.791379000	-0.625054000
8	0.635278000	-5.519349000	-1.704168000
7	-4.927878000	-2.326299000	-0.131142000
8	-5.561050000	-1.387792000	0.375596000
8	-5.458845000	-3.282381000	-0.698810000

E= –2329.7830595 a.u.
H= –2329.7830595 + 0.563142 (thermal correction 6-31+G**) a. u.
G= –2329.7830595 + 0.448066 (thermal correction 6-31+G**) a. u.

5d– akamptisomer *amplo, parvo* ($\boldsymbol{t_1}$)

9	-0.248803000	-0.051259000	-1.221466000
5	-1.545573000	0.595839000	1.078992000
5	0.423105000	-0.281271000	0.010137000
8	-0.276961000	0.069675000	1.178321000
9	-2.124211000	0.915245000	2.322283000

6	-1.336084000	4.039340000	-0.514845000
6	-0.633170000	2.852941000	-0.049696000
6	0.769250000	2.826046000	-0.021418000
6	1.807520000	1.867318000	0.014573000
6	3.220975000	2.177123000	-0.010131000
6	3.944968000	1.009517000	-0.070121000
6	3.011093000	-0.072122000	-0.086616000
6	3.195462000	-1.493651000	-0.110496000
6	2.042147000	-2.303403000	0.026136000
6	1.883122000	-3.740587000	0.165699000
6	0.553228000	-4.002593000	0.313223000
6	-0.163339000	-2.746516000	0.269883000
6	-1.568493000	-2.691666000	0.333876000
6	-2.595812000	-1.726979000	0.251114000
6	-3.946113000	-2.119685000	-0.046848000
6	-4.716327000	-0.978168000	-0.207450000
6	-3.797294000	0.152477000	0.000878000
6	-3.937083000	1.529507000	-0.191262000
6	-2.811999000	2.375827000	-0.168989000
6	-2.668387000	3.741221000	-0.603245000
7	-1.589283000	1.897247000	0.194646000
7	1.752706000	0.497319000	-0.029209000
7	0.774728000	-1.768133000	0.091092000
7	-2.564966000	-0.357736000	0.332265000
1	2.669548000	-4.475672000	0.169910000
1	0.088427000	-4.970146000	0.447997000
1	1.180112000	3.821273000	-0.137233000
1	-1.987341000	-3.695070000	0.360636000
1	-4.884491000	1.963193000	-0.468971000
6	4.574585000	-2.191752000	-0.270950000
6	5.779566000	-1.258666000	-0.522567000
6	4.919917000	-2.978063000	1.023415000
6	4.532675000	-3.128614000	-1.511769000
1	5.635773000	-0.617334000	-1.396200000
1	6.021151000	-0.644337000	0.347788000
1	6.656504000	-1.883175000	-0.718691000
1	4.179539000	-3.732647000	1.285313000
1	5.886276000	-3.480182000	0.902814000
1	4.999237000	-2.289164000	1.870868000
1	5.487842000	-3.656830000	-1.604503000
1	3.740076000	-3.873692000	-1.475598000
1	4.383645000	-2.537177000	-2.421461000
7	-6.036875000	-0.932923000	-0.575340000
6	-6.759219000	-2.200499000	-0.550937000
1	-7.785213000	-2.041306000	-0.884234000
1	-6.292353000	-2.915861000	-1.236158000
1	-6.773385000	-2.644069000	0.456381000

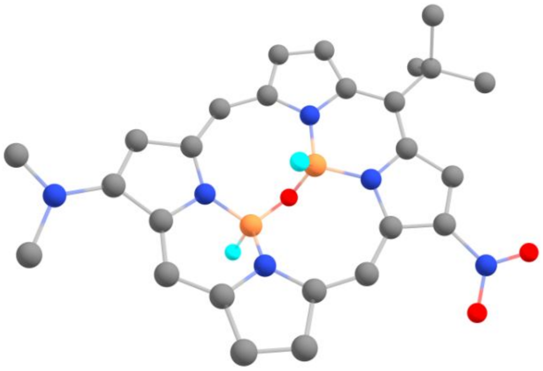

6	-6.813224000	0.296253000	-0.444892000
1	-6.561280000	1.006968000	-1.239334000
1	-7.872986000	0.059055000	-0.542346000
1	-6.658591000	0.780051000	0.528789000
1	-3.468929000	4.372558000	-0.964105000
1	-0.844983000	4.960930000	-0.797787000
7	3.870127000	3.465790000	0.025593000
8	5.104606000	3.478393000	-0.032838000
8	3.177452000	4.488075000	0.110947000
1	5.015627000	0.980184000	-0.079269000
1	-4.258358000	-3.143977000	-0.181756000

E= –1809.856366 a.u.
H= –1809.856366 + 0.517685 (thermal correction 6-31+G**) a. u.
G= –1809.856366 + 0.424271 (thermal correction 6-31+G**) a. u.

5d– akamptisomer *parvo, amplo* (***t***$_2$)

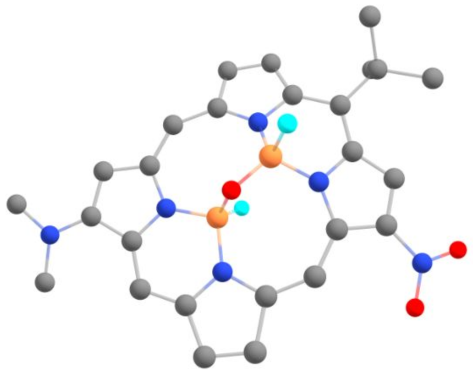

9	-1.246541000	-0.756912000	2.343539000
5	1.300537000	0.413960000	0.016399000
5	-0.663725000	-0.446392000	1.100251000
8	0.613088000	0.055572000	1.192151000
9	0.600841000	0.205932000	-1.204591000
6	1.508845000	4.140059000	0.377166000
6	0.748671000	2.906125000	0.295866000
6	-0.647797000	2.860787000	0.335722000
6	-1.673777000	1.888555000	0.209677000
6	-3.027573000	2.228168000	-0.194778000
6	-3.771067000	1.077213000	-0.314447000
6	-2.918628000	-0.005528000	0.036434000
6	-3.136826000	-1.419752000	-0.034806000
6	-1.989722000	-2.243320000	-0.084700000
6	-1.816304000	-3.615247000	-0.511143000
6	-0.479794000	-3.882940000	-0.484806000
6	0.219479000	-2.687403000	-0.053176000
6	1.633538000	-2.680427000	-0.032745000
6	2.683300000	-1.745030000	-0.028973000
6	4.069348000	-2.113609000	-0.088468000
6	4.831450000	-0.954589000	-0.139597000
6	3.874652000	0.166098000	-0.098540000
6	4.038344000	1.553084000	-0.044185000
6	2.922391000	2.402058000	0.083209000
6	2.837728000	3.832417000	0.249148000
7	1.660528000	1.899646000	0.104946000
7	-1.681211000	0.522169000	0.357355000
7	-0.736867000	-1.741479000	0.206421000
7	2.619200000	-0.375946000	-0.053003000
1	-2.590465000	-4.285181000	-0.847070000

1	0.009536000	-4.797619000	-0.792248000
1	-1.071007000	3.856627000	0.370716000
1	2.020699000	-3.691460000	-0.137112000
1	5.018032000	2.003589000	-0.053628000
6	-4.546174000	-2.062889000	-0.089676000
6	-4.849785000	-2.597871000	-1.512029000
6	-4.613350000	-3.206846000	0.961480000
6	-5.703588000	-1.106113000	0.290531000
1	-4.821765000	-1.781142000	-2.240849000
1	-4.140279000	-3.358202000	-1.841286000
1	-5.852666000	-3.038862000	-1.533828000
1	-4.455547000	-2.803342000	1.966902000
1	-5.605182000	-3.670221000	0.929902000
1	-3.874197000	-3.990865000	0.805616000
1	-6.594518000	-1.706672000	0.497262000
1	-5.478808000	-0.521023000	1.186557000
1	-5.974181000	-0.426530000	-0.519222000
7	6.196681000	-0.880126000	-0.186360000
6	6.895803000	0.339343000	-0.569247000
1	7.918580000	0.089289000	-0.854224000
1	6.416436000	0.821762000	-1.429324000
1	6.945194000	1.057572000	0.259089000
6	6.931753000	-2.136903000	-0.163315000
1	6.631721000	-2.734904000	0.704413000
1	6.753459000	-2.732923000	-1.071554000
1	8.000031000	-1.933603000	-0.080503000
1	3.680616000	4.509571000	0.271762000
1	1.067454000	5.117994000	0.515590000
7	-3.590921000	3.516963000	-0.522231000
8	-2.901624000	4.537169000	-0.389334000
8	-4.763612000	3.534940000	-0.913142000
1	4.425340000	-3.132445000	-0.102182000
1	-4.781444000	1.046889000	-0.673476000

E= –1809.8578364 a.u.
H= –1809.8578364 + 0.51737 (thermal correction 6-31+G**) a. u.
G= –1809.8578364 + 0.423349 (thermal correction 6-31+G**) a. u.

5d– transition state (*TS*)

9	0.468651000	-0.441124000	-2.075232000
5	-1.478685000	0.560296000	0.746407000
5	0.589813000	-0.384486000	-0.665450000
8	-0.445924000	0.085608000	0.036694000
9	-1.341574000	0.627030000	2.151898000
6	-1.498567000	4.047521000	-0.630033000
6	-0.769842000	2.855935000	-0.204043000

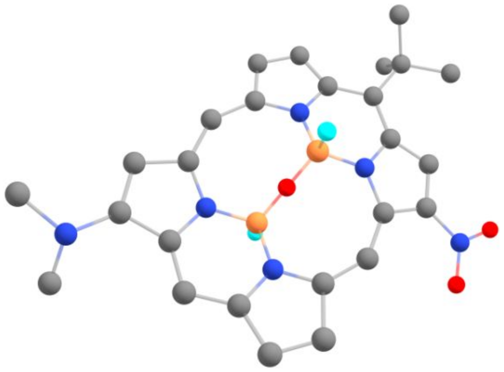

6	0.641767000	2.782323000	-0.254642000
6	1.736352000	1.853488000	-0.155027000
6	3.125126000	2.225092000	0.060622000
6	3.916888000	1.093091000	0.090066000
6	3.058018000	-0.028194000	-0.130075000
6	3.264205000	-1.456668000	-0.077660000
6	2.119191000	-2.297659000	0.033546000
6	1.933114000	-3.692250000	0.399420000
6	0.585716000	-3.924727000	0.479540000
6	-0.119603000	-2.689248000	0.185564000
6	-1.550817000	-2.613059000	0.199389000
6	-2.631999000	-1.689388000	0.191234000
6	-4.004766000	-2.102481000	-0.029053000
6	-4.816174000	-0.981969000	-0.038848000
6	-3.924580000	0.172339000	0.167988000
6	-4.100988000	1.555269000	-0.014196000
6	-2.986896000	2.428939000	-0.087313000
6	-2.842949000	3.784147000	-0.577459000
7	-1.744638000	1.959048000	0.174443000
7	1.795379000	0.490593000	-0.290874000
7	0.858972000	-1.780626000	-0.106143000
7	-2.678282000	-0.327614000	0.380335000
1	2.707444000	-4.409771000	0.617942000
1	0.106275000	-4.851761000	0.765370000
1	1.045964000	3.776347000	-0.411405000
1	-1.952273000	-3.625633000	0.215158000
1	-5.081838000	1.946827000	-0.250932000
6	4.667453000	-2.110425000	-0.098752000
6	4.697925000	-3.202656000	-1.204070000
6	5.825150000	-1.146271000	-0.445182000
6	4.996192000	-2.719928000	1.289192000
1	3.946708000	-3.979718000	-1.069477000
1	4.530580000	-2.750425000	-2.187267000
1	5.681965000	-3.683546000	-1.213945000
1	6.074742000	-0.479636000	0.382564000
1	6.721643000	-1.740638000	-0.645866000
1	5.615022000	-0.549068000	-1.336911000
1	5.994868000	-3.170045000	1.266295000
1	4.992080000	-1.940404000	2.058042000
1	4.286698000	-3.488998000	1.595731000
1	-1.024418000	4.955646000	-0.978419000
1	-3.653426000	4.433817000	-0.878558000
1	-4.310151000	-3.125053000	-0.196106000
1	4.969257000	1.111818000	0.299110000
7	3.683884000	3.536786000	0.282953000
8	4.905985000	3.611209000	0.452206000
8	2.930144000	4.518809000	0.285432000

7	-6.169421000	-0.901768000	-0.308336000
6	-6.813236000	-2.132705000	-0.747793000
1	-7.826157000	-1.902269000	-1.088035000
1	-6.256832000	-2.564506000	-1.583593000
1	-6.879726000	-2.881292000	0.060006000
6	-7.011259000	-0.097677000	0.583250000
1	-7.891422000	0.256696000	0.038354000
1	-7.348371000	-0.690465000	1.448920000
1	-6.463388000	0.762911000	0.962967000

E= –1809.8173 a.u.
H= –1809.8173 + 0.515685 (thermal correction 6-31+G**) a. u.
G= –1809.8173 + 0.423558 (thermal correction 6-31+G**) a. u.
f= 176.6142 *i*

5d– akamptisomer *amplo, amplo* (c_1)

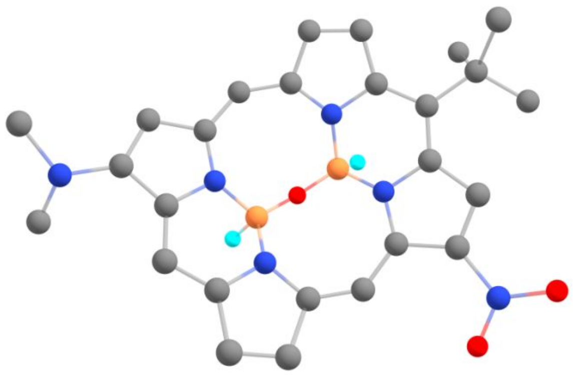

9	-2.461246000	0.974136000	2.185395000
5	0.758186000	-0.428877000	1.140244000
5	-1.649054000	0.592579000	1.089964000
8	-0.450928000	0.085880000	1.512592000
9	1.542449000	-0.774763000	2.267561000
6	0.429000000	-3.938169000	-0.210818000
6	-0.236852000	-2.717615000	0.182133000
6	-1.640184000	-2.705429000	0.277169000
6	-2.645636000	-1.738432000	0.175491000
6	-3.993119000	-2.097256000	-0.201051000
6	-4.700048000	-0.937404000	-0.436142000
6	-3.755574000	0.155936000	-0.217403000
6	-3.867089000	1.517515000	-0.477586000
6	-2.753984000	2.357402000	-0.327098000
6	-2.603165000	3.752547000	-0.632839000
6	-1.314722000	4.086491000	-0.316702000
6	-0.641028000	2.885008000	0.141727000
6	0.746807000	2.867425000	0.277648000
6	1.754441000	1.885327000	0.191375000
6	3.104452000	2.198607000	-0.234337000
6	3.792975000	1.029615000	-0.449113000
6	2.914310000	-0.038756000	-0.123682000
6	3.099827000	-1.451530000	-0.212676000
6	1.940253000	-2.253875000	-0.134950000
6	1.746393000	-3.655797000	-0.416512000
7	0.707912000	-1.726642000	0.235816000
7	-2.558740000	-0.372654000	0.215753000
7	-1.565079000	1.870046000	0.157236000
7	1.697838000	0.515978000	0.264644000
1	-0.072101000	-4.882993000	-0.373775000

1	-4.335597000	-3.114000000	-0.327168000
1	-3.372181000	4.386780000	-1.052411000
1	-0.824577000	5.041522000	-0.451904000
1	2.491899000	-4.348589000	-0.767990000
1	-2.046523000	-3.711417000	0.213376000
1	-4.787037000	1.927788000	-0.872899000
1	1.168939000	3.862733000	0.234731000
1	4.790349000	0.985613000	-0.839096000
6	4.484678000	-2.135674000	-0.376392000
6	5.708364000	-1.203029000	-0.214462000
1	5.868751000	-0.562997000	-1.084420000
1	5.644475000	-0.584567000	0.684774000
1	6.602326000	-1.827051000	-0.123277000
6	4.647479000	-3.197281000	0.750319000
1	5.616211000	-3.696552000	0.641696000
1	4.620742000	-2.709566000	1.730001000
1	3.874219000	-3.962874000	0.748321000
6	4.612719000	-2.779075000	-1.781960000
1	4.534093000	-2.010635000	-2.558294000
1	5.592804000	-3.259161000	-1.878469000
1	3.850440000	-3.530975000	-1.986207000
7	-5.998485000	-0.789440000	-0.895078000
6	-6.659329000	-2.010285000	-1.339728000
1	-7.600208000	-1.746212000	-1.829802000
1	-6.027309000	-2.532719000	-2.062423000
1	-6.885030000	-2.694199000	-0.503673000
6	-6.895558000	0.099064000	-0.144823000
1	-7.673237000	0.485184000	-0.810782000
1	-7.378525000	-0.440483000	0.685637000
1	-6.346640000	0.937120000	0.280548000
7	3.707230000	3.483336000	-0.507097000
8	4.834548000	3.478216000	-1.013445000
8	3.095135000	4.518284000	-0.212720000

E= –1809.8455106 a.u.
H= –1809.8455106 + 0.517104 (thermal correction 6-31+G**) a. u.
G= –1809.8455106 + 0.423796 (thermal correction 6-31+G**) a. u.

5e– akamptisomer *amplo, parvo* (***t***$_{1}$)

9	0.600509000	0.048643000	1.241392000
5	1.961708000	0.676433000	-1.032819000
5	-0.033285000	-0.195710000	-0.005672000
8	0.686809000	0.168327000	-1.156158000
9	2.571527000	0.977996000	-2.263769000
6	1.744278000	4.148722000	0.513686000
6	1.048833000	2.959231000	0.043525000
6	-0.347588000	2.920669000	-0.011292000

6	-1.384060000	1.952718000	-0.052606000
6	-2.764911000	2.329185000	-0.046730000
6	-3.538429000	1.165608000	0.001990000
6	-2.622157000	0.038498000	0.034052000
6	-2.809110000	-1.397126000	0.072063000
6	-1.661829000	-2.211614000	-0.050691000
6	-1.493874000	-3.650265000	-0.205240000
6	-0.162163000	-3.909036000	-0.323684000
6	0.553715000	-2.656088000	-0.250862000
6	1.964688000	-2.608200000	-0.274223000
6	2.983681000	-1.648983000	-0.170551000
6	4.357651000	-2.026726000	0.154696000
6	5.077650000	-0.868162000	0.346200000
6	4.159706000	0.236652000	0.120032000
6	4.328549000	1.602385000	0.314394000
6	3.217554000	2.468117000	0.241843000
6	3.069032000	3.840441000	0.654486000
7	2.006017000	1.992118000	-0.159077000
7	-1.363734000	0.585523000	-0.005233000
7	-0.384635000	-1.678741000	-0.089994000
7	2.942545000	-0.279991000	-0.249811000
1	-2.268922000	-4.390004000	-0.249156000
1	0.302992000	-4.875991000	-0.461400000
1	-0.766409000	3.918755000	0.094106000
1	2.375803000	-3.615057000	-0.274706000
1	5.284795000	1.995090000	0.639720000
6	-4.242738000	-1.961030000	0.238936000
6	-4.860480000	-1.386321000	1.549055000
6	-5.065416000	-1.645749000	-1.041925000
6	-4.369220000	-3.491796000	0.424213000
1	-4.394887000	-1.877658000	2.410090000
1	-4.736794000	-0.316833000	1.689351000
1	-5.934208000	-1.595731000	1.572677000
1	-4.683596000	-2.249729000	-1.871781000
1	-6.117217000	-1.903630000	-0.882090000
1	-5.030708000	-0.606717000	-1.357188000
1	-5.413408000	-3.706837000	0.671175000
1	-4.144017000	-4.047857000	-0.488049000
1	-3.755891000	-3.867840000	1.247398000
7	4.911601000	-3.315560000	0.349081000
7	6.377111000	-0.767930000	0.775899000
1	3.861240000	4.470217000	1.035432000
1	1.252451000	5.077981000	0.769271000
1	6.897624000	-1.635503000	0.717055000
1	6.899638000	0.048073000	0.490194000
1	4.686256000	-3.971690000	-0.391147000
1	4.680194000	-3.730219000	1.247647000

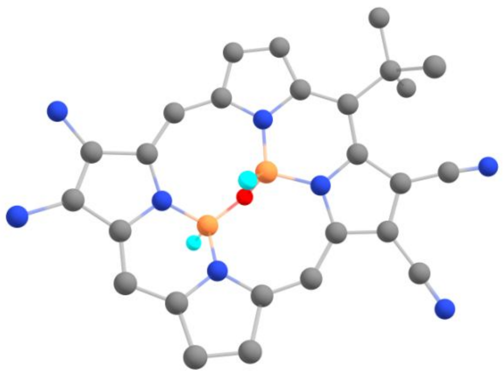

6	-4.955191000	1.258967000	0.007798000
7	-6.106916000	1.430534000	0.016989000
6	-3.248025000	3.662435000	-0.079350000
7	-3.604948000	4.770617000	-0.098479000

E= –1766.5512407a.u.
H= –1766.5512407 + 0.472259 (thermal correction 6-31+G**) a. u.
G= –1766.5512407 + 0.38052 (thermal correction 6-31+G**) a. u.

5e– akamptisomer *parvo, amplo* ($\boldsymbol{t_2}$)

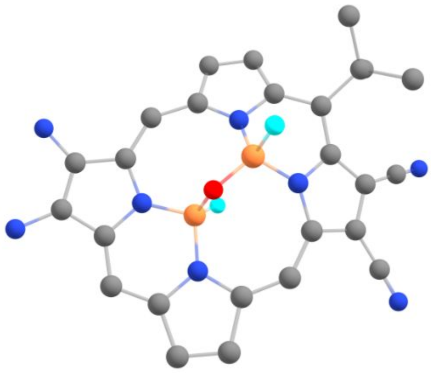

9	-0.838584000	-0.716373000	2.355951000
5	1.673890000	0.514532000	0.019407000
5	-0.276756000	-0.360566000	1.119261000
8	1.001051000	0.137915000	1.199401000
9	0.927471000	0.385392000	-1.185185000
6	1.996494000	4.228802000	0.446187000
6	1.206399000	3.014709000	0.352557000
6	-0.187359000	2.983710000	0.383261000
6	-1.224337000	2.016933000	0.251505000
6	-2.519311000	2.433857000	-0.215805000
6	-3.323129000	1.299301000	-0.350193000
6	-2.536285000	0.176477000	0.089485000
6	-2.796119000	-1.240010000	0.080621000
6	-1.670897000	-2.071839000	-0.085719000
6	-1.558627000	-3.407256000	-0.629697000
6	-0.229340000	-3.700023000	-0.703999000
6	0.521783000	-2.559564000	-0.210535000
6	1.937418000	-2.591304000	-0.233922000
6	2.999496000	-1.680873000	-0.192004000
6	4.398900000	-2.062119000	-0.309775000
6	5.152909000	-0.907332000	-0.286826000
6	4.223215000	0.206698000	-0.153118000
6	4.450609000	1.574323000	-0.040929000
6	3.365428000	2.458594000	0.126413000
6	3.316686000	3.888384000	0.314337000
7	2.092388000	1.985694000	0.150983000
7	-1.296675000	0.656822000	0.429856000
7	-0.393014000	-1.612045000	0.162741000
7	2.958170000	-0.312619000	-0.121964000
1	-2.371378000	-4.011892000	-0.997875000
1	0.221292000	-4.590953000	-1.120746000
1	-0.600173000	3.989281000	0.416287000
1	2.296797000	-3.602337000	-0.414259000
1	5.460589000	1.966167000	-0.047632000
6	-4.206014000	-1.845280000	0.285926000
6	-4.900314000	-2.140656000	-1.064581000

6	-4.103131000	-3.158799000	1.111866000
6	-5.085716000	-0.910396000	1.155571000
1	-5.043751000	-1.234748000	-1.656096000
1	-4.317126000	-2.848810000	-1.661430000
1	-5.886528000	-2.581352000	-0.881783000
1	-3.526151000	-2.998801000	2.027865000
1	-5.112001000	-3.475998000	1.393254000
1	-3.649438000	-3.984854000	0.567072000
1	-6.004274000	-1.441314000	1.423531000
1	-4.566746000	-0.642790000	2.081404000
1	-5.393285000	0.001846000	0.652338000
1	4.175962000	4.544143000	0.342754000
1	1.582265000	5.216811000	0.596635000
6	-4.579466000	1.329205000	-1.007912000
7	-5.591059000	1.401131000	-1.580458000
6	-2.872113000	3.761598000	-0.567925000
7	-3.135052000	4.864927000	-0.832578000
7	4.968931000	-3.355420000	-0.434355000
1	4.604389000	-3.880244000	-1.223767000
1	4.876716000	-3.918515000	0.406383000
7	6.520560000	-0.817746000	-0.309009000
1	6.935390000	0.019973000	-0.690187000
1	6.990575000	-1.677968000	-0.564434000

E= –1766.5551785 a.u.
H= –1766.5551785 + 0.471691 (thermal correction 6-31+G**) a. u.
G= –1766.5551785 + 0.380174 (thermal correction 6-31+G**) a. u.

5e– transition state (*TS*)

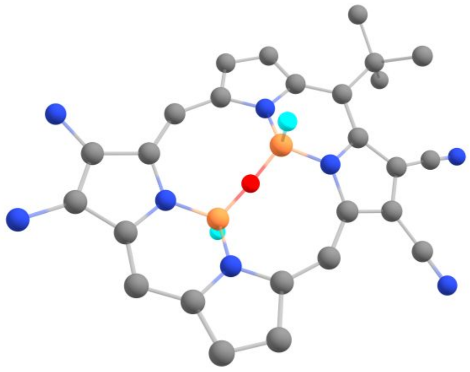

9	-0.070850000	-0.380642000	2.096622000
5	1.882038000	0.627784000	-0.711916000
5	-0.199092000	-0.302155000	0.692185000
8	0.817766000	0.203878000	-0.014589000
9	1.757807000	0.690721000	-2.119077000
6	1.975393000	4.142447000	0.581716000
6	1.225782000	2.950288000	0.198176000
6	-0.184192000	2.882480000	0.248395000
6	-1.286530000	1.955488000	0.170115000
6	-2.628354000	2.403269000	-0.086662000
6	-3.476864000	1.285491000	-0.132415000
6	-2.661389000	0.117118000	0.140054000
6	-2.890432000	-1.322255000	0.123040000
6	-1.760807000	-2.167741000	-0.042596000
6	-1.601686000	-3.540598000	-0.502743000
6	-0.262444000	-3.788406000	-0.619274000
6	0.470837000	-2.588694000	-0.251699000

6	1.902589000	-2.547194000	-0.271315000
6	2.997818000	-1.649030000	-0.216213000
6	4.381658000	-2.084688000	-0.033410000
6	5.184098000	-0.959933000	0.032200000
6	4.309850000	0.200994000	-0.125911000
6	4.529584000	1.576088000	0.025883000
6	3.434140000	2.480565000	0.087707000
6	3.315606000	3.853113000	0.533436000
7	2.184541000	2.023034000	-0.148405000
7	-1.399536000	0.596685000	0.328918000
7	-0.485263000	-1.679714000	0.103006000
7	3.053240000	-0.280957000	-0.331082000
1	-2.388022000	-4.220490000	-0.778627000
1	0.195311000	-4.699798000	-0.980764000
1	-0.583642000	3.886879000	0.383501000
1	2.280087000	-3.566170000	-0.349990000
1	5.528725000	1.945500000	0.225164000
6	-4.319201000	-1.896926000	0.267755000
6	-4.347818000	-3.383520000	0.711047000
6	-5.063106000	-1.145119000	1.405899000
6	-5.074816000	-1.805102000	-1.081673000
1	-4.164483000	-4.076115000	-0.110779000
1	-3.633990000	-3.588399000	1.514099000
1	-5.349991000	-3.611324000	1.085700000
1	-5.138364000	-0.070913000	1.266431000
1	-6.085578000	-1.528243000	1.479139000
1	-4.560696000	-1.325843000	2.362151000
1	-6.092737000	-2.191913000	-0.961766000
1	-5.153375000	-0.788037000	-1.461119000
1	-4.564001000	-2.410898000	-1.837565000
7	4.895462000	-3.396731000	0.131853000
1	4.647418000	-4.024630000	-0.626666000
1	4.631601000	-3.825316000	1.014729000
7	6.525662000	-0.928050000	0.307418000
1	7.064749000	-0.153698000	-0.051897000
1	6.985662000	-1.829044000	0.253207000
6	-4.845534000	1.419109000	-0.479699000
7	-5.960214000	1.599033000	-0.765659000
6	-3.009124000	3.751328000	-0.311742000
7	-3.286301000	4.870599000	-0.474762000
1	1.520988000	5.070458000	0.903186000
1	4.137390000	4.499390000	0.809878000

E= –1766.5126439a.u.
H= –1766.5126439 + 0.470262 (thermal correction 6-31+G**) a. u.
G= –1766.5126439 + 0.378926 (thermal correction 6-31+G**) a. u.
f= 174.9268 *i*

5e– akamptisomer *amplo, amplo* (c_1)

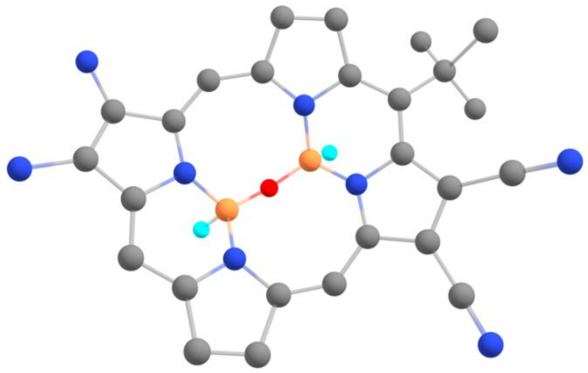

9	-2.957717000	0.784751000	2.111183000
5	0.369448000	-0.384439000	1.140094000
5	-2.070105000	0.566154000	1.028663000
8	-0.862500000	0.097110000	1.472181000
9	1.102570000	-0.796647000	2.275900000
6	0.231390000	-3.731656000	-0.656749000
6	-0.498682000	-2.600355000	-0.128056000
6	-1.905599000	-2.654682000	-0.063863000
6	-2.931813000	-1.716794000	-0.075695000
6	-4.292434000	-2.086188000	-0.454657000
6	-4.983761000	-0.917721000	-0.697323000
6	-4.073089000	0.167963000	-0.422331000
6	-4.264991000	1.540462000	-0.529350000
6	-3.207896000	2.418699000	-0.242792000
6	-3.105599000	3.840828000	-0.425701000
6	-1.834319000	4.191980000	-0.065054000
6	-1.125143000	2.980285000	0.308961000
6	0.259606000	2.976885000	0.431603000
6	1.290747000	2.017108000	0.291103000
6	2.575423000	2.441296000	-0.193799000
6	3.349262000	1.301407000	-0.421678000
6	2.558192000	0.172411000	-0.019197000
6	2.812897000	-1.237630000	-0.071265000
6	1.677010000	-2.058087000	-0.196695000
6	1.553624000	-3.401577000	-0.702509000
7	0.403800000	-1.603652000	0.127825000
7	-2.864355000	-0.348005000	0.004939000
7	-2.017497000	1.937866000	0.235979000
7	1.326435000	0.650666000	0.379748000
1	-0.231751000	-4.642285000	-1.012271000
1	-3.888422000	4.479891000	-0.810750000
1	-1.376863000	5.170919000	-0.121240000
1	2.350359000	-3.993471000	-1.122127000
1	-2.274575000	-3.657816000	-0.258256000
1	-5.202125000	1.944134000	-0.894314000
1	0.661765000	3.986320000	0.422732000
6	4.226979000	-1.852996000	0.088015000
6	5.135072000	-0.933680000	0.946263000
1	5.442957000	-0.020735000	0.445118000
1	4.639179000	-0.669907000	1.885586000
1	6.053477000	-1.476634000	1.188775000
6	4.144131000	-3.174583000	0.904711000
1	5.158909000	-3.484040000	1.172386000
1	3.575476000	-3.026673000	1.827748000
1	3.694486000	-4.001642000	0.358677000

6	4.882632000	-2.134830000	-1.283785000
1	5.015204000	-1.221770000	-1.867243000
1	5.870956000	-2.583005000	-1.133530000
1	4.279787000	-2.832767000	-1.873449000
6	2.935551000	3.778987000	-0.496897000
7	3.204218000	4.888837000	-0.726263000
6	4.574277000	1.342016000	-1.136558000
7	5.554593000	1.425210000	-1.759548000
7	-4.715841000	-3.396372000	-0.685779000
1	-4.726352000	-4.009926000	0.120061000
1	-5.551016000	-3.495212000	-1.247177000
7	-6.274731000	-0.790750000	-1.195392000
1	-6.910606000	-1.526440000	-0.910015000
1	-6.698377000	0.118764000	-1.066478000

E= –1766.5417107 a.u.

H= –1766.5417107 + 0.471311 (thermal correction 6-31+G**) a. u.
G= –1766.5417107 + 0.378677 (thermal correction 6-31+G**) a. u.
